# Reducing skin microbiome exposure impacts through swine farm biosecurity

**DOI:** 10.1093/gigascience/giaf062

**Published:** 2025-07-26

**Authors:** Ilya B Slizovskiy, Tara N Gaire, Peter M Ferm, Carissa A Odland, Scott A Dee, Joel Nerem, Jonathan E Bravo, Alejandro D Kimball, Christina Boucher, Noelle R Noyes

**Affiliations:** Purdue Applied Microbiome Sciences Program, Purdue University, West Lafayette, IN 47907, USA; Veterinary Clinical Sciences Department, College of Veterinary Medicine, Purdue University, West Lafayette, IN 47907, USA; Department of Veterinary Population Medicine, College of Veterinary Medicine, University of Minnesota, St. Paul, MN 55108, USA; Department of Veterinary Population Medicine, College of Veterinary Medicine, University of Minnesota, St. Paul, MN 55108, USA; Department of Veterinary Population Medicine, College of Veterinary Medicine, University of Minnesota, St. Paul, MN 55108, USA; Pipestone Veterinary Services, Pipestone, MN 56164, USA; Pipestone Applied Research, Pipestone, MN 56164, USA; Pipestone Applied Research, Pipestone, MN 56164, USA; Department of Computer and Information Science and Engineering, Herbert Wertheim College of Engineering, University of Florida, Gainesville, FL 32611, USA; Regenstrief Center for Healthcare Engineering, Purdue University, West Lafayette, IN 47904, USA; Department of Computer and Information Science and Engineering, Herbert Wertheim College of Engineering, University of Florida, Gainesville, FL 32611, USA; Department of Veterinary Population Medicine, College of Veterinary Medicine, University of Minnesota, St. Paul, MN 55108, USA

**Keywords:** metagenomics, microbiome, antimicrobial resistance, mobile genetic elements, farmworkers, public health

## Abstract

**Background:**

Livestock work is unique due to worker exposure to animal-associated microbiomes within the workplace. Swine workers are a unique cohort within the US livestock labor force, as they have direct daily contact with pigs and undertake mandatory biosecurity interventions. However, investigating this occupational cohort is challenging, particularly within tightly regulated commercial swine operations. Thus, little is known about the impacts of animal exposure and biosecurity protocols on the swine worker microbiome. We obtained unique samples from US swine workers, using a longitudinal study design to investigate temporal microbiome dynamics.

**Results:**

We observed a significant increase in bacterial DNA load on worker skin during the workday, with concurrent changes in the composition and abundance of microbial taxa, resistance genes, and mobile genetic elements. However, mandatory showering at the end of the workday partially returned the skin’s microbiome and resistome to their original state.

**Conclusions:**

These novel results from a human cohort demonstrate that existing biosecurity practices can ameliorate work-associated microbiome impacts.

## Introduction

Occupational exposures can significantly influence the microbiomes of workers and, in some cases, have been linked to health outcomes [1, 2]. People working with animals encounter a unique workplace microbiome with frequent exposure to animal microbiomes, either through direct contact or indirect exposures. The impact of animal exposure on human microbiomes has been demonstrated across several settings, including research facilities [3, 4] and livestock farms [5], as well as within homes [6]. For example, dairy and swine workers have more diverse oral and nasal bacterial taxa than non-livestock workers [7]. Additionally, the skin microbiome of livestock workers harbors a higher relative abundance of Pseudomonadota and a lower relative abundance of Actinomycetota and Bacteroidota compared to people with non-livestock occupations [8]. Livestock-associated bacteria and their antimicrobial resistance genes (ARGs) have been documented in agricultural worker cohorts, including farmers [9–11], veterinarians [12, 13], and abattoir workers [10, 14], and short-term visitation to swine farms has been linked to an enrichment in farm-associated bacteria and ARGs in the human gut [5]. These findings have been attributed to livestock exposure, but few studies have actually tracked daily animal exposure and on-site worker behavior, particularly on commercial farms [15, 16]. Such studies require careful consideration of workplace habits and exposures, and sampling must occur within the constraints of commercial livestock production. Given these challenges, detailed studies of livestock worker microbiomes are uncommon, and the specific influence of occupational exposures on livestock worker microbiomes remains poorly understood.

In the United States, swine workers are a unique cohort within the livestock labor force. Their job tasks involve intensive one-on-one animal handling, working within enclosed and climatically regulated facilities, with work duration ranging from 48 to 54 hours per week [17, 18]. These working conditions contrast markedly with other production systems; beef cattle and poultry are rarely handled by workers, and cattle work tends to occur in open-air facilities. Moreover, most workers in North American swine farms adhere to strict biosecurity measures to control pathogen transmission between and within farms [19, 20], including showering into and out of the farm, which may reduce transfer of microbes and ARGs between swine farms and the general public. However, the impact of mandatory showering on the likelihood of worker-mediated farm-to-community transmission is unknown.

**Figure 1:**
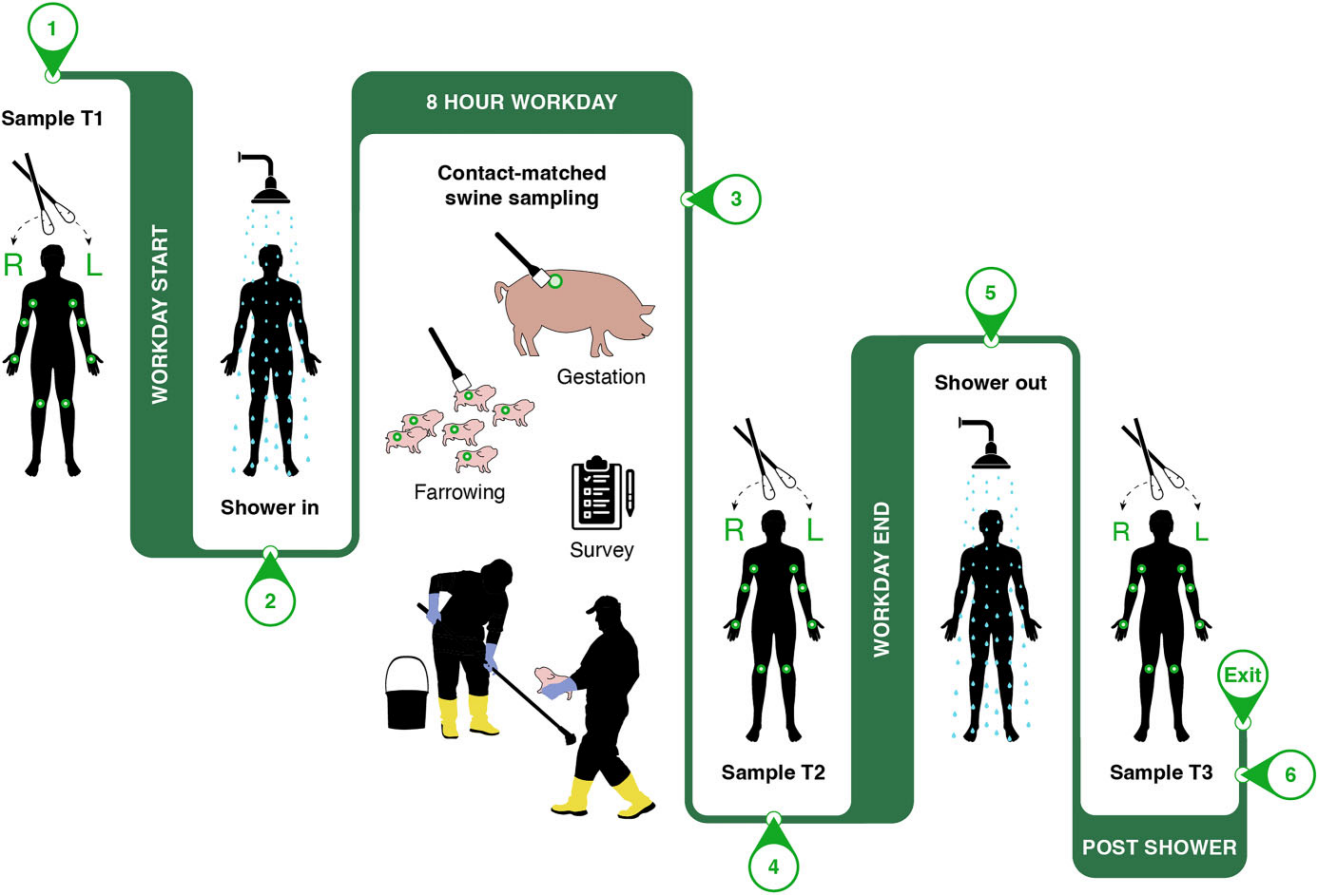
Study overview. Farmworkers from a commercial farrow-to-wean operation in the Midwestern United States were voluntarily enrolled into a single longitudinal microbiome sampling campaign during a typical 8-hour workday shift. For each worker, swab kits were used to self-collect samples from the epidermis in a standardized fashion by passing each swab across 4 body sites, achieving a single composite skin sample for the left and right body representing microbiomes from the manus, interdigital space, antecubital fossa, popliteal fossa, and axilla. Workers were asked to perform the first self-collection (“Sample **T1**”) prior to entry into the swine facility (**1**). Workers underwent mandatory showering prior to entry into the animal holding areas (**2**). During the day shift, workers were observed handling animals or working in specific animal pens, and dorsal skin swabs (from withers to tail base) were taken from contact-matched animals on a pooled multipen level. Additionally, a 15-minute questionnaire was administered to collect biometric, health, lifestyle, and occupational task performance information from each worker (**3**). In a similar fashion, self-collected skin samples were taken immediately upon conclusion of the workday (“Sample **T2**”) (**4**). Workers underwent mandatory showering procedures immediately after exiting the animal holding areas (**5**), and a third self-collection of samples was performed (“Sample **T3**”) after showering and immediately prior to exiting the farm facility (**6**).

We report on a longitudinal investigation of the swine worker microbiome–resistome on a commercial US swine facility, with sampling occurring as part of a normal workday that included showering as a mandatory biosecurity intervention (Fig. 1). We observed a significant increase in bacterial DNA load on worker skin during the workday, with concurrent changes in the composition and abundance of specific microbial taxa, ARGs, and mobile genetic elements (MGEs) that could harbor ARGs. We further observed that compulsory showering at the end of the workday reverted the skin microbiome and resistome to a baseline state, which differs from previous results that did not include showering in the study design [5]. These results suggest that occupational work in swine facilities can significantly impact workers’ skin microbiomes, but these impacts can be transient if biosecurity interventions are implemented. The relevancy of these findings for short- and long-term swine worker health requires further study. However, the observation that showering may dampen daily microbiome impacts have important public health implications, as it demonstrates that biosecurity protocols could be leveraged to minimize microbial transmission from farms to general communities.

## Results

### Individual skin microbiomes experience dramatic yet transient shifts during on-farm work with swine

**Figure 2:**
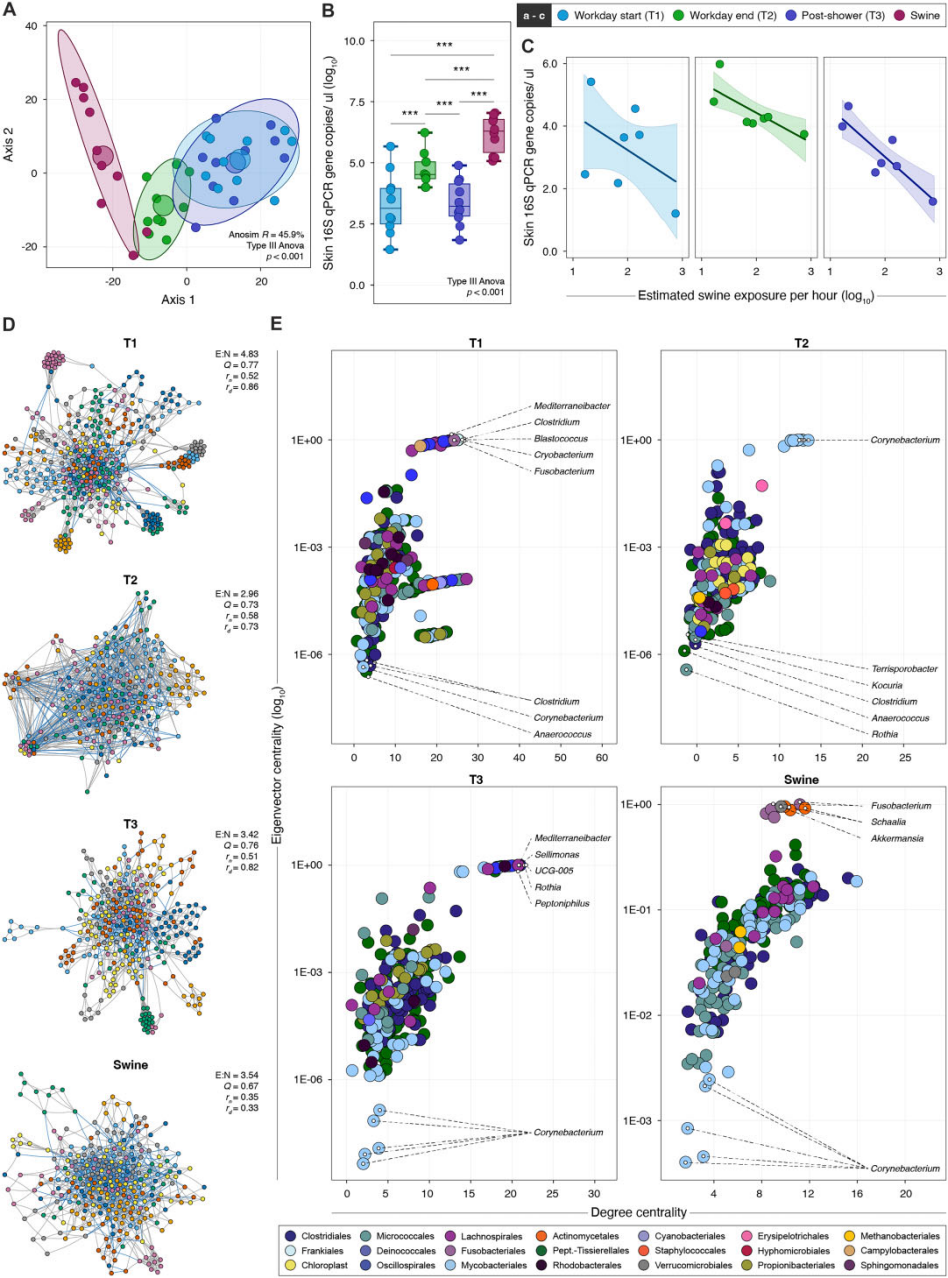
Changes in skin microbial load, microbiome composition, and community structure. (A) Genus-level worker skin microbiome β-diversity across collection phases (T1–T3) and contact-matched swine skin samples using principal component ordination of robust Aitchison compositions. Within-group centroids and 95% confidence intervals are depicted with a large circle and shaded ellipsoids, respectively. (B) Log_10_-normalized 16S rRNA quantitative PCR (PCR) copy number/μL (y-axis), stratified by collection phase (x-axis). *** indicates statistical significance (*P* < 0.001) of pairwise comparisons based on a linear regression model with Tukey’s adjustment for multiple comparisons. (C) General linear analysis of the log_10_-normalized 16S rRNA qPCR copy number/μL (y-axis) and log_10_-normalized hourly exposure to swine (x-axis) based on workers’ estimates from daily task assignments indicates a negative correlation (*P* = 0.01) across all collection phases. Shaded areas represent the 95% confidence interval around the linear trendline. (D) Worker skin microbiome networks across workday collection phases and for contact-matched swine were inferred from inverse covariance estimation for compositions based on centered-log ratios of subsetted ASV counts containing >100 counts and >10% prevalence per ASV per sample. Inferred networks consist of nodes representing ASVs colored by shared subcommunity membership. Edges between nodes represent a significant predicted positive (blue) or negative (gray) interaction. Reported topology characteristics include network connectivity based on the edge-to-node ratio (*E:N*), modularity (*Q*), subcommunity assortativity (*r_n_*), and degree assortativity (*r_d_*). (E) Scatterplots of microbial constituents from the corresponding networks are displayed based on the log_10_-normalized node eigenvector centrality (y-axis) and node degree centrality (x-axis). Taxa with the highest centrality measures (top right of the distribution) are considered critical connectors and major hubs in community networks and thus putative keystone taxa. ASV-level nodes are colored based on their taxonomic classification at the class level. Genus-level labels are displayed only for genera most likely to be keystone (i.e., >95th percentile of the plot distribution) (top right) and least likely to be keystone (i.e.,<5th percentile of the plot distribution) (bottom left).

16S rRNA sequencing and analysis was performed on 40 skin samples collected longitudinally from 10 healthy and predominantly male swine workers and contact-matched swine (SI Appendix SI Methods, Supplementary Note 1, Supplementary File 1). The skin microbiome composition underwent significant shifts in the course of a single 8-hour workday (analysis of similarities test [ANOSIM] *P* < 0.001; *R* = 57.6%, adjusted permutational multivariate analysis of variance [PERMANOVA] *P* < 0.001, Fig. 2A). Specifically, samples taken at the end of work but prior to showering (T2) had a significantly different composition than samples taken at the beginning of work (T1) (pairwise *R*^2^ = 22.0%; false discovery rate [FDR]–adjusted *P* = 0.004), and at the end of the workday following showering (T3), the microbiome underwent yet another shift relative to T2 (pairwise *R*^2^ = 18.6%; FDR-adjusted *P* = 0.004). However, the microbiome at T3 was not significantly different than at T1, suggesting at least a partial reversion of the skin microbial composition after showering (pairwise *R*^2^ = 4.9%; FDR-adjusted *P* = 0.12). These shifts corresponded with changes in skin-borne bacterial load as quantified using 16S gene concentration (copies/µL, Fig. 2B). Specifically, 16S gene concentration increased by ~200-fold from T1 to T2 but then decreased back to baseline levels at T3 (type III analysis of variance [ANOVA] *P* < 0.0001 with Tukey’s *post hoc* analysis and adjustment for FDR). There were no significant differences in average sequencing depth, sequencing quality, and taxonomic discovery rates between the 3 collection time points, suggesting that these technical factors did not significantly bias comparisons across collection phases (Supplementary Note 2, Supplementary Figs. S1, S2).

We next assessed associations between crude swine exposure rates and 16S gene concentration (i.e., bacterial load). There was large variation in estimated hourly swine exposure rates (Supplementary Table S1), and the hourly exposure density was found to be inversely correlated with bacterial load (estimate [SE]= −1.23 [0.44], *glm P* = 0.01, Fig. 2C). This relationship persisted at T2 and T3 (Fig. 2C). Though more granular and systematic exposure assessments are needed, these patterns suggest that differences in swine exposure density may be a proxy for different workday tasks that ultimately dictate levels of microbial biomass acquisition. For example, tasks such as feeding, health checks, and decontamination require walking through swine holding rooms but involve very little direct interfacing with animals and their by-products; such tasks would be classified as “high density,” but in reality, there may be less opportunity for direct acquisition of swine-related microbes. In contrast, activities such as vaccinating and obstetrical management require prolonged contact with individual pigs, but not necessarily moving through multiple swine holding rooms; thus, such tasks may have lower density but more opportunities for acquisition of swine-sourced microbes through direct contact.

A total of 6,840 unique amplicon sequence variants (ASVs) representing 356 distinct genera were recovered across all worker skin samples (Supplementary Fig. S2, Supplementary Datafiles 2, 3). The ASV diversity and the dominant phyla were consistent with findings from previous microbiome studies of human skin [21–23]. The relative abundances of dominant phyla remained largely similar across T1–T3, with the exception of Cyanobacteriota, which were more abundant on skin at T2 versus T1 and T3 (Supplementary Fig. S3a, b). Phylum-level and genus-level richness and evenness of the worker skin microbiome remained unchanged throughout the course of the day and did not significantly differ from contact-matched swine skin samples or the environmental samples (type III ANOVA-adjusted *P* > 0.1, Supplementary Fig. S4a–d). More than 400 genera were detected in at least 1 sample from each of the pairwise collection phases under comparison (T1 vs. T2 [*n* = 76], T2 vs. T3 [*n* = 76], and T1 vs. T3 [*n* = 75]), and fewer than 13% of these genera exhibited significant changes in relative abundance over the 3 time points (Supplementary Datafile 4, Supplementary Fig. S5). The relative abundances of *Methanobrevibacter, Negativibacillus, Butyricicoccus, Agathobacter*, and *Lachnospiraceae UCG-010* were significantly higher in T2 versus T1 samples. These genera inhabit mammalian oral cavity and digestive tracts. Genera with significantly higher relative abundance in T3 compared to T2 skin samples included taxa also primarily found as gastrointestinal microbiota. These include relatively new and unclassified rumenal genera such as *UCG-005* (*Oscillospiraceae*) and *Candidatus soleaferrea* (*Ruminococcaceae*) that have been previously reported in swine intestinal microbiomes but not functionally described [24, 25], and *CHKCI001* (*Lachnospiraceae*), which, to our knowledge, has hitherto been only described in chicken intestinal microbiomes [26, 27]. Other differentially abundant genera at T3 relative to T2 include *Agathobacter*, which exhibited the largest fold-change increase in relative abundance and was also found at a significantly greater relative abundance at T2 versus T1 (Supplementary Datafile 4). *Agathobacter* is a fiber-degrading and butyrate-producing keystone genus that has been described in growing piglets [28, 29]. We noted that a mixed population of genera were differentially abundant at T3 relative to T1, including intestinal genera such as *Negativibacillus*, which accounted for the greatest fold-change at T2 relative to T1; *Fastidiosipila*, a poorly characterized genus of methanogenic anaerobic bacteria [30]; and highly ubiquitous environmental bacteria *Brevundimonas*.

### The skin microbiome becomes unstructured and dominated by enteric and environmental microbes during on-farm work

Inferred association networks were explored using a compositional modeling approach to describe the topology and connectivity of microbial constituents in worker skin microbiomes across collection phases and in comparison to contact-matched animals (SI Appendix, SI Methods). Networks were constructed using standard cutoffs, and ASVs with >100 counts present in >10% sample prevalence were included, which represented 4.1–5.7% of all ASVs used as input into network generation, depending on the collection phase. The resulting networks (1 per collection phase) were each composed of a singular interconnected component, with the most connected network being T1 and the most sparse T2 (Fig. 2D).

Given the significant differences in network topology across T1, T2, and T3 (Supplementary Note 4), we further analyzed each network to identify the most dominant and interconnected genera (i.e., keystone taxa), as indicated by high eigenvector centrality and high node degree. At T1, keystone genera included a mixed population of bacteria not typically considered ubiquitous inhabitants of the human skin microbiome [31], including usually enteric members such as *Clostridium* and *Mediterraneibacter* and environmental bacteria such as *Blastococcus* and *Cryobacterium*. Other keystone genera included human skin commensals like *Anaerococcus* and *Fusobacterium*. However, *Corynebacterium*, well known as a ubiquitous inhabitant of both swine and human skin, was among the least influential members of the skin microbiome (Fig. 2E). Conversely, at T2, the interactions and modular domains within the skin microbiota became notably sparse, with *Corynebacterium* genera by far the most extensively represented among the keystone bacteria [21, 32, 33] (Fig. 2E). The least influential bacteria at T2 were typically enteric bacteria characteristic of mammalian intestinal microbiota, including *Clostridium, Rothia, Kocuria*, and *Terrisporobacter*. After showering and prior to exit from the swine facility (T3), dominant keystone genera were an admixture of Actinobacteria and Clostridia classes, encompassing genera associated with the mammalian gastrointestinal tract, including *Sellimonas, Rothia, Mediterraneibacter*, and UCG-005, as well as commensal human genera such as *Peptoniphilus* and Family XI of the Peptostreptococcales-Tissierellales order, previously identified among medically important inhabitants of human axillae [34]. We note that the swine skin ecological network was composed of dominant keystone genera *Fusobacterium, Schaalia*, and *Akkermansia*, typically associated with skin, oral mucous membrane, and intestinal microbiota. Notably, *Fusobacterium* in swine corresponded with the same genus found among keystone organisms at T1 in swine workers collected prior to entry into the main animal holding areas, while the least interconnected swine skin genera of *Corynebacterium* were observed in worker skin ecosystem dominance at T2, collected at the end of the workday.

### The worker skin resistome and mobilome shifted significantly during the workday and differed from that of contact-matched swine

Target enrichment was used to selectively capture and amplify all potential known ARGs and MGEs within the metagenomic DNA of all samples [35]. As with the microbiome, the resistome shifted significantly between each collection phase (ANOSIM *P* < 0.0001; *R* = 37.5%, Supplementary Fig. S6a), but there were no statistically significant differences in ARG group richness or Shannon’s diversity across the 3 collection phases (Supplementary Fig. S7). However, when normalized to the bacterial load as measured by the 16S rRNA gene copy number, the total ARG burden was significantly altered over the course of the workday. While bacterial load increased at the end of the workshift (T2) versus the start (Fig. 2B), we highlight in Supplementary Fig. S8a that workers at T1 and T3 carried a significantly greater total ARG abundance than at T2, though it was also observed that showering led to a significant reduction of total skin ARGs relative to T1 (type III ANOVA FDR-adjusted *P* < 0.001 with Tukey’s *post hoc* analysis). The T1 worker skin also harbored a greater ARG burden than the skin of pigs (type III ANOVA FDR-adjusted *P* = 0.005), and the total ARG burden in the environment did not significantly differ from that of any worker or swine skin sample. Similarly, plasmids, integrative conjugative elements (ICEs), insertional sequences (ISs), and other transposable elements (TEs) underwent significant shifts in β-diversity. Collection phase accounted for >40% of the variation in composition of ICEs and TEs, >20% of variation in composition of plasmids and IS, and ~9% of variation in composition of viruses and prophages (plasmid, ICE, TE, and IS ANOSIM *P* < 0.001; virus and prophage ANOSIM *P* = 0.02; Supplementary Fig. S6b). Plasmid, ICE, IS, and TE compositions were significantly different between T1 and T2, as well as between T2 and T3 (all PERMANOVA *P* < 0.001), while virus and prophage composition differed between the collection phases T1 and T2 (PERMANOVA *P* < 0.05). The observed MGE compositional shifts at T2 coincided with a greater relative abundance of ICE genes and a reduced relative abundance of plasmids, including plasmidic mechanisms of replication, transcription, translation, and regulation (Supplementary Fig. S6c, d). Between T1 and T3, there were significant differences in the composition of IS and TE genes (PERMANOVA *P* = 0.048 and 0.037, respectively), but not plasmids and ICEs. The worker resistome and MGE composition were significantly different from swine at all 3 collection phases, with the exception of viral and prophages at T2 (Supplementary Fig. S6b). Regarding the mobilome, the total MGE burden on the skin normalized to bacterial load (Supplementary Fig. S8b) was significantly higher at T1 and T3 compared to T2 (type III ANOVA, FDR-adjusted *P* < 0.0001, Tukey’s *post hoc* analysis). While the total MGE abundance on swine skin did not differ significantly from worker skin at T1 and T2, worker skin at T3 (after showering) harbored a greater number of MGE alleles than swine skin (type III ANOVA, FDR-adjusted *P* = 0.0268, Tukey’s *post hoc* analysis). In contrast to the resistome samples, environmental samples contained the highest abundance of MGEs observed in the study (type III ANOVA, FDR-adjusted *P* < 0.001, Tukey’s *post hoc* analysis).

### The clinically important fraction of the worker skin resistome varied throughout the workday and remained distinct from swine

We subsetted the MEGARes v2.0 database for 29 specific ARG groups previously identified as “clinically important” (i.e., priority ARGs) [36, 37]. In 41 of 42 enriched metagenomic samples, we detected 19 distinct priority ARG groups at gene coverage fraction >99.9%; 1 T2 sample did not contain any priority ARGs. These ARGs represented a low proportion of the total resistome across all worker (median [IQR] = 7.46% [8.23]) and swine samples (median [IQR] = 5.57% [4.27]), and their overall median relative abundance did not differ between workday collection phases and swine samples (type III ANOVA *P* > 0.05).

**Figure 3:**
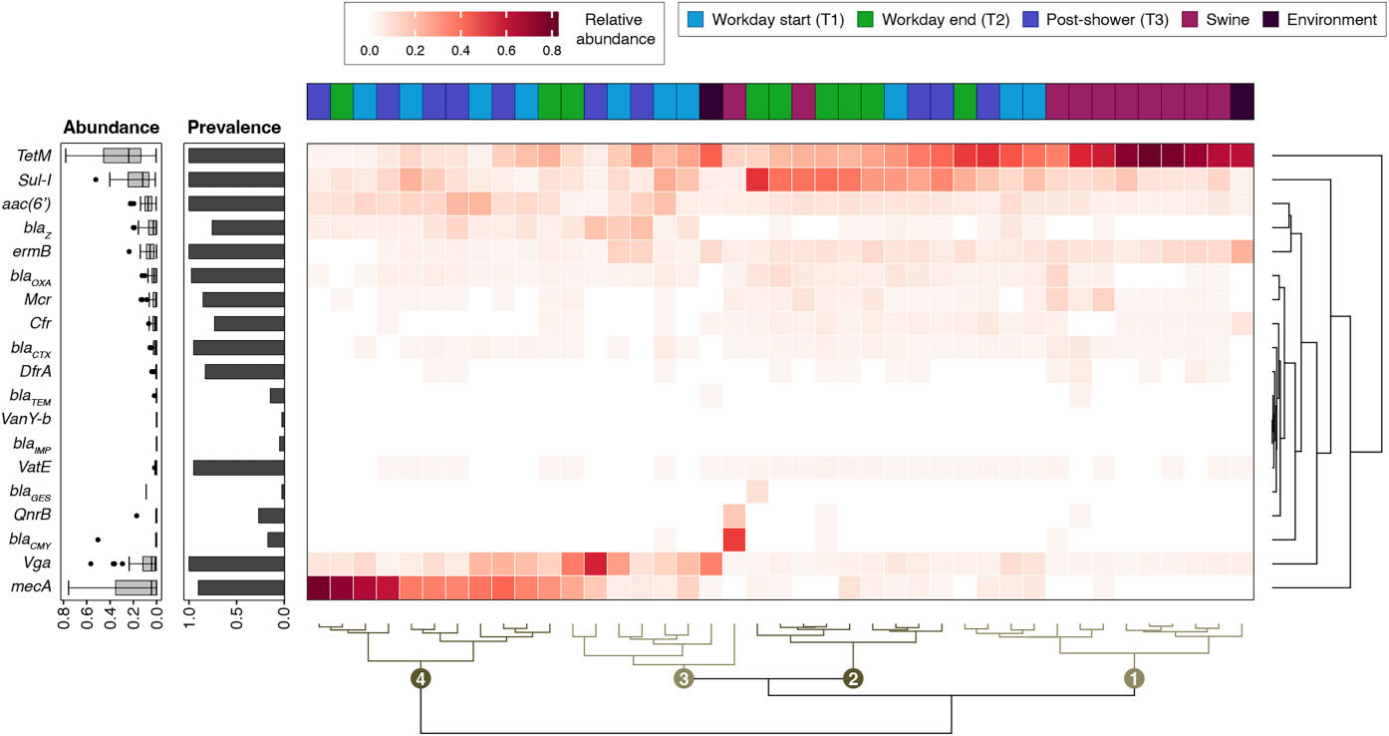
Occurrence of medically important ARGs on the skin of workers and swine. Unique medically important (i.e., priority) ARG alleles were identified at >99.9% alignment gene coverage across all collection phases (top ribbon annotation) and are displayed using a heatmap summarizing their sample-level relative abundance across each of the respective 19 ARG gene groups. The cladograms along the x-axis demonstrate the hierarchical clustering of samples according to their medically important resistome composition using optimal leaf sorting and Euclidean distances. Four major subclades are colored and numbered. Major ARG group prevalence and median abundance across study samples are summarized via the associated barplots and boxplots along the y-axis.

Tetracycline (*TetM*); sulfonamide (*Sul1*); multidrug resistance to classes of antibiotics, including lincosamides, streptogramins, and pleuromutilins (*Vga*); and methicillin (*mecA*) genes were the most prevalent and abundant of the priority ARGs (Fig. 3) and strongly influenced hierarchical clustering of samples into 4 major groups (i.e., subclades). Subclade 1 was characterized by high *TetM* relative abundance and contained 8 of the 10 swine samples and 1 or 2 worker samples from each of T1–T3. Subclade 2 contained 5 of the 9 T2 samples, 1 swine sample, and 1 T1 and 2 T3 samples, and it was characterized by a higher relative abundance of *Sul1*. Subclades 3 and 4 contained the majority of the T1 and T3 samples (i.e., 14/20), with subclade 3 defined by a higher abundance of *Vga* and subclade 4 containing the highest relative abundance of *mecA*.

The *mecA* gene, a methicillin resistance allele, consistently appeared in worker but rarely in swine samples (Fig. 3). *Staphylococcus aureus* in swine has been proposed as a key source of methicillin-resistant *S. aureus* (MRSA) in Danish swine workers [38], especially among workers of Danish pig herds in which historical MRSA prevalence exceeds 85%. However, recent reports suggest that *Staphylococcus* spp. are actually rare members of the porcine skin microbiome and typically account for <1% of the overall relative abundance of all Staphylococci [33]. We performed marker-based strain-level taxonomic profiling of metagenomic reads via StrainPhlAn to ascertain possible Staphylococcal sources of *mecA*. Strains of *Staphylococcus epidermidis, Staphylococcus haemolyticus, Staphylococcus hominis*, and *Staphylococcus equorum* were the only prevalent strains identified (>75% prevalence), and no *S. aureus* strains were identified at this prespecified prevalence level (Supplementary Datafile 9, Supplementary Fig. S9). Further phylogenetic analysis suggested that these Staphylococci were rarely shared between workers and swine, as most strains were tightly clustered by worker ID rather than collection phase (Supplementary Fig. S10). Major coagulase-negative Staphylococci (CoNS) are known carriers of *mecA*, and ~90% of US *S. epidermidis* clinical isolates in particular are methicillin resistant [39–41]. Taken together, these findings suggest that worker CoNS and not *S. aureus* were likely sources of *mecA* in this study.

### Post-work showering incompletely reverses changes in resistome and mobilome gene abundance

**Figure 4:**
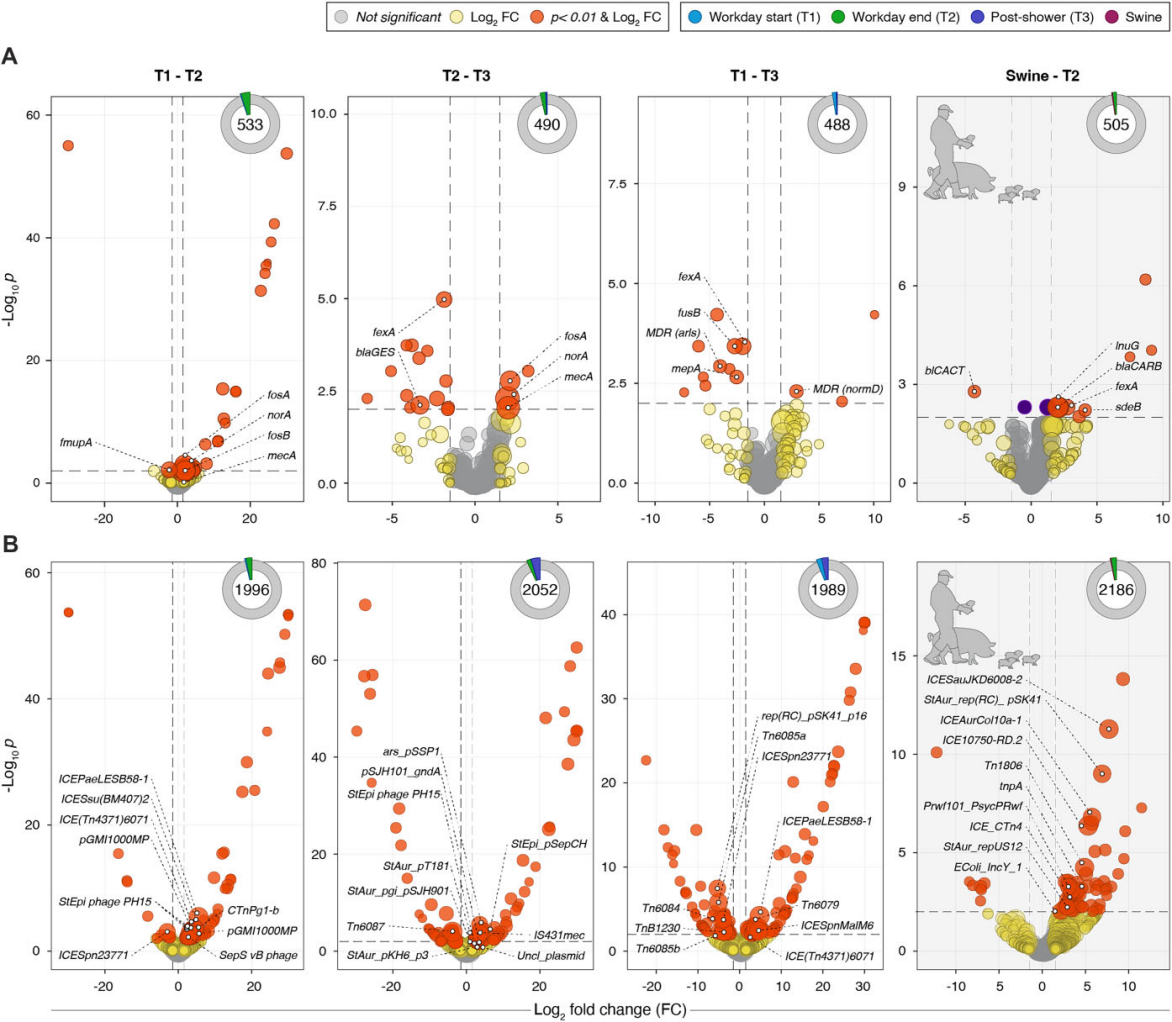
Differential abundance analysis across collection phases and at the interface period between workers and pigs. Volcano plots are used to visualize differential abundance of unique (A) ARG groups and (B) MGE accessions in log_2_-fold change (x-axis) and −log_10_ *P* value (y-axis) of the global worker skin resistome or mobilome between key workshift collection phases: workday start (T1) versus workday end (T2), workday end (T2) versus post-shower (T3), and workday start (T1) versus post-shower (T3). An additional comparison is made between workday end and swine skin samples representing the worker’s contact phase with animals (gray). Features with a significant shift in abundance (Wald’s *P* < 0.01 with FDR adjustment for ARG group and MGE accessions) are displayed above the horizontal line, while biologically significant fold-change is demarcated by vertical dashed lines at 1.5 log_2_-fold change. Labels are displayed for only the 5 most abundant ARG groups and 10 most abundant MGEs significantly amplified (log_2_-fold change >1.5 or <−1.5) at each phase comparison. For each volcano plot, an associated pie chart displays the number of unique ARG groups and MGE accessions common to each of the workshift collection phases compared, as well as the proportion of the total differentially abundant MGEs associated with each phase.

After controlling for worker age, gender, body mass index, smoking status, frequency of pork consumption, and host-removed sequencing depth, only 5.4%, 3.7%, and 2.7% of ARG groups exhibited significant changes in relative abundance at T1 versus T2, T2 versus T3, and T1 versus T3, respectively. Between T1 and T2 collection phases, most significantly changing ARG groups exhibited increases in abundance (i.e., 27/29 ARG groups, 93%) (Fig. 4A, Supplementary Datafile 10). Because log-fold differential abundance testing can produce false positives for low-count features, we highlighted only high-abundance ARG groups with a statistically significant change in relative abundance. For the T1 versus T2 comparison, this included ARG groups within the β-lactams (e.g., *mecA*), fosfomycins (e.g., *fosA* and *fosB*), and mupirocins (e.g., *mupA*). In contrast to the T1–T2 comparison, far fewer ARG groups experienced statistically significant changes in relative abundance from T2 to T3 (*n* = 18), and most of these (i.e., *n* = 13, or 72%) decreased in relative abundance, including β-lactams (e.g., *bla_GES_*), fusidic acids (e.g., *fusB*), phenicols (e.g., *cmlA*), sulfonamides (e.g., *sulIV*), MLS (e.g., *ereA*), and multidrug or multicompound classes (e.g., *fexA, ttgB, mexW, lmrD*). Two abundant ARG groups that exhibited significant increases in relative abundance at T2 compared to T1 also remained elevated after showering in T3, most notably *mecA*, and *norA*, the general drug and biocide efflux system of Staphylococci [42]. When compared to T1, 13 ARG groups at T3 were significantly differentially abundant, and 10 of these (77%) exhibited a significant decrease in relative abundance (Fig. 4A). For example, among the most abundant T3 ARGs, there was a significant decrease in multicompound and fusidic acid resistance (e.g., *fexA, mepA, fusB*), as well as multidrug resistance regulators and efflux systems (Supplementary Datafile 10).

Among MGEs with significant changes in relative abundance between collection phases, ICEs were most prominent (Fig. 4B). Specifically, *ICEPaeLESB58-1, ICETn4371*, and *ICESsu* (*BM407*) were more abundant in T2 versus T1 samples; the first two mobilize heavy metal resistance [43], while the latter mobilizes ARGs narrowly within *Streptococcus suis*, an emergent pathogen in humans that is considered a host-adapted swine pathobiont [43, 44]. Further strain analysis using gene markers confirmed the presence of *S. suis* in all swine and 9 of 10 of worker samples in each collection phase with a mean relative abundance of 10.2% at T1, 26.1% at T2, and 12.8% at T3 (Supplementary Datafile 9). However, this marker-based approach is unable to further distinguish zoonotic from non-zoonotic serotypes. Compared to T1, T2 samples also contained significantly higher relative abundance of replication and recombination machinery of the host-adapted *S. epidermidis* bacteriophage (e.g., helicase loader and replication helicases and Holliday junction resolvases), as well as *IS6* sequences associated with methicillin resistance (*IS431mec*), erythromycin resistance (*IS257-1*), and transposable components of *IS6*/*IS26* and *TnAS3* involved in mobilizable resistance [44] at human–animal interface contexts.

Following showering, the most abundant ICE module *ICETn6087* was reduced in relative abundance compared to T2; however, the next 9 most-prevalent MGEs increased in relative abundance at T3 versus T2 (Fig. 4B), notably *IS431mec*, which already exhibited a significant increase from T1 to T2. Other significantly more abundant MGEs in T3 versus T2 samples included plasmids of *S. epidermidis* (*pSepCH, SE_p410*), *S. aureus* (repV: *pT181*; pgi: *pSJH901*), and *S. epidermidis* and *Bacillus cereus* bacteriophages and prophages. *S. suis–*adapted *ICEPaeLESB58-1* and *ICETn4371* were significantly more abundant at T3 versus T1, while promiscuous tetracycline-associated Tn916-like ICE *ICETn6085a, ICETn6085b*, and *ICETn6084* were significantly less abundant in T3 compared to T1 [45] (Supplementary Datafile 11).

Worker skin at T2 (i.e., following work with swine) had a higher MGE abundance than contact-matched swine (Fig. 4B), dominated by mucous membrane, respiratory tract, and enterically adapted ICEs. Prominent among these were Streptococcal RD2 element (10750-RD.2), *ICETn1806*, and *ICESauJKD6008* and *ICECTn4*, known to mobilize vancomycin and tetracycline resistance in *S. aureus*, Enterococci, and *Clostridioides difficile* [46–49]. *S. aureus* and *Escherichia coli* plasmid replicon modules were also in higher abundance in T2 worker versus swine skin (e.g., *repUS12_pUB110, repUS23._repA* (*SAP099B*)*_GQ900449.1*, and *IncY_1__K02380*). Additionally, worker skin contained a higher relative abundance of *Psychrobacter*-associated plasmid *pRWF101_PsycPRwf. Psychrobacter* species were identified as the most abundant member of the swine skin microbiota in strain analysis (Fig. 2E), especially *Psychrobacter pasteurii* and *Psychrobacter piechaudii*, whereas strains of *Psychrobacter faecalis* and *Psychrobacter maritums* were detected in nearly all human, swine, and environmental samples. The detection of *Psychrobacter*-associated MGE alleles in human samples was, however, unexpected. Historically, *Psychrobacter* isolates have been obtained from arctic, marine, sediment, and limited terrestrial environments [50]. However, recently, this genus has been detected in pig slurries, manure, and swine carcass processing facilities [51, 52], and *Psychrobacter* spp. have been identified as dominant microbes within the nares of workers involved in swine transport [53].

### Metagenome-assembled genomes recovered from worker and swine skin samples represent putatively novel strains

**Figure 5:**
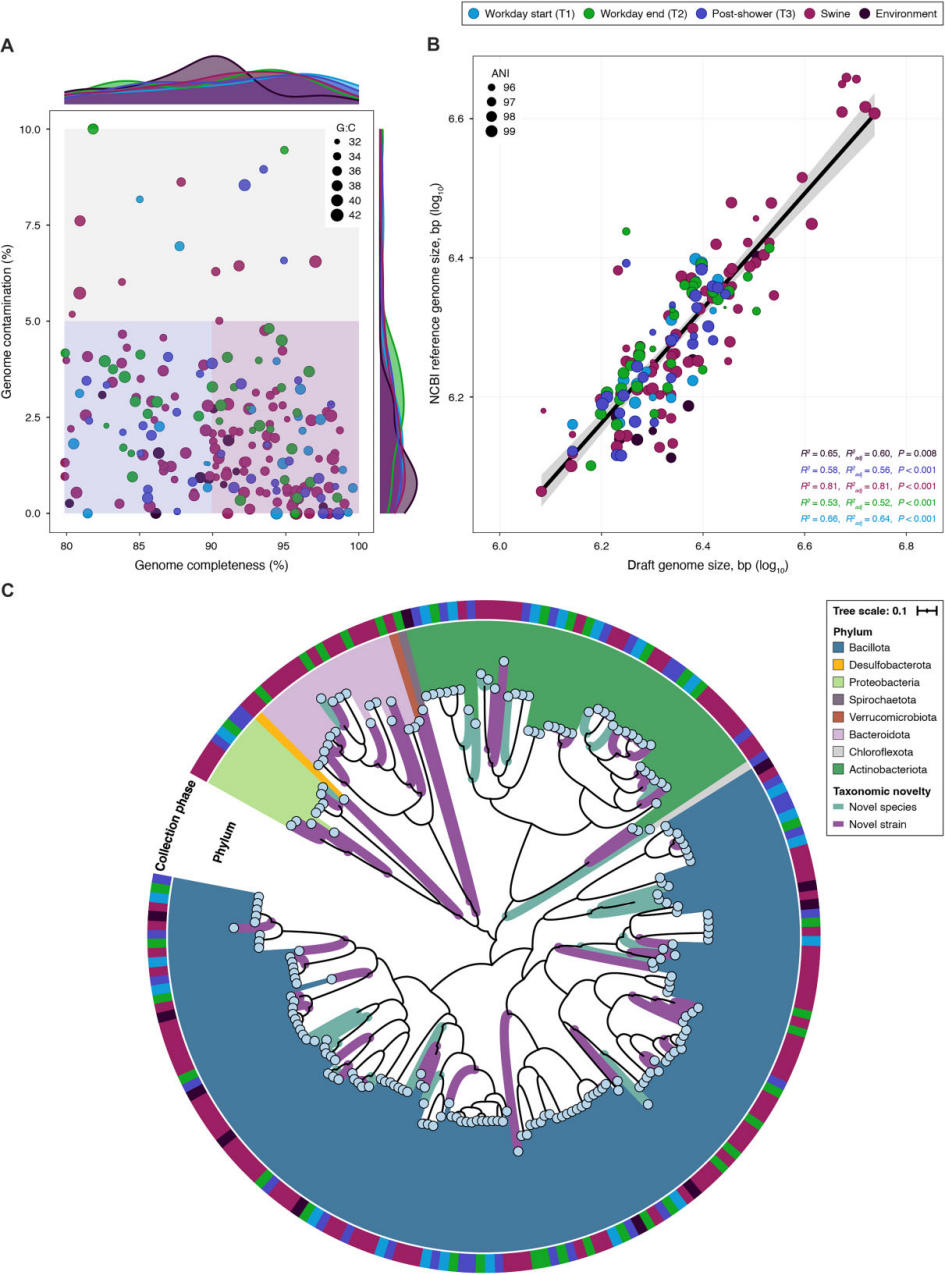
Taxonomic diversity and novelty of resolved MAGs recovered from the human–swine interface and ambient environment. (A) Scatterplot of points representing individual GTDB taxonomically binned MAGs according to their estimated percent completeness (x-axis) relative to their estimated percent contamination (y-axis). Point size is proportional to the quantified MAG GC-content. Pink, purple, and gray regions of the plot demarcate the density of “high quality,” “medium quality–low contamination,” and “medium quality” MAGs retrieved across all collection phases. (B) Scatterplot of estimated MAG size is displayed based on the log_10_-normalized nucleotide count (x-axis) and the nearest assigned NCBI reference genome size expressed in log_10_-normalized nucleotide count (y-axis). A global regression line and 95% confidence interval (shaded region) are displayed, and results of significance testing (*R*^2^; *P* < 0.05) using a generalized linear model are colored for MAGs recovered for each collection phase. (C) Phylogenomic tree of de-replicated and high-confidence MAGs recovered from target-enriched metagenomes across each collection phase (outer ring). The area below each leaf is colored according to the taxonomically assigned phylum. Branches are displayed in teal for proposed novel species (ANI <95%) and in purple for proposed novel strains (ANI <99%).

High-quality metagenome-assembled genomes (MAGs) were constructed via *de novo* genome assembly for all individual samples and also as coassemblies of samples within T1, T2, T3, swine, and environmental samples (Fig. 5A–C, Supplementary Datafile 12). The *Bacillota* phylum was by far predominant across all genomes (*n* = 139), followed by *Actinobacteriota* (*n* = 44), *Bacteroidota* (*n* = 17), and *Pseudomonadota* (syn. *Proteobacteria*) (*n* = 9). The distribution of the most abundant phyla across all MAGs was consistent with the phyla detected by 16S microbiome sequencing. A large proportion of identified MAGs had poor taxonomic representation among known Genome Taxonomy Database (GTDB) MAGs, as 47 (22%) were classified as putatively novel species (i.e., <95% average nucleotide identity (ANI) with a known sequenced genome in GTDB), and 167 (78%) were identified as putatively novel strains (i.e., <99% ANI with a known sequenced genome in GTDB).

Approximately 60% (28/47) of all MAGs considered putatively novel species were recovered from swine skin samples, even though swine samples represented <25% of analyzed samples (i.e., 10/42). Swine samples also accounted for ~53% (89/167) of the MAGs identified as putatively novel strains. We detected new strains that were highly abundant in recent swine intestinal MAG catalogs [54] and that we also identified either via 16S or strain gene-marker analysis in this study, including *Psychrobacter* (*P. pasteurii*), *Streptococcus* (*S. hyovaginalis, S. pluranimalium, S. dysgalactiae*), *Corynebacterium* (*C. xerosis, C. variabile, C. glutamicum, C. pollutisoli, C. stationis*), and *Lachnospiraceae*.

Worker skin samples accounted for ~45% of the de-replicated MAGs, retrieved predominantly from coassembly (*n* = 62) versus individual (*n* = 33) approaches. Nearly 75% (71/95) of MAGs recovered from human samples represented either novel species or strains. These novel taxa comprised 9 genera, 6 of which are known to be natural inhabitants of environmental matrices, including *Microbacterium, Marihabitans, Marmoricola, Chloroflexi* bacterium, *Qipengyuania*, and *Tsuneonella*. Samples representing the swine farm exposure phases (i.e., T2 and T3) accounted for 76% of the total putatively novel strains detected in worker microbiomes (T2: 28/95; T3: 26/95). Though samples from workday start (T1) accounted for the smallest proportion of all recovered human MAGs (23%), we nevertheless captured major expected cutaneous taxa as documented in previous strain-resolved MAG workflows [55, 56], including *Staphylococci* (e.g., *S. hominis, S. epidermidis, S. capitis*), *Corynebacterium* (e.g., *C. xerosis, C. mucifaciens, C. kefirresidentii*), *Cutibacterium* (e.g., *C. granulosum* and *C. acnes*), and *Lactobacillaceae* (e.g., *Lactobacillus amylovorus, Latilactobacillus sakei, Limosilactobacillus reuteri*).

In addition to the genera observed at T1, MAGs from T2 samples also included 6 genera of the Clostridial coabundance gene group 138 (i.e., CAG-138) previously linked with critical functions for fiber degradation in the swine enteric system [57]. Genera not assigned with NCBI taxonomic nomenclature from Lachnospiraceae, Butyricicoccaceae, Oscillospiraceae, and Treponemataceae were also recovered, and their identities were concordant with best-matched NCBI genomes sequenced from fecal samples of piglets <30 days old. Among MAGs recovered from T3, ~55% (20/36) included species identical to those observed in both T1 and T2 samples. However, T3 MAGs also included species that were only observed at T2 (and not at T1), including taxa typically identified in livestock such as *Streptococcus alactolyticus*, known as part of the *Streptococcus bovis*/*Streptococcus equinus* complex (SBSEC); *Aerococcus urinaeequi*; and uncharacterized MAGs previously identified in swine fecal samples (GenBank ID: GCA_016293975.1, GCA_004558825.1, GCA_004556755.1) [58]. Though minor human skin commensals were exclusively detected in T3 samples, such as *Lawsonella clevelandensis* and *Corynebacterium aurimucosum*, genera previously isolated from environmental matrices were also exclusively recovered in T3 samples, including *Tsuneonella* sp., *Qipengyuania* sp., *Marmoricola* sp., and *Chloroflexi* bacterium UBA6265.

## Discussion

Environmental exposure histories play a determinative role in shaping adult microbiomes, even more so than individual-level variables [59–61]. Cutaneous microbiota are recognized for their remarkable stability in the face of environmental perturbation over short time scales [62–64]. Despite this, we demonstrate that swine worker skin experiences a significant increase in bacterial load and a significant shift in microbiota composition during a single 8-hour workday. However, showering at the end of work seems to dampen these changes, indicating that biosecurity interventions reduce not only worker-borne swine pathogen transmission [65, 66] but also work-associated microbiome impacts. We likewise demonstrate that accumulation of ARGs on the skin can be counteracted with showering, suggesting that such biosecurity practices could be important public health measures to reduce the bidirectional flow of resistant bacteria between animal-associated workplaces and the general community [67–69]. Our work highlights that showering may have a variable impact on other aspects of the microbial metagenome, such as mobile genetic elements, which were in some cases enriched even after showering at the end of the workday. Effects of farm protocols and biosecurity have not been expressly evaluated in recent investigations of “shareable” microbial features between humans, animals, and the farm environment [5, 53, 70–73]. Our results suggest that a more nuanced understanding of these practices on different components of the metagenome is warranted, particularly given the different intrashift dynamics we observed across the resistome, mobilome, and microbiome. Future studies should include detailed characterization or even measurement of process controls, including the use of biosecurity practices for decontamination and exposure reduction via personal protective equipment (PPE), occupational training and monitoring, and environmental management.

Within-farm sources of microbes that shape worker microbiomes remain unknown, and there are no systematic, established methods for conducting microbiome-based surveillance in occupational health contexts, particularly for commercial farmwork. The striking proportion of possibly novel species that we recovered from the skin of both swine and workers suggests that skin may be an important yet underrepresented sampling target for on-farm occupational health research (Fig. 5). We focused our study on the skin for several reasons, including ease of sampling and a high proportion of skin-associated diseases within livestock workers; additionally, the skin surface is continuously exposed to the farm environment and thus likely to serve as a competent catchment for air-borne bacteria. Bacteria from livestock feces, soil, and water are relevant sources of exposure that may induce shifts in human microbiomes. For example, swine farms and especially swine feces can impact the antibiotic resistome of individuals living in proximity to the farm [74]. However, emerging evidence suggests that air and dust should not be overlooked as important sources of microbiome and resistome richness [75–77]. Farm dust has also been shown to be protective against asthma [77], and together, these studies underpin the need to further investigate the skin–environment interface. Though our study was not expressly designed for robust source attribution of the microbiome, ARGs, and MGEs in worker skin, future work could conduct more precise analyses of the bacteria and their genetic features. Such detail could inform methods to control or reduce bidirectional exchange of bacteria along the human–animal–environment continuum. To achieve this, more thorough sampling of diverse environmental matrices would be needed, and microbiomes of workers should be studied with more precise measures of exposures to air, soil, feed, dust, feces, and animals when performing a range of tasks over the course of the workday.

One major question is whether work-acquired microbes become incorporated into the cutaneous microbiome as long-term, stable members of the community. Our study design did not include long-term follow-up, and thus we could not quantify the proportion of taxa that become *de facto* colonizers following repeated workday exposures. However, we did demonstrate that showering seemed to literally wash away many of the microbes, ARGs, and MGEs that accumulated on the skin during the workday. This may be due to the fact that bacteria acquired during the workday become only weakly adherent to the skin and thus are easily washed away. The impact of showering is even more robust when one considers the heterogeneity in showering practices, as workers in this study were told to shower as they normally would at the end of their workday, including use of their own preferred soaps and other personal care products. However, it is important to note that showering did not completely eliminate newly acquired taxa, and workers may continue to harbor microbes from enteric and environmental taxa that are characteristic of the swine farm context, as shown in our 16S rRNA and MAG results (Figs. 2 and 5), and reported in a previous study of swine farmworkers who resided in a Chinese swine farm for ~3 months [5]. In this study, we focused on showering as a key biosecurity intervention to reduce the theoretical carryover of farm microbiota, ARGs, and MGEs. However, swine workers followed multiple biosecurity practices throughout their workday, which may have influenced the microbiome composition at T2 and ultimately at T3. The effects of additional protective measures—such as wearing coveralls, boots, gloves, handwashing, and sanitizer use—could not be isolated from the impacts of swine farm exposure at T2, as these practices were either mandatory or encouraged for all workers as part of occupational safety programs at the commercial swine farm. However, the detection of significant microbiota shifts at T2, despite the variability in how swine workers traditionally adhere to PPE protocols and hygiene [78, 79], suggests that farm factors beyond PPE and other biosafety measures contribute to dynamic changes in worker skin microbiota at T2 and T3.

Our findings underscore several critical considerations for future research on workplace and environmental influences on human microbiota. Most importantly, this area of occupational research requires refined methodologies for sampling, microbiome quantification, and molecular epidemiologic analysis. These methodologies must be developed for the diverse environmental conditions, varying microbial burdens, and the complex interactions at human–animal–environment interfaces. In this study, we employed a multisite composite sampling strategy to examine the skin microbiome and metagenome across different phases of collection. This approach was carefully designed to address key challenges in occupational skin microbiome research: (i) we sampled multiple skin sites representing distinct microenvironments (e.g., dry, moist, oily) that support different bacterial taxa. (ii) We selected sampling sites based on a range of exposure likelihoods to air, dust, feces, and other components of the swine environment, considering sampling areas that were both protected and unprotected by PPE. (iii) By pooling samples into a composite, we streamlined the collection process, making it less burdensome and more easily integrated into the daily routines of agricultural workers. Second, occupational microbiome research is challenged to establish causal links—that is, do workplace exposures influence the human microbiome in the long-term, and do these influences lead to different health outcomes? This study is a prime example of this challenge, as we enrolled farmworkers who already had months or years of on-farm exposures. Thus, the “baseline” T1 skin microbiome may have already been impacted by previous on-farm exposures, but we have no robust method for detecting these prior impacts. Similarly, it is tempting to compare our data to publicly available human skin microbiome data, but such a comparison would be inextricably biased by confounders such as the well-documented “healthy worker” effect and other demographic variables known to impact human microbiomes. The need for robust epidemiological study design is even more pronounced in cohort-based microbiome studies because of the lability of the human microbiome. We therefore expressly avoided comparisons across worker and nonworker cohorts and took care not to extrapolate our findings into long-term microbiome or health impacts. Instead, we focused our analysis on a time-series sampling design of the same workers before and after exposure and showering, allowing for a targeted analysis of daily farmwork and biosecurity interventions. This intraindividual focus allowed us to circumvent many of the biases that beset ecological analyses, but at the expense of external validity and ability to draw conclusions about the long-term impact of farmwork as compared to non-farmwork.

Finally, we used a combination of sequencing and bioinformatic approaches that were tailored to maximize sensitivity for resistome and mobilome analysis while also supporting a broader investigation of the microbiome. Specifically, we used target-enriched shotgun sequencing, an adaptation of traditional shotgun metagenomics, to enhance detection of ARGs and MGEs, which are typically undetected by metagenomic workflows [35, 80, 81]. Such false-negative findings are most pronounced in low biomass samples, such as skin samples, which are replete with nonmicrobial genomic DNA (gDNA) [64, 82]. Thus, our choice of target-enriched metagenomics was driven by its capacity to reduce false negatives in resistome and mobilome profiling. The enrichment procedure hinges on biotinylated hybridization probes, which tolerate >40% sequence mismatch between the sequence target and the probe (i.e., up to 40 base mismatches across a 120-*mer* oligo probe). This inherent hybridizing flexibility facilitates the capture of both predefined targets and other potential closely related variants. Despite this enrichment, resistome and mobilome genes constituted <16% of the host-filtered metagenomic reads in this study, meaning that >84% of sequencing data across samples did not originate from ARGs or MGEs (i.e., was “off-target”). We used this remaining off-target data to reveal strain-level dynamics from the recovery of specific strain markers and the analysis of high-quality genomes recovered from human, animal, and environmental metagenomes. However, because the process of target enrichment purposefully induces a nonuniform impact on metagenomic content, its effects on compositional analyses remain poorly characterized at this time. Given these potential biases, we complemented our analysis with 16S rRNA amplicon sequencing to provide a less biased assessment of microbial community structure across the skin of swine workers, swine, and environmental samples. This dual-sequencing strategy ensured both high-resolution resistome–mobilome characterization and robust taxonomic profiling of the skin microbiome.

It is difficult to extrapolate our single-day, single-farm study to longer-term dynamics of the worker skin microbiome, and our results suggest that further work is needed to determine whether daily environmental exposures impact the long-term profile of the worker skin microbiome, as well as whether such impacts carry over into the general community via human-to-human transfer. We note, however, that this study provides new insights into the stability of the microbiome, resistome, and mobilome of farmworkers as they enter and exit a commercial US swine production system. Given the challenges of conducting research in controlled swine environments, such microbiome exposure assessments on US farms are scarce. These results therefore offer an important foundation from which more robust microbiome investigations can emerge.

## Materials and Methods

### Study design and procedures

Ten workers from a single commercial farrow-to-wean operation in the Midwestern region of the United States were voluntarily enrolled into the study after providing written informed consent. At time of sampling, the farm housed approximately 3,500 sows and >15,000 piglets, and weaning occurred at an average age of 22.5 days of age. This large-scale swine farm had been in operation for >20 years and was chosen because all facility personnel were day-shift workers and tended to have specialized job tasks requiring a wide array of swine contact, ranging from no direct contact (e.g., facilities maintenance, sanitation, manure management) to intensive direct contact (e.g., assisting with farrowing, piglet processing, or providing veterinary care). The human sample collection events for this study were integrated into a preexisting, long-standing biosecurity pathogen surveillance program that already involved self-collection of skin swabs. Incorporating microbiome sampling within the existing workplace surveillance program increased study participation and self-sampling consistency and was determined to be least disruptive to work-related schedules and task performance.

All workers were enrolled and sampled on the same day. Worker enrollment eligibility criteria included a restricted age range (18–60 years); proficiency of spoken and written English; minimum of 6 months continuous employment at the facility; no other contact with swine outside of the workplace; no exposure to antibiotics, immunosuppressants, or antiviral drugs in the prior 3 months; no hospitalization or incarceration for >24 hours in the 3 months prior to enrollment; free of any *known* symptoms, infections, or diseases of the upper respiratory tract (including ears, nose, and throat) and skin at the time of enrollment; no *known* current or past diagnosis of autoimmune disease; no cancer diagnosis or related therapy in the previous 5 years; and an afebrile status at the time of enrollment. Participants were asked to abstain from any additional showering or application of personal care products to their skin other than those required at the swine facility for a period of 24 hours prior to sampling. Following the provision of study description information and free informed consent forms for review, formal consent was obtained in confidence and without the presence of farm management personnel from all participants. Participants agreed to self-collect repeated skin swabs at 3 points in their working day and to complete a short questionnaire regarding personal demographic and biometric information, smoking history, dietary and hygiene habits, and occupational- and nonoccupational-related exposure assessments. Variables from the questionnaire were used in downstream multivariable statistical analyses. All study participants, their samples, and questionnaire data were kept deidentified to study personnel and investigators using an assigned alphanumeric coding scheme and an electronic tracking system. Monetary remuneration for study participation was provided in the form of $100 debit cards.

### Sample collection

Epidermal swabs (hereafter referred to as “skin swabs”) were self-collected at 3 workday collection phases: prior to entry into the animal holding areas of the swine operation and before showering-in (T1: “Workday start”); at the conclusion of the 8-hour period of assigned work, but before showering-out (T2: “Workday end”); and upon exiting the facility following a mandatory showering procedure (T3: “Post-shower”). At each time point, collection kits were provided to workers for self-sampling. The kits contained 2 sterile BD BBL CultureSwabs EZ (Becton, Dickinson and Co.) premoistened with a solution containing 0.9% NaCl and 0.1% Tween-20 biosurfactant (Thermo Fisher Scientific). Each swab was used for self-swabbing of all locations on one-half of the body (i.e., left or right), to obtain a single composite representing the following locations on each body half: manus and interdigital space, antecubital fossa, popliteal fossa, and axilla [83]. These locations were selected to represent sebaceous, moist, and dry epidermal microenvironments of the human skin [83]. Swab pattern, pressure, duration, and frequency were performed in accordance with the Human Microbiome Project [84] core microbiota sampling protocol A, including the use of a z-like pattern of consistent swabbings over specified surfaces via 50 passes over a 30-second period. For the manus, “z-like” pattern swabbing was conducted across the palm and surface of the fingers for a 30-second duration as well as an additional 30 seconds of linear passing in the interdigital spaces. For all workday collection phases, the left and right composite swab tips were separated from the swab stems and placed into 1× phosphate-buffered saline (PBS) at pH 7.4, immediately transferred to dry ice for transport, and subsequently placed in −80°C for long-term storage.

Swine skin swab samples were collected as follows: each worker was observed handling or working near specific pens containing sows, piglets, or both in a room during their dayshift. Immediately after the worker completed their contact or their required duties in the specific pen, pen-level composite skin samples were collected by passing a sterile EZ Reach Sponge sampler on the dorsal aspect of the skin of each pig in the pen, from withers to tail base (World Bioproducts), impregnated with 10 mL of 1× PBS. In cases of farrowing pens, the sow and all piglets in a litter were sampled. In gestational pens, only the pregnant sows contacted by workers assigned to a specific area of the facility were sampled. Swine samples were assigned a worker-matched ID, transferred to sterile Whirl-Pak bags (Nasco), and placed on dry ice for transport.

Two additional sterile EZ Reach Sponges were exposed to ambient air for ~20 seconds, one each in gestational and weaning environments of the swine facility, where workers spent the majority of their dayshift; these swabs were meant to capture the airborne environmental microbes within the primary working areas of the facility. After transport (~2 hours driving distance), all samples were immediately placed in −80°C for long-term storage at the Food Centric Corridor infectious diseases laboratory at the University of Minnesota.

### Sample processing and gDNA extraction

All sample processing occurred in a class II biological safety cabinet decontaminated using UV radiation and 70% ethanol in between handling human, animal, and environmental samples. After thawing samples at −20°C and then at room temperature, human swab tips and excess storage fluid were transferred for DNA extraction. An additional 100 μL of 1× PBS was used to recover possible residual microbial material in each sample tube. For swine and environmental samples, the ~10-mL buffer adsorbed to each polyurethane sponge paddle was expressed and reserved in 50-mL conical centrifuge tubes. An additional 10 mL of fresh 1× PBS was allowed to readsorb to each sponge and equilibrate for 10 minutes, and this “rinse” was expressed into the same conical tube as the original rinsate for subsequent centrifugation (8,000 × *g* 15 minutes at 4 °C) and transfer of pellets into ~200 μL PBS for DNA extraction.

gDNA extraction began with mechanical and chemical lysis using the Qiagen DNEasy Powersoil Pro kit (lot 163044275) and PowerBead Pro tubes containing zirconium beads and 800 µL lysis buffer. Molecular-grade sterile water was placed into 3 randomly selected tubes to serve as negative controls (extraction blanks). After 6 seconds of vortexer-mediated homogenization, samples were placed on a 115 V BioSpec Products Mini-Beadbeater-96 for mechanical lysis. Sample bead beating proceeded at 2,400 rpm for 30 seconds for a total of 3 rounds with a 2-minute pause on ice between each round, to prevent overheating. The remainder of the extraction and purification procedure was performed following the PowerSoil Pro protocol with inhibitor removal steps using the QIAcube Connect automated instrument. The final 50 µL of eluted gDNA was stored at −20°C.

Additionally, gDNA was extracted from two 200-µL aliquots of ZymoBIOMICS microbial community II standard (cat. D6310), consisting of 8 prokaryotic and 2 eukaryotic microorganisms in known log-distributed abundance to serve as positive controls. The aliquots were individually centrifuged at 15,000 × *g* for 5 minutes, and the pellets were suspended in 400 μL Qiagen CD1 lysis buffer, transferred to PowerBead Pro tubes, and vortexed for 10 minutes at maximum speed on a Vortex Genie 2 mixer (Scientific Industries) with a Qiagent adaptor (cat. 13000-V1-24). After isolating and reserving the initial supernatant, an additional 400 μL CD1 lysis buffer was added to the same PowerBead Pro tubes, and these were subjected to bead beating (Mini-Beadbeater-96) at 2,400 rpm for four 5-minute cycles with 5-minute rest intervals on ice in between each cycle. After centrifugation at 15,000 × *g* for 2 minutes, the resulting supernatant was recombined with the previously reserved supernatants for each aliquot. The gDNA from the two ~700-μL lysates were isolated and purified on the QIAcube Connect according to the same protocol as for the previously described samples.

### 16S rRNA amplicon library preparation and sequencing

The 16S rRNA gene copy number in each sample was measured using quantitative PCR (qPCR) prior to library preparation. For 16S sequencing, the target copy number threshold was set at 167,000 molecules/µL. Amplification of libraries was performed using a dual-indexing Illumina primer set (forward primer: 5′-TCGTCGGCAGCGTCAGATGTGTATAAGAGACAGCCTACGGGAGGCAGCAG-3′ and reverse primer: 5′-GTCTCGTGGGCTCGGAGATGTGTATAAGAGACAGGGACTACHVGGGTWTCTAAT-3′) targeting the V3−V4 region [85]. Amplicons were quantified using a PicoGreen dsDNA assay kit (Life Technologies). Sequencing was performed at the University of Minnesota Genomics Center (UMGC) using Illumina’s v3 chemistry (2 × 300-bp paired-end reads) on the Illumina MiSeq platform. All libraries were sequenced on the same sequencing run.

### Target-enriched metagenomic library preparation and sequencing

All gDNA samples were subjected to a targeted capture and deep sequencing workflow to increase detection sensitivity for the resistome and mobilome features within the metagenomic DNA. This approach was chosen to enhance sequencing and thus detection of ARG and MGE targets, which are rare genomic features within microbiomes [35]. Targeted enrichment was performed using a custom-designed biotinylated cDNA probe panel for selective hybridization and capture. For probe design, a comprehensive list of predefined publicly available unique nucleotide sequences was compiled for 7,868 ARGs from MEGARes v2.0 [86] (including accessions for drug resistance, metal resistance, multicompound resistance, and biocide resistance) and for 738 MGE accessions (including full-length sequences for (a) ICEs from ICEBerg v2.0 (RRID:SCR_006026) [87] and (ii) plasmid replicons of *Enterobacteriaceae* and gram-positive bacteria from PlasmidFinder v2.1 [88]. These ARG and MGE sequences comprised 8.55 Mb of total sequence. The CATCH pipeline [89] was used to generate the custom probe panel using the following parameters: probe stride, 120; probe length, 120; mismatches, 5; and extension coverage, 100. The final probe design contained 71,309 unique 120mer oligos, which provided 100% horizontal coverage of all ARG and MGE targets, with at least 2× depth of probe coverage for every nucleotide. Probes were manufactured by Agilent with additional “bait-boosting” to amplify GC-rich regions (defined as GC >65%) for fast hybridization reactions to produce a final panel of 148,162 probes covering 17.78 Mb. Probes were stored at −80°C prior to use.

All gDNA samples (*n* = 42) were initially subjected to additional Agencourt AMPure XP (Beckman Coulter) bead-based purification to retain fragments >100 bp using a 40:50 vol/vol ratio. After size selection, targeted enrichment and library preparation using the Agilent SureSelect XTHS V2 system and our custom bait design were performed using a minimum input of 100 ng purified DNA. Initial enzymatic fragmentation employing the Agilent XT Low Input Enzymatic Fragmentation Kit was used to generate short (150–250 bp) fragments, which were subsequently end-repaired, dA-tailed, and adapter-ligated for Illumina paired-end sequencing with multiplexing, following manufacturer recommendations. After probe-based hybridization following the 90-min PCR protocol, capture using MyOne streptavidin T1 beads (Thermo Fisher Scientific) was increased to 2 hours with minimized vortexing (1,200 rpm). Additional quality control steps were followed, including use of a Qubit 4.0 fluorometer (Thermo Fisher Scientific) and TapeStation 4200 for gDNA analysis (Agilent), as well as high-sensitivity TapeStation 4200 analysis for pre- and postcapture libraries.

After pooling libraries, a KAPA qPCR Library Quantification kit (Roche) was used to confirm functionality of the barcoded pool, and a MiSeq Nano run (V2 chemistry, 2 × 150-bp paired-end reads) was used to assess final barcode balance. The final multiplexed library was sequenced by the UMGC on a single lane of a NovaSeq6000 (Illumina) with S4 cell chemistry to obtain 2 × 150-bp paired-end reads (675 Gb/lane), with an expected depth of ~54 M paired-end reads per sample.

### 16S rRNA bioinformatic analysis

Amplicon primers were trimmed from the 5′ and 3′ ends of forward and reverse reads using cutadapt with default settings [90]. The trimmed sequences were then input to the DADA2 (RRID:SCR_023519) v1.26 pipeline [91] to generate ASVs. The *filterandtrim* function was used for additional quality trimming, including truncation of forward and reverse reads to 250 bp and 220 bp, as well as filtering of phiX reads and reads with a maximum expected error rate >4. Cleaned sequence reads were used as input to the *learnerrors* function, and the output error-rate matrix was used in read error correction (i.e., denoising) using the *dada* function. Error-corrected reads were aligned and combined into contigs using the *mergepairs* function with the minimum overlap threshold set to 12 bp. An ASV table was generated after removing chimeric contigs using the *removechimera* function. ASVs with a sequence length between 401 and 431 were retained. The *assigntaxonomy* function was used for taxonomic assignment of ASVs using the SILVA v138.2 reference database [92] via the native Bayesian classifier, and species annotation was performed via the *addspecies* function. Potential contaminating ASVs were identified using 16S qPCR copy number results per μL using *isContaminant* function in the Decontam v3.6 R package, as implemented in the frequency method [93], and were removed from downstream analysis. The final ASV count matrix with taxonomy file was generated from the DADA2 pipeline and saved in RDS file format for subsequent downstream analysis.

### Targeted shotgun metagenomics bioinformatic analysis

An alignment-based approach was used to detect ARG and MGE target sequences in all enriched metagenomic data, as implemented in the AMRPlusPlus v2.0 pipeline [86]. Briefly, read trimming and quality filtering were performed using TRIMMOMATIC (RRID:SCR_011848) v0.33 [94]. Host reads were identified in worker swab and swine/environmental samples by aligning sequence reads to the *Homo sapiens* (hg19) and *Sus scrofa* reference genomes, respectively, using BWA v0.7.17 [95]. Nonhost reads were then extracted using SAMtools (RRID:SCR_002105) v1.9 [96] and aligned to the MEGARes v2.0 reference database using BWA-MEM. To mitigate the impact of mobilome misclassification due to sequence homology between MGEs and ARGs [97], we developed a custom script (extract_paired_unmapped.py) to extract paired reads not aligned to any resistome accessions. The ARG-filtered reads were separately aligned to sequences of a concatenated MGE database for ICEs, plasmid replicons, plasmid modules, prophage, and virus (bacteriophage) extracted from ICEberg v2.0, PlasmidFinder v2.0.2, and ACLAME v0.4 [98]. For PlasmidFinder, only replicon accessions for gram-positive bacteria and *Enterobacteriaceae* were included. Hits to accessions of plasmid modules, prophage, and virus/bacteriophage within the ACLAME database were further parsed for ISs and other TEs utilizing a custom scripting process that automates interfacing with ISfinder [99, 100] and ISbrowser [101]. Briefly, this script takes as input reads that are aligned to any aforementioned ACLAME accessions and uses Entrez queries of the associated NCBI GenBank accessions. Each resulting query outputs metadata details for the accession (e.g., accession_id, start, stop, gene_description, locus name) and the associated full sequences. The Selenium webdriver for Python v4.8.3 is used to automate submission of the GenBank-queried ACLAME sequences to ISbrowser. The native BLASTN algorithm is used to parse hits for plasmid, prophage, and virulence accessions, to detect and differentiate IS families and related TEs. Query hits for genes were retained if they met the following criteria: minimum identity of 80%, >80% coverage of the query length, and an E-value ≤1 × 10^–10^. When multiple hits with similar threshold values were obtained, the assignment with the highest bitscore was chosen. BLASTN results were parsed and each sequence query was called as “likely IS,” “likely TE,” or “unclassified” and flagged for additional manual analysis if an accession was discontinued from further curation in NCBI GenBank. Additionally, all ACLAME accessions not identified as IS or TE were manually checked against current GenBank annotations and grouped according to putative functional attributes, as confirmed with Universal Protein KnowledgeBase (UniProtKB) annotation [102], including biosynthesis regulation, non-conjugative efflux and transport, relaxase and mobilization machinery, replication initiation and maintenance, plasmid replicon module, DNA secretion and conjugative machinery, stress-SOS-tox/anti-tox-partitioning, transcription and translation regulation, transposition and recombination, and virulence and pathogenicity domains. Accessions with unconfirmed protein function and unassigned annotation in GenBank were considered “unclassified,” and accessions with both an unconfirmed protein function and an unsupported GenBank accession were categorized as “hypothetical.”

To reduce false-positive detection of ARGs and MGEs, only ARG and MGE accessions attaining a conservative gene fraction cutoff of 80% within a given sample were considered positively identified, using the default settings in AMRPlusPlus v2.0. Gene fraction was defined as the proportion of nucleotides within a given accession aligned by at least 1 sequence read. The default deduplication procedures of the AMRPlusPlus v2.0 pipeline were used to account for potential amplification bias introduced during the molecular target enrichment process described above [86]. Identified ARGs and associated alignment counts were aggregated at the “group,” “mechanism,” and “class” levels using the standard MEGARes ontology. Additionally, 29 ARG groups were flagged as “clinically important” (i.e., priority ARGs) due to their prevalence in clinical disease and frequent co-occurrence with MGEs [36, 37, 103]: *bla_CTX-M_, bla_GES_, bla_IMI_, bla_KPC_,bla_SHV_, bla_TEM_, bla_IMP_, bla_NDM_, bla_CMY_, bla_OXA_, mecA, mcr-1, mcr-2, vat, vga, vgb, bla_SME_, cfr, aac(6’)-I, bla_Z_, bla_VIM_, ermB, qnrA, qnrB, tetM, dfrA, vanY-b, vanY-d, vanY-a*, and *sulI*. Prior to analysis, all hits to ARG accessions requiring SNP confirmation (signified by the “*RequiresSNPConfirmation*” in the MEGARes header) were removed, as additional confirmatory assessments would be needed to ensure accurate detection of these genes. Since no mobilome-wide ontology currently exists, counts for MGEs were aggregated to the HGT mechanism type (i.e., ICE, plasmid, prophage, virulence, and IS/TE).

### Assessment of sequencing depth, quality, and host genome abundance

Microbiome sequencing variables, including the number of raw reads generated from 16S rRNA sequencing, 16S copy number, and 16S mean quality score, were assessed with respect to the following independent variables: collection phase (i.e., Workday start, Workday end, Post-shower, Swine, and Environment), worker exposure type (i.e., direct vs. indirect), and sample type (i.e., human skin swab, swine skin swab, standard mock microbial community, and negative control). Enriched metagenomic sequencing variables, including total generated raw reads (i.e., sequencing depth), total number of host versus nonhost reads, and mean quality score, were similarly assessed with respect to collection phase, worker exposure type, and sample type. Associations between these independent variables on 16S and shotgun metagenomic sequencing metrics were independently quantified via linear mixed models using the *lme4* v1.1–29 package [104] with subject ID as random effect to account for repeated measures. Statistical significance of each independent variable was evaluated using ANOVA with a predefined α of 0.05. All independent variables in the final models were subjected to Tukey-adjusted pairwise comparisons using the Emmeans v2.30–0 package [105], again with a predefined α of 0.05. Statistically significant variables were included as covariates in all subsequent models to account for potential confounding related to sequencing effort and/or quality. Assumptions of normality for dependent variables were assessed using visual inspection of residuals, and log_10_ variable transformations were applied to meet model assumptions.

### Quantification of microbiome, resistome, and mobilome diversity and abundance

Richness and Shannon’s diversity were estimated for each collection phase and for swine samples using the *estimate_richness* function in the Phyloseq v3.20 package under the Bioconductor release repository. Diversity metrics were calculated at the genus level for 16S data, at the group level for ARGs, and at the mechanism level for MGEs. Differences in diversity were evaluated using linear mixed models via the *lme4* function, specifying 16S copy number (and host-removed sequencing depth for enriched shotgun data) as covariates and worker ID as a random effect. Model building and extraction of model estimates were performed as reported above for the assessment of sequencing depth and quality. Rarefaction analysis and relative abundance analysis of genera, ARG groups, and MGE accessions were performed using the MicrobiotaProcess R package v1.18 [106]. Due to the limited sample size, the “Environment” samples were excluded from all diversity analyses.

Prior to assessing β-diversity, genera not present in the 16S data or ARGs/MGEs absent from shotgun data across all human and swine samples were handled using a compositional approach [107] to imputation of zero-inflated count matrices performed using the zCompositions package v1.5.0–4 [108] calling the geometric Bayesian-multiplicative replacement of zero counts function *cmultRepl*, which outputs pseudo-counts. To account for differences in sequencing depth and to attenuate the influence of highly abundant accessions, robust center log ratio (rclr) normalization was applied to all microbiome, resistome, and mobilome counts, which were then transformed to Euclidean distances using the *vegdist* and *decostand* functions in the Vegan package v2.6–8 [109]. The *ordinate* function in Phyloseq was applied to the resulting Aitchison compositions using principal component analysis (PCA). As with the α-diversity metrics, β-diversity was assessed at the genus level for 16S data, at the group level for ARGs, and at the mechanism level for MGEs, with a predefined α of 0.05 for all statistical tests. Differences by collection phase were first assessed via the omnibus ANOSIM. PERMANOVA was performed using the *adonis* function in Vegan based on 10,000 permutations. *Post hoc* pairwise comparisons were performed using the *pairwise.adonis* function as well as *adonis2* with stratification on worker ID for skin samples when comparing across collection phases T1–T3. As both ANOSIM and PERMANOVA tests are susceptible to dispersion heterogeneity, which may confound between-group with within-group variance, β-dispersion was assessed via the *betadisper* function in Vegan.

Resistome and mobilome feature counts were utilized to analyze the total ARG and MGE sample load. This was done using a modified approach of Li et al. [110], where the gene feature counts are expressed on the basis of the sample qPCR 16S gene copy number as well as the reference feature sequence length. This “abundance” of the resistome or mobilome was then log_10_-normalized and summarized by collection phase. Statistical differences in resistome or mobilome abundance were assessed using the linear mixed-model approach and pairwise comparisons as described for sequencing and diversity assessments.

### Microbiome, resistome, and mobilome differential abundance analysis

Raw count matrices from the microbiome, resistome, and mobilome were utilized as input for differential abundance testing. In addition to filtering for sparse features as described in the β-diversity analysis, ARGs and MGEs were subjected to additional pruning; specifically, ARGs and MGEs with <80% prevalence in any of the 3 collection phases (T1, T2, or T3) or the swine samples were removed from differential abundance analysis. Additionally, for each pairwise set of collection phases being compared during differential abundance analysis, features were censored if they had <10% prevalence and <10 counts. Prior to each differential abundance test, ARGs were agglomerated to both the group and mechanism levels, while MGE accessions were not subjected to agglomeration as no unified MGE taxonomy exists.

All count matrices and associated metadata stored as phyloseq objects were passed to the DESeq2 v1.46.0 R package [111]. The *poscounts* option was used for estimation of size factors, which utilizes a modified relative log expression to account for missing alleles across samples. Estimates of dispersions were based on the negative binomial likelihood for each allele as implemented natively in DESeq2. Shrinkage of dispersion estimates and subsequent log_2_-fold change (log_2_FC) effect sizes were performed via the empirical Bayes method of adaptive shrinkage implemented in the ashr R package to minimize the FDR [112]. Log_2_FC estimation was performed using results of a negative binomial generalized linear model specifying collection phase as the main predictor and library cDNA concentration, host-removed sequencing depth, gender, body mass index, smoking status, and consumption of pork as model covariates. Hypothesis testing was performed with the default Wald test and FDR adjustment. Differential abundance testing was performed for the following comparisons: workday start (T1) versus workday end (T2), workday end (T2) versus post-shower (T3), workday start (T1) versus post-shower (T3), and the “interfacing period” represented by swine versus T2 samples. To guard against spurious findings, predefined cutoffs for statistical significance and effect size were used. At the genus level, we used an adjusted α of 0.05 and an effect size (log_2_FC) of ±1.5. For ARG group and MGE mechanism levels, we used an adjusted α of 0.01 and an effect size (log_2_FC) of ±1.5; for ARGs at the mechanism level, the adjusted α was increased to 0.05.

### Inferring microbial community connectivity and ecological dominance from sparse datasets

The SParse InversE Covariance Estimation for Ecological Association Inference (SPIEC-EASI) [113] approach was deployed as implemented in R v1.1.1 to infer and analyze ecological networks and keystone members of the microbiome. Separate networks were built for each collection phase and for the swine samples. To build each network, we fit a negative binomial distribution to *clr*-normalized ASV count data. For input, ASV counts found in >1 sample with >100 raw counts were included in all network analyses, based on recommended filtering procedures [113]. Since only 2 samples were collected for the “Environment” collection phase, these samples were not assessed. Model inferences were performed using the Meinshausen and Bühlmann neighborhood selection framework [114], and model sparseness was inferred using the Stability Approach to Regularization Selection (StARS), as implemented in the Pulsar R package v0.3.10 [115] with parameters *lambda.min.rati o*= 0.01 and *nlambda* = 20. Stable networks were produced under subsampling with 100 rounds, as well as subsequently analyzed and visualized using igraph v2.0 R package [116]. Community detection procedures based on analysis of optimized “*spin state*” configurations [117] were applied, and modular clustering of subcommunities was evaluated based on Newman and Girvan [118] global modularity (*Q*) estimation. Network assortativity based on modular identity and node degree was evaluated using base igraph procedures. Eigenvector centrality was regressed according to degree centrality for each ASV (node) to estimate putative keystone membership within each network by identifying taxa in the top fifth percentile.

### Identifying strain-level detail in targeted metagenomes

Trimmed and host-removed target-enriched metagenomic reads were mapped to the 1.1 M taxonomic markers contained in the MetaPhlAn4 v4.1 [119] database using bowtie2 and default parameters [120]. Clade-specific marker coverage and normalization across all detected clades using default parameters produced a relative abundance taxonomic profile for each sample. The sample-specific abundance profiles were merged into a single matrix using the MetaPhlAn4 utility script (merge_metaphlan_tables.py). The matrix was then filtered to species prevalence ≥75% of samples. Alignment results on each sample from MetaPhlan3 were used to create files of consensus marker genes for each species. Additional marker sequences were extracted from the MetaPhlAn4 database and in turn were blasted against *Lactobacillus amylovorus* (RefSeq accession: GCA_000194115.1) The sample and reference reconstructed strain marker files were inputted into StrainPhlAn v4.1 (available in MetaPhlAn4), which filters based on the presence of selected clade markers. Multiple sequence alignments for each marker were created by calling PhyloPhlAn (available in MetaPhlAn4) for phylogenetic reconstruction using RAxML and based on default PhyloPhlAn bootstrap values. StrainPhlAn results were used to confirm the presence of specific species of bacteria that had been identified via 16S analysis. StrainPhlAn results were also used to infer sample-level “*sharing*” of strains across collection phases. Briefly, consensus sequences for taxonomic markers were identified by alignment via the MetaPhlan4 pipeline across all metagenomic samples. Major species-level genome bins (SGBs) for all Staphylococci, Streptococci, and *E. coli* identified in initial StrainPhlan results were used to extract SGB marker genes. After extracting SGBs (*S. epidermidis*: SGB7865; *S. haemolyticus*: SGB7860, SGB7861; *S. hominis*: SGB7858; *E. coli*: SGB10068; *S. suis*: SGB8209, SGB29820; *S. alactolyticus*: SGB8017), strain sharing was assessed by profiling the maximum similarity across as many samples as possible using the required inputs and the following parameters: *strainphlan –mutation_rates –marker_in_n_samples 1 –sample_with_n_markers 10 –phylophlan_mode accurate*. Pairwise alignment between targeted SGB markers from each sample pair was aligned and an RAxML phylogenetic distance was inferred for any putative transmission events.

### *De novo* assembly and binning of genomes from targeted metagenomes

Adapter-trimmed and host-removed target-enriched metagenomic reads were used as input for metagenomic assembly with MEGAHIT v1.2.9 [121]. Single-sample assembly for each of the 42 metagenomes was carried out using default parameters. In addition, the 42 metagenomes were coassembled using MEGAHIT on forward and reverse reads with the following options: *–continue –kmin-1pass –min-contig-len 1000*. Contigs >2,000 bp were mapped to single and coassemblies via BWA-MEM [95], and SAMtools was used to sort and convert SAM files to BAM files [96]. The MetaBAT2 v2.15 [122] pipeline was used to assess coverage of assembled contigs and for both single- and coassembly binning. Coassemblies and single-sample assemblies were constructed separately to produce 638 and 712 bins, respectively. All bins were aggregated, and dRep v3.4.0 [123] was used for *post hoc* dereplication with the following flags: *dereplicate -comp 80 -con 10 -sa 0.95*, and CheckM v1.2.2 [124] lineage workflow was used to retain only those MAGs with contamination <10% and completeness >80% using *–checkM_method lineage_wf*. After retaining all primary bins and only the highest-scoring secondary cluster bins, we produced 145 MAGs from coassemblies and 73 MAGs from single-sample assemblies. MAGs were assigned a taxonomy using GTDB-tk v2.1.1 [125], and of the 218 MAGs, 5 were assigned to archaea and not considered in further downstream analysis. Of the remaining 213 draft genomes, >57% (*n* = 123) were categorized as “*high quality*,” >34% (*n* = 74) as “*medium quality*” with minimal contamination, and <8% (*n* = 16) as “*medium-quality*” draft genomes (Fig. 5A). We detected no significant differences (type III ANOVA *P* > 0.1) in genomic parameters for recovered MAGs across T1–T3, swine, and environmental samples (Supplementary Datafile 12), including GC ratio (median range: 35.5–37.4), ANI distribution (median range: 98.2–98.8%), and N50 contig length distribution (median range: 11,949–15,798 bp). MAGs were assessed for their taxonomic novelty by following the procedures of Glendinning et al. [126]. Briefly, MAGs with ANI <95% were considered as putatively novel species and ANI <99% as putatively novel strains. Additionally, MAGs not assigned a provisional genus name were assessed for genus-level novelty via CompareM v0.1.2 at <60% average amino acid identity (AAI). Multiple sequence alignment files generated from the GTDB-tk workflow were concatenated across all collection phases and used as input for phylogenomic clustering, as implemented in IQTREE v2.2.0 [127]. A best-fit substitution model was chosen using the native *ModelFinder Plus* via the Bayesian information criterion. The resulting model (LG+R8) was used to construct the taxonomic tree of MAGs, which was visualized across all study samples using iTOL (RRID:SCR_018174) [128].

## Ethical Approval and Consent to Participate

The University of Minnesota’s Institutional Review Board approved the study (protocol: STUDY 00007351) as no greater than minimal risk to study participants. All procedures, including obtaining informed consent, were followed in accordance with the ethical standards of the Office for Human Research Protections (US Department of Health and Human Services) and with the Declaration of Helsinki (2013). All animals were sampled under authorization from the Institutional Animal Care and Use Committees of the University of Minnesota and participating farms under a collaborative agreement (protocol #5–19).

## Supplementary Material

giaf062_Supplemental_Files

giaf062_Authors_Response_To_Reviewer_Comments_original_submission

giaf062_Authors_Response_To_Reviewer_Comments_revision_1

giaf062_Authors_Response_To_Reviewer_Comments_revision_2

giaf062_GIGA-D-24-00356_original_submission

giaf062_GIGA-D-24-00356_Revision_1

giaf062_GIGA-D-24-00356_Revision_2

giaf062_GIGA-D-24-00356_Revision_3

giaf062_Reviewer_1_Report_Original_Submission Oscar Menc఺-Ares, Ph.D -- 10/29/2024

giaf062_Reviewer_1_Report_Revision_1 Oscar Menc఺a-Ares, Ph.D -- 3/4/2025

giaf062_Reviewer_2_Report_Original_Submission Alex Bossers -- 11/29/2024

giaf062_Reviewer_2_Report_Revision_1 Alex Bossers -- 3/21/2025

## Data Availability

Raw sequence data and sample metadata can be accessed via the Sequence Read Archive (SRA) hosted by the National Center for Biotechnology Information (NCBI) under BioProject PRJNA987158. Sample metadata were recorded using the MIMARKS host-associated metagenomic sample guidelines of Yilmaz et al. [129]. All statistical analysis scripts were executed in R V4.4.0 and are publicly available at [130]. The datasets supporting the results of this article are available in GigaDB v4.4.7 [131]. An archived software heritage snapshot of the LaborOME project has been made publicly available [132].

## Additional Files

**Supplementary note 1**. Study site description

**Supplementary note 2**. Descriptive summary of microbiome sequencing results

**Supplementary note 3**. Descriptive summary of target-enriched shotgun sequencing results

**Supplementary note 4**. Network analysis results

**Supplementary figure 1**. Sequencing parameters of 16s rRNA Illumina MiSeq samples including **a** sequenced read depth (y-axis) and **b** Phred quality (y-axis), displayed as boxplots for worker collection phases and contact-matched swine samples (x-axis).

**Supplementary figure 2**. Rarefaction analysis displaying the discovery rate of unique ASVs (y-axis) at varying depths of 16s rRNA Illumina MiSeq sequencing (x-axis).

**Supplementary figure 3**. Relative abundance of the **a** top 30 phyla, **b** top 10 phyla, **c** top 30 classes, and **d** top 10 classes of taxonomic assignment of microbiomes across workday collection phases, swine, and environmental samples. Barplots for **a** and **c** represent relative abundance of features on a sample basis, while **b** and **d** represent mean relative abundance of features across all samples for each collection phase.

**Supplementary figure 4**. Distribution (median and interquartile range) of **a** Phylum richness, **b** Phylum richness and evenness (Shannon diversity index), **c** Genus richness, and **d** and Genus richness and evenness (Shannon diversity index) based on 16S rRNA taxonomic assignment of microbiomes across workday collection phases, swine, and environmental samples. Global statistical differences are assessed via a generalized linear model with Type III ANOVA, followed by Tukey's *post hoc* multiple comparison analysis with FDR adjustment. All richness and diversity measures across all collection phase pairwise comparisons were not statistically different (*p*>0.05).

**Supplementary figure 5**. Volcano plots are used to visualize differential abundance of unique genera in log_2_-fold change (x-axis) and -log_10_*P* value (y-axis) of the global worker skin microbiome between key workshift collection phases: **a** Workday start vs. workday end; **b** Workday end vs. post-shower; and **c** Post-shower vs. workday start. An additional comparison **d** is made between workday end and swine skin samples representing the worker's contact phase. Following the Bayesian-multiplicative approach to impute pseudo counts, variance stabilizing transformation was applied to the microbiome genus-level count matrix as implemented in the *DESeq2* method. Features with significant shift in abundance (Wald's *p*<0.05) are displayed above the horizontal line, while biologically significant fold-change is demarcated by vertical dashed lines at 1.5 log_2_-fold change. Labels are displayed for up to five most abundant genera in the comparison significantly amplified between each phase pair being compared. The total number of genera in common for each comparison is displayed below each plot.

**Supplementary Figure 6. Workday fluctuations in worker skin resistome and mobilome composition, compared to contact-matched swine**. Principal component ordination of robust Aitchison compositions for the **a** total resistome and **b** major components of the total mobilome, including plasmids, integrative conjugative elements, virus, prophage, insertional sequences, and transposable elements. Clustering significance was explored with the omnibus analysis of similarity (ANOSIM) test (*p*<0.05). Ordinations are summarized by depicting within-group centroids and shaded ellipsoids representing the 90% confidence interval. The distribution of major mobilome accessions are displayed as relative abundance stacked bar graphs across collection phases and individual samples for **c** major MGE mechanisms; **d** plasmidic sequences; and. **e** insertional sequence family identity. Legend abbreviations include ICE: integrative conjugative element, IS: insertional sequences, Plasmid ARG: Plasmid-borne antimicrobial resistance genes, TE: Transposable elements; PREPIM: Plasmid replication initiation and maintenance; PTTREG: Plasmid transcription, translation, and regulation.

**Supplementary figure 7**. Distribution (median and interquartile range) of **a** ARG group richness, **b** ARG group richness and evenness (Shannon diversity index), **c** Clinically important ARG group richness, and **d** and Clinically important richness and evenness (Shannon diversity index) based on target-enriched shotgun metagenomic sequencing of samples collected across workday phases, swine, and environmental samples. Global statistical differences are assessed via a generalized linear model with Type III ANOVA assessment, followed by Tukey's *post hoc* multiple comparison analysis with FDR adjustment. Pairwise significance is denoted by * (*p*<0.05).

**Supplementary figure 8**. Distribution of total **a** ARG and **b** MGE abundance as determined by target-enriched shotgun metagenomic sequencing and normalized using 16S rRNA gene copy in samples collected across workday phases, swine, and environmental samples. Abundance is expressed on a log_10_-basis to adhere to normality assumptions and assist in visualization. Global statistical differences are assessed via a generalized linear mixed model with Type III ANOVA assessment, followed by Tukey's *post hoc* multiple comparison analysis with FDR adjustment. Pairwise significance is denoted by ** (*p*<0.001), *** (*p*<0.0001).

**Supplementary figure 9**. Relative abundance of discrete strains found across all worker, swine, and environmental samples using StrainPhlAn on shotgun metagenomic data.

**Supplementary figure 10**. Maximum likelihood phylogenetic trees of species shared across collection phases, profiled using StrainPhlAn. Detection of species-level genomes was performed using a minimum of a 1% prevalence threshold across all study samples.

**Supplementary table 1**. Descriptive statistics of the study site swine worker cohort, abstracted from an administered questionnaire

**Data S1. (Supplementary_datafile_1_metadata.csv)**

Study sample metadata and associated questionnaire response data

**Data S2. (Supplementary_datafile_2_microbiome.data.file.counts.csv)**

Microbiome 16S rRNA amplicon sequencing count matrix

**Data S3. (Supplementary_datafile_3_microbiome.data.file.taxonomy.csv)**

Microbiome 16S rRNA amplicon sequencing taxonomy matrix

**Data S4. (Supplementary_datafile_4_DESeq_results_microbiome.csv)**

Microbiome 16S rRNA amplicon sequencing differential abundance analysis results

**Data S5. (Supplementary_datafile_5_resistome.data.file.counts.csv)**

Shotgun target-enriched metagenomic sequencing resistome gene count matrix

**Data S6. (Supplementary_datafile_6_resistome.data.file.annotation.csv)**

Shotgun target-enriched metagenomic sequencing resistome gene annotation matrix

**Data S7. (Supplementary_datafile_7_mobilome.data.file.counts.csv)**

Shotgun target-enriched metagenomic sequencing mobilome gene count matrix

**Data S8. (Supplementary_datafile_8_mobilome.data.file.annotation.csv)**

Shotgun target-enriched metagenomic sequencing mobilome gene annotation matrix

**Data S9. (Supplementary_datafile_9_StrainPhlAn_analysis.counts.csv)**

Relative abundance analysis results of strains identified by StrainPhlAn following shotgun target-enriched metagenomic sequencing

**Data S10. (Supplementary_datafile_10_DESeq_results_resistome.csv)**

Shotgun target-enriched metagenomic resistome sequencing differential abundance analysis results

**Data S11. (Supplementary_datafile_11_DESeq_results_mobilome.csv)**

Shotgun target-enriched metagenomic mobilome sequencing differential abundance analysis results

**Data S12. (Supplementary_datafile_12_MAG_metadata.csv)**

Metagenomic assembled genome phylogenetic analysis and metadata

## Abbreviations

ANOSIM: analysis of similarities test; ANOVA: analysis of variance; ARG: antimicrobial resistance gene; ASV: amplicon sequence variant; CoNS: coagulase-negative Staphylococci; FDR: false discovery rate; gDNA: genomic DNA; GTDB: Genome Taxonomy Database; ICE: integrative conjugative element; IS: insertional sequence; MAG: metagenome-assembled genome; MGE: mobile genetic element; MRSA: methicillin-resistant *Staphylococcus aureus*; PBS: phosphate-buffered saline; PCA: principal component analysis; PERMANOVA: permutational multivariate analysis of variance; PPE: personal protective equipment; qPCR: quantitative PCR; SGB: species-level genome bin; SPIEC-EASI: SParse InversE Covariance Estimation for Ecological Association Inference; StARS: Stability Approach to Regularization Selection; TE: transposable element; UMGC: University of Minnesota Genomics Center.
