# Supplementary material for: Reducing skin microbiome exposure impacts through swine farm biosecurity
Source: Gigascience. 2025 Jul 26;14:giaf062. doi: 10.1093/gigascience/giaf062 (PMC12810053; doi:10.1093/gigascience/giaf062)
Supplement: giaf062_Supplemental_Files [file giaf062_supplemental_files.zip › Supplementary_Materials.docx]

**SUPPLEMENTARY NOTES**

**Supplementary note 1.** Study site description

***General study site characteristics***

Study enrollment, sampling, and administration of questionnaires was conducted in the Spring of 2021 at a commercial swine farm located in the USDA designated Southwestern agricultural district of Minnesota, USA. Land use around this farm includes predominantly open corn and soybean cropland with surrounding livestock operations mainly (> 53%) focused on pig and hog production. The closed ‘*all-in-all-out*’ farm system is centered on farrowing and weaning phases with >20 years of consistent operation. The farm houses >3500 sows with a median 10 piglets per litter. Mean weaning age of piglets is at 22.5 days. The facility employs routine monitoring for specific swine pathogens including porcine reproductive and respiratory syndrome virus (PRRSV), swine influenza type A virus (SIAV), and porcine epidemic diarrhea virus (PEDV). During the sampling period, herds tested negative for these viruses. The farm has never reported a positive PEDV case in its operational history. However, a PRSSV challenge among a portion of its herd was used as part of a research project conducted in 2014. The farm utilizes negative pressure ventilation and a multi-stage filtration system. During the collection phase of the study, antimicrobial drugs were utilized to control occasional microbial infections among individual sows or piglets. These included (in order of largest to smallest administered antimicrobials in total grams): chlortetracycline> ampicillin> lincomycin>enrofloxacin>spectinomycin> penicillin>tylosin>Oxytetracycline. The farm deploys a number of decontamination products to sanitize and control density of infectious agents in the farm environment. These include bactericidal and virucidal quaternary ammonium compound-containing sanitizers for hard non-porous surfaces, lignocellulose drying powder for moisture control, sodium hypochlorite bleach, 2% chlorhexidine solution, calcium salt hypochlorite, chloride, and hydroxide-based foot pan powder, and clay mineral-based sanitizing powder.

***Farm worker characteristics***

Farm personnel were day-shift workers with similar start and end workday schedules. These workers were approached for enrollment in part due to their familiarity with performing self-collection of swab samples according to a standard set of procedures. The worker cohort is predominantly male, with a median age of 33 years, have achieved a minimum of a high school education, identified as either White or of Hispanic / Latin American, were not active smokers at the time of sample collection and only two participants have reported to ever smoke tobacco (Supplementary table 1). More than 70% of the participants reported handling or eating pork, chicken, or beef products, and while some participants reported raising domestic animals or keeping companion animals, no participants raised or handled live swine outside of their workplace. Workers at this facility have been employed and engaged in direct swine contact for a median of 4 years, though workers at this facility have a median of 8 years of working experience requiring direct contact with swine.

We conducted site visits to catalog the range of tasks performed by workers at the facility (**Supplementary table 1**). We were unaware of any existing microbiome-based workplace exposure assessment protocols validated for agricultural contexts, and therefore we relied on external review by an occupational medicine practitioner with expertise in agricultural worker health, to dichotomize potential microbiological exposures associated with each facility task as either likely ‘*direct*’ or ‘*indirect*’ exposure to swine microbiota. Based on the balance of tasks performed by each participant identified via questionnaire, we then assigned each worker to either the likely direct or likely indirect exposure groups. Though the small sample size in this study is underpowered to handle imbalanced observations among exposure groups (direct exposure *n*= 7; indirect exposure *n*= 3), we nevertheless followed our *a priori*-defined analytical plan to test the hypothesis that heterogeneity in skin microbiota may be explained by facility task exposure factors. Analysis of microbiota across all collection phases combined and at each individual phase T1–T3 reveals marginal clustering by direct or indirect exposure at Phylum and Genus levels, though these separations were not statistically significant (Phylum and all Genus PCA ordinations, PERMANOVA *p*> 0.05).

***Farm worker procedures***

Components of the farm are segregated by a functional ‘line-of-separation’ (LOS) system which partitions the farm into three zones: A) ‘***Transitional zone***’ with compulsory biosecurity procedures designated to enter or leave the facility proper (protocol outlined below); B) ‘***Clean zone***’ which includes areas designated for office space, breakroom / kitchen, lavatory, and laundry facilities; and C) ‘***Production zone***’ characterized by portions of the facility dedicated strictly to animal holding and husbandry (e.g. breeding / gestation and farrowing / nursing rooms). Within the transitional zone, a one-way (i.e. unidirectional) flow of personnel is used to enter and exit the facility.

Upon entering the facility the following protocol is followed in the transitional zone prior to entry into clean and production zones of the farm proper:

1. Record date and time of entrance, recent history of swine exposure outside of the facility, and any syndromic / health details
2. Inanimate objects to be brought into the facility must be decontaminated with disinfecting wipes and placed into the UV-C light box for 15 minutes of conditioning
3. Individual must sit on a bench and swing body / feet around to the transitional zone without touching the floor of the entrance / ‘dirty’ area
4. Once crossed into the transitional zone, workers must enter private changing areas to remove all clothing, undergarments, and jewelry
5. Workers must shower before exiting into the opposite (‘clean’) side of the shower. Showering procedures include:
   1. Lathering and rinsing the body and hair using soap and shampoo
   2. Utilizing a brush and soap to completely clean hands and fingernails
6. Dedicated towels, undergarments, coveralls, and hair protection provided by the facility are donned on the ‘clean’ side of the showers, and personnel continue into the clean zone.

If for any reason an individual must cross back into the ‘dirty’ side of the shower or anywhere in the transitional zone, the individual is required to repeat showering steps before entering the other zones. After donning dedicated boots, personal protective equipment (PPE), as well as any other optional hair, eye, and ear protection, workers may transition between the clean zone and the production zone. Returning from the production zone into the clean zone requires decontamination and removal of boots / footwear, removal and disposal of all PPE, and washing or sanitizing hands.

The following procedures are followed into the transitional zone after exiting the clean / production zones of the farm proper:

1. Inanimate objects to be brought out of the facility must be decontaminated with disinfecting wipes and placed into the UV-C light box for 15 minutes of conditioning
2. Workers must enter the transitional zone changing rooms and remove all facility-provided garments to be laundered
3. Workers must enter the shower from the ‘clean’ side and follow showering procedures as previously described
4. After showering and toweling off while still in the shower, workers are to dispose of the used towel for laundering and step into the opposite side (i.e. away from the ‘clean’ side) to donn original clothing and undergarments
5. After stepping out of the changing/showering area, workers are to exit the transitional zone by swinging legs / feet over the bench toward the exit side of the bench.
6. Exit date and time are to be recorded
7. After putting on dedicated shoes and protective covering, workers are to exit the facility

Workers on this farm are provided a wide latitude of personal care products, soaps, and detergents that could be utilized in the showering phases, and no controls are implemented to account for inter-individual variation in body washing (i.e. duration and frequency of lathering, scrubbing, rinsing, drying, etc.). All sanitary products, as well as barrier protections, work clothes, undergarments, and towels are provided by the facility and remain on the premises following a period of initial conditioning and UV-C irradiation in pass-through chambers. All garments and towels are washed on-premises after each use.

**Supplementary note 2.** Descriptive summary of microbiome sequencing results

Microbiome-level V3-V4 16S rRNA sequencing of individually collected longitudinal worker skin and worker contact-matched swine skin microbiota, was performed across 40 swab specimens, yielding a total of 3.27 M paired-end MiSeq reads across the study with a median of 76,560 paired-end reads per sample (IQR: 34,766). An additional two swab samples (termed ‘Environment’) were collected following exposure to ambient air in both gestational (i.e. maternal swine pen) and farrowing (i.e. birthing and nursing pens) areas, yielding 257,607 paired-end reads. Quality trimming resulted in the median removal of 23.3% (IQR: 2.0) per sample. Balanced sequencing depth was achieved across collection phases of worker samples and swine samples (Type III ANOVA *p*> 0.05 with Tukey’s *post hoc* and FDR adjustment). Phred quality >30 was achieved across all samples with median Phred score of 31.8 per sample (IQR: 0.275) and was statistically balanced (Type III ANOVA *p*> 0.05 with Tukey’s *post hoc* and FDR adjustment). Following removal of contaminants sequences, reads across all samples were merged to generate the ASV table containing counts for 6,840 unique ASVs that were identified across all samples.

**Supplementary note 3.** Descriptive summary of target-enriched shotgun sequencing results

Targeted shotgun metagenomic sequencing formed the basis of the resistome analysis to ascertain if the contingent of ARGs in swine worker metagenomes coincided with shifts in ecological composition and structure of microbial communities throughout the workday. The integrated hybridization-enrichment protocol with shotgun sequencing, was chosen to overcome known barriers to functional gene profiling of samples collected from skin and cutaneous adnexa replete with host DNA (i.e. off-target DNA). Across all samples (n=42), a total of 2.91B paired-end raw short reads were generated with a median of 67.2M reads per sample (IQR: 10.7M). Filtering for unpaired and low quality reads resulted in the median removal of 7.2% (IQR: 6.8) reads per sample, and across all samples <1% of raw reads were filtered due to quality alone. Fragment quality scores were statistically balanced across all samples for forward and reverse fragments, ranging 35.1–37.1 and 32.6–36.8, respectively (Type III ANOVA *p*> 0.05). The overall raw sequencing depth was statistically balanced for metagenomes collected across all phases of work in worker samples, swine samples, and ambient air. However, after filtering host-mapped reads, a proportionally greater sequencing depth was achieved for swine samples (Median[IQR]: 39.4M[12.8]) relative to all worker sample types: 17.5–31.7M[6.8–9.1] (Type III ANOVA *p*< 0.0001 with Tukey’s *post hoc* and FDR adjustment), and among worker metagenomes, a marginally greater depth of sequencing was achieved for samples collected at T3 vs T1 (22.2M[9.1] vs. 17.5M[7.8], Type III ANOVA *p*= 0.046 with Tukey’s *post hoc* and FDR adjustment). As expected, a significantly greater proportion of host reads mapping to the human genome were recovered from workday start (Median[IQR]: 66.3%[11.0]) and workday end swabs (66.1%[11.2]), relative to Post-shower swabs (48.3%[14.2]) (Type III ANOVA of lmm *p*<0.0001 with Tukey’s *post hoc* and FDR adjustment). Consistent with previous surveys of mammalian skin and environmental microbiota[(48)](https://www.zotero.org/google-docs/?QkFnGs), when accounting for sequencing depth, a significantly lower proportion of swine and facility air metagenomes mapped to the host genome (*S. scrofa*) relative to those of the human subjects (Median[IQR]: 47.4%1[6]) and 28.1%[3.75], respectively, Type III ANOVA of LMM *p*<0.0001 with Tukey’s *post hoc* and FDR adjustment).

Target enrichment resulted in a median of 14% of generated sequence reads in each library originating from ARG DNA (IQR: 14.6%), with a median ARG-containing read duplication rate of 11.7% per library (IQR: 14.1%) (**Supplementary datafile 1**). The enriched data consisted of 3,100 non-redundant canonical ARGs across all samples, of which 2,749 required no additional SNP confirmation, and thus were included in resistome analyses (**Supplementary datafile 5,6**). Based on the MEGARes(v. 2.0) ontology[(5)](https://www.zotero.org/google-docs/?WZlDLz), this set of ARGs represents 685 unique ARG groups known to confer resistance to 44 unique classes of antimicrobial drugs, metals and biocides, via 131 unique mechanisms of action. For MGE enrichment, 15.9% of generated sequence reads originated from the 748 targeted MGEs (median per sample: 10%, IQR: 10.1%).

**Supplementary note 4.** Network analysis results

The T1 network consisted of 392 unique ASVs supporting 1,893 edges, which generated the highest neighbor connectivity of all four networks (median node degree [IQR]= 7.0 [8.25]). The most sparse network was identified at the conclusion of the workday (T2), consisting of 253 unique ASVs supporting 750 edges (median node degree [IQR]= 6.0 [3.0]), which represents a >60% reduction in overall edge-node connectivity compared to baseline (T1). This reduced connectivity generally persisted in the post-shower network (T3), which consisted of 308 unique ASVs and 1,052 edges (median node degree [IQR]= 6.0 [4.0]). We explored the nature of taxa interconnectedness by applying community detection procedures based on optimized ‘*spin state*’ configurations[(36)](https://www.zotero.org/google-docs/?uHuuVi) and determined that all worker networks contained readily identifiable subcommunities based on Newman and Girvan[(37)](https://www.zotero.org/google-docs/?QNqDU4) global modularity (*Q*) estimation (**Figure 2d**). The T1 and T3 network comprised the highest number of subcommunities (i.e., 36 and 37, respectively), compared to 30 in T2. Both T1 and T3 displayed slightly higher modularity (*Q*=0.77 and 0.76), than T2 (*Q*=0.73). The worker skin microbiome exhibited moderate interconnectedness within subcommunities during all phases of the workday (Newman’s nominal assortativity [*r_n_*]= 0.58 at T2 vs. 0.52 and 0.51 at T1 and T3) and a notably higher interconnectivity among homophilous influential taxa (Newman’s degree assortativity), suggesting the presence of ecological niches, stability, or a capacity for adaptation even at after an 8-hour period of interfacing with swine and the swine environment, though a moderate attenuation of interconnectivity was observed relative to T1 and T3 ([*r_d_*]= 0.73 at T2 vs. 0.86 and 0.82 at T1 and T3, respectively).

**SUPPLEMENTARY FIGURES**

**Supplementary figure 1.** Sequencing parameters of 16s rRNA Illumina MiSeq samples including **a** sequenced read depth (y-axis) and **b** Phred quality (y-axis), displayed as boxplots for worker collection phases and contact-matched swine samples (x-axis).

**
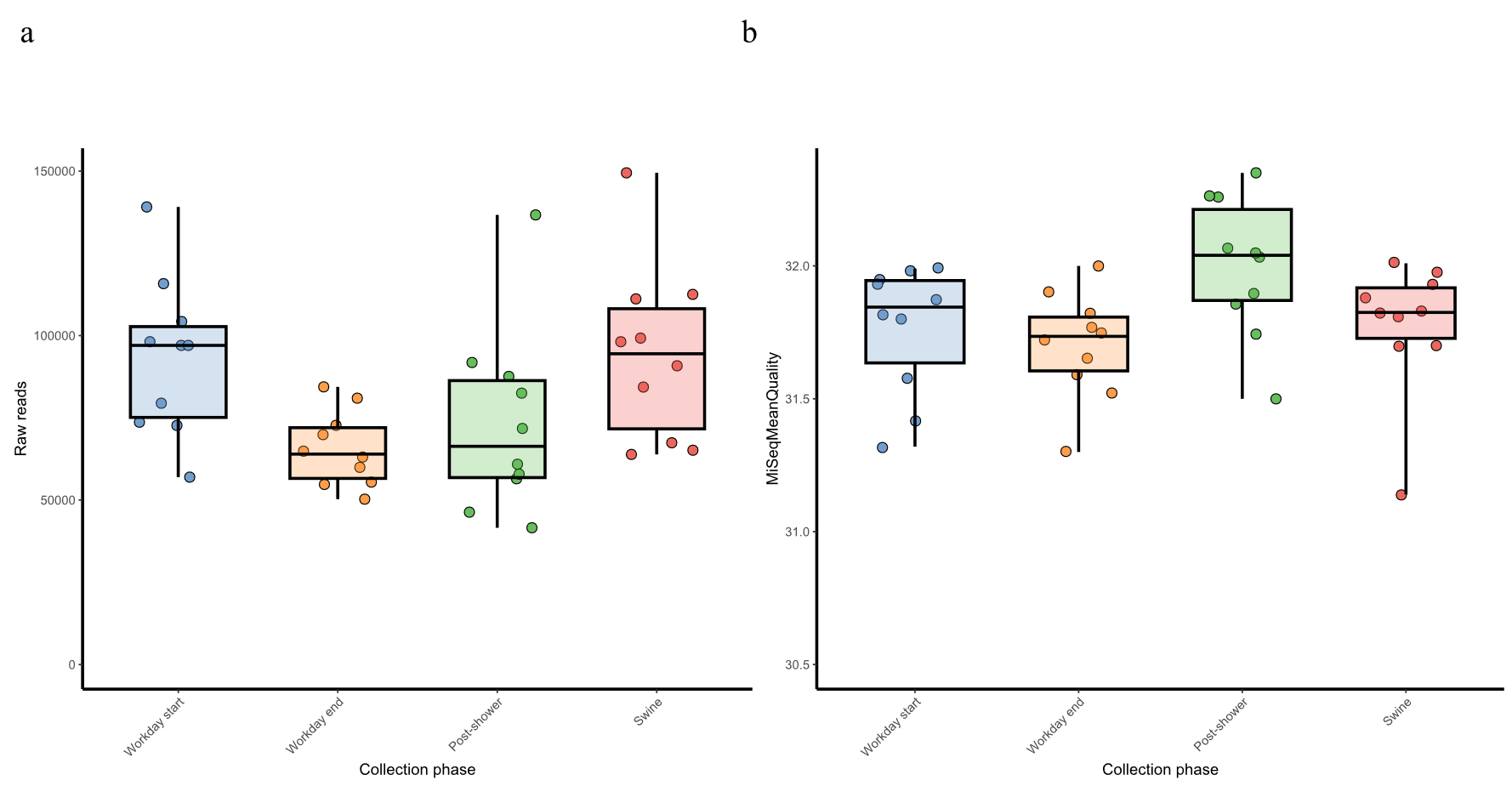
**

**Supplementary figure 2.** Rarefaction analysis displaying the discovery rate of unique ASVs (y-axis) at varying depths of 16s rRNA Illumina MiSeq sequencing (x-axis).

**
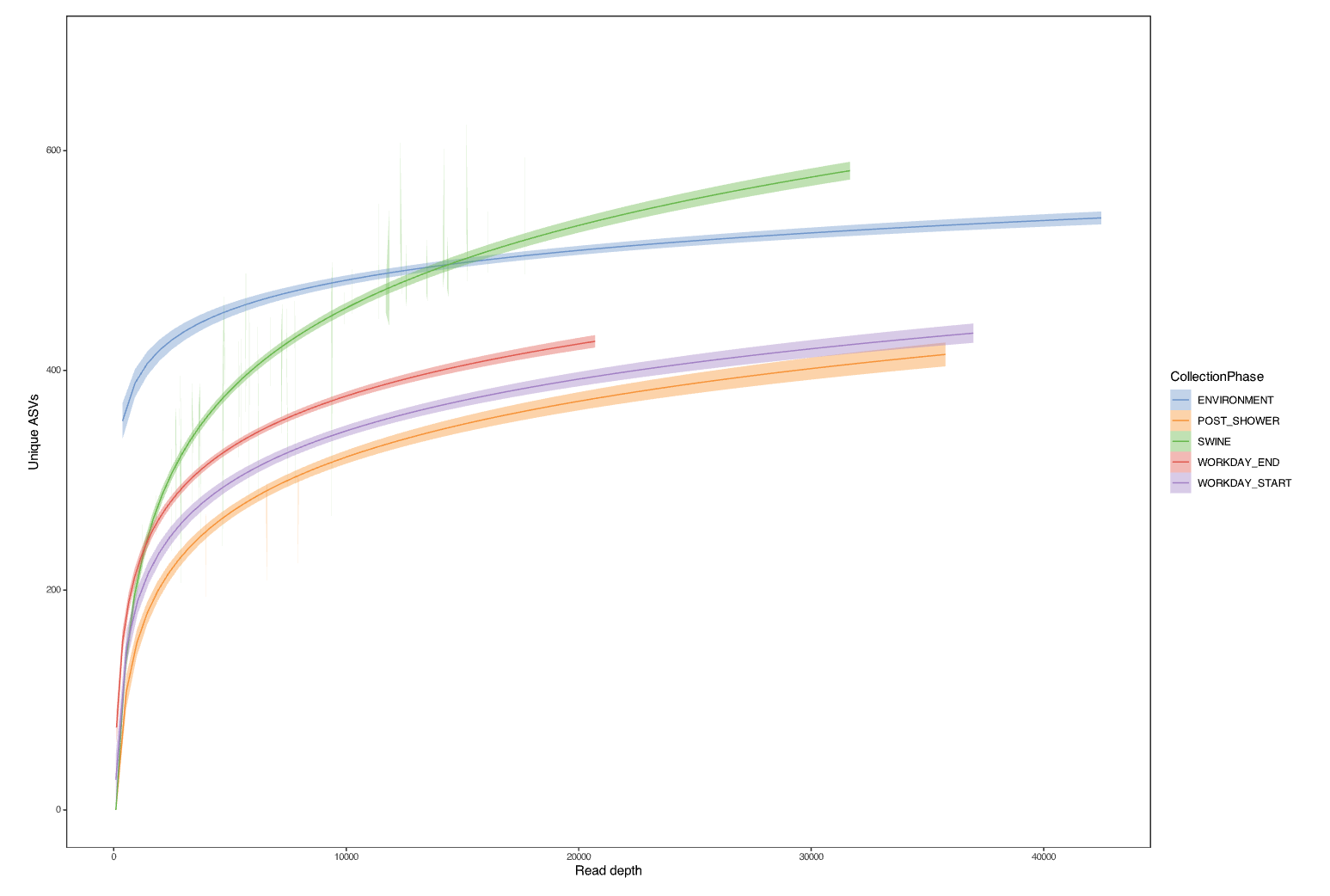
**

**Supplementary figure 3.** Relative abundance of the **a** top 30 phyla, **b** top 10 phyla, **c** top 30 classes, and **d** top 10 classes of taxonomic assignment of microbiomes across workday collection phases, swine, and environmental samples. Barplots for **a** and **c** represent relative abundance of features on a sample basis, while **b** and **d** represent mean relative abundance of features across all samples for each collection phase.


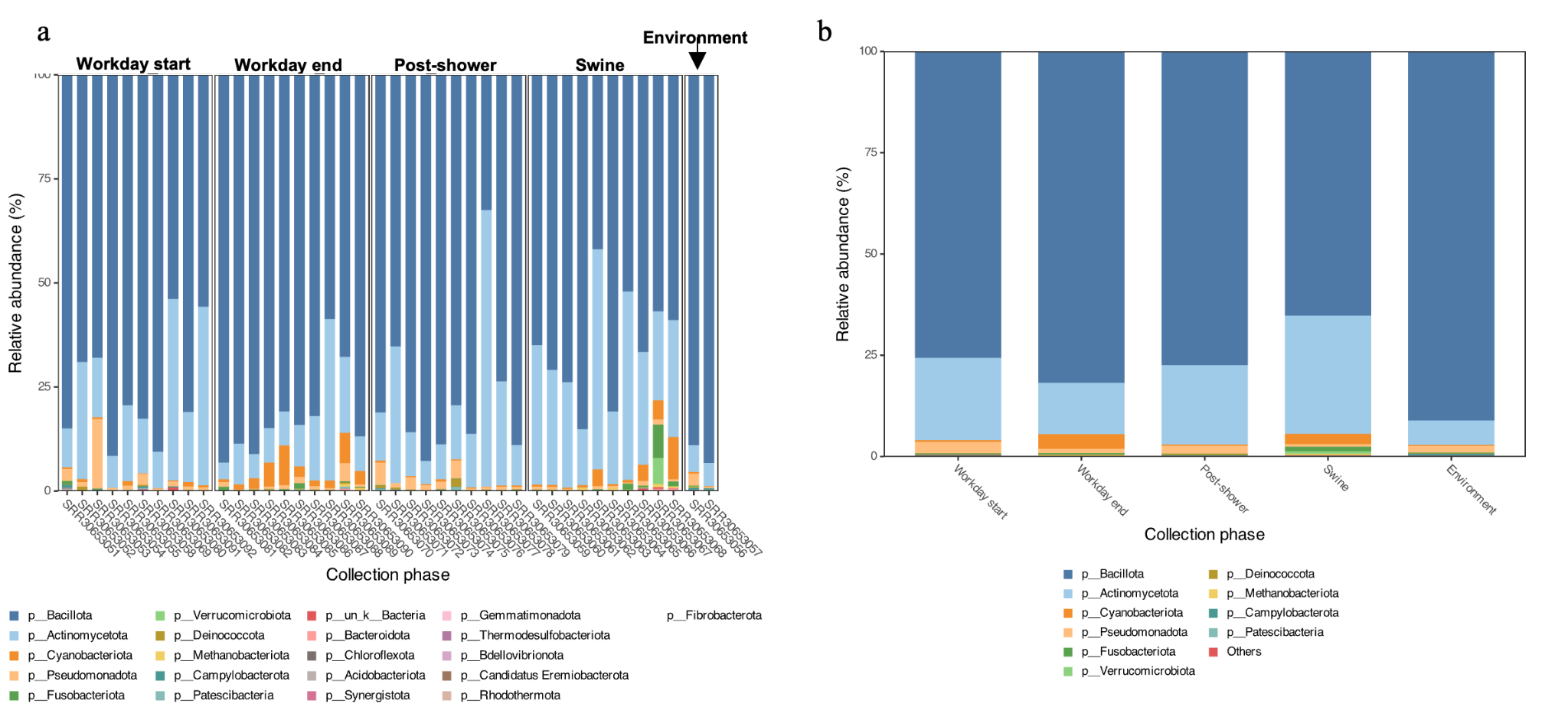

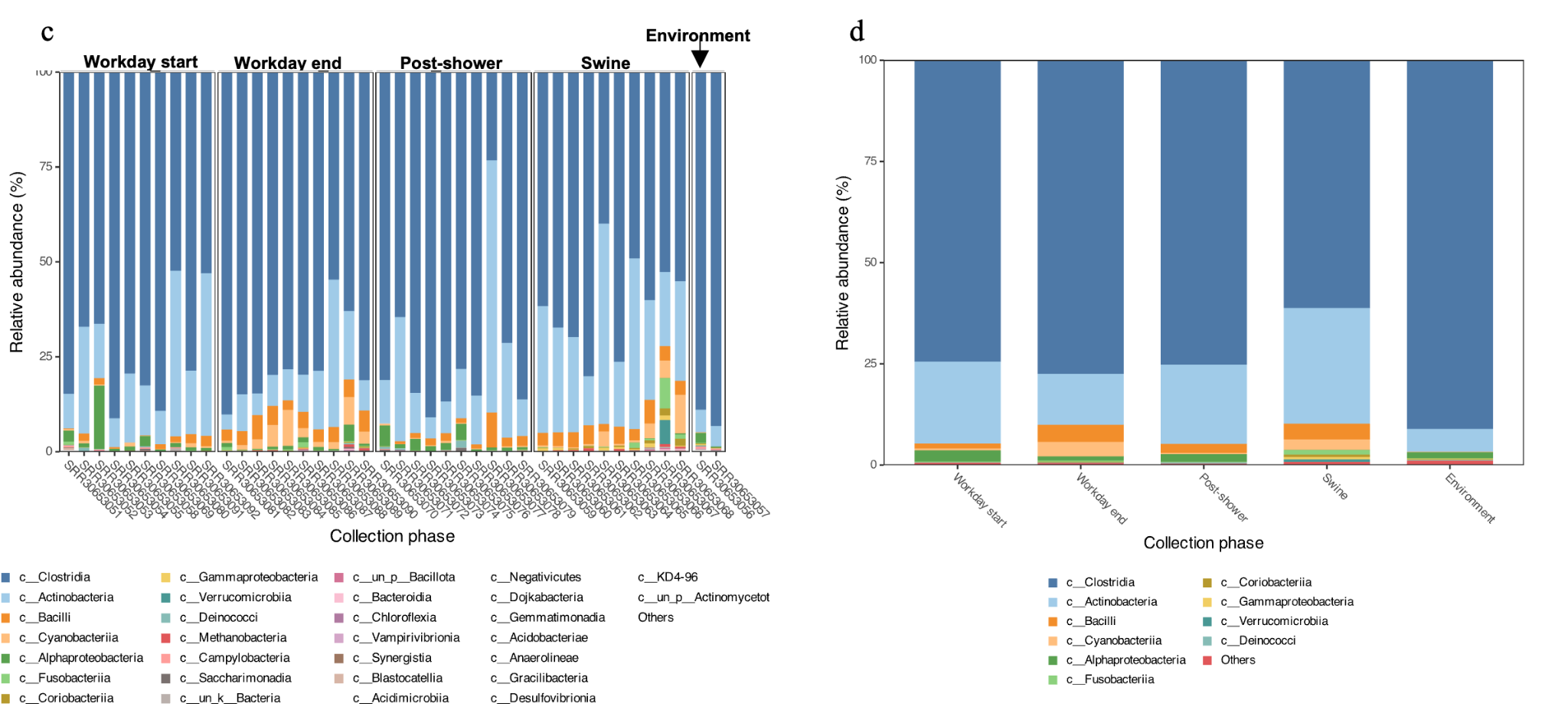


**Supplementary figure 4.** Distribution (median and interquartile range) of **a** Phylum richness, **b** Phylum richness and evenness (Shannon diversity index), **c** Genus richness, and **d** and Genus richness and evenness (Shannon diversity index) based on 16S rRNA taxonomic assignment of microbiomes across workday collection phases, swine, and environmental samples. Global statistical differences are assessed via a generalized linear model with Type III ANOVA, followed by Tukey’s *post hoc* multiple comparison analysis with FDR adjustment. All richness and diversity measures across all collection phase pairwise comparisons were not statistically different (*p*>0.05).

**
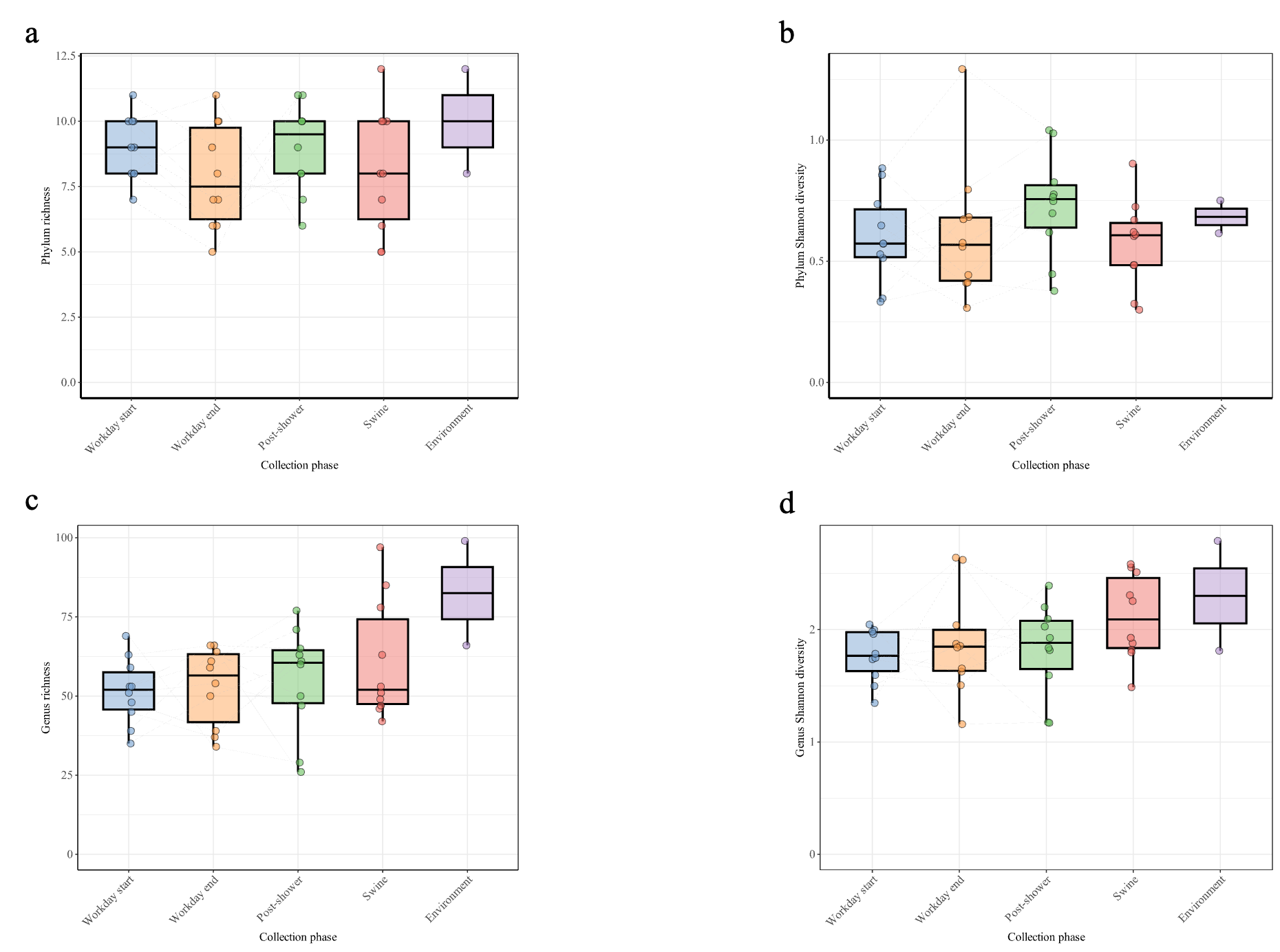
**

**Supplementary figure 5.** Volcano plots are used to visualize differential abundance of unique genera in log_2_-fold change (x-axis) and -log_10_*P* value (y-axis) of the global worker skin microbiome between key workshift collection phases: **a** Workday start vs. workday end; **b** Workday end vs. post-shower; and **c** Post-shower vs. workday start. An additional comparison **d** is made between workday end and swine skin samples representing the worker’s contact phase. Following the Bayesian-multiplicative approach to impute pseudo counts, variance stabilizing transformation was applied to the microbiome genus-level count matrix as implemented in the *DESeq2* method. Features with significant shift in abundance (Wald’s *p*<0.05) are displayed above the horizontal line, while biologically significant fold-change is demarcated by vertical dashed lines at 1.5 log_2_-fold change. Labels are displayed for up to five most abundant genera in the comparison significantly amplified between each phase pair being compared. The total number of genera in common for each comparison is displayed below each plot.


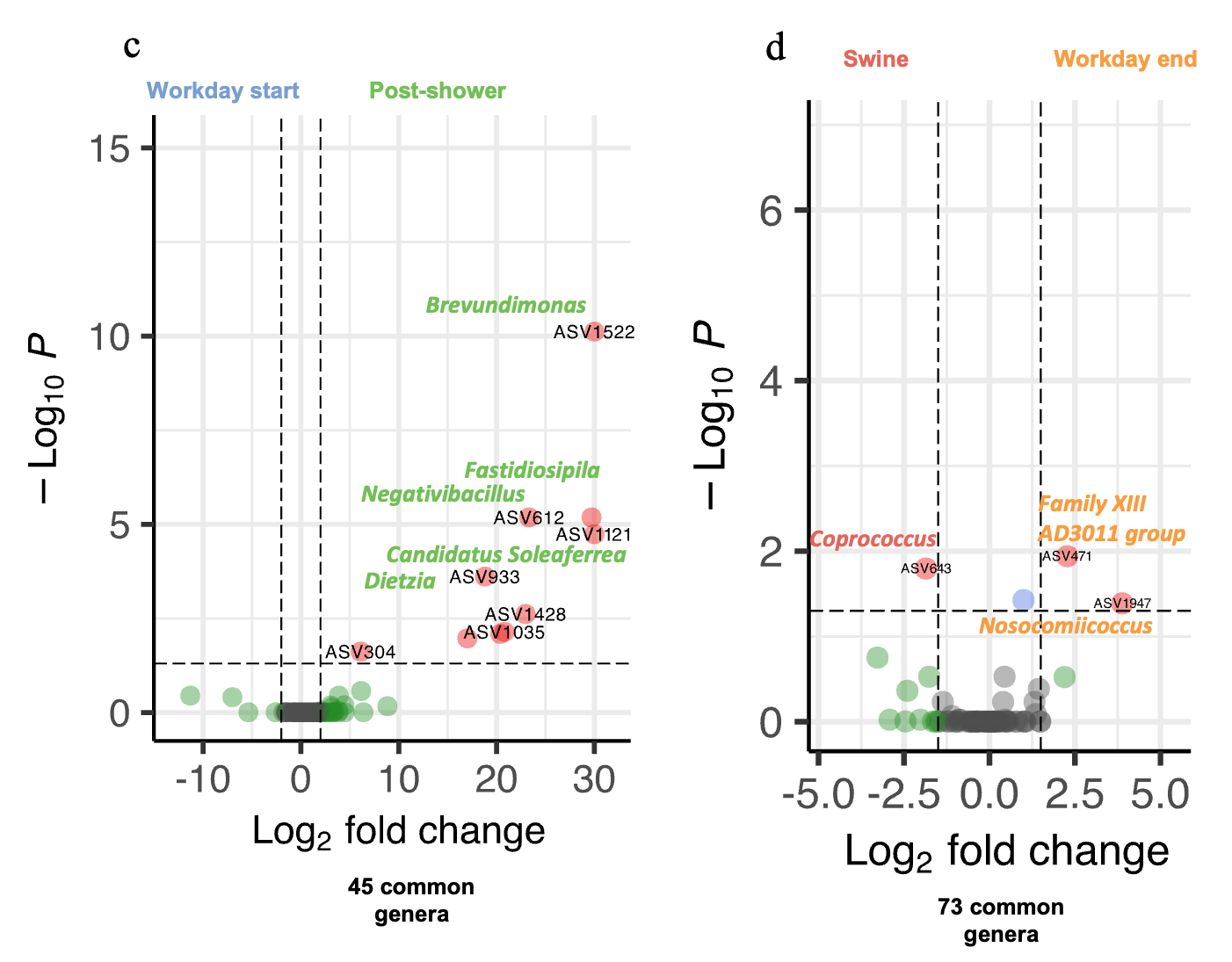


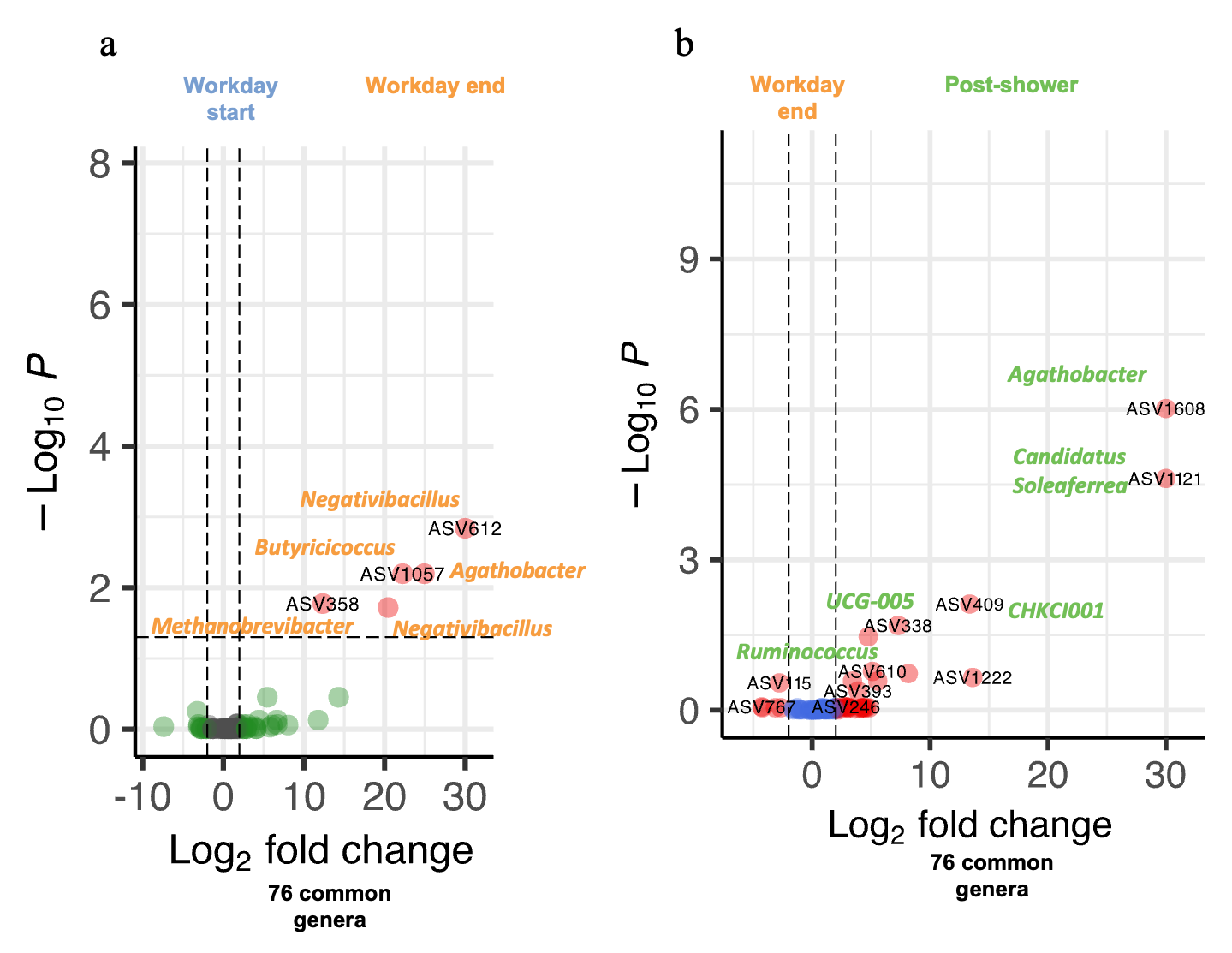


**Supplementary Figure 6. Workday fluctuations in worker skin resistome and mobilome composition, compared to contact-matched swine.** Principal component ordination of robust Aitchison compositions for the **a** total resistome and **b** major components of the total mobilome, including plasmids, integrative conjugative elements, virus, prophage, insertional sequences, and transposable elements. Clustering significance was explored with the omnibus analysis of similarity (ANOSIM) test (*p*<0.05). Ordinations are summarized by depicting within-group centroids and shaded ellipsoids representing the 90% confidence interval. The distribution of major mobilome accessions are displayed as relative abundance stacked bar graphs across collection phases and individual samples for **c** major MGE mechanisms; **d** plasmidic sequences; and. **e** insertional sequence family identity. Legend abbreviations include ICE: integrative conjugative element, IS: insertional sequences, Plasmid ARG: Plasmid-borne antimicrobial resistance genes, TE: Transposable elements; PREPIM: Plasmid replication initiation and maintenance; PTTREG: Plasmid transcription, translation, and regulation.


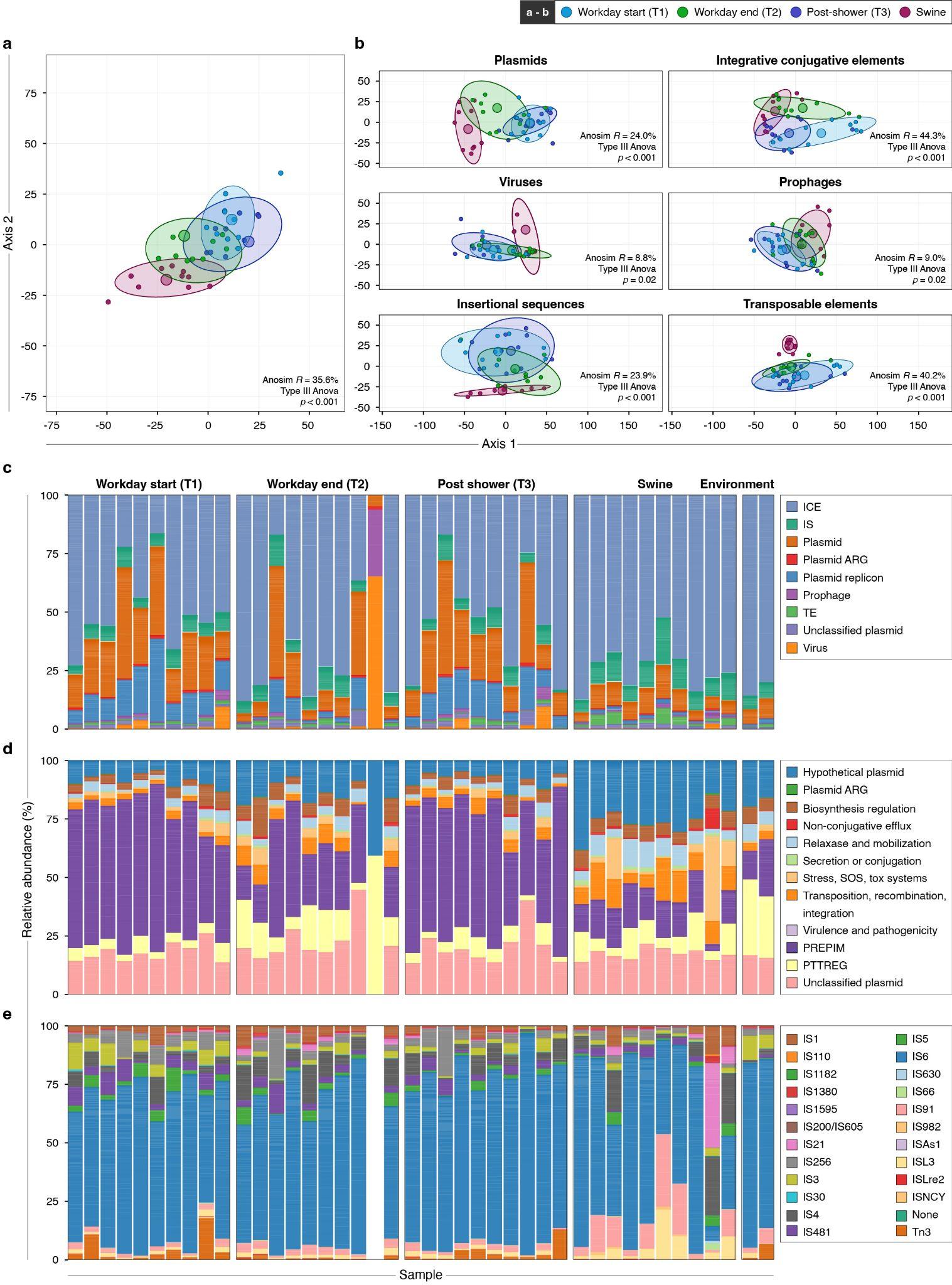


**Supplementary figure 7.** Distribution (median and interquartile range) of **a** ARG group richness, **b** ARG group richness and evenness (Shannon diversity index), **c** Clinically important ARG group richness, and **d** and Clinically important richness and evenness (Shannon diversity index) based on target-enriched shotgun metagenomic sequencing of samples collected across workday phases, swine, and environmental samples. Global statistical differences are assessed via a generalized linear model with Type III ANOVA assessment, followed by Tukey’s *post hoc* multiple comparison analysis with FDR adjustment. Pairwise significance is denoted by * (*p*<0.05).


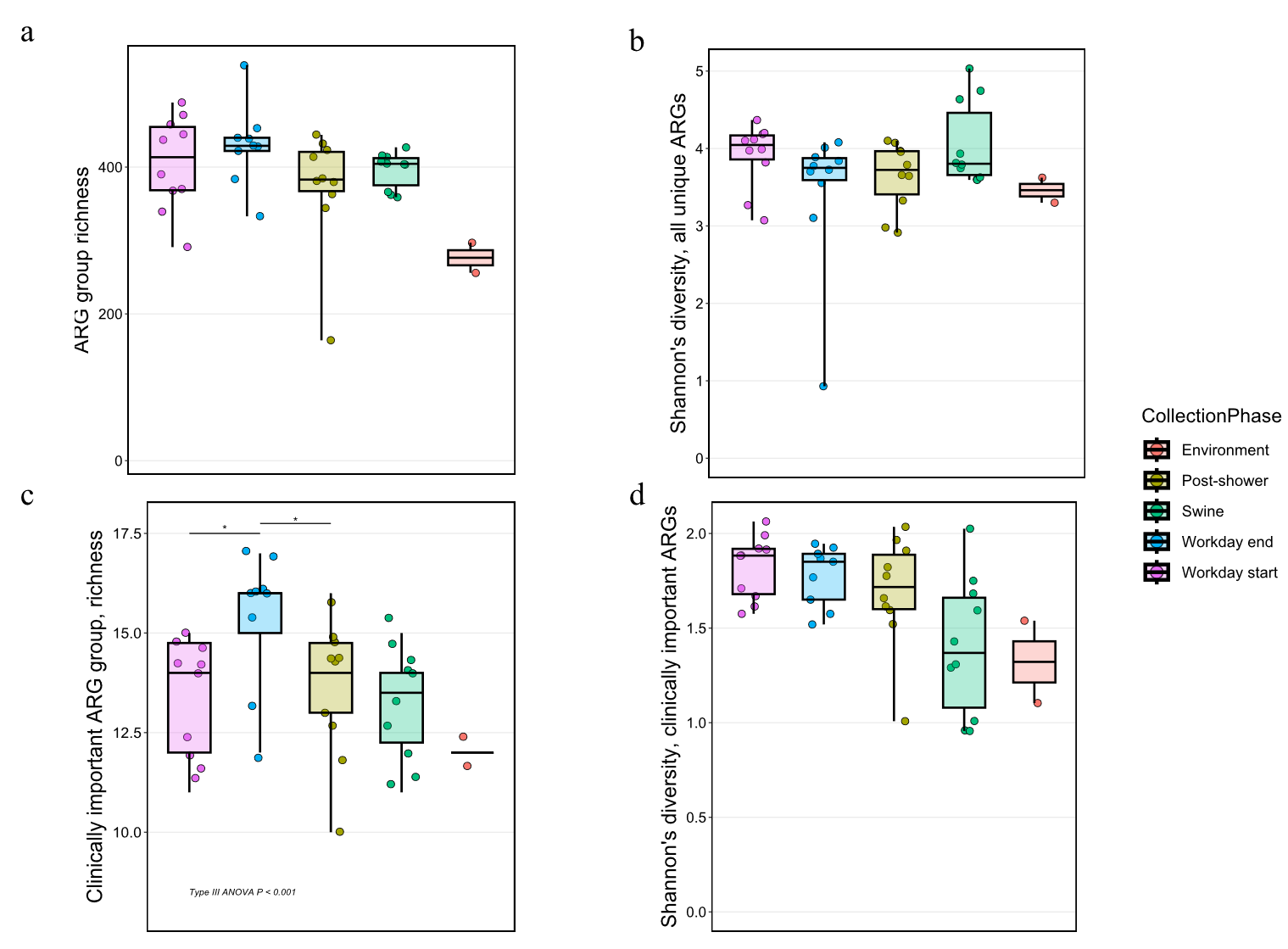


**Supplementary figure 8.** Distribution of total **a** ARG and **b** MGE abundance as determined by target-enriched shotgun metagenomic sequencing and normalized using 16S rRNA gene copy in samples collected across workday phases, swine, and environmental samples. Abundance is expressed on a log_10_-basis to adhere to normality assumptions and assist in visualization. Global statistical differences are assessed via a generalized linear mixed model with Type III ANOVA assessment, followed by Tukey’s *post hoc* multiple comparison analysis with FDR adjustment. Pairwise significance is denoted by ** (*p*<0.001), *** (*p*<0.0001).


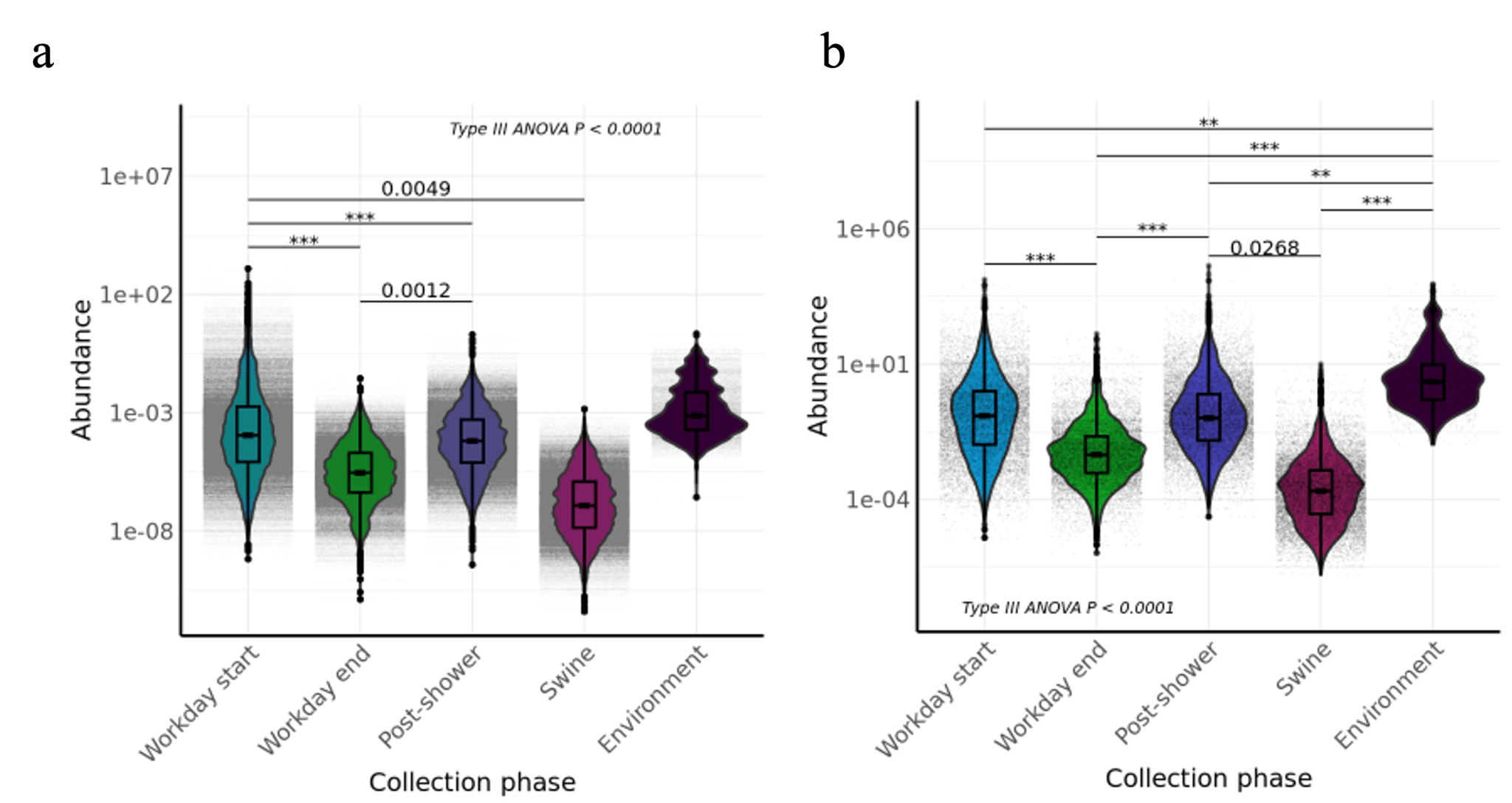


**Supplementary figure 9.** Relative abundance of discrete strains found across all worker, swine, and environmental samples using StrainPhlAn on shotgun metagenomic data.


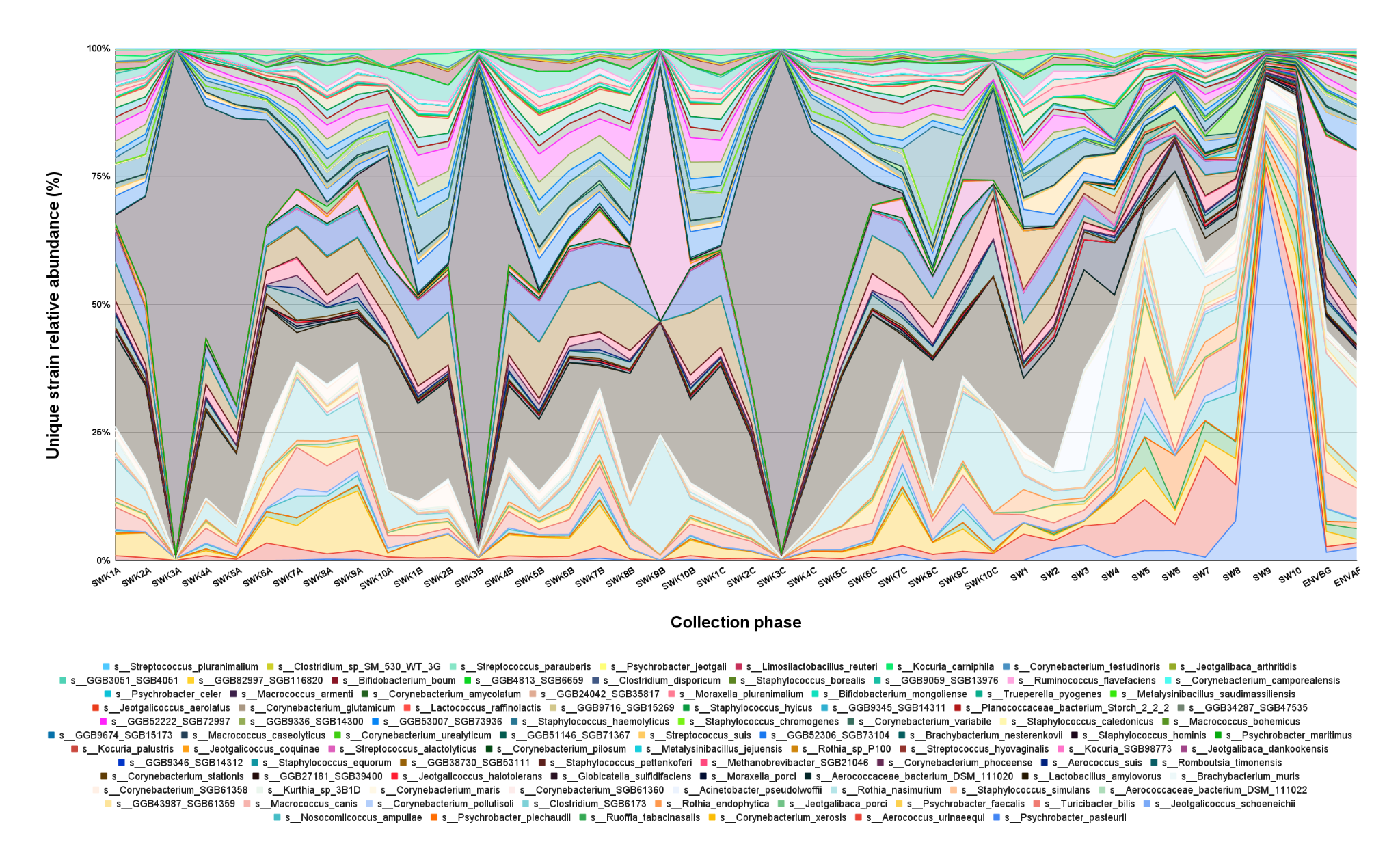


**Supplementary figure 10.** Maximum likelihood phylogenetic trees of species shared across collection phases, profiled using StrainPhlAn. Detection of species-level genomes was performed using a minimum of a 1% prevalence threshold across all study samples.

**
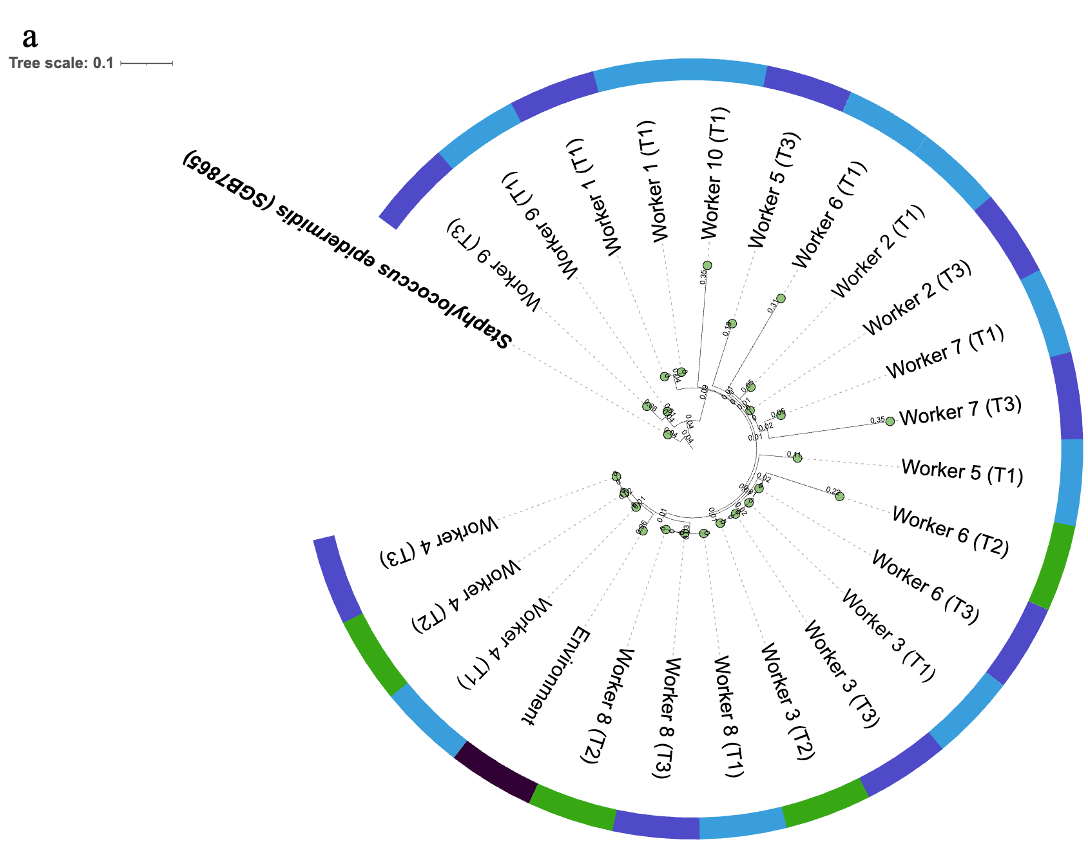
**

**
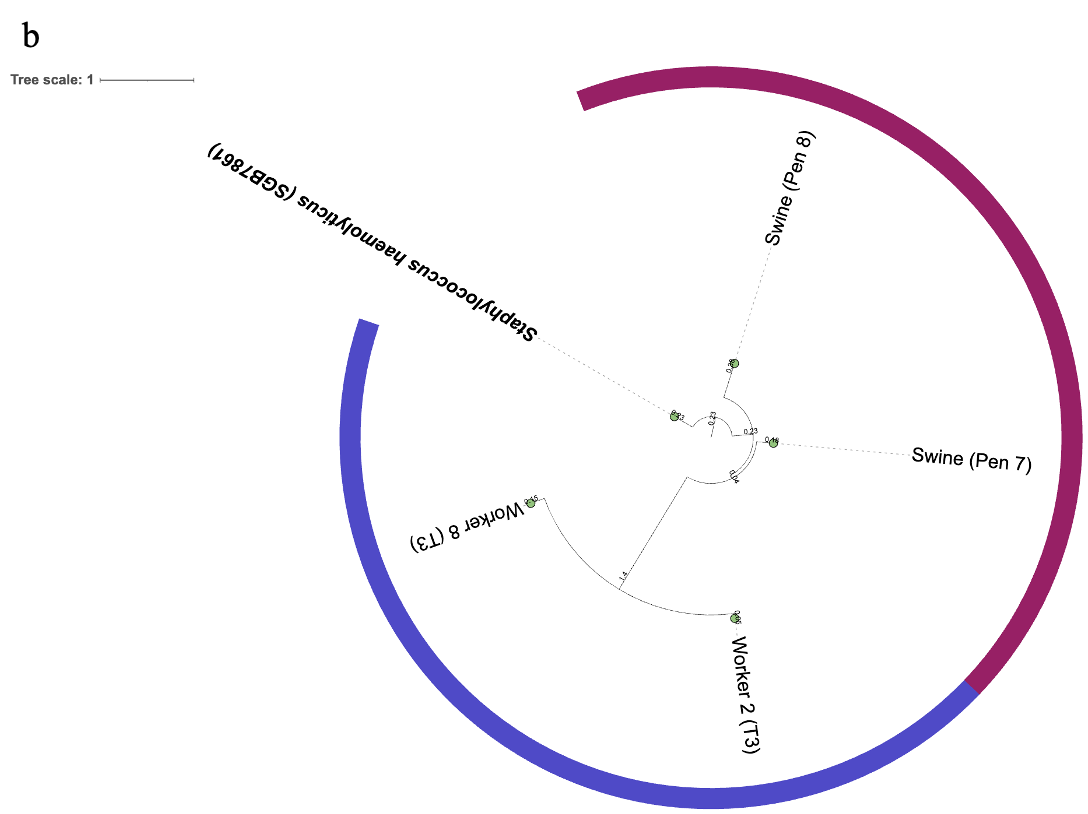
**

**
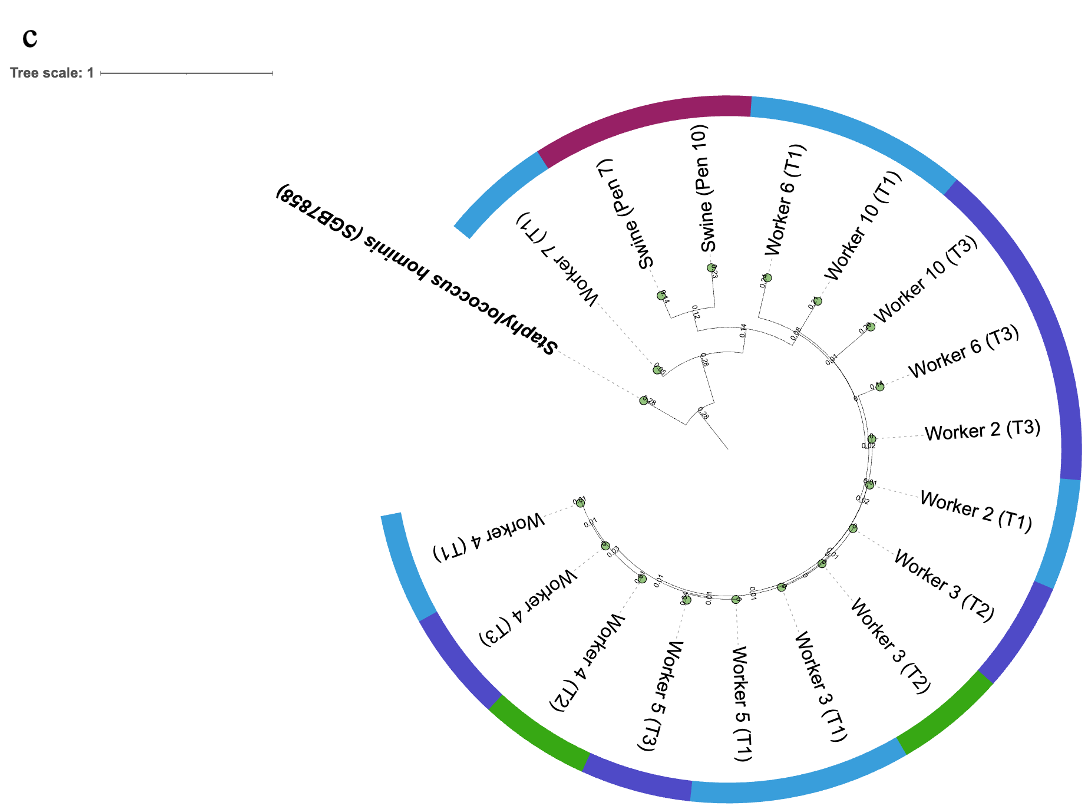
**

**
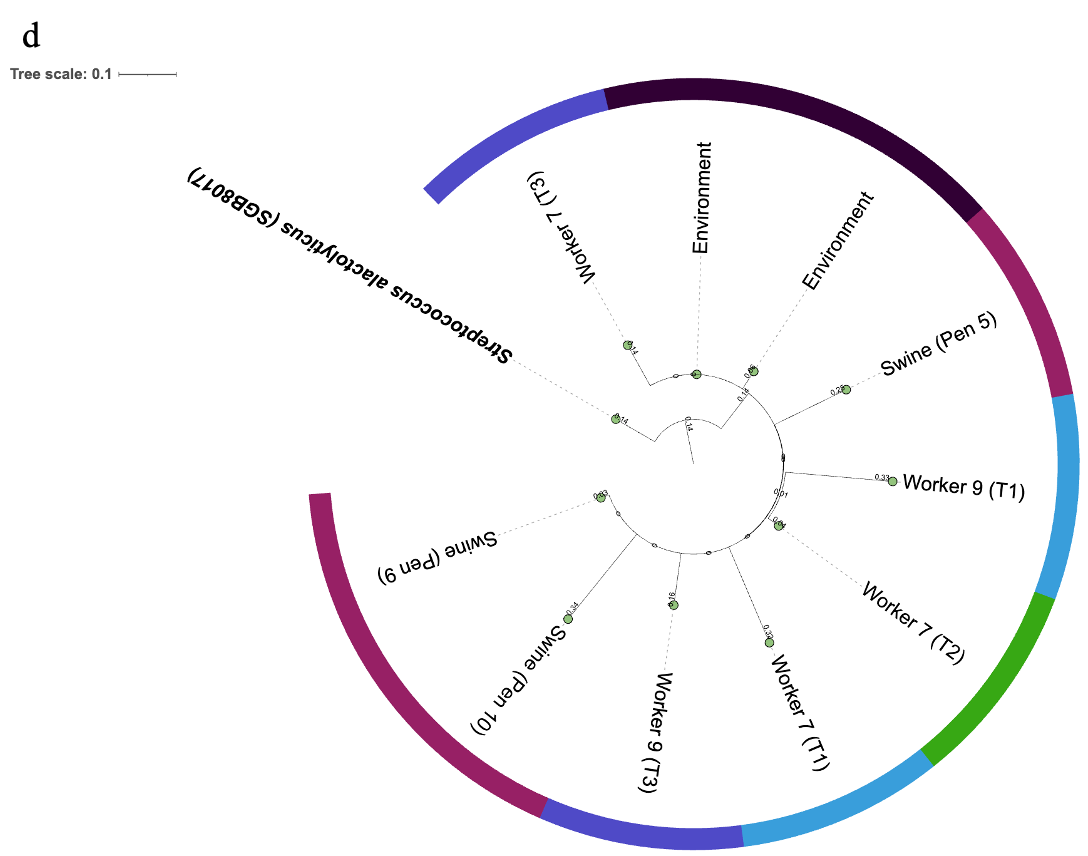
**

**
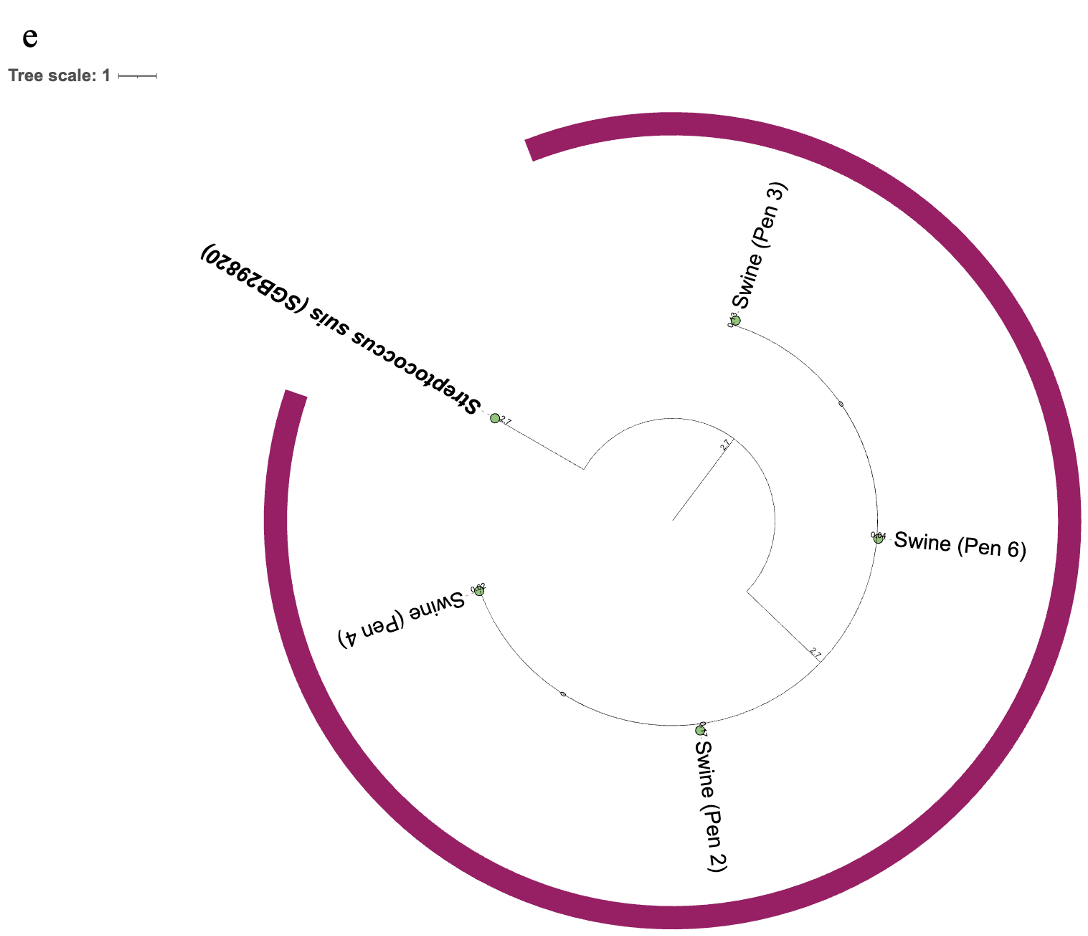
**

**
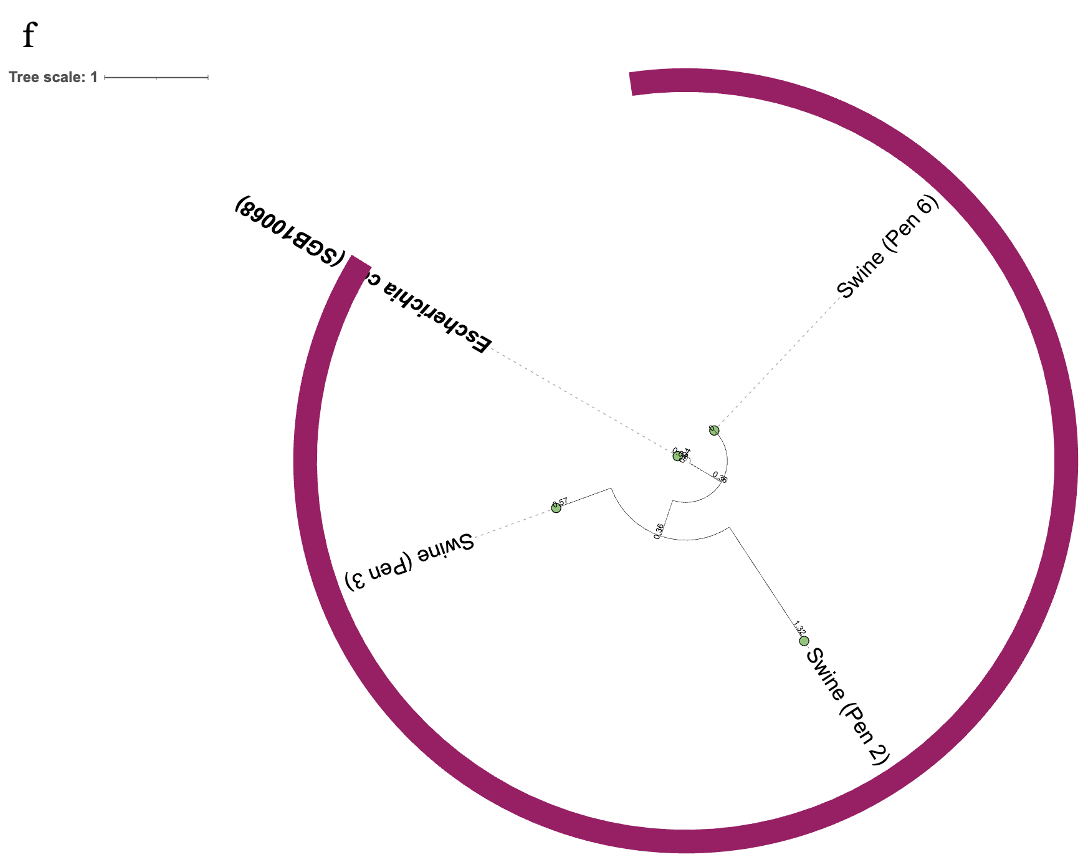
**

**SUPPLEMENTARY TABLES**

**Supplementary table 1.** Descriptive statistics of the study site swine worker cohort, abstracted from an administered questionnaire

| **Study variable** | **Individual parameter (n=10)** |
| --- | --- |
| Age (years, median ± 1sd) | 33 ± 8.7 |
| Biological sex (n) |  |
| Male | 8 |
| Female | 2 |
| Highest education attained (n) |  |
| Up to high school | 2 |
| Up to baccalaureate | 6 |
| Up to graduate / professional | 1 |
| Body mass index (kg/m^2^, median ± 1sd) | 27.5 ± 4.0 |
| Self-identification (n) |  |
| White | 5 |
| Hispanic / Latino | 5 |
| Other | 0 |
| Current smoker (n, yes / no) | 0/10 |
| Ever smoker (n, yes/no) | 2/8 |
| Diet frequency (n, never / >1 per week) |  |
| Pork | 2/8 |
| Beef | 0/10 |
| Poultry | 1/9 |
| Non-occupation handling frequency (n, never / >1 per week) |  |
| Pork | 2/8 |
| Beef | 1/9 |
| Poultry | 3/7 |
| Non-occupational contact, 1 month (n, yes / no) |  |
| Chickens | 2/8 |
| Cattle | 3/7 |
| Swine | 0/10 |
| Equids: Horse, Donkey, Mule | 2/8 |
| Goats | 0/10 |
| Sheep | 1/9 |
| Turkeys | 0/10 |
| Dogs | 5/5 |
| Cats | 4/6 |
| Other | 2/8 |
| Direct swine contact work at facility  (years, median ± 1sd) | 4.0 ± 5.0 |
| Direct swine contact work, all facilities  (years, median ± 1sd) | 8.0 ± 12.9 |
| Hourly swine contact rate (median ± 1sd) | 58 ± 222 |
| Weekly swine contact rate (median ± 1sd) | 325 ± 665 |
| Workday tasks, 1 year (n, yes / no) |  |
| Breeding | 9/1 |
| Farrowing | 4/6 |
| Piglet processing | 7/3 |
| Sow husbandry | 4/6 |
| Barn, stall, room sanitation | 8/2 |
| Euthanasia | 6/4 |
| Transport / move animals | 10/0 |
| Waste disposal, waste decontamination, waste disposal | 9/1 |
| Examining, diagnosing, treating | 7/3 |
| Specimen collection (e.g. blood, urine, feces) | 9/1 |
| Equipment disinfection, cleaning, management | 10/0 |
| General housekeeping in non-swine areas | 6/4 |
| Administrative, clerical, supervisory work | 8/2 |

**SUPPLEMENTARY FILES**

**Data S1. (Supplementary_datafile_1_metadata.csv)**

Study sample metadata and associated questionnaire response data

**Data S2. (Supplementary_datafile_2_microbiome.data.file.counts.csv)**

Microbiome 16S rRNA amplicon sequencing count matrix

**Data S3. (Supplementary_datafile_3_microbiome.data.file.taxonomy.csv)**

Microbiome 16S rRNA amplicon sequencing taxonomy matrix

**Data S4. (Supplementary_datafile_4_DESeq_results_microbiome.csv)**

Microbiome 16S rRNA amplicon sequencing differential abundance analysis results

**Data S5. (Supplementary_datafile_5_resistome.data.file.counts.csv)**

Shotgun target-enriched metagenomic sequencing resistome gene count matrix

**Data S6. (Supplementary_datafile_6_resistome.data.file.annotation.csv)**

Shotgun target-enriched metagenomic sequencing resistome gene annotation matrix

**Data S7. (Supplementary_datafile_7_mobilome.data.file.counts.csv)**

Shotgun target-enriched metagenomic sequencing mobilome gene count matrix

**Data S8. (Supplementary_datafile_8_mobilome.data.file.annotation.csv)**

Shotgun target-enriched metagenomic sequencing mobilome gene annotation matrix

**Data S9. (Supplementary_datafile_9_StrainPhlAn_analysis.counts.csv)**

Relative abundance analysis results of strains identified by StrainPhlAn following shotgun target-enriched metagenomic sequencing

**Data S10. (Supplementary_datafile_10_DESeq_results_resistome.csv)**

Shotgun target-enriched metagenomic resistome sequencing differential abundance analysis results

**Data S11. (Supplementary_datafile_11_DESeq_results_mobilome.csv)**

Shotgun target-enriched metagenomic mobilome sequencing differential abundance analysis results

**Data S12. (Supplementary_datafile_12_MAG_metadata.csv)**

Metagenomic assembled genome phylogenetic analysis and metadata

**SUPPLEMENTARY REFERENCES**

[1. A. L. Byrd, Y. Belkaid, J. A. Segre, The human skin microbiome. *Nat Rev Microbiol* **16**, 143–155 (2018).](https://www.zotero.org/google-docs/?6vB8IK)

[2. Human Microbiome Project Consortium, Structure, function and diversity of the healthy human microbiome. *Nature* **486**, 207–214 (2012).](https://www.zotero.org/google-docs/?6vB8IK)

[3. D. M. Gohl, *et al.*, Systematic improvement of amplicon marker gene methods for increased accuracy in microbiome studies. *Nat Biotechnol* **34**, 942–949 (2016).](https://www.zotero.org/google-docs/?6vB8IK)

[4. N. R. Noyes, *et al.*, Enrichment allows identification of diverse, rare elements in metagenomic resistome-virulome sequencing. *Microbiome* **5**, 142 (2017).](https://www.zotero.org/google-docs/?6vB8IK)

[5. E. Doster, *et al.*, MEGARes 2.0: a database for classification of antimicrobial drug, biocide and metal resistance determinants in metagenomic sequence data. *Nucleic Acids Res* **48**, D561–D569 (2020).](https://www.zotero.org/google-docs/?6vB8IK)

[6. M. Liu, *et al.*, ICEberg 2.0: an updated database of bacterial integrative and conjugative elements. *Nucleic Acids Res.* **47**, D660–D665 (2019).](https://www.zotero.org/google-docs/?6vB8IK)

[7. A. Carattoli, *et al.*, In Silico Detection and Typing of Plasmids using PlasmidFinder and Plasmid Multilocus Sequence Typing. *Antimicrob Agents Chemother* **58**, 3895–3903 (2014).](https://www.zotero.org/google-docs/?6vB8IK)

[8. H. C. Metsky, *et al.*, Capturing sequence diversity in metagenomes with comprehensive and scalable probe design. *Nature Biotechnology* **37**, 160–168 (2019).](https://www.zotero.org/google-docs/?6vB8IK)

[9. M. Martin, Cutadapt removes adapter sequences from high-throughput sequencing reads. *EMBnet.journal* **17**, 10–12 (2011).](https://www.zotero.org/google-docs/?6vB8IK)

[10. B. J. Callahan, *et al.*, DADA2: High-resolution sample inference from Illumina amplicon data. *Nature Methods* **13**, 581–583 (2016).](https://www.zotero.org/google-docs/?6vB8IK)

[11. C. Quast, *et al.*, The SILVA ribosomal RNA gene database project: improved data processing and web-based tools. *Nucleic Acids Research* **41**, D590–D596 (2013).](https://www.zotero.org/google-docs/?6vB8IK)

[12. N. M. Davis, D. M. Proctor, S. P. Holmes, D. A. Relman, B. J. Callahan, Simple statistical identification and removal of contaminant sequences in marker-gene and metagenomics data. *Microbiome* **6**, 226 (2018).](https://www.zotero.org/google-docs/?6vB8IK)

[13. A. M. Bolger, M. Lohse, B. Usadel, Trimmomatic: a flexible trimmer for Illumina sequence data. *Bioinformatics* **30**, 2114–2120 (2014).](https://www.zotero.org/google-docs/?6vB8IK)

[14. H. Li, Aligning sequence reads, clone sequences and assembly contigs with BWA-MEM (2013) https:/doi.org/10.48550/arXiv.1303.3997 (March 27, 2023).](https://www.zotero.org/google-docs/?6vB8IK)

[15. H. Li, *et al.*, The Sequence Alignment/Map format and SAMtools. *Bioinformatics* **25**, 2078–2079 (2009).](https://www.zotero.org/google-docs/?6vB8IK)

[16. I. B. Slizovskiy, K. Mukherjee, C. J. Dean, C. Boucher, N. R. Noyes, Mobilization of antibiotic resistance: Are current approaches for colocalizing resistomes and mobilomes useful? *Front. Microbiol.* **11** (2020).](https://www.zotero.org/google-docs/?6vB8IK)

[17. R. Leplae, G. Lima-Mendez, A. Toussaint, ACLAME: a CLAssification of Mobile genetic Elements, update 2010. *Nucleic Acids Res.* **38**, D57-61 (2010).](https://www.zotero.org/google-docs/?6vB8IK)

[18. P. Siguier, J. Perochon, L. Lestrade, J. Mahillon, M. Chandler, ISfinder: the reference centre for bacterial insertion sequences. *Nucleic Acids Res.* **34**, D32-36 (2006).](https://www.zotero.org/google-docs/?6vB8IK)

[19. P. Kichenaradja, P. Siguier, J. Pérochon, M. Chandler, ISbrowser: an extension of ISfinder for visualizing insertion sequences in prokaryotic genomes. *Nucleic Acids Research* **38**, D62–D68 (2010).](https://www.zotero.org/google-docs/?6vB8IK)

[20. R. Apweiler, *et al.*, UniProt: the Universal Protein knowledgebase. *Nucleic Acids Res* **32**, D115-119 (2004).](https://www.zotero.org/google-docs/?6vB8IK)

[21. E. Doster, *et al.*, Investigating Effects of Tulathromycin Metaphylaxis on the Fecal Resistome and Microbiome of Commercial Feedlot Cattle Early in the Feeding Period. *Front Microbiol* **9** (2018).](https://www.zotero.org/google-docs/?6vB8IK)

[22. A.-N. Zhang, *et al.*, An omics-based framework for assessing the health risk of antimicrobial resistance genes. *Nat Commun* **12**, 4765 (2021).](https://www.zotero.org/google-docs/?6vB8IK)

[23. T. K. Nielsen, P. D. Browne, L. H. Hansen, Antibiotic resistance genes are differentially mobilized according to resistance mechanism. *GigaScience* **11**, giac072 (2022).](https://www.zotero.org/google-docs/?6vB8IK)

[24. D. Bates, M. Mächler, B. Bolker, S. Walker, Fitting Linear Mixed-Effects Models Using lme4. *Journal of Statistical Software* **67**, 1–48 (2015).](https://www.zotero.org/google-docs/?6vB8IK)

[25. R. Lenth, J. Love, M. Herve, emmeans: Estimated Marginal Means, aka Least-Squares Means (2018) (April 26, 2023).](https://www.zotero.org/google-docs/?6vB8IK)

[26. S. Xu, *et al.*, MicrobiotaProcess: A comprehensive R package for deep mining microbiome. *The Innovation* **4**, 100388 (2023).](https://www.zotero.org/google-docs/?6vB8IK)

[27. T. P. Quinn, *et al.*, A field guide for the compositional analysis of any-omics data. *Gigascience* **8**, giz107 (2019).](https://www.zotero.org/google-docs/?6vB8IK)

[28. J. Palarea-Albaladejo, J. A. Martín-Fernández, zCompositions — R package for multivariate imputation of left-censored data under a compositional approach. *Chemometrics and Intelligent Laboratory Systems* **143**, 85–96 (2015).](https://www.zotero.org/google-docs/?6vB8IK)

[29. J. Oksanen, Vegan: an introduction to ordination. 12 (2019).](https://www.zotero.org/google-docs/?6vB8IK)

[30. M. I. Love, W. Huber, S. Anders, Moderated estimation of fold change and dispersion for RNA-seq data with DESeq2. *Genome Biology* **15**, 550 (2014).](https://www.zotero.org/google-docs/?6vB8IK)

[31. M. Stephens, False discovery rates: a new deal. *Biostatistics* **18**, 275–294 (2017).](https://www.zotero.org/google-docs/?6vB8IK)

[32. Z. D. Kurtz, *et al.*, Sparse and Compositionally Robust Inference of Microbial Ecological Networks. *PLOS Computational Biology* **11**, e1004226 (2015).](https://www.zotero.org/google-docs/?6vB8IK)

[33. N. Meinshausen, P. Bühlmann, High dimensional graphs and variable selection with the LASSO. *The Annals of Statistics* **34** (2006).](https://www.zotero.org/google-docs/?6vB8IK)

[34. C. L. Müller, R. Bonneau, Z. Kurtz, Generalized Stability Approach for Regularized Graphical Models (2016) https:/doi.org/10.48550/arXiv.1605.07072 (April 3, 2023).](https://www.zotero.org/google-docs/?6vB8IK)

[35. G. Csardi, T. Nepusz, The Igraph Software Package for Complex Network Research. *InterJournal* **Complex Systems**, 1695 (2005).](https://www.zotero.org/google-docs/?6vB8IK)

[36. J. Reichardt, S. Bornholdt, Statistical Mechanics of Community Detection. *Phys. Rev. E* **74**, 016110 (2006).](https://www.zotero.org/google-docs/?6vB8IK)

[37. M. E. J. Newman, M. Girvan, Finding and evaluating community structure in networks. *Phys. Rev. E* **69**, 026113 (2004).](https://www.zotero.org/google-docs/?6vB8IK)

[38. F. Beghini, *et al.*, Integrating taxonomic, functional, and strain-level profiling of diverse microbial communities with bioBakery 3. *eLife* (2021) https:/doi.org/10.7554/eLife.65088 (April 4, 2023).](https://www.zotero.org/google-docs/?6vB8IK)

[39. B. Langmead, S. L. Salzberg, Fast gapped-read alignment with Bowtie 2. *Nat Methods* **9**, 357–359 (2012).](https://www.zotero.org/google-docs/?6vB8IK)

[40. D. Li, C.-M. Liu, R. Luo, K. Sadakane, T.-W. Lam, MEGAHIT: an ultra-fast single-node solution for large and complex metagenomics assembly via succinct de Bruijn graph. *Bioinformatics* **31**, 1674–1676 (2015).](https://www.zotero.org/google-docs/?6vB8IK)

[41. D. D. Kang, *et al.*, MetaBAT 2: an adaptive binning algorithm for robust and efficient genome reconstruction from metagenome assemblies. *PeerJ* **7**, e7359 (2019).](https://www.zotero.org/google-docs/?6vB8IK)

[42. M. R. Olm, C. T. Brown, B. Brooks, J. F. Banfield, dRep: a tool for fast and accurate genomic comparisons that enables improved genome recovery from metagenomes through de-replication. *ISME J* **11**, 2864–2868 (2017).](https://www.zotero.org/google-docs/?6vB8IK)

[43. D. H. Parks, M. Imelfort, C. T. Skennerton, P. Hugenholtz, G. W. Tyson, CheckM: assessing the quality of microbial genomes recovered from isolates, single cells, and metagenomes. *Genome Res* **25**, 1043–1055 (2015).](https://www.zotero.org/google-docs/?6vB8IK)

[44. P.-A. Chaumeil, A. J. Mussig, P. Hugenholtz, D. H. Parks, GTDB-Tk v2: memory friendly classification with the genome taxonomy database. *Bioinformatics* **38**, 5315–5316 (2022).](https://www.zotero.org/google-docs/?6vB8IK)

[45. L. Glendinning, R. D. Stewart, M. J. Pallen, K. A. Watson, M. Watson, Assembly of hundreds of novel bacterial genomes from the chicken caecum. *Genome Biology* **21**, 34 (2020).](https://www.zotero.org/google-docs/?6vB8IK)

[46. , IQ-TREE 2: New Models and Efficient Methods for Phylogenetic Inference in the Genomic Era | Molecular Biology and Evolution | Oxford Academic (March 27, 2023).](https://www.zotero.org/google-docs/?6vB8IK)

[47. , Interactive Tree Of Life (iTOL) v5: an online tool for phylogenetic tree display and annotation | Nucleic Acids Research | Oxford Academic (March 27, 2023).](https://www.zotero.org/google-docs/?6vB8IK)

[48. A. A. Ross, K. M. Müller, J. S. Weese, J. D. Neufeld, Comprehensive skin microbiome analysis reveals the uniqueness of human skin and evidence for phylosymbiosis within the class Mammalia. *Proc Natl Acad Sci U S A* **115**, E5786–E5795 (2018).](https://www.zotero.org/google-docs/?6vB8IK)
