# Supplementary material for: Reducing skin microbiome exposure impacts through swine farm biosecurity
Source: Gigascience. 2025 Jul 26;14:giaf062. doi: 10.1093/gigascience/giaf062 (PMC12810053; doi:10.1093/gigascience/giaf062)
Supplement: giaf062_GIGA-D-24-00356_original_submission [file giaf062_giga-d-24-00356_original_submission.pdf]

GigaScience

Reducing Skin Microbiome Exposure Impacts Through Swine Farm Biosecurity

--Manuscript Draft--

**Manuscript Number:**	GIGA-D-24-00356
**Full Title:**	Reducing Skin Microbiome Exposure Impacts Through Swine Farm Biosecurity
**Article Type:**	Research
**Funding Information:**	Division of Intramural Research, National Institute of Allergy and Infectious Diseases (1R01AI141810-01) — Dr Christina Boucher<br>National Institute for Occupational Safety and Health (T42 OH008434) — Dr Ilya Slizovskiy
**Abstract:**	Livestock work is unique due to worker exposure to animal-associated microbiomes within the workplace. Swine workers are a unique cohort within the U.S. livestock labor force, as they have direct daily contact with pigs and undertake mandatory biosecurity interventions. However, investigating this occupational cohort is challenging, particularly within tightly regulated commercial swine operations. Thus, little is known about the impacts of animal exposure and biosecurity protocols on the swine worker microbiome. We obtained unique samples from U.S. swine workers, using a longitudinal study design to investigate temporal microbiome dynamics. We observed a significant increase in bacterial DNA load on worker skin during the workday, with concurrent changes in the composition and abundance of microbial taxa, resistance genes and mobile genetic elements. However, compulsory biosecurity showering at the end of the workday reverted the skin microbiome and resistome to the baseline state. These novel results from a human cohort demonstrate that existing biosecurity practices ameliorate work-associated microbiome impacts.
**Corresponding Author:**	Noelle Noyes<br>University of Minnesota College of Veterinary Medicine: University of Minnesota Twin Cities College of Veterinary Medicine<br>UNITED STATES
**Corresponding Author Secondary Information:**	
**Corresponding Author's Institution:**	University of Minnesota College of Veterinary Medicine: University of Minnesota Twin Cities College of Veterinary Medicine
**Corresponding Author's Secondary Institution:**	
**First Author:**	Ilya Slizovskiy, DVM, PhD, MPH
**First Author Secondary Information:**	
**Order of Authors:**	Ilya Slizovskiy, DVM, PhD, MPH<br>Tara N Gaire, DVM, PhD<br>Peter M Ferm, M.Sc.<br>Carissa A Odland, DVM, M.Sc.<br>Scott A Dee, DVM, PhD<br>Joel Nerem, DVM, M.Sc.<br>Jonathan E Bravo, M.Sc.<br>Christina Boucher, PhD<br>Noelle R Noyes, DVM, PhD
**Order of Authors Secondary Information:**	
**Additional Information:**	

Powered by Editorial Manager® and ProduXion Manager® from Aries Systems Corporation

Question	Response
Are you submitting this manuscript to a special series or article collection?	No
**Experimental design and statistics**<br><br>Full details of the experimental design and statistical methods used should be given in the Methods section, as detailed in our Minimum Standards Reporting Checklist. Information essential to interpreting the data presented should be made available in the figure legends.<br><br>Have you included all the information requested in your manuscript?	Yes
**Resources**<br><br>A description of all resources used, including antibodies, cell lines, animals and software tools, with enough information to allow them to be uniquely identified, should be included in the Methods section. Authors are strongly encouraged to cite Research Resource Identifiers (RRIDs) for antibodies, model organisms and tools, where possible.<br><br>Have you included the information requested as detailed in our Minimum Standards Reporting Checklist?	Yes
**Availability of data and materials**<br><br>All datasets and code on which the conclusions of the paper rely must be either included in your submission or deposited in publicly available repositories (where available and ethically appropriate), referencing such data using a unique identifier in the references and in the "Availability of Data and Materials" section of your manuscript.	Yes

Powered by Editorial Manager® and ProduXion Manager® from Aries Systems Corporation

Have you have met the above requirement as detailed in our Minimum Standards Reporting Checklist?	

Powered by Editorial Manager® and ProduXion Manager® from Aries Systems Corporation

Manuscript
Click here to access/download;Manuscript;LaborOME_Project_Submission_G

FRONT MATTER

Title

Reducing Skin Microbiome Exposure Impacts Through Swine Farm Biosecurity

Authors

Ilya B. Slizovskiy[1,2,3], Tara N. Gaire[1], Peter M. Ferm[1], Carissa A. Odland[4], Scott A. Dee[5], Joel Nerem[5], Jonathan E. Bravo[6], Christina Boucher[6], Noelle R. Noyes*[1]

Affiliations

[1]Food-Centric Corridor, Infectious Disease Laboratory, Department of Veterinary Population Medicine, College of Veterinary Medicine, University of Minnesota, St. Paul, MN, USA.
[2]Department of Veterinary Clinical Sciences, College of Veterinary Medicine, Purdue University, West Lafayette, IN, USA.
[3]Purdue Applied Microbiome Sciences Program, Purdue University, West Lafayette, IN, USA.
[4]Pipestone Veterinary Services, Pipestone, MN, USA.
[5]Pipestone Applied Research, Pipestone, MN, US.
[6]Department of Computer and Information Science and Engineering, Herbert Wertheim College of Engineering, University of Florida, Gainesville, FL, USA.

To whom correspondence shall be addressed:

Dr. Noelle R. Noyes
nnoyes@umn.edu
385D AnSci/VM
1988 Fitch Avenue
St. Paul, MN 55108
United States

ABSTRACT

Livestock work is unique due to worker exposure to animal-associated microbiomes within the workplace. Swine workers are a unique cohort within the U.S. livestock labor force, as they have direct daily contact with pigs and undertake mandatory biosecurity interventions. However, investigating this occupational cohort is challenging, particularly within tightly regulated commercial swine operations. Thus, little is known about the impacts of animal exposure and biosecurity protocols on the swine worker microbiome. We obtained unique samples from U.S. swine workers, using a longitudinal study design to investigate temporal microbiome dynamics. We observed a significant increase in bacterial DNA load on worker skin during the workday, with concurrent changes in the composition and abundance of microbial taxa, resistance genes and mobile genetic elements. However, compulsory biosecurity showering at the end of the workday reverted the skin microbiome and resistome to the baseline state. These novel results from a human cohort demonstrate that existing biosecurity practices ameliorate work-associated microbiome impacts.

Summary statement

Showering as a biosecurity procedure halts the accumulation of farm-associated bacteria and antimicrobial resistance on worker skin, and can be useful in dampening microbial transmission out of livestock production facilities.

MAIN TEXT

Occupational exposures can significantly influence the microbiomes of workers, and in some cases have been linked to health outcomes(1, 2). People working with animals encounter a unique workplace microbiome with frequent exposure to animal microbiomes, either through direct contact or indirect exposures. The impact of animal exposure on human microbiomes has been demonstrated across several settings including research facilities(3, 4) and livestock farms(5), as well as within homes(6). For example, dairy and swine workers have more diverse oral and nasal bacterial taxa than non-livestock workers(7). Additionally, the skin microbiome of livestock workers harbors a higher relative abundance of Proteobacteria and lower relative abundance of Actinobacteria and Bacteroidetes compared to people with non-livestock occupations(8). Livestock-associated bacteria and their antimicrobial resistance genes (ARGs) have been documented in agricultural worker cohorts, including farmers(9–11), veterinarians(12, 13) , and abattoir workers(10, 14) ; and short-term visitation to swine farms has been linked to an enrichment in farm-associated bacteria and ARGs in the human gut(5). These findings have been attributed to livestock exposure, but few studies have actually tracked daily animal exposure and on-site worker behavior, particularly on commercial farms. Such studies require careful consideration of workplace habits and exposures, and sampling must occur within the constraints of commercial livestock production. Given these challenges, detailed studies of livestock worker microbiomes are uncommon, and the specific influence of occupational exposures on livestock worker microbiomes remains poorly understood.

In the United States, swine workers are a unique cohort within the livestock labor force. Their job tasks involve intensive one-on-one animal handling, working within enclosed and climatically regulated facilities, with work duration ranging 48–54 hours per week(15, 16). These working conditions contrast markedly with other production systems; beef cattle and poultry are rarely handled by workers, and cattle work tends to occur in open-air facilities. Moreover, most workers

in North American swine farms adhere to strict biosecurity measures to control pathogen transmission between and within farms(17, 18), including showering into and out of the farm, which may reduce transfer of microbes and ARGs between swine farms and the general public. However, the impact of mandatory showering on the likelihood of worker-mediated farm-to-community transmission is unknown.

We report on a longitudinal investigation of the swine worker microbiome-resistome on a commercial U.S. swine facility, with sampling occurring as part of a normal workday that included showering as a mandatory biosecurity intervention (**Figure 1**). We observed a significant increase in bacterial DNA load on worker skin during the workday, with concurrent changes in the composition and abundance of specific microbial taxa, ARGs, and mobile genetic elements (MGEs) that could harbor ARGs. We further observed that compulsory showering at the end of the workday reverted the skin microbiome and resistome to a baseline state, which differs from previous results that did not include showering in the study design(5) . These results suggest that occupational work in swine facilities can significantly impact workers' skin microbiomes, but that these impacts can be transient if biosecurity interventions are implemented. The relevancy of these findings for short- and long-term swine worker health requires further study. However, the observation that showering may dampen daily microbiome impacts have important public health implications, as it demonstrates that biosecurity protocols could be leveraged to minimize microbial transmission from farms to general communities.

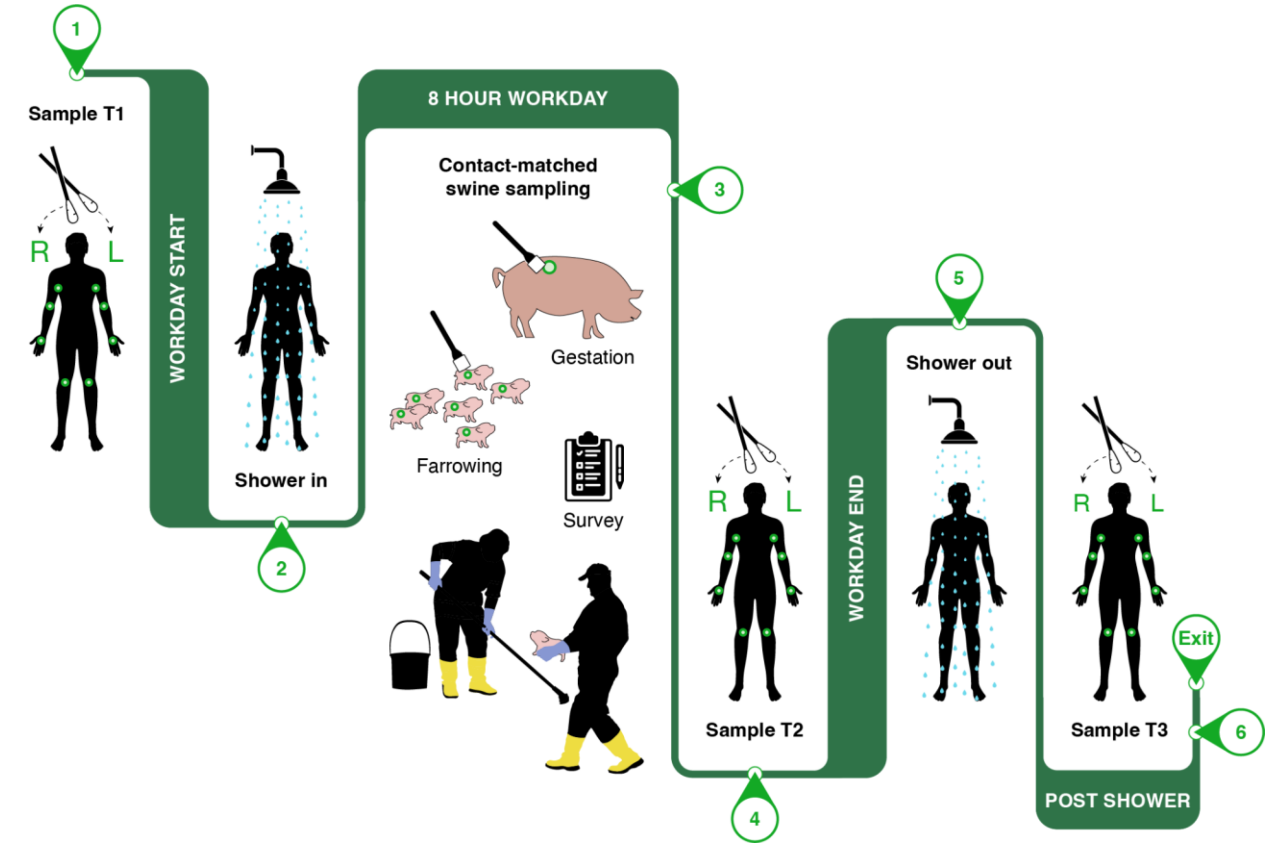

Figure 1. Study overview. Farm workers from a commercial farrow-to-wean operation in the Midwestern United States were voluntarily enrolled into a single longitudinal microbiome sampling campaign during a typical 8-hour workday shift. For each worker, swab kits were used to self-collect samples from the epidermis in a standardized fashion by passing each swab across four body sites, achieving a single composite skin sample for the left and right body representing microbiomes from the manus, interdigital space, antecubital fossa, popliteal fossa, and axilla. Workers were asked to perform the first self-collection ('Sample **T1**') prior to entry into the swine facility (**1**). Workers underwent mandatory showering prior to entry into the animal holding areas (**2**). During the day shift, workers were observed handling animals or working in specific animal pens, and dorsal skin swabs (from withers to tail-base) were taken from contact-matched animals on a pooled multi-pen level. Additionally, a 15-minute questionnaire was administered to collect biometric, health, lifestyle, and occupational task performance information from each worker (**3**). In a similar fashion, self-collected skin samples were taken immediately upon conclusion of the workday ('Sample **T2**') (**4**). Workers underwent mandatory showering procedures immediately after exiting the animal holding areas (**5**) and a third self-collection of samples was performed ('Sample **T3**') after showering and immediately prior to exiting the farm facility (**6**).

RESULTS

Individual skin microbiomes experience dramatic yet transient shifts during on-farm work with swine

16S rRNA sequencing and analysis was performed on 40 skin samples collected longitudinally from 10 healthy and predominantly male swine workers and contact-matched swine (SI Appendix SI Methods, **Supplementary note 1**, **Supplementary file 1**). The skin microbiome composition underwent significant shifts in the course of a single 8-hour workday (ANOSIM $p < 0.001$;

R=45.9%, adjusted PERMANOVA $p< 0.001$, **Figure 2a**). Specifically, samples taken at the end of work but prior to showering (T2) had a significantly different composition than samples taken at the beginning of work (T1) (pairwise R^2=15.5%; FDR adjusted p=0.002); and at the end of the workday following showering (T3) the microbiome underwent yet another shift relative to T2 (pairwise R^2=14.0%; FDR adjusted p=0.002). However, the microbiome at T3 was not significantly different than at T1, suggesting a reversion of the skin microbiome during showering (pairwise R^2=5.7%; FDR adjusted p=0.25). These shifts corresponded with changes in skin-borne bacterial load as quantified using 16S gene concentration (copies/µl, **Figure 2b**). Specifically, 16S gene concentration increased by ~200-fold from T1 to T2, but then decreased back to baseline levels at T3 (Type III ANOVA $p< 0.0001$ with Tukey's *post hoc* analysis and adjustment for FDR). There were no significant differences in average sequencing depth, sequencing quality and taxonomic classification rates between the three collection time points, suggesting that these technical factors did not significantly bias comparisons across collection phases (**Supplementary note 2**, **Supplementary figures 1, 2**).

We next assessed associations between crude swine exposure rates and 16S gene concentration, i.e., bacterial load (SI Appendix, SI Methods). There was large variation in estimated hourly swine exposure rates (**Supplementary table 1**), and the hourly exposure density was found to be inversely correlated with bacterial load (Estimate[SE]= -1.23[0.44], *glm* p=0.01, **Figure 2c**). This relationship persisted at T2 and T3 (**Figure 2c**). Though more granular and systematic exposure assessments are needed, these patterns suggest that differences in swine exposure density may be a proxy for different workday tasks that ultimately dictate levels of microbial biomass acquisition. For example, tasks such as feeding, health-checks, and decontamination require walking through swine holding rooms, but involve very little direct interfacing with animals and their byproducts; such tasks would be classified as "high density", but in reality there may be less opportunity for

direct acquisition of swine-related microbes. In contrast, activities such as vaccinating and obstetrical management require prolonged contact with individual pigs, but not necessarily moving through multiple swine holding rooms; thus, such tasks may have lower density but more opportunities for acquisition of swine-sourced microbes through direct contact.

A total of 9,358 unique amplicon sequence variants (ASVs) representing ~600 distinct genera were recovered across all worker skin samples (**Supplemental figure 2, Supplementary datafile 2,3**). The ASV diversity and the dominant phyla were consistent with findings from previous microbiome studies of human skin(19–21). The relative abundances of dominant phyla remained largely similar across T1–T3, with the exception of Cyanobacteria, which were more abundant on skin at T2 versus T1 and T3 (**Supplemental figure 3a-b**). Phylum-level richness and evenness of the worker skin microbiome remained unchanged throughout the course of the day, and was significantly lower than in contact-matched swine skin samples (Type III ANOVA adjusted $p < 0.01$, **Supplemental figure 4a-b**). Similarly, at the genus level, alpha diversity remained stable between T1 and T2, though after showering Shannon's diversity decreased significantly compared to T2 samples (Type III ANOVA adjusted $p < 0.05$, **Supplemental figure 4d**).

More than 400 genera were detected in at least one sample from each of the pairwise collection phases under comparison (T1 vs. T2 [n=464], T2 vs. T3 [n= 401], and T1 vs. T3 [n=463]), and fewer than 5% of these genera exhibited significant changes in relative abundance over the three timepoints (**Supplementary datafile 4**, **Supplementary figure 5**). The relative abundances of *Neisseria*, *Haemophilus*, *Leuconostoc*, *Actinomyces*, and *Pantoea* were significantly higher in T2 versus T1 samples. These genera inhabit mammalian mucous membranes, including in the oral cavity and digestive tracts. Genera with significantly higher relative abundance in T3 compared to T1 samples included many environmental taxa such as *Agrococcus*, *Skermanella*, and

Exiguobacterium. *Prevotella* exhibited the largest fold-change increase in relative abundance in T3 versus T1 samples, and were also found at a significantly greater relative abundance at T2 vs T1 (**Supplementary datafile 4**). *Prevotella* is a keystone member of the dominant enterotype of growing piglets in farrowing units(22, 23), and swine have been a proposed source of *Prevotellaceae* dominance in swine worker fecal microbiota(24).

The skin microbiome becomes unstructured and dominated by enteric and environmental microbes during on-farm work

Inferred association networks were explored using a compositional modeling approach to describe the topology and connectivity of microbial constituents in worker skin microbiomes across collection phases and in comparison to contact-matched animals (SI Appendix, SI Methods). Networks were constructed using standard cutoffs, and ASVs with >100 counts in every sample were included, which represented 2-4% of all ASVs used as input into network generation, depending on the collection phase. The resulting networks (one per collection phase) were each composed of a singular interconnected component, with the most connected network being T1 and the most sparse T2 (**Figure 2d**).

Given the significant differences in network topology across T1, T2 and T3 (**Supplementary note 4**), we further analyzed each network to identify the most dominant and interconnected genera (i.e., keystone taxa), as indicated by high eigenvector centrality and high node degree. At T1, keystone genera included commensals such as *Corynebacterium* and *Actinomyces*, well-known as ubiquitous inhabitants of human skin(25) (**Figure 2e**). The most influential genus in the T1 network was the newly described and poorly characterized genus *W5053* of the order Peptostreptococcales-Tissierellales, previously identified among medically important inhabitants

of human axillae(26). Other keystone T1 genera included *Staphylococcus* and *Anaerococcus*, however, these taxa were among the least important constituents of the network at T2, based on their low eigenvector and node degree centralities (**Figure 2e**); and were replaced by new keystone genera from the Clostridia and Clostridia-like class, including the previously uncultured *Ruminococcaceae* UCG-014, *Terrisporobacter*, *Romboutsia*, and *Cellulosilyticum*. These taxa are characteristic of mammalian intestinal flora. After showering, keystone genera were more heterogenous, comprising a mixture of taxa predominantly associated with the mammalian gastrointestinal tract (e.g., *Romboutsia*, *Rothia*, *Gemella*); with environmental samples (e.g., *Mucilaginibacter* and *Massilia*); and with extremophilic function (e.g., *Thermoactinomyces*).

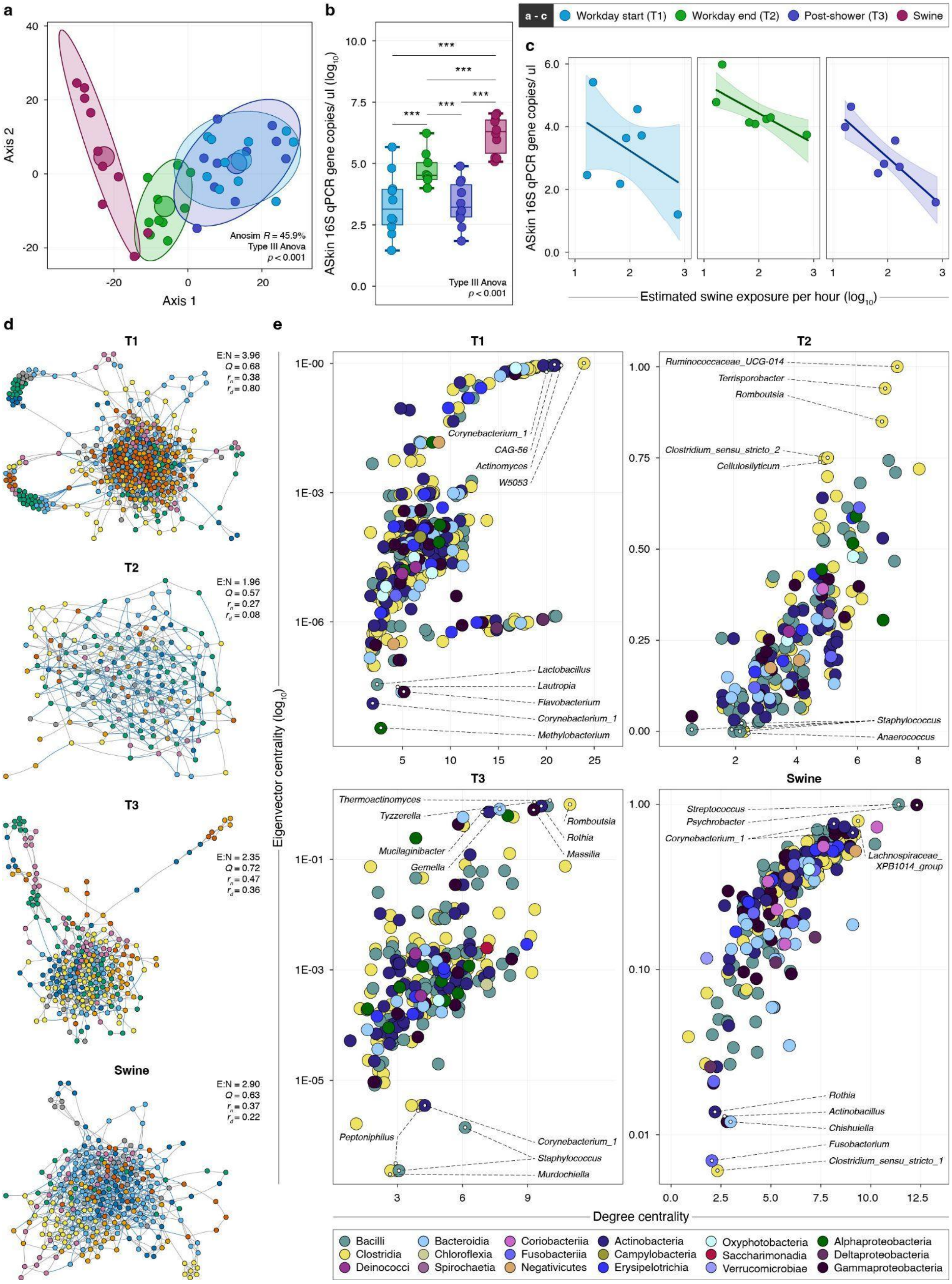

Figure 2. Changes in skin microbial load, microbiome composition, and community structure. **a.** Genus-level worker skin microbiome □-diversity across collection phases (T1–T3) and contact-matched swine skin samples using principal component ordination of robust Aitchison compositions. Within-group centroids and 95% confidence intervals are depicted with a large circle and shaded ellipsoids, respectively. **b.** Log_{10}-normalized 16S rRNA qPCR copy number /

µl (y-axis), stratified by collection phase (x-axis). *** indicates statistical significance (p<0.001) of pairwise comparisons based on a linear regression model with Tukey's adjustment for multiple comparisons. **c.** General linear analysis of the log_{10}-normalized 16S rRNA qPCR copy number / µl (y-axis) and log_{10}-normalized hourly exposure to swine (x-axis) based on workers' estimates from daily task assignments indicates a negative correlation (p= 0.01) across all collection phases. Shaded areas represent the 95% confidence interval around the linear trendline. **d.** Worker skin microbiome networks across workday collection phases and for contact-matched swine were inferred from inverse covariance estimation for compositions based on centered-log ratios of subsetted ASV counts containing >100 counts per ASV per sample. Inferred networks consist of nodes representing ASVs colored by shared subcommunity membership. Edges between nodes represent a significant predicted positive (blue) or negative (gray) interaction. Reported topology characteristics include network connectivity based on the edge to node ratio ($E:N$), modularity (Q), subcommunity assortativity (r_n), and degree assortativity (r_d). **e**. Scatter plots of microbial constituents from the corresponding networks are displayed based on the log_{10}-normalized node eigenvector centrality (y-axis) and node degree centrality (x-axis). Taxa with the highest centrality measures (top right of the distribution) are considered to be critical connectors and major hubs in community networks, and thus putative keystone taxa. ASV-level nodes are colored based on their taxonomic classification at the Class level. Genus-level labels are displayed only for genera most likely to be keystone, i.e., >95th percentile of the plot distribution (top right) and least likely to be keystone, i.e.<5th percentile of the plot distribution (bottom left).

The worker skin resistome and mobilome shifted significantly during the workday, and differed from that of contact-matched swine

Target enrichment was used to selectively capture and amplify all potential known ARGs and MGEs within the metagenomic DNA of all samples (SI Appendix, SI Methods)(27). As with the microbiome, the resistome shifted significantly between each collection phase (ANOSIM p< 0.0001; R=37.5%, **Supplementary figure 6a**), but there were no statistically significant differences in ARG group richness or Shannon's diversity across the three collection phases (**Supplementary figure 7**). Similarly, plasmids, ICE, IS, and TE underwent significant shifts in □-diversity, with collection phase accounting for up to 44% of the variation observed within each MGE type (all ANOSIM p<0.001, **Supplementary figure 6b**). By contrast, virus and prophage composition did not differ significantly by collection phase (ANOSIM p>0.05). Plasmids, ICE, IS and TE compositions were significantly different between T1 and T2, and between T2 and T3 (all PERMANOVA p<0.001). The observed MGE compositional shifts at T2 coincided with a greater relative abundance of ICE genes and a reduced relative abundance of plasmids, including plasmidic mechanisms of replication, transcription, translation, and regulation (**Supplementary figure 6c-d**). Between T1 and T3, there were significant differences in composition of IS and TE genes (PERMANOVA p=0.048 and 0.037, respectively), but not plasmids and ICE. The worker

resistome and MGEs at all three phases were significantly different from swine at all three collection phases, with the exception of viral and prophages at T2 (**Supplementary figure 6**).

The clinically important fraction of the worker skin resistome varied throughout the workday and remained distinct from swine

We subsetted the MEGARes v2.0 database for 29 specific ARG groups previously identified as 'clinically important' (i.e., priority ARGs) (28, 29). In 41/42 enriched metagenomic samples, we detected 19 distinct priority ARG groups at gene coverage fraction >99.9%; one T2 sample did not contain any priority ARGs. These ARGs represented a low proportion of the total resistome across all worker (median[IQR]= 7.46%[8.23]) and swine samples (median[IQR]= 5.57%[4.27]), and their overall median relative abundance did not differ between workday collection phases and swine samples (Type III ANOVA $p > 0.05$).

Tetracycline (*TetM*), sulfonamide (*Sul1*), multi-drug resistance (*Vga*), and methicillin (*mecA*) genes were the most prevalent and abundant of the priority ARGs (**Figure 3)**, and strongly influenced hierarchical clustering of samples into four major groups (i.e. subclades). Subclade 1 was characterized by high *TetM* relative abundance and contained eight of the 10 swine samples and one or two worker samples from each of T1-T3. Subclade 2 contained five of the 9 T2 samples, one swine sample, one T1 and two T3 samples, and was characterized by a higher relative abundance of *Sul1*. Subclades 3 and 4 contained the majority of the T1 and T3 samples (i.e., 14/20), with subclade 3 defined by a higher abundance of *Vga* and subclade 4 containing the highest relative abundance of *mecA*.

The *mecA* gene, a methicillin resistance allele, consistently appeared in worker but rarely in swine samples (**Figure 3**). *Staphylococcus aureus* in swine has been proposed as a key source of methicillin resistant *S. aureus* (MRSA) in Danish swine workers(30), especially among workers of Danish pig herds in which historical MRSA prevalence exceeds 85%. However, recent reports suggest that *Staphylococcus* spp. are actually rare members of the porcine skin microbiome, and typically account for <1% of the overall relative abundance of all Staphylococci(31). We performed marker-based strain-level taxonomic profiling of metagenomic reads via StrainPhlAn to ascertain possible Staphylococcal sources of *mecA*. Strains of *S. epiderimidis*, *S. haemolyticus*, and *S. hominis* were the only prevalent strains identified (>75% prevalence), and no *S. aureus* strains were identified at this pre-specified prevalence level (**Supplementary datafile 9**, **Supplementary figure 8**). Further phylogenetic analysis suggested that these Staphylococci were rarely shared between workers and swine, as most strains were tightly clustered by worker ID rather than collection phase (**Supplementary figure 9**). Major coagulase negative Staphylococci (CoNS) are known carriers of *mecA*, and ~90% of U.S. *Staphylococcus epidermidis* clinical isolates in particular are methicillin resistant(32–34). Taken together, these findings suggest that worker CoNS and not *S. aureus* were likely sources of *mecA* in this study. Interestingly, no major Staphylococcal strains were detected at T2. This could be due to the increased microbial biomass in T2 samples (**Figure 2b**), which may have reduced the relative abundance of *Staphylococcus* sequences within the extracted DNA and resulting metagenomic data, effectively pushing *Staphylococcus* under the limit of detection needed for robust StrainPhlan analysis.

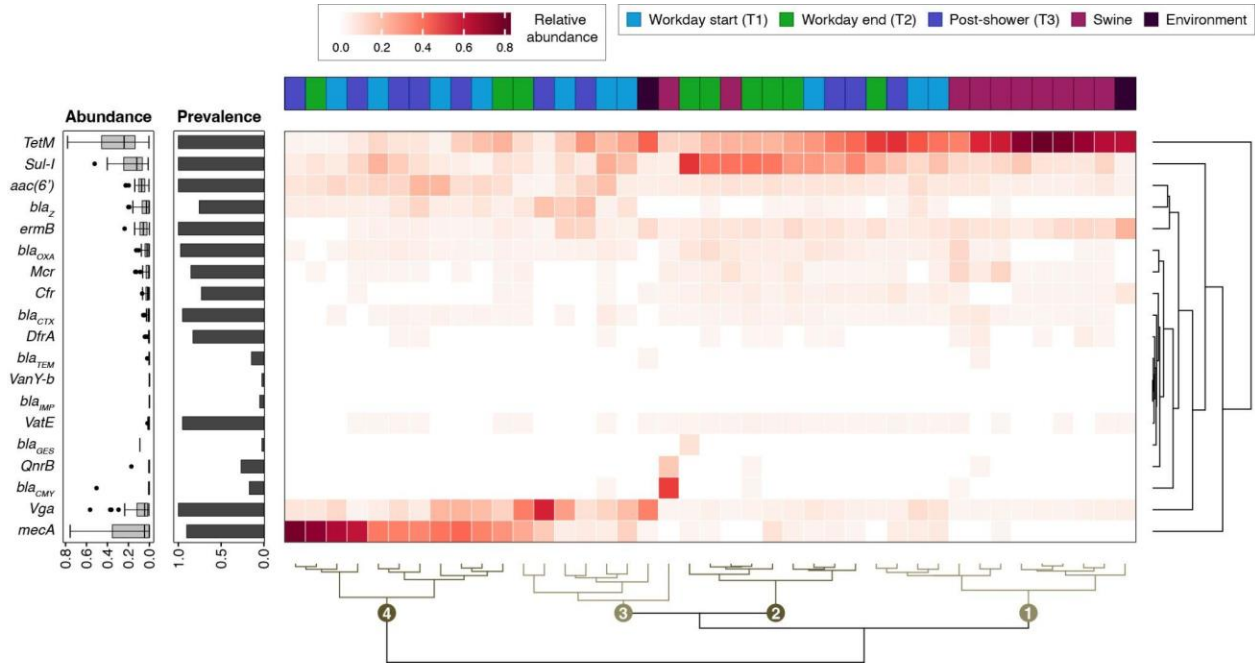

Figure 3. Occurrence of medically important ARGs on the skin of workers and swine. Unique medically important (i.e., priority) ARG alleles were identified at >99.9% alignment gene coverage across all collection phases (top ribbon annotation) and are displayed using a heatmap summarizing their sample-level relative abundance across each of the respective 19 ARG gene groups. The cladograms along the x-axis demonstrate the hierarchical clustering of samples according to their medically important resistome composition using optimal leaf sorting and euclidean distances. Four major subclades are colored and numbered. Major ARG group prevalence and median abundance across study samples are summarized via the associated barplots and boxplots along the y-axis.

Post-work showering incompletely reverses changes in resistome and mobilome gene abundance

After controlling for worker age, gender, BMI, smoking status, frequency of pork consumption, as well as host-removed sequencing depth, only 5.4%, 3.7%, and 2.7% of ARG groups exhibited significant changes in relative abundance at T1 vs. T2, T2 vs. T3, and T1 vs. T3, respectively. Between T1 and T2 collection phases, the vast majority of significantly changing ARG groups exhibited increases in abundance (i.e., 27/29 ARG groups, 93%) (**Figure 4a, Supplementary datafile 10**). Because log-fold differential abundance testing can produce false positives for low-count features, we highlighted only high-abundance ARG groups with a statistically significant change in relative abundance. For the T1 versus T2 comparison, this included ARG groups within the □-lactams (e.g., *mecA*), fosfomycins (e.g., *fosA* and *fosB*), and mupirocins (e.g., *mupA*). In contrast to the T1-T2 comparison, far fewer ARG groups experienced statistically significant

changes in relative abundance from T2 to T3 (n=18), and most of these (i.e., n=13, or 72%) decreased in relative abundance, including □-lactams (e.g., bla_{GES}), fusidic acids (e.g., *fusB*), phenicols (e.g., *cmlA*), sulfonamides (e.g., *sulIV*), MLS (e.g., *ereA*), and multi-drug or multi-compound classes (e.g., *fexA, ttgB, mexW, lmrD*). Two abundant ARG groups that exhibited significant increases in relative abundance at T2 compared to T1 also remained elevated after showering in T3, most notably *mecA*, and *norA*, the general drug and biocide efflux system of Staphylococci(35). When compared to T1, 13 ARG groups at T3 were significantly differentially abundant, and 10 of these (77%) exhibited a significant decrease in relative abundance (**Figure 4a**). For example, among the most abundant T3 ARGs, there was a significant decrease in multi-compound and fusidic acid resistance (e.g., *fexA*, *mepA*, *fusB*), and multi-drug resistance regulators and efflux systems (**Supplementary datafile 10**).

Among MGEs with significant changes in relative abundance between collection phases, ICE were most prominent (**Figure 4b**). Specifically, *ICEPaeLESB58-1*, *ICETn4371* and *ICESsu(BM407)* were more abundant in T2 versus T1 samples; the first two mobilize heavy metal resistance(36), while the latter mobilizes ARGs narrowly within *Streptococcus suis*, an emergent pathogen in humans that is considered a host-adapted swine pathobiont(36, 37). Further strain analysis confirmed presence of *S. suis* in all swine and T2 samples, as well as 7/10 samples in each of T1 and T3 (**Supplementary datafile 9**). Compared to T1, T2 samples also contained significantly higher relative abundance of replication and recombination machinery of the host-adapted *Staphylococcus epidermidis* bacteriophage (e.g., helicase loader and replication helicases and Holliday junction resolvases), as well as *IS6* sequences associated with methicillin resistance (*IS431mec*), erythromycin resistance (*IS257-1*), and transposable components of *IS6*/*IS26* and *TnAS3* involved in mobilizable resistance(37) at human-animal interface contexts.

Following showering, the most abundant ICE module *ICETn6087* was reduced in relative abundance compared to T2; however, the next 9 most-prevalent MGEs increased in relative abundance at T3 versus T2 (**Figure 4b**), notably *IS431mec* which already exhibited a significant increase from T1 to T2. Other significantly more abundant MGEs in T3 versus T2 samples included plasmids of *Staphylococcus epidermidis* (*pSepCH*, *SE_p410*), *Staphylococcus aureus* (repV: *pT181*; pgi: *pSJH901*); as well as *Staphylococcus epidermidis* and *Bacillus cereus* bacteriophages and prophages. *S. suis*-adapted *ICEPaeLESB58-1* and *ICETn4371* were significantly more abundant at T3 versus T1; while promiscuous tetracycline-associated Tn916-like ICE *ICETn6085a*, *ICETn6085b*, and *ICETn6084* were significantly less abundant in T3 compared to T1 (38) (**Supplementary datafile 11**).

Worker skin at T2 (i.e., following work with swine) had a higher MGE abundance than contact-matched swine (**Figure 4b**), dominated by mucous membrane, respiratory tract, and enterically adapted ICE. Prominent among these were Streptococcal RD2 element (10750-RD.2), *ICETn1806*, as well as *ICESauJKD6008* and *ICECTn4* known to mobilize vancomycin and tetracycline resistance in *Staphylococcus aureus,* Enterococci, and *Clostridioides difficile*(39–42). *Staphylococcus aureus* and *Escherichia coli* plasmid replicon modules were also in higher abundance in T2 worker versus swine skin (e.g., *repUS12_pUB110*, *repUS23._repA(SAP099B)_GQ900449.1*, and *IncY_1__K02380*). Additionally, worker skin contained a higher relative abundance of *Psychrobacter*-associated plasmid *pRWF101_PsycPRwf*. Psychrobacter was identified as the most dominant keystone member of the swine skin microbial community network (**Figure 2e**), and the detection of *psychrobacter*-associated MGE alleles in human samples was unexpected. Historically, *Psychrobacter* isolates have been obtained from arctic, marine, sediment, and limited terrestrial environments(43). However, recently this genus has been detected in pig slurries, manure, and swine carcass processing facilities(44, 45) and

Psychrobacter spp. have been identified as dominant microbes within the nares of workers involved in swine transport(46). We demonstrate that skin-borne *Psychrobacter* and its mobilomic components may be suitable sentinels for the monitoring of microbial exposure to swine.

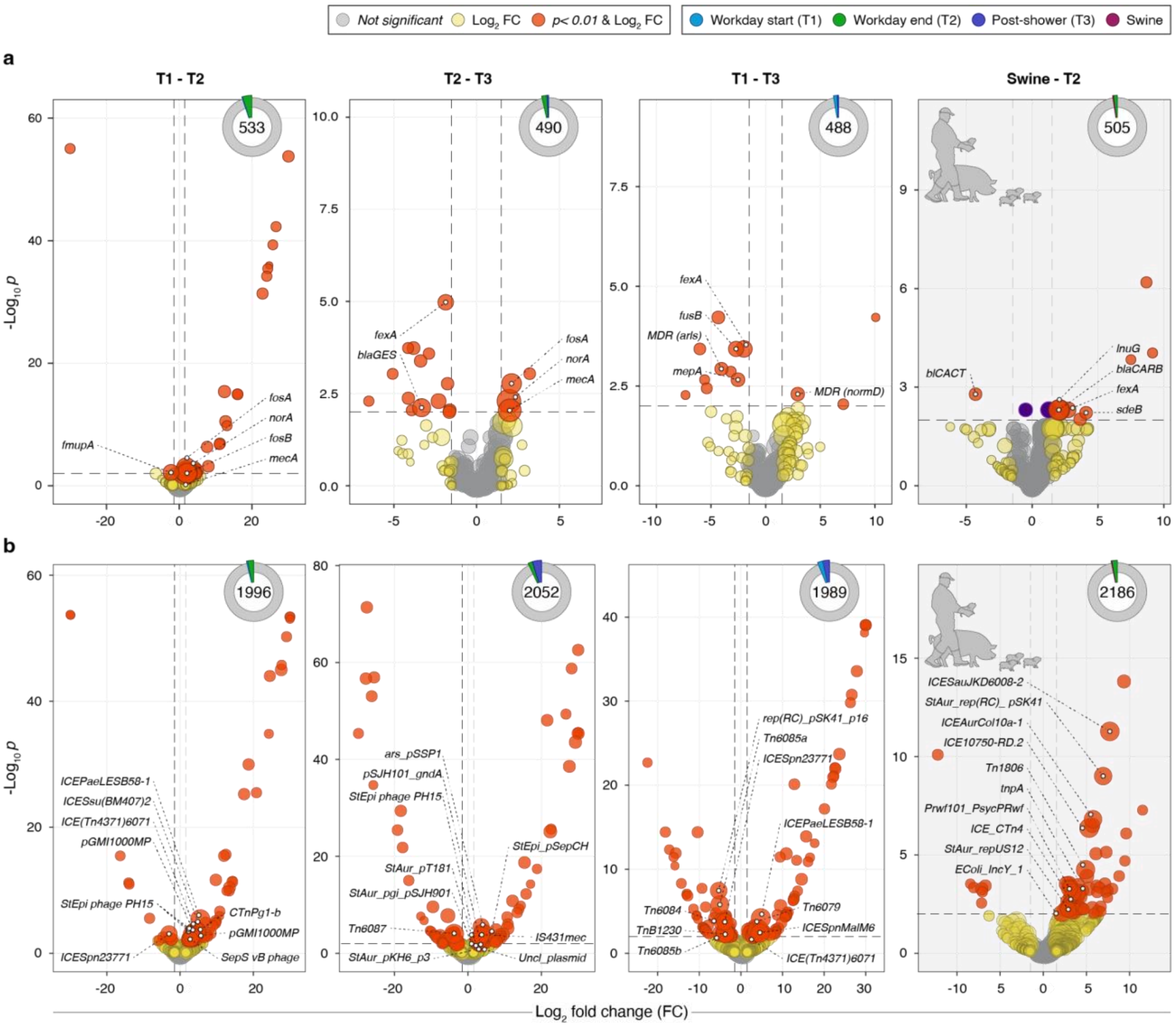

Figure 4. Differential abundance analysis across collection phases and at the interface period between workers and pigs. Volcano plots are used to visualize differential abundance of unique **a** ARG groups and **b** MGE accessions in $\log_2$-fold change (x-axis) and $-\log_{10}P$ value (y-axis) of the global worker skin resistome or mobilome between key workshift collection phases: Workday start (**T1**) vs. workday end (**T2**); Workday end (**T2**) vs. post-shower (**T3**); and Workday start (**T1**) vs. post-shower (**T3**). An additional comparison is made between workday end and swine skin samples representing the worker's contact phase with animals (gray). Features with significant shift in abundance (Wald's $p<0.01$ with FDR adjustment for ARG group and MGE accessions) are displayed above the horizontal line, while biologically significant fold-change is demarcated by vertical dashed lines at 1.5 $\log_2$-fold change. Labels are displayed for only the 5 most abundant ARG groups and 10 most abundant MGEs significantly amplified ($\log_2$-fold change >1.5 or < -1.5) at each phase comparison. For each volcano plot, an associated pie chart displays the number of unique ARG groups and MGE accessions common to each of the workshift collection phases compared, as well as the proportion of the total differentially abundant MGEs associated with each phase.

MAGs recovered from worker and swine skin samples represent putatively novel strains

High quality metagenome-assembled genomes (MAGs) were constructed via *de novo* genome assembly for all individual samples and also as co-assemblies of samples within T1, T2, T3, swine and environmental (**Figure 5a–c**). The *Firmicutes* phylum was by far predominant across all genomes (n= 139), followed by *Actinobacteriota* (n=44), *Bacteroidota* (n=17), and *Proteobacteria* (n=9). The distribution of the most abundant phyla across all MAGs was consistent with the phyla detected by 16S microbiome sequencing. A large proportion of identified MAGs had poor taxonomic representation among known GTDB MAGs, as 47 (22%) were classified as putatively novel species (i.e., <95% ANI with a known sequenced genome in GTDB), and 167 (78%) were identified as putatively novel strains (i.e., <99% ANI with a known sequenced genome in GTDB).

Approximately 60% (28/47) of all MAGs considered to be putatively novel species were recovered from swine skin samples, even though swine samples represented <25% of analyzed samples (i.e., 10/42). Swine samples also accounted for ~53% (89/167) of the MAGs identified as putatively novel strains. We detected new strains that were highly abundant in recent swine intestinal MAG catalogs(47) and that we also identified via 16S analysis in this study (**Figure 2e**), including *Psychrobacter* (*P. pasteurii*), *Streptococcus* (*S. hyovaginalis, S. pluranimalium, S. dysgalactiae*), *Corynebacterium* (*C. xerosis*, *C. variabile*, *C. glutamicum*, *C. pollutisoli*, *C. stationis*), and *Lachnospiraceae*.

Worker skin samples accounted for ~45% of the de-replicated MAGs, retrieved predominantly from co-assembly (n=62) vs. individual (n=33) approaches. Nearly 75% (71/95) of MAGs

recovered from human samples represented either novel species or strains. These novel taxa comprise 9 genera, 6 of which are known to be natural inhabitants of environmental matrices, including *Microbacterium*, *Marihabitans*, *Marmoricola*, *Chloroflexi* bacterium, *Qipengyuania*, and *Tsuneonella*. Samples representing the swine farm exposure phases (i.e., T2 and T3) accounted for 76% of the total putatively novel strains detected in worker microbiomes (T2: 28/95; T3: 26/95). Though samples from workday start (T1) accounted for the smallest proportion of all recovered human MAGs (23%), we nevertheless captured major expected cutaneous taxa as documented in previous strain-resolved MAG workflows(48, 49), including *Staphylococci* (e.g. *S. hominis, S. epidermidis, S. capitis*), *Corynebacterium* (e.g. *C. xerosis*, *C. mucifaciens, C. kefirresidentii*), *Cutibacterium* (e.g. *C. granulosum* and *C. acnes*), and *Lactobacillaceae* (e.g. *Lactobacillus amylovorus, Latilactobacillus sakei*, *Limosilactobacillus reuteri*).

In addition to the genera observed at T1, MAGs from T2 samples also included 6 genera of the Clostridial co-abundance gene group 138 (i.e., CAG-138) previously linked with critical functions for fiber degradation in the swine enteric system(50). Genera not assigned with NCBI taxonomic nomenclature from Lachnospiraceae, Butyricicoccaceae, Oscillospiraceae, and Treponemataceae were also recovered, and their identities were concordant with best-matched NCBI genomes sequenced from fecal samples of piglets <30 days old. Among MAGs recovered from T3, ~55% (20/36) included species identical to those observed in both T1 and T2 samples. However, T3 MAGs also included species that were only observed at T2 (and not at T1), including taxa typically identified in livestock such as *Streptococcus alactolyticus* known as part of the *Streptococcus bovis*/*Streptococcus equinus* complex (SBSEC), *Aerococcus urinaeequi*, as well as uncharacterized MAGs previously identified in swine fecal samples (GenBank ID: *GCA_016293975.1*, *GCA_004558825.1*, *GCA_004556755.1*)(51). Though minor human skin commensals were exclusively detected in T3 samples, such as *Lawsonella clevelandensis* and

Corynebacterium aurimucosum, genera previously isolated from environmental matrices were also exclusively recovered in T3 samples, including *Tsuneonella* sp., *Qipengyuania* sp., *Marmoricola* sp., and *Chloroflexi* bacterium UBA6265.

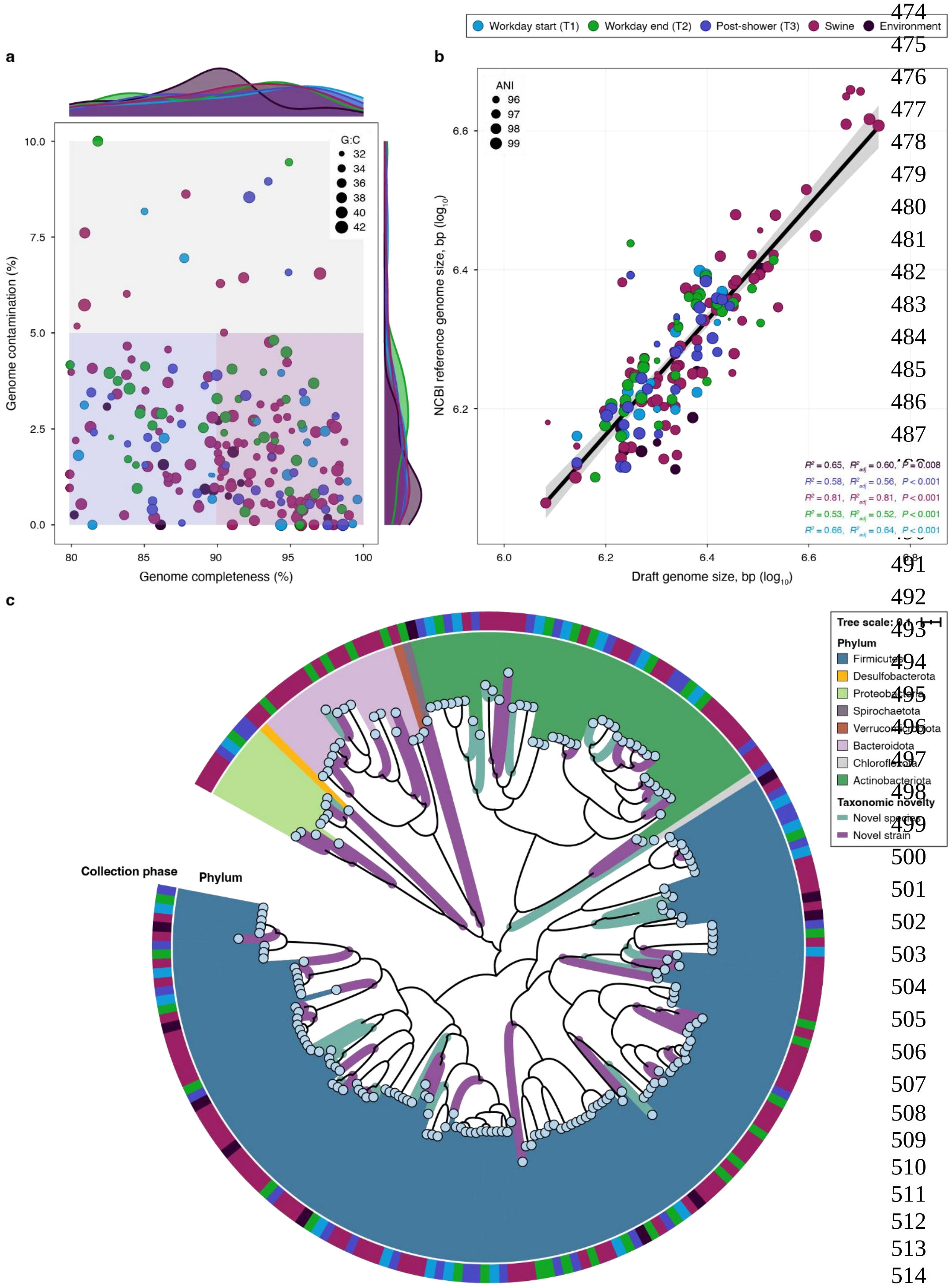

Figure 5. Taxonomic diversity and novelty of resolved metagenomic assembled genomes (MAGs) recovered from the human-swine interface and ambient environment. **a** Scatter plot of points representing individual GTDB taxonomically binned MAGs according to their estimated percent completeness (x-axis)

relative to their estimated percent contamination (y-axis). Point size is proportional to the quantified MAG GC-content. Pink, purple, and gray regions of the plot demarcate the density of ‘high quality’, ‘medium quality-low contamination’, and ‘medium quality’ MAGs retrieved across all collection phases. **b** Scatter plot of estimated MAG size is displayed based on the log_{10}-normalized nucleotide count (x-axis) and the nearest assigned NCBI reference genome size expressed in log_{10}-normalized nucleotide count (y-axis). A global regression line and 95% confidence interval (shaded region) is displayed and results of significance testing (R^2; p<0.05) using a generalized linear model are colored for MAGs recovered for each collection phase. **c** Phylogenomic tree of de-replicated and high-confidence MAGs recovered from target-enriched metagenomes across each collection phase (outer ring). The area below each leaf is colored according to the taxonomically assigned Phylum. Branches for proposed novel species (ANI <95%) are displayed in teal, and for proposed novel strains (ANI <99%) are displayed in purple.

DISCUSSION

Environmental exposure histories play a determinative role in shaping adult microbiomes, even more so than individual-level variables(52–54). Cutaneous microbiota are recognized for their remarkable stability in the face of environmental perturbation over short time scales(55–57). Despite this, we demonstrate that swine worker skin experiences a significant increase in bacterial load and a significant shift in microbiota composition during a single 8-hour workday. However, showering at the end of work seems to dampen these changes, indicating that biosecurity interventions not only reduce worker-borne swine pathogen transmission(58, 59), but also work-associated microbiome impacts. We likewise demonstrate that accumulation of ARGs on the skin can be counteracted with showering, suggesting that such biosecurity practices could be important public health measures to reduce the bidirectional flow of resistant bacteria between animal-associated workplaces and the general community (60–62). Effects of farm protocols and biosecurity have not been expressly evaluated in recent investigations of ‘shareable’ microbial features between humans, animals, and the farm environment(5, 46, 63–66). Our results suggest that process controls must not be overlooked when investigating the links between agricultural sources of AMR and public health risks.

Within-farm sources of microbes that shape worker microbiomes remain unknown, and there are no systematic, established methods for conducting microbiome-based surveillance in occupational health contexts, and particularly for commercial farm work. The striking proportion of possibly novel species that we recovered from the skin of both swine and workers suggests that

skin may be an important yet under-represented sampling target for on-farm occupational health research (**Figure 5**). We focused our study on the skin for several reasons, including ease of sampling and a high proportion of skin-associated diseases within livestock workers; additionally, the skin surface is continuously exposed to the farm environment and thus likely to serve as a competent catchment for air-borne bacteria. Indeed, recent studies have shown that farm dust and air can significantly impact worker microbiomes and resistomes, further demonstrating the need to investigate the skin-environment interface(67–69).

One major question is whether work-acquired microbes become incorporated into the cutaneous microbiome as long-term, stable members of the community. Our study design did not include long-term follow-up, and thus we could not quantify the proportion of taxa that become *de facto* colonizers following repeated workday exposures. However, we did demonstrate that showering seemed to literally wash away many of the microbes that accumulated on the skin during the workday. This may be due to the fact that microbes acquired during the workday become only weakly adherent to the skin and thus are easily washed away. The impact of showering is even more robust when one considers the heterogeneity in showering practices, as workers in this study were told to shower as they normally would at the end of their workday, including use of their own preferred soaps and other personal care products. However, it is important to note that showering did not completely eliminate newly-acquired taxa, and workers may continue to harbor microbes from enteric and environmental taxa that are characteristic of the swine farm context, as shown in our 16S rRNA and MAG results (**Figures 2** and **5**), and reported in a previous study of swine farm workers who resided in a Chinese swine farm for ~3 months(5). It is difficult to extrapolate our single-day, single-farm study to longer-term dynamics of the worker skin microbiome, and our results suggest that further work is needed to determine whether daily environmental exposures impact the long-term profile of the worker skin microbiome; and

whether such impacts carry over into the general community via human-to-human transfer. Results reported herein represent the microbiome dynamics of only 10 workers. We note, however, that his study is the first of its kind to deeply and systematically interrogate the stability of the microbiome, resistome, and mobilome of farm workers as they enter and exit a commercial U.S. swine production system. Owing to difficulties in accessing tightly controlled swine environments, such microbiome exposure assessments on U.S. farms are scarce. These results therefore offer an important foundation from which more robust microbiomic investigations can emerge.

Our findings prompt several important considerations for future assessments of workplace and environmental impacts on human microbiota. First, we highlight an urgent need to integrate classical public health exposure risk assessment with "-omics"-based workflows, which will require tailoring techniques in sampling, quantitation, and prediction across diverse environments, microbiologic burdens, and instances of human-animal-environmental interfaces. Second, occupational cohort microbiome research is challenged to establish causal links, i.e., do workplace exposures influence the human microbiome in the long-term, and do these influences lead to different health outcomes? This study is a prime example of this challenge, as we enrolled farm workers who already had months or years of on-farm exposures. Thus, the "baseline" T1 skin microbiome may have already been impacted by previous on-farm exposures, but we have no robust method for detecting these prior impacts. Similarly, it is tempting to compare our data to publicly available human skin microbiome data, but such a comparison would be inextricably biased by confounders such as the well-documented 'healthy worker' effect and other demographic variables known to impact human microbiomes. The need for robust epidemiological study design is even more pronounced in cohort-based microbiome studies because of the lability of the human microbiome. We therefore expressly avoided comparisons

across worker and non-worker cohorts and took care not to extrapolate our findings into long-term microbiome or health impacts. Instead, we focused our analysis on a time-series sampling design of the same workers before and after exposure and showering, allowing for a targeted analysis of daily farmwork and biosecurity interventions. This intra-individual focus allowed us to circumvent many of the biases that beset ecological analyses, but at the expense of external validity and ability to draw conclusions about the long-term impact of farm work as compared to non-farm work.

MATERIALS AND METHODS

Study participants (N=10) were enrolled from a single commercial sow facility in the midwestern United States. Participants self-sampled their skin from multiple pre-defined body sites (Figure 1) at three collection phases: prior to showering at the beginning of work (T1), prior to showering at the end of the workday (T2), and after showering at the end of the workday (T3). During the workday, swine that came into contact with enrolled workers were also sampled. Details of study participants and sample collection protocols are included in SI Appendix. All samples were subjected to total DNA extraction, which was further processed for 16S rRNA sequencing and capture-based enrichment and sequencing of ARGs and MGEs using biotinylated probes. Sample processing details can be found in SI Appendix. Resulting sequence datasets were used to perform microbiome, resistome and mobilome profiling, with a focus on comparing differences between collection phases and between worker and swine skin samples. Bioinformatic and statistical analysis details can be found in the **Supplementary Information Appendix**.

REFERENCES

1. B. G. Wu, *et al.*, Evidence for Environmental–Human Microbiota Transfer at a Manufacturing Facility with Novel Work-related Respiratory Disease. *Am J Respir Crit Care Med* **202**, 1678–1688 (2020).
2. P. S. Lai, D. C. Christiani, Impact of occupational exposure on human microbiota. *Curr Opin Allergy Clin Immunol* **19**, 86–91 (2019).
3. P. S. Lai, *et al.*, Impact of environmental microbiota on human microbiota of workers in academic mouse research facilities: An observational study. *PLOS ONE* **12**, e0180969 (2017).
4. A. M. Marcelloni, *et al.*, How Working Tasks Influence Biocontamination in an Animal Facility. *Applied Sciences* **9**, 2216 (2019).
5. J. Sun, *et al.*, Environmental remodeling of human gut microbiota and antibiotic resistome in livestock farms. *Nature Communications* **11**, 1427 (2020).
6. S. J. Song, *et al.*, Cohabiting family members share microbiota with one another and with their dogs. *Elife* **2**, e00458 (2013).
7. N. Mucci, *et al.*, WORKbiota: A Systematic Review about the Effects of Occupational Exposure on Microbiota and Workers' Health. *IJERPH* **19**, 1043 (2022).
8. M. Peng, D. Biswas, Environmental Influences of High-Density Agricultural Animal Operation on Human Forearm Skin Microflora. *Microorganisms* **8**, 1481 (2020).
9. D. Yang, *et al.*, Antimicrobial resistance genes aph(3')-III, erm(B), sul2 and tet(W) abundance in animal faeces, meat, production environments and human faeces in Europe. *J Antimicrob Chemother* **77**, 1883–1893 (2022).
10. L. Van Gompel, *et al.*, Description and determinants of the faecal resistome and microbiome of farmers and slaughterhouse workers: A metagenome-wide cross-sectional study. *Environ Int* **143**, 105939 (2020).
11. A. M. Hammerum, *et al.*, Characterization of extended-spectrum β-lactamase (ESBL)-producing Escherichia coli obtained from Danish pigs, pig farmers and their families from farms with high or no consumption of third- or fourth-generation cephalosporins. *Journal of Antimicrobial Chemotherapy* **69**, 2650–2657 (2014).
12. A. Oppliger, *et al.*, Antimicrobial Resistance of Staphylococcus aureus Strains Acquired by Pig Farmers from Pigs. *Appl Environ Microbiol* **78**, 8010–8014 (2012).
13. J. Sun, *et al.*, Longitudinal study of Staphylococcus aureus colonization and infection in a cohort of swine veterinarians in the United States. *BMC Infect Dis* **17**, 690 (2017).
14. S. M. Hatcher, *et al.*, The Prevalence of Antibiotic-Resistant Staphylococcus aureus Nasal Carriage among Industrial Hog Operation Workers, Community Residents, and Children Living in Their Households: North Carolina, USA. *Environ. Health Perspect.* **125**, 560–569 (2017).
15. Wages and Benefits for Farm Employees | Ag Decision Maker (March 27, 2023).
16. Swine Human Resources: Managing Employees – Hogs, Pigs, and Pork (March 27, 2023).
17. CDC Interim Guidance for Workers who are Employed at Commercial Swine Farms: Preventing the Spread of Influenza A Viruses | CDC (2020) (April 1, 2023).
18. USDA APHIS | Pork Producers (April 1, 2023).
19. E. A. Grice, *et al.*, Topographical and Temporal Diversity of the Human Skin Microbiome. *Science* **324**, 1190–1192 (2009).
20. A. Bouslimani, *et al.*, Molecular cartography of the human skin surface in 3D. *Proceedings of the National Academy of Sciences* **112**, E2120–E2129 (2015).
21. G. Agostinetto, *et al.*, SKIOME Project: a curated collection of skin microbiome datasets enriched with study-related metadata. *Database* **2022**, baac033 (2022).
22. Y. Luo, *et al.*, Dynamic Distribution of Gut Microbiota in Pigs at Different Growth Stages: Composition and Contribution. *Microbiology Spectrum* **10**, e00688-21 (2022).

23. Y. Ramayo-Caldas, *et al.*, Phylogenetic network analysis applied to pig gut microbiota identifies an ecosystem structure linked with growth traits. *ISME J* **10**, 2973–2977 (2016).
24. J. Moor, *et al.*, Influence of pig farming on human Gut Microbiota: role of airborne microbial communities. *Gut Microbes* **13**, 1–13 (2021).
25. J. J. van Rensburg, *et al.*, The Human Skin Microbiome Associates with the Outcome of and Is Influenced by Bacterial Infection. *mBio* **6**, 10.1128/mbio.01315-15 (2015).
26. M. Arikan, *et al.*, Axillary Microbiota Is Associated with Cognitive Impairment in Parkinson's Disease Patients. *Microbiology Spectrum* **10**, e02358-21 (2022).
27. N. R. Noyes, *et al.*, Enrichment allows identification of diverse, rare elements in metagenomic resistome-virulome sequencing. *Microbiome* **5**, 142 (2017).
28. A.-N. Zhang, *et al.*, An omics-based framework for assessing the health risk of antimicrobial resistance genes. *Nat Commun* **12**, 4765 (2021).
29. T. K. Nielsen, P. D. Browne, L. H. Hansen, Antibiotic resistance genes are differentially mobilized according to resistance mechanism. *GigaScience* **11**, giac072 (2022).
30. M. F. Davis, *et al.*, Occurrence of Staphylococcus aureus in swine and swine workplace environments on industrial and antibiotic-free hog operations in North Carolina, USA: a One Health pilot study. *Environ Res* **163**, 88–96 (2018).
31. M. L. Strube, J. E. Hansen, S. Rasmussen, K. Pedersen, A detailed investigation of the porcine skin and nose microbiome using universal and Staphylococcus specific primers. *Sci Rep* **8**, 12751 (2018).
32. L. May, E. Y. Klein, R. E. Rothman, R. Laxminarayan, Trends in Antibiotic Resistance in Coagulase-Negative Staphylococci in the United States, 1999 to 2012. *Antimicrobial Agents and Chemotherapy* **58**, 1404–1409 (2014).
33. K. Becker, C. Heilmann, G. Peters, Coagulase-Negative Staphylococci. *Clin Microbiol Rev* **27**, 870–926 (2014).
34. E. M. Barros, H. Ceotto, M. C. F. Bastos, K. R. N. dos Santos, M. Giambiagi-deMarval, Staphylococcus haemolyticus as an Important Hospital Pathogen and Carrier of Methicillin Resistance Genes. *J Clin Microbiol* **50**, 166–168 (2012).
35. S. S. Costa, *et al.*, Genetic Diversity of norA, Coding for a Main Efflux Pump of Staphylococcus aureus. *Frontiers in Genetics* **9** (2019).
36. R. V. Houdt, *et al.*, *The Tn4371 ICE Family of Bacterial Mobile Genetic Elements* (Landes Bioscience, 2013) (March 31, 2023).
37. M. Razavi, E. Kristiansson, C.-F. Flach, D. G. J. Larsson, The Association between Insertion Sequences and Antibiotic Resistance Genes. *mSphere* **5**, e00418-20 (2020).
38. A. P. Roberts, P. Mullany, Tn916-like genetic elements: a diverse group of modular mobile elements conferring antibiotic resistance. *FEMS Microbiology Reviews* **35**, 856–871 (2011).
39. B. P. Howden, *et al.*, Complete Genome Sequence of Staphylococcus aureus Strain JKD6008, an ST239 Clone of Methicillin-Resistant Staphylococcus aureus with Intermediate-Level Vancomycin Resistance. *Journal of Bacteriology* **192**, 5848–5849 (2010).
40. H. Sanderson, *et al.*, Comparative genomics of multidrug-resistant Enterococcus spp. isolated from wastewater treatment plants. *BMC Microbiol* **20**, 20 (2020).
41. X. Bellanger, S. Payot, N. Leblond-Bourget, G. Guédon, Conjugative and mobilizable genomic islands in bacteria: evolution and diversity. *FEMS Microbiology Reviews* **38**, 720–760 (2014).
42. M. Sebaihia, *et al.*, The multidrug-resistant human pathogen Clostridium difficile has a highly mobile, mosaic genome. *Nat Genet* **38**, 779–786 (2006).
43. D. K. Welter, *et al.*, Free-Living, Psychrotrophic Bacteria of the Genus Psychrobacter Are Descendants of Pathobionts. *mSystems* **6**, e00258-21 (2021).

44. K. G. Byrne-Bailey, *et al.*, Prevalence of sulfonamide resistance genes in bacterial isolates from manured agricultural soils and pig slurry in the United Kingdom. *Antimicrob Agents Chemother* **53**, 696–702 (2009).
45. J. F. Cobo-Díaz, *et al.*, Microbial colonization and resistome dynamics in food processing environments of a newly opened pork cutting industry during 1.5 years of activity. *Microbiome* **9**, 204 (2021).
46. A. C. Ingham, *et al.*, Dynamics of the Human Nasal Microbiota and Staphylococcus aureus CC398 Carriage in Pig Truck Drivers across One Workweek. *Applied and Environmental Microbiology* **87**, e01225-21 (2021).
47. C. Chen, *et al.*, Expanded catalog of microbial genes and metagenome-assembled genomes from the pig gut microbiome. *Nat Commun* **12**, 1106 (2021).
48. S. Saheb Kashaf, *et al.*, Integrating cultivation and metagenomics for a multi-kingdom view of skin microbiome diversity and functions. *Nat Microbiol* **7**, 169–179 (2022).
49. K. Arikawa, *et al.*, Recovery of strain-resolved genomes from human microbiome through an integration framework of single-cell genomics and metagenomics. *Microbiome* **9**, 202 (2021).
50. G. Liu, *et al.*, Metagenomic Analysis Reveals New Microbiota Related to Fiber Digestion in Pigs. *Frontiers in Microbiology* **12** (2021).
51. M. Crossfield, *et al.*, Archaeal and Bacterial Metagenome-Assembled Genome Sequences Derived from Pig Feces. *Microbiol Resour Announc* **11**, e01142-21.
52. J. Ahn, R. B. Hayes, Environmental Influences on the Human Microbiome and Implications for Noncommunicable Disease. *Annual Review of Public Health* **42**, 277–292 (2021).
53. Environmental factors shaping the gut microbiome in a Dutch population | Nature (April 1, 2023).
54. D. Rothschild, *et al.*, Environment dominates over host genetics in shaping human gut microbiota. *Nature* **555**, 210–215 (2018).
55. J. Oh, A. L. Byrd, M. Park, H. H. Kong, J. A. Segre, Temporal Stability of the Human Skin Microbiome. *Cell* **165**, 854–866 (2016).
56. E. K. Costello, *et al.*, Bacterial Community Variation in Human Body Habitats Across Space and Time. *Science* **326**, 1694–1697 (2009).
57. M. Boxberger, V. Cenizo, N. Cassir, B. La Scola, Challenges in exploring and manipulating the human skin microbiome. *Microbiome* **9**, 125 (2021).
58. K. R. L. Larson, T. C. Smith, K. J. Donham, Self-reported Methicillin-resistant Staphylococcus aureus infection in USA pork producers.
59. A. Beaudoin, S. Johnson, P. Davies, J. Bender, M. Gramer, Characterization of Influenza A Outbreaks in Minnesota Swine Herds and Measures Taken to Reduce the Risk of Zoonotic Transmission. *Zoonoses and Public Health* **59**, 96–106 (2012).
60. K. P. Myers, *et al.*, Are swine workers in the United States at increased risk of infection with zoonotic influenza virus? *Clin Infect Dis* **42**, 14–20 (2006).
61. C. Chen, F. Wu, Livestock-associated methicillin-resistant Staphylococcus aureus (LA-MRSA) colonisation and infection among livestock workers and veterinarians: a systematic review and meta-analysis. *Occup Environ Med* **78**, 530–540 (2021).
62. R. N. Sieber, *et al.*, Drivers and Dynamics of Methicillin-Resistant Livestock-Associated Staphylococcus aureus CC398 in Pigs and Humans in Denmark. *mBio* **9**, e02142-18 (2018).
63. D. Sudatip, N. Mostacci, V. Thamlikitkul, A. Oppliger, M. Hilty, Influence of occupational exposure to pigs or chickens on human gut microbiota composition in Thailand. *One health* **15**, 100463 (2022).
64. D. Sudatip, *et al.*, The risk of pig and chicken farming for carriage and transmission of

Escherichia coli containing extended-spectrum beta-lactamase (ESBL) and mobile colistin resistance (mcr) genes in Thailand. *Microbial Genomics* **9**, 000951 (2023).
65. A. Maciel-Guerra, *et al.*, Dissecting microbial communities and resistomes for interconnected humans, soil, and livestock. *ISME J* **17**, 21–35 (2023).
66. O. Mencía-Ares, *et al.*, Genomic Insights into the Mobilome and Resistome of Sentinel Microorganisms Originating from Farms of Two Different Swine Production Systems. *Microbiology Spectrum* **10**, e02896-22 (2022).
67. J. G. Kraemer, S. Aebi, A. Oppliger, M. Hilty, The Indoor-Air Microbiota of Pig Farms Drives the Composition of the Pig Farmers' Nasal Microbiota in a Season-Dependent and Farm-Specific Manner. *Applied and Environmental Microbiology* **85**, e03038-18.
68. R. E. C. Luiken, *et al.*, Farm dust resistomes and bacterial microbiomes in European poultry and pig farms. *Environment International* **143**, 105971 (2020).
69. P. V. Kirjavainen, *et al.*, Farm-like indoor microbiota in non-farm homes protects children from asthma development. *Nat Med* **25**, 1089–1095 (2019).

ACKNOWLEDGEMENTS

We would like to express our gratitude to Lidiya V. Zyskina-Slizovskiy who provided support for graphical data visualization as well as Pavel A. Pevzner for providing valuable feedback in the development of this manuscript.

Ethical approval and consent to participate: The University of Minnesota's Institutional Review Board approved the study (protocol: STUDY 00007351) as no greater than minimal risk to study participants. All procedures, including obtaining informed consent, were followed in accordance with the ethical stands of the Office for Human Research Protections (U.S. Department of Health and Human Services) and with the Helsinki Declaration (2013). All animals were sampled under authorization from the Institutional Animal Care and Use Committees of the University of Minnesota and participating farms under a collaborative agreement (protocol #5-19).

Funding: Financial support for this work was provided by:

National Institute of Health (NIH) National Institute of Allergy and Infectious Disease (NIAID) grant 1R01AI141810-01 (CB)

Midwest Center for Occupational Safety and Health (MCOHS) Pilot Projects Research Training Program (PPRTP) funded through the National Institute of Occupational Safety and Health (NIOSH) grant T42 OH008434 (IBS)

The University of Minnesota Doctoral Dissertation Fellowship (IBS)

Author contributions:

Conceptualization: IBS, NRN, SAD, JN, CAO
Methodology: IBS, NRN, CAO
Investigation: IBS, CAO
Formal Analysis: IBS, TNG, PMF, JEB, NRN
Visualization: IBS, NRN
Supervision: NRN, CB
Writing—original draft: IBS, NRN
Writing—review & editing: IBS, TNG, PMF, CAO, SAD, JN, CB, NRN

Competing interests: All authors declare that they have no competing interests.

Data and materials availability: Upon publication, raw sequence data and sample metadata can be accessed via the Sequence Read Archive (SRA) hosted by the National Center for Biotechnology Information (NCBI) under BioProject PRJNA987158. Sample metadata was recorded using the MIMARKS host-associated metagenomic sample guidelines (Yilmaz et al., 2011). All statistical analysis scripts were executed on R V4.2.0 and are publicly available at https://github.com/IS233489/LaborOME-project.

FIGURE CAPTIONS

Figure 1. Study overview. Farm workers from a commercial farrow-to-wean operation in the Midwestern United States were voluntarily enrolled into a single longitudinal microbiome sampling campaign during a typical 8-hour workday shift. For each worker, swab kits were used to self-collect samples from the epidermis in a standardized fashion by passing each swab across four body sites, achieving a single composite skin sample for the left and right body representing microbiomes from the manus, interdigital space, antecubital fossa, popliteal fossa, and axilla. Workers were asked to perform the first self-collection ('Sample **T1**') prior to entry into the swine facility (**1**). Workers underwent mandatory showering prior to entry into the animal holding areas (**2**). During the day shift, workers were observed handling animals or working in specific animal pens, and dorsal skin swabs (from withers to tail-base) were taken from contact-matched animals on a pooled multi-pen level. Additionally, a 15-minute questionnaire was administered to collect biometric, health, lifestyle, and occupational task performance information from each worker (**3**). In a similar fashion, self-collected skin samples were taken immediately upon conclusion of the workday ('Sample **T2**') (**4**). Workers underwent mandatory showering procedures immediately after exiting the animal holding areas (**5**) and a third self-collection of samples was performed ('Sample **T3**') after showering and immediately prior to exiting the farm facility (**6**).

Figure 2. Changes in skin microbial load, microbiome composition, and community structure. a. Genus-level worker skin microbiome □-diversity across collection phases (T1–T3) and contact-matched swine skin samples using principal component ordination of robust Aitchison compositions. Within-group centroids and 95% confidence intervals are depicted with a large circle and shaded ellipsoids, respectively. **b.** Log_{10}-normalized 16S rRNA qPCR copy number / µl (y-axis), stratified by collection phase (x-axis). *** indicates statistical significance (p<0.001) of pairwise comparisons based on a linear regression model with Tukey's adjustment for multiple comparisons. **c.** General linear analysis of the log_{10}-normalized 16S rRNA qPCR copy number / µl (y-axis) and log_{10}-normalized hourly exposure to swine (x-axis) based on workers' estimates from daily task assignments indicates a negative correlation (p= 0.01) across all collection phases. Shaded areas represent the 95% confidence interval around the linear trendline. **d.** Worker skin microbiome networks across workday collection phases and for contact-matched swine were inferred from inverse covariance estimation for compositions based on centered-log ratios of subsetted ASV counts containing >100 counts per ASV per sample. Inferred networks consist of nodes representing ASVs colored by shared subcommunity membership. Edges between nodes represent a significant predicted positive (blue) or negative (gray) interaction. Reported topology characteristics include network connectivity based on the edge to node ratio (*E:N*), modularity (*Q*), subcommunity assortativity (r_n), and degree assortativity (r_d). **e**. Scatter plots of microbial constituents from the corresponding networks are displayed based on the log_{10}-normalized node eigenvector centrality (y-axis) and node degree centrality (x-axis). Taxa with the highest centrality measures (top right of the distribution) are considered to be critical connectors and major hubs in community networks, and thus putative keystone taxa. ASV-level nodes are colored based on their taxonomic classification at the Class level. Genus-level labels are displayed only for genera most likely to be keystone, i.e., >95th percentile of the plot distribution (top right) and least likely to be keystone, i.e.<5th percentile of the plot distribution (bottom left).

Figure 3. Occurrence of medically important ARGs on the skin of workers and swine. Unique medically important (i.e., priority) ARG alleles were identified at >99.9% alignment gene coverage across all collection phases (top ribbon annotation) and are displayed using a heatmap summarizing their sample-level relative abundance across each of the respective 19 ARG gene groups. The cladograms along the x-axis demonstrate the hierarchical clustering of samples according to their medically important resistome composition using optimal leaf sorting and euclidean distances. Four major subclades are colored and numbered. Major ARG group prevalence and median abundance across study samples are summarized via the associated barplots and boxplots along the y-axis.

Figure 4. Differential abundance analysis across collection phases and at the interface period between workers and pigs. Volcano plots are used to visualize differential abundance of unique **a** ARG groups and **b** MGE accessions in log_2-fold change (x-axis) and $-log_{10}P$ value (y-axis) of the global worker skin resistome or mobilome between key workshift collection phases: Workday start (**T1**) vs. workday end (**T2**); Workday end (**T2**) vs. post-shower (**T3**); and Workday start (**T1**) vs. post-shower (**T3**). An additional comparison is made between workday end and swine skin samples representing the worker's contact phase with animals (gray). Features with significant shift in abundance (Wald's p<0.01 with FDR adjustment for ARG group and MGE accessions) are displayed above the horizontal line, while biologically significant fold-change is demarcated by vertical dashed lines at 1.5 log_2-fold change. Labels are displayed for only the 5 most abundant ARG

groups and 10 most abundant MGEs significantly amplified (log_2-fold change >1.5 or < -1.5) at each phase comparison. For each volcano plot, an associated pie chart displays the number of unique ARG groups and MGE accessions common to each of the workshift collection phases compared, as well as the proportion of the total differentially abundant MGEs associated with each phase.

Figure 5. Taxonomic diversity and novelty of resolved metagenomic assembled genomes (MAGs) recovered from the human-swine interface and ambient environment. **a** Scatter plot of points representing individual GTDB taxonomically binned MAGs according to their estimated percent completeness (x-axis) relative to their estimated percent contamination (y-axis). Point size is proportional to the quantified MAG GC-content. Pink, purple, and gray regions of the plot demarcate the density of 'high quality', 'medium quality-low contamination', and 'medium quality' MAGs retrieved across all collection phases. **b** Scatter plot of estimated MAG size is displayed based on the log_{10}-normalized nucleotide count (x-axis) and the nearest assigned NCBI reference genome size expressed in log_{10}-normalized nucleotide count (y-axis). A global regression line and 95% confidence interval (shaded region) is displayed and results of significance testing (R^2; p<0.05) using a generalized linear model are colored for MAGs recovered for each collection phase. **c** Phylogenomic tree of de-replicated and high-confidence MAGs recovered from target-enriched metagenomes across each collection phase (outer ring). The area below each leaf is colored according to the taxonomically assigned Phylum. Branches for proposed novel species (ANI <95%) are displayed in teal, and for proposed novel strains (ANI <99%) are displayed in purple.

Click here to access/download
Supplementary Material
GIGASCI_Supplementary_Materials_Submission.docx

Supplementary_datafile_1

Click here to access/download
Supplementary Material
Supplementary_datafile_1_metadata.csv

Supplementary_datafile_2

Click here to access/download

Supplementary Material

Supplementary_datafile_2_microbiome.data.file.counts.c
sv

Supplementary_datafile_3

Click here to access/download
Supplementary Material
Supplementary_datafile_3_microbiome.data.file.taxa.csv

Click here to access/download
Supplementary Material
Supplementary_datafile_4_DESeq_results_microbiome.x
lsx

Supplementary_datafile_5

Click here to access/download

Supplementary Material

Supplementary_datafile_5_resistome.data.file.counts.csv

Supplementary_datafile_6

Click here to access/download
Supplementary Material
Supplementary_datafile_6_resistome.data.file.annotation
.csv

Click here to access/download
Supplementary Material
Supplementary_datafile_7_mobilome.data.file.counts.csv

Click here to access/download
Supplementary Material
Supplementary_datafile_8_mobilome.data.file.annotation
.csv

Click here to access/download
Supplementary Material
Supplementary_datafile_9_StrainPhlAn_analysis.counts.csv

Supplementary_datafile_10

Click here to access/download
Supplementary Material
Supplementary_datafile_10_DESeq_results_resistome.xl
sx

Click here to access/download
Supplementary Material
Supplementary_datafile_11_DESeq_results_mobilome.xl
sx

Click here to access/download
Supplementary Material
Supplementary_datafile_12_MAG_metadata.csv

Cover letter
Click here to access/download;Personal Cover;CoverLetter.docx

August 25th, 2024

Dear editorial board,

We are pleased to submit our manuscript entitled "*Reducing Skin Microbiome Exposure Impacts Through Swine Farm Biosecurity*" to the Journal *GigaScience*.

Our work tackles a fundamental question within environmental microbiome and public health risk assessment research which is highly salient to a broad audience*: How do workplace exposures impact the human microbial ecosystem*? Our study focuses on a unique cohort of workers, i.e., individuals who work in a commercial U.S. swine farm. Due to sensitivities regarding the tightly controlled environment and welfare of pigs in the U.S. food production system, the study of such farm workers is scarce, and the biological samples obtained in our worker-swine microbial profiles are novel. A major advancement is that we used occupational health-aware study and sampling design, which led to our discovery that **existing farm biosecurity practices significantly influences the worker skin-borne microbial interactions, composition, and the antimicrobial resistome carry-over from the farm environment. This finding has direct and immediate implications for microbiome researchers, as well as public health and occupational health experts.**

Additionally, we implemented an advanced molecular-sequencing approach to increase our sensitivity to detect antimicrobial resistance genes within the microbiomes of workers. This allowed us to detect low-abundance resistance genes with public health importance as well as the identification of novel bacterial species and strains within the swine farm environment, which we describe in the manuscript.

This work thus provides fundamental knowledge about the microbiome and antimicrobial resistance dynamics of a relatively under-studied human worker population. Importantly, this population also plays an important interfacing role between livestock populations and rural communities. As such, we believe our work will be of great interest to a wide readership.

This manuscript has not been previously published and is not under review at any other journal. All listed authors have contributed significantly to this research based on the requirement for authorship guidelines and declare no conflicts of interest. The study from which data was generated was conducted in accordance with University of Minnesota and U.S. federal guidelines pertaining to human subject investigations, including the acquisition of informed consent following the review of study details and expectations with each participant. Steps to ensure security of any associated personal identifying information were taken. All raw sequencing data and metadata will be made accessible via the Sequenced Read Archive (SRA) under BioProject PRJNA987158. All statistical scripts are made publicly available at https://github.com/IS233489/LaborOME-project.

Thank you for your consideration of our submitted manuscript.

Sincerely,

Dr. Noelle Noyes, MA, DVM, PhD
Associate Professor
University of Minnesota
1988 Fitch Avenue, St. Paul, MN, 55108
Office: 612-624-3562, Mobile: 617-953-7837
