# Supplementary material for: Reducing skin microbiome exposure impacts through swine farm biosecurity
Source: Gigascience. 2025 Jul 26;14:giaf062. doi: 10.1093/gigascience/giaf062 (PMC12810053; doi:10.1093/gigascience/giaf062)
Supplement: giaf062_GIGA-D-24-00356_Revision_1 [file giaf062_giga-d-24-00356_revision_1.pdf]

GigaScience

Reducing Skin Microbiome Exposure Impacts Through Swine Farm Biosecurity

--Manuscript Draft--

**Manuscript Number:**	GIGA-D-24-00356R1
**Full Title:**	Reducing Skin Microbiome Exposure Impacts Through Swine Farm Biosecurity
**Article Type:**	Research
**Funding Information:**	Division of Intramural Research, National Institute of Allergy and Infectious Diseases (1R01AI141810-01) — Dr Christina Boucher<br>National Institute for Occupational Safety and Health (T42 OH008434) — Dr Ilya Slizovskiy<br>National Institute for Occupational Safety and Health (2 U54OH010170-11-00) — Dr Noelle R Noyes
**Abstract:**	Livestock work is unique due to worker exposure to animal-associated microbiomes within the workplace. Swine workers are a unique cohort within the United States livestock labor force, as they have direct daily contact with pigs and undertake mandatory biosecurity interventions. However, investigating this occupational cohort is challenging, particularly within tightly regulated commercial swine operations. Thus, little is known about the impacts of animal exposure and biosecurity protocols on the swine worker microbiome. We obtained unique samples from U.S. swine workers, using a longitudinal study design to investigate temporal microbiome dynamics. We observed a significant increase in bacterial DNA load on worker skin during the workday, with concurrent changes in the composition and abundance of microbial taxa, resistance genes and mobile genetic elements. However, mandatory showers at the end of the workday partially returned the skin's microbiome and resistome to their original state. These novel results from a human cohort demonstrate that existing biosecurity practices ameliorate work-associated microbiome impacts.
**Corresponding Author:**	Noelle Noyes<br>University of Minnesota College of Veterinary Medicine: University of Minnesota Twin Cities College of Veterinary Medicine<br>UNITED STATES
**Corresponding Author Secondary Information:**	
**Corresponding Author's Institution:**	University of Minnesota College of Veterinary Medicine: University of Minnesota Twin Cities College of Veterinary Medicine
**Corresponding Author's Secondary Institution:**	
**First Author:**	Ilya Slizovskiy, DVM, PhD, MPH
**First Author Secondary Information:**	
**Order of Authors:**	Ilya Slizovskiy, DVM, PhD, MPH
	Tara N Gaire, DVM, PhD
	Peter M Ferm, M.Sc.
	Carissa A Odland, DVM, M.Sc.
	Scott A Dee, DVM, PhD
	Joel Nerem, DVM, M.Sc.
	Jonathan E Bravo, M.Sc.
	Alejandro D. Kimball
	Christina Boucher, PhD

Powered by Editorial Manager® and ProduXion Manager® from Aries Systems Corporation

	Noelle R Noyes, DVM, PhD
**Order of Authors Secondary Information:**	
**Response to Reviewers:**	RE: GIGA-D-24-00356<br>Reducing Skin Microbiome Exposure Impacts Through Swine Farm Biosecurity<br>Ilya Slizovskiy; Tara N Gaire; Peter M Ferm; Carissa A Odland; Scott A Dee; Joel Nerem; Jonathan E Bravo; Christina Boucher; Noelle R Noyes<br>Dear Editor and Reviewers,<br>We thank you for your thorough and thoughtful reviews of our manuscript "Reducing Skin Microbiome Exposure Impacts Through Swine Farm Biosecurity" (GIGA-D-24-00356). We appreciate your detailed feedback and constructive comments, which have been invaluable in improving our study. We have addressed all points raised and made the necessary revisions throughout the manuscript, including updates to the 16S rRNA analysis, methodological clarifications, and textual refinements.<br>Thank you again for your time and insightful suggestions.<br><br>- According to reviewer #1, the 16S reference database used for taxonomic assignment is outdated and should no longer be used; the analysis should be re-done with the up-to-date set.<br>Response: We re-analyzed all 16S-related sequencing data and have uploaded new figures / supporting files to reflect these changes. We have made alterations where 16S analysis is relevant to the text of the resubmission.<br><br>- Both reviewers have technical questions and concerns regarding the protocol and statistical analyses; for example with respect to using 16S data instead of direct metagenomic sequencing, and generally with respect to the sampling protocol.<br>Response: We addressed all concerns regarding our analytical and sequencing approaches. In addition, we re-analyzed metagenomic sequencing data with updated reference databases.<br><br>- Addressing the comments of Reviewer #2 in particular will also require a more careful discussion and more exact wording of conclusions, interpretations, and potential confounders.<br>Response: We made substantial modifications within the text describing the results and in particular within discussion of results, methods, and interpretations in the discussion section.<br><br>On an editorial note, please include the "supplemental methods" in the main manuscript. We have no word limit, and all methods that were used for obtaining the results described in the article should also be part of the main paper instead of the supplement.<br>Response: We incorporated all of the methods described in the supplemental section into the main document of the resubmission.<br><br>Reviewer #1:<br><br>The article titled "Reducing Skin Microbiome Exposure Impacts Through Swine Farm Biosecurity" is very interesting and well-structured. It provides solid methodological information and results, written in a detailed manner that makes it easy to follow. However, there are some major comments that need to be addressed, particularly related to the 16S rRNA sequencing database used for taxonomic assignment (Silva 132), which is from 2017 and does not reflect the important taxonomic changes made since then. Therefore, I highly recommend reanalyzing these data with an updated database. Below are the detailed comments.<br><br>General comments:<br><br>- Comment 1. Please include spaces between citations and the text. Review this structure throughout the manuscript.<br>Response: We noted this suggestion, and made changes to in-text citations throughout the main text and supplementary materials document.

- Comment 2. Greek letters (alpha, beta) appear as squares in several places throughout the text. Please review.
Response: Thank you for bringing this to our attention. After reviewing our document we believe this could have happened during the conversion step to PDF in the submission portal. We ensured that this did not appear in the resubmission stage.

Abstract:
- Comment 3. Line 51. Replace "U.S." with "United States".
Response: We have made the suggested change in the abstract, in line 47.

Introduction:
- Comment 4. Section title. Please replace "MAIN TEXT" with "INTRODUCTION".
Response: We have made the suggested change to the section title.

- Comment 5. Line 90. The phylum Proteobacteria is currently categorized as Pseudomonadota.
Response: We made the suggested change to the Phylum name of Proteobacteria, in line 80.

- Comment 6. Line 97. Please include a citation for studies tracking daily animal exposure and on-site worker behavior.
Response: We made the suggested change by adding two relevant citations, in lines 85–87.

- Comment 7. Line 102. Include the abbreviation for "United States" for further use throughout the manuscript.
Response: We made the suggested change by introducing the ‘U.S.’ abbreviation, in line 92.

Materials and methods - Supplementary Information
- Comment 8. Materials and Methods general comment. I understand the relevance of using a target-enriched metagenomic library preparation for AMR and MGE, given their low representation in samples. However, many studies assessing the mobilome and resistome conduct sequencing without prior enrichment, which reduces bias in the analysis, especially in uncharacterized MGEs. Additionally, this approach created the need for 16S rRNA sequencing analysis, which used an outdated database; this would not have been necessary with direct metagenomic sequencing without enrichment, enabling taxonomic assignment using updated tools such as Kraken. Furthermore, MetaPhlAn3 and MetaBAT2 were used for detecting taxonomic markers and MAGs binning, respectively, which introduces some bias due to AMR and MGE marker enrichment. Please explain the reasoning behind this approach and its potential limitations. Wouldn't it be possible to assign taxonomy directly from the metagenomic approach?
Response: We thank the reviewer for the opportunity to clarify these issues, and we address them here and in the main text of the manuscript, especially in the Discussion section:

Use of targeted enrichment vs. traditional metagenomics: The target-enriched shotgun sequencing approach was utilized for high-sensitivity resistome and mobilome analysis (Figures 3 and 4). We have considerable experience in the use of targeted metagenomic approaches for the characterization of resistomes and mobilomes in both short read (PMID: 29041965) and long-read (PMID: 36324140, PMID: 39500537) sequencing platforms. It has been established that targeted sequencing yields a more accurate characterization of low-abundance genes of public health importance, including ARGs and MGEs that are traditionally under-sequenced by standard shotgun metagenomics, by multiple orders of magnitude. However, even with targeted approaches, molecular enrichment in metagenomic workflows does not result in 100% on-target sequencing of targets—Indeed, our metadata (Supplementary datafile 1) demonstrates that the resistome and mobilome accounted for a median of 15% and 10% across all metagenomes analyzed in this study. The remaining >85% of the ‘off-target’ metagenomic data is still useful. We used this off-target data to perform MAG construction and select strain-level analyses that focused primarily on sentinel strains commonly associated with human and animal enteric and adnexal microbiomes (E. coli, Streptococci, and Staphylococci). Because the off-target sequence data may be

	biased (due to the enrichment and PCR amplification process), it is not suitable for comprehensive taxonomic analyses with metagenomic tools like Kraken. We therefore relied on the 16S rRNA amplicon sequencing approach for more robust taxonomic characterization. This approach yields reliable classification to the genus level. We have added further discussion on the reasoning of using targeted-metagenomic approaches in the study, as well as their advantages and limitations in lines 638–660.<br><br>Target-enrichment approaches may bias sequencing: The reviewer notes that many studies utilize traditional metagenomic sequencing without any prior enrichment, and that this "...reduces bias in the analysis, especially in uncharacterized MGEs." We extended our discussion describing this method as well as its well-researched foundations, including work that has been extensively done in our labs over the years. We note that the study of uncharacterized genes was not the focus of our manuscript. Nevertheless, we would like to emphasize that the use of traditional shotgun metagenomic sequencing would not alleviate the issue of under-discovery of novel targets that the reviewer describes, because such data would need to still be compared to a database of known reference sequences either by direct read alignment, or the alignment of de novo assembled contigs. Because we utilized a targeted sequencing approach with built-in allelic flexibility, a larger proportion of sequencing effort was dedicated to ARG and MGE regions and their potential homologs than what otherwise would have been possible in traditional shotgun sequencing. Therefore, our approach may lead to less bias, particularly in terms of detection sensitivity.<br><br>Outdated reference database in the 16S rRNA analysis: We agree with both reviewers that at this point the 16S rRNA SILVA database that we originally used has become outdated. We therefore repeated our entire microbiome analyses using the most updated SILVA reference available, and we updated all of our microbiome results (lines 137–237). This resulted in changes to some of the key taxa that were found to be dominant at various work phase timepoints (and their taxonomic nomenclature), however, the overall patterns in alpha diversity, beta diversity, and network interactions paralleled the original results presented in the primary manuscript submission.<br><br>- Comment 9. The Silva database 132 is updated from December 13, 2017, meaning the taxonomic information is quite outdated, with significant changes in taxonomic classifications in the last seven years, such as phylum renaming. Therefore, I highly recommend reanalyzing the 16S rRNA sequencing data with an updated database that reflects current phyla. Otherwise, I would unfortunately find it difficult to accept this manuscript for publication, given the high impact and relevance of the journal, and despite the quality of the study's statistical and bioinformatics approaches.<br>Response: We thank the reviewer for this important observation, and we agree. As stated above, we re-analyzed and updated all of the microbiome results throughout the manuscript, including in the supplementary figures and datafiles.<br><br>- Comment 10. Materials and Methods general comment. Please ensure that the version of each program and pipeline used in the study is included. For example, lines 231 and 234 mention bioinformatics tools without version numbers. Please also include the version of all R packages.<br>Response: We have made these changes throughout the entire Materials and Methods section, now found in the main manuscript.<br><br>- Comment 11. Line 301. Why were parametric analyses such as ANOVA used? Did the data follow a normal distribution or were normalized?<br>Response: We thank the reviewer for their question regarding meeting assumptions of normality in ANOVA. In all cases, visual analysis of residuals did not indicate significant departure from normality. The inclusion of random effects in the linear mixed model further ensures that the non-independence of the repeated observations per worker were accounted for in the model. Additionally, the lme4 package implementation and the use of linear mixed models are known to be robust to departures from normality, we therefore believe that the use of ANOVA is appropriate. We included additional language to clarify these assumptions in the MATERIALS AND METHODS section (lines 941–943).<br><br>- Comment 12. Lines 318-336. In this section, beta diversity is said to have been

analyzed with PCA. However, in line 512, NMDS is mentioned. Please review the analytical details in the methodology.
Response: We thank the reviewer for catching this discrepancy. We have updated the language, as we have used PCA for this analysis.

Results:
- Comment 13. Sections 1 and 2. The information provided in these sections, while interesting and very well-analyzed, is outdated due to the use of an outdated database, as noted in Comment 9. Therefore, I highly recommend reanalyzing the 16S rRNA sequencing data with an updated database reflecting new phyla classification, as the provided data relies on a 2017 database. For instance, the Prevotella genus has recently been reclassified into different genera, potentially altering the study's results (lines 205-207).
Response: We thank the reviewer for this important observation, and we agree. As stated above, we re-analyzed and updated all of the microbiome results throughout the manuscript.

- Comment 14. Figure 2d. The colors of the lines are difficult to distinguish for positive or negative associations. Please revise the colors or increase the line thickness.
Response: We thank the reviewer for this comment. We increased the line thickness of the network graphs to distinguish the positive and negative associations in Figure 2d.

- Comment 15. Line 233. Replace "flora" with "microbiota."
Response: We thank the reviewer for this comment. We have replaced ‘flora’ with ‘microbiota’ in the revised manuscript (line 234).

- Comment 16. Section "The skin microbiome becomes unstructured and dominated by enteric and environmental microbes during on-farm work." Consider adding a reference to Figure 2e related to Swine in this section and explaining if there is a relationship with the predominant taxa in T2 or T3.
Response: We thank the reviewer for noting this. We omitted referencing to results of the swine network, and we have done that in the resubmission (lines 231–237).

- Comment 17. Line 268. Regarding "up to 44%," this figure applies only to ICEs and TEs, while plasmids and IS reach only about 24%. Please revise to avoid misinterpretation.
Response: We thank the reviewer for catching this obscure wording. We clarified this in the resubmission (lines 278–281).

- Comment 18. Lines 269-270. In Figure S6, significance ($p < 0.05$) is indicated for both viruses and prophages.
Response: We thank the reviewer for catching this mistake, we clarified the wording here as we meant to describe that shifts in viruses and prophages between the collection phases T1 and T2 were more modest by comparison to the other MGEs. (lines 283–284).

- Comment 19. Line 292. Please include the antimicrobial classes for which vga genes confer resistance.
Response: We made additional notes of the antimicrobial classes for which the vga genes confer resistance (lines 313–314).

- Comment 20. Lines 349-358. It is noteworthy that while Figure 3 shows the highest abundance and prevalence of mecA in worker samples from T1 and T3, there is a significant increase in Figure 4a for mecA in T2, which remains elevated in T3. Could you provide an explanation or further discuss this?
Response: We thank the reviewer for noting this. Two sources contribute to these differences between the two figures, including threshold of detection as well as method of analysis. Figure 3 shows normalized abundance and prevalence results on a per-sample basis using a uniquely high-threshold gene detection approach (i.e. >99.99% gene fraction cutoff in the read alignment step) applied to a subset of the resistome that we term: medically important ARGs. Conversely, Figure 4a shows results of differential abundance for the overall resistome, detected by read alignment with a default >80% gene fraction cutoff. The results in Figure 4a are based on an aggregate of counts across all samples at the ARG group level, with model inclusion for both

sequencing and worker biometric covariates.

- Comment 21. MAGs section and Figure 5c. Please include the updated phylum taxonomy.
Response: We thank the reviewer for noting this, we made the changes in this figure as well as in the corresponding text (1st paragraph of MAG section). We also checked the other names to ensure that all other phyla are up to date with the current List of Prokaryotic names with Standing in Nomenclature.

- Comment 22. Line 429. Please include the abbreviation of GTDB.
Response: We thank the reviewer for this comment. We clarified the GTDB acronym in the revised manuscript (1st paragraph of MAG section).

- Comment 23. Figure S10 is not referenced in the manuscript. Please check if it is needed or should be removed.
Response: This figure was not referenced and was not needed. Hence, we removed this figure from the supplementary files portion of the manuscript.

Discussion:

- Comment 24. Line 578. Replace "his" with "this."
Response: This change was made in line 666.

- Comment 25. General comment. I would recommend including information on the limitations and benefits of metagenomic enrichment, as the mobilome may include MGEs that remain undescribed, which could lead to their underrepresentation. Additionally, it would be beneficial to highlight the advantages of combining 16S with metagenomic enrichment, given that the more common approach is metagenomic analysis with taxonomic assignment from these sequences, without prior enrichment.
Response: We thank the reviewer for their comment, we addressed it in our response to comment 8.

Reviewer #2: Slizovskiy et al. studied the skin microbiome, resistome and mobilome compositions of swine workers at three stages of their working day, before, during and at the end. They conclude that showering as part of their biosecurity measures at the swine farm effectively contains potential pathogen and AMR spread. The study is of interest and too little is known on occupational exposure effects in the flow of pathogen and AMR spread.

However, I do have a few more generic and more specific questions/remarks related to the study, the text and figures.

Generic remarks;

1) The statement of restoring the microbiome to baseline (i.e. line 60 and 536) seems too strong as the authors themselves state for instance at page 14 that the microbiome composition is not restored. Furthermore there are significant differences between T1 and T3 (lines 200-204).
Response: Thank you for highlighting the need for precision in describing our findings. We have revised the wording in the abstract (lines 55–56) to clarify that while there is evidence of partial reversion towards baseline, complete restoration was not observed—as also detailed on page 14. Additionally, we have acknowledged the significant differences between T1 and T3 and adjusted the discussion accordingly. We

	appreciate your input, which has helped us better reflect the nuances of our results.<br><br>2) I am also a bit puzzled about the actual exposure and what the authors measured; skin microbiome at various places on the body (fig 1) which were pooled to one sample. Is protective clothing (as imaged in fig 1) not part of biosecurity? How come the workers get microbial exposure at most likely completely covered regions of their body? In line 554 they write full exposure of the skin. Could another confounder be the result of all these microbial changes at T2? This should be made more clear in text and discussion (and/or change fig1 image as that might be misleading?).<br>Response: We thank the reviewer for their comment. We clarified the sampling procedure and protocol in lines (lines 605–618) of the discussion section. Our sampling procedure was strategically conducted to account for a number of possible sources of microbial variation. This includes skin biogeography and its variable impact on how taxa will change over the workday, as well as different regions that may have either higher or lower exposures to the swine farm environment. We purposely chose skin sampling sites that represent a range of exposures (both clothing-covered- and uncovered- skin). This was done as there have been no studies to suggest the existence of optimal skin sites for microbiome sampling in occupational contexts. Our approach is highly conservative. We reason that since microbiome alterations were detected despite sampling skin locations that are less exposed to the farm environment, this shift represents a likely under-estimate and enhances the credibility and robustness of the results.<br>We agree that biosecurity measures such as protective clothing may impact worker microbiomes throughout the workday. However, the effect of PPE during the workday cannot be discerned in this study design as all workers are required to wear the same type of PPE, for approximately the same duration, and used approximately in the same way. We made this more clear in the text (lines 593–603). Therefore, we believe that Figure 1 accurately represents our sampling design and does not introduce any confusion regarding the methodology.<br><br>3) Linked to remark #2; The authors do not see a clear link with for instance environmental samples or pig skin microbiomes. So where did all the stuff in T2 come from? In their discussion it might be worthwhile to also address the potential influence of pig feces as a source? Knowing the source can help to minimize spillover events. (Ilya–I thought we discussed this at length, but perhaps we eliminated some of the language for brevity for the other journals?)<br>Response: We thank the reviewer for their comment. We expanded our discussion of possible sources that may contribute to alteration to worker skin microbiomes, including the influence of swine feces (lines 564–567). However, again, we note that in this preliminary investigation, precise source-tracking of bacteria was not possible as we now discuss in the updated discussion section (lines 570–577).<br><br>4) Some discussion seems to be required on other routes (besides skin) for pathogen and AMR transmission/exposure. The nasal cavity is known to be a direct reflection of the (air) environment, while oral/ingestion might result in colonization of the airways and gut. Thereby providing a continuous source of "baseline" exposure. This might also explain some of the strange observations from Figure 3, for instance the high increase at T2 samples for Vga and Sul1 which do not seem to be highly abundant at high prevalence in non-T2 samples.<br>Response: We appreciate the reviewer's valuable insight regarding alternative exposure routes such as the nasal cavity and oral ingestion. Although our study primarily focused on skin microbiome changes rather than transmission per se, we recognize that these other pathways may contribute to baseline exposures and could help contextualize some of the observations in Figure 3, such as the notable increases in Vga and Sul1 at T2. We discuss in lines 622–624 the influence of prior exposures that may have contributed to the worker's baseline microbiome. As stated in comment #3, we also now reference relevant literature on shifts in the nasal and gastrointestinal microbiomes and acknowledge their potential roles in AMR exposure / burden. We hope this addition adequately addresses your perspective while staying within the scope of our study.<br><br>5) The authors used ANOSIM to do GROUP comparisons of microbial compositions and later PERMANOVAs. Since the same worker is sampled 3 times we have repeated measures for each individual. ANOSIM as rank based group comparison seems not

	the optimal choice. Why not utilizing PERMANOVA for abundance over time using stratification/blocking approach on the individual IDs? This has been applied for 16S qPCR count data (lme). The same accounts for alpha diversity metrics and a glmm approach.<br>Response: We thank the reviewer for raising this point. We performed this additional step using the adonis2 function in the Vegan package during our re-analysis of the ordination PERMANOVAs with individual worker IDs as strata for repeated measures. We updated these results in the manuscript (lines 270–299), and described this step in the methods (lines 973–975). We note that this updated approach did not alter the conclusions reached in comparison to those using the approach from the original analysis.<br><br>6) Why did the authors first use 16S rRNA amplicon sequencing to determine microbial diversity at mostly genus taxonomic level while later shotgun-metagenomics and assembly are utilized of the same samples at strain resolution? Authors describe that similar results for microbial diversity were obtained at lines 427-428.<br>Response: We thank the reviewer for raising this question. We made further clarifications on this point in the main manuscript discussion section (lines 654–660).<br><br>7) Why are "only" 40 samples from phase 1 of the laborOme project utilized and not the complete set of phase 2 (2 facilities, each 40 workers, 3 timepoints)?<br>Response: We apologize to the reviewer for any confusion. We included all of the samples in our analysis for this project. There is no phase 2 and no additional samples were collected beyond those we reported on. We removed any indication of this in our Github page.<br><br>8) The authors measured microbial load using 16S rRNA-based qPCR. Most of the AMR and mobilome data are analysed as compositional data. Even though compositions might answer some of the questions for absence/presence, the actual AMR load is an important metric as described in literature. This AMR load might be inferred using the compositional data and the qPCR data.<br>Response: We agree with this point. We performed additional quantitative and statistical analyses of ARG/ MGE burdens, with updated details in the methods (lines 980–986) and results (lines 270–277 / lines 291–299).<br><br>More specific comments.<br><br>1) References #17 #18 and #53 seem incomplete.<br>Response: We thank the reviewer for catching these. We made updates to these references.<br><br>2) Line 215; filtering conditions. Isn't this filtering way to strict? This will exclude (near) zero or <100 taxon counts taxa which actually may be the most interesting ones. Was the 100 cutoff set and used on even depth rarefied data? Would using a prevalence cutoff not being better?<br>Response: We applied a strict cut-off by taxonomic count, as this is a recommendation provided in the documentation of the SPIEC-EASI tool. While the taxa at near zero (<100 taxon counts) may be of interest, we nevertheless are wary of the possibility of spurious associations that can be reached from these rare taxa, even with the use of robust network approaches for compositional datasets and appropriate normalization techniques as used in the manuscript. Consequently, we consider the implementation of this stringent filtering approach a necessary measure to ensure the reliability of our analyses. However, we agree with the reviewer regarding the use of a prevalence cutoff. In our re-analysis of the microbiome with the updated SILVA reference database, we applied a 10% cutoff (i.e. >10% / >1 sample prevalence) in addition to the counts cutoff. This was updated in the methods section of the resubmission (lines 1025).<br><br>3) Line 221 significant; stats are missing.<br>Response: We thank the reviewer for noting this. We altered the language in this sentence, as there are no specific statistical tests that we are referencing.<br><br>4) Line 233 flora; There is no such think as intestinal flora. Revise to microbiota.<br>Response: We have replaced ‘flora’ with ‘microbiota’ in the revised manuscript across

	multiple areas of the manuscript.<br><br>5) At many places in the document the symbols for beta etc got replaced with a square.<br>Response: Thank you for bringing this to our attention. After reviewing our document we believe this could have happened during the conversion step to PDF in the submission portal for journal review. We ensured that this does not appear in the resubmission stage.<br><br>6) Line 286; One sample T2 was quite different. What was done with this sample (taken out)? How does this effect the previously mentioned >100 counts cutoff applied?<br>Response: We believe that the reviewer may have been mistaken in this comment. We did not apply a 100 count cutoff to the analysis of the medically important ARGs referenced in line 286 of the original submission. In this analysis, we applied a near 100% nucleotide alignment match requirement in order for the pre-specified clinically important ARGs to be considered ‘present’ in a given sample.<br><br>7) Technical issue metaphlan; It has been described and known that especially early versions of metaphlan (the database) was strong bias to annotation for human microbiome-derived data. The authors used version 3, albeit the tool maintainers claim version 4 should have addressed this issue for non-human hosts.<br>Response: We agree with the reviewer’s suggestion and we performed this re-analysis. We repeated our analysis using updated versions of MetaPhlAn and StrainPhlAn subsequent analysis. We updated our results of this analysis throughout the manuscript. We note that overall, this update did not alter the major conclusions reached.<br><br>8) Line 398+ and 403-405; How sure is the suggested link between psychrobacter and its actual presence in the data?<br>Response: Since we performed our analysis with two different versions of MetaPhlAn / StrainPhlAn, we believe we have confirmed the presence of Psychrobacter in our data. This finding parallels Psychrobacter identified via 16S rRNA sequencing, and Psychrobacter MAGs were also recovered. This consistency supports the more than likely preponderance of Psychrobacter’s presence in the various samples.<br><br>9) Line 536-537 biosecurity measures; Looking at the presented data improving protective clothing might be an important consideration which is not discussed.<br>Response: We thank the reviewer for this comment. We increased the discussion of PPE and biosecurity measures in the discussion section (e.g. lines 593–603)<br><br>10) Line 544 process controls; Explanation required.<br>Response: We thank the reviewer for this comment. We provided detailed examples of process controls in the revised manuscript (lines 551–554).
**Additional Information:**	
**Question**	**Response**
Are you submitting this manuscript to a special series or article collection?	No
**Experimental design and statistics**<br><br>Full details of the experimental design and statistical methods used should be given in the Methods section, as detailed in our Minimum Standards Reporting Checklist. Information essential to interpreting the data presented should be made available in the figure legends.	Yes

Have you included all the information requested in your manuscript?	
**Resources**<br><br>A description of all resources used, including antibodies, cell lines, animals and software tools, with enough information to allow them to be uniquely identified, should be included in the Methods section. Authors are strongly encouraged to cite Research Resource Identifiers (RRIDs) for antibodies, model organisms and tools, where possible.<br><br>Have you included the information requested as detailed in our Minimum Standards Reporting Checklist?	Yes
**Availability of data and materials**<br><br>All datasets and code on which the conclusions of the paper rely must be either included in your submission or deposited in publicly available repositories (where available and ethically appropriate), referencing such data using a unique identifier in the references and in the "Availability of Data and Materials" section of your manuscript.<br><br>Have you have met the above requirement as detailed in our Minimum Standards Reporting Checklist?	Yes

Revised_Manuscript

Click here to access/download;Manuscript;Manuscript_Revised.docx

Reducing Skin Microbiome Exposure Impacts Through Swine Farm Biosecurity

Ilya B. Slizovskiy[1,2,3], Tara N. Gaire[3], Peter M. Ferm[3], Carissa A. Odland[4], Scott A. Dee[5], Joel Nerem[5], Jonathan E. Bravo[6], Alejandro D. Kimball[7], Christina Boucher[6], Noelle R. Noyes*[3]

Purdue Applied Microbiome Sciences Program, Purdue University, West Lafayette, IN, USA[1]
Veterinary Clinical Sciences Department, College of Veterinary Medicine, Purdue University, West Lafayette, IN, USA[2]
Food-Centric Corridor, Infectious Disease Laboratory, Department of Veterinary Population Medicine, College of Veterinary Medicine, University of Minnesota, St. Paul, MN, USA[3] Pipestone Veterinary Services, Pipestone, MN, USA[4]
Pipestone Applied Research, Pipestone, MN, US[5]
Department of Computer and Information Science and Engineering, Herbert Wertheim College of Engineering, University of Florida, Gainesville, FL, USA[6]
Regenstrief Center for Healthcare Engineering, Purdue University, West Lafayette, IN, USA[7]

To whom correspondence shall be addressed:

Dr. Noelle R. Noyes
nnoyes@umn.edu
385D AnSci/VM
1988 Fitch Avenue
St. Paul, MN 55108
United States

***Running head*:**

Farm biosecurity shapes microbiomes

Study funding:

Financial support for this work was provided by the National Institute of Health (NIH) National Institute of Allergy and Infectious Disease (NIAID), *Project No. 1R01AI141810-01*; the Midwest Center for Occupational Safety and Health (MCOHS) Pilot Projects Research Training Program (PPRTP) funded through the National Institute of Occupational Safety and Health (NIOSH), *Project No.* T42 OH008434; and the University of Minnesota Doctoral Dissertation Fellowship.

SUPPLEMENTARY FIGURES

SUPPLEMENTARY DATAFILES

ABSTRACT

Livestock work is unique due to worker exposure to animal-associated microbiomes within the workplace. Swine workers are a unique cohort within the United States livestock labor force, as they have direct daily contact with pigs and undertake mandatory biosecurity interventions. However, investigating this occupational cohort is challenging, particularly within tightly regulated commercial swine operations. Thus, little is known about the impacts of animal exposure and biosecurity protocols on the swine worker microbiome. We obtained unique samples from U.S. swine workers, using a longitudinal study design to investigate temporal microbiome dynamics. We observed a significant increase in bacterial DNA load on worker skin during the workday, with concurrent changes in the composition and abundance of microbial taxa, resistance genes and mobile genetic elements. However, mandatory showers at the end of the workday partially returned the skin's microbiome and resistome to their original state. These novel results from a human cohort demonstrate that existing biosecurity practices ameliorate work-associated microbiome impacts.

KEY WORDS

Metagenomics; Microbiome; Antimicrobial resistance; Mobile genetic elements; Farm workers; Public health

INTRODUCTION

Occupational exposures can significantly influence the microbiomes of workers, and in some cases have been linked to health outcomes [1,2]. People working with animals encounter a unique workplace microbiome with frequent exposure to animal microbiomes, either through direct contact or indirect exposures. The impact of animal exposure on human microbiomes has been demonstrated across several settings including research facilities [3,4] and livestock farms [5], as well as within homes[6]. For example, dairy and swine workers have more diverse oral and nasal bacterial taxa than non-livestock workers [7]. Additionally, the skin microbiome of livestock workers harbors a higher relative abundance of Pseudomonadota and lower relative abundance of Actinobacteria and Bacteroidetes compared to people with non-livestock occupations [8]. Livestock-associated bacteria and their antimicrobial resistance genes (ARGs) have been documented in agricultural worker cohorts, including farmers [9–11], veterinarians [12,13] , and abattoir workers [10,14] ; and short-term visitation to swine farms has been linked to an enrichment in farm-associated bacteria and ARGs in the human gut [5]. These findings have been attributed to livestock exposure, but few studies have actually tracked daily animal exposure and on-site worker behavior, particularly on commercial farms [15,16]. Such studies require careful consideration of workplace habits and exposures, and sampling must occur within the constraints of commercial livestock production. Given these challenges, detailed studies of livestock worker microbiomes are uncommon, and the specific influence of occupational exposures on livestock worker microbiomes remains poorly understood.

In the United States (U.S.), swine workers are a unique cohort within the livestock labor force. Their job tasks involve intensive one-on-one animal handling, working within enclosed and climatically regulated facilities, with work duration ranging 48–54 hours per week [17,18]. These working conditions contrast markedly with other production systems; beef cattle and poultry are rarely handled by workers, and cattle work tends to occur in open-air facilities. Moreover, most workers in North American swine farms adhere to strict biosecurity measures to control pathogen transmission between and within farms [19,20], including showering into and out of the farm, which may reduce transfer of microbes and ARGs between swine farms and the general public. However, the impact of mandatory showering on the likelihood of worker-mediated farm-to-community transmission is unknown.

We report on a longitudinal investigation of the swine worker microbiome-resistome on a commercial U.S. swine facility, with sampling occurring as part of a normal workday that included showering as a mandatory biosecurity intervention (**Figure 1**). We observed a significant increase in bacterial DNA load on worker skin during the workday, with concurrent changes in the composition and abundance of specific microbial taxa, ARGs, and mobile genetic elements (MGEs) that could harbor ARGs. We further observed that compulsory showering at the end of the workday reverted the skin microbiome and resistome to a baseline state, which differs from previous results that did not include showering in the study design [5] . These results suggest that occupational work in swine facilities can significantly impact workers' skin microbiomes, but that these impacts can be transient if biosecurity interventions are implemented. The relevancy of these findings for short- and long-term swine worker health requires further study. However, the observation that showering may dampen daily microbiome impacts have important public health implications, as it demonstrates that biosecurity protocols could be leveraged to minimize microbial transmission from farms to general communities.

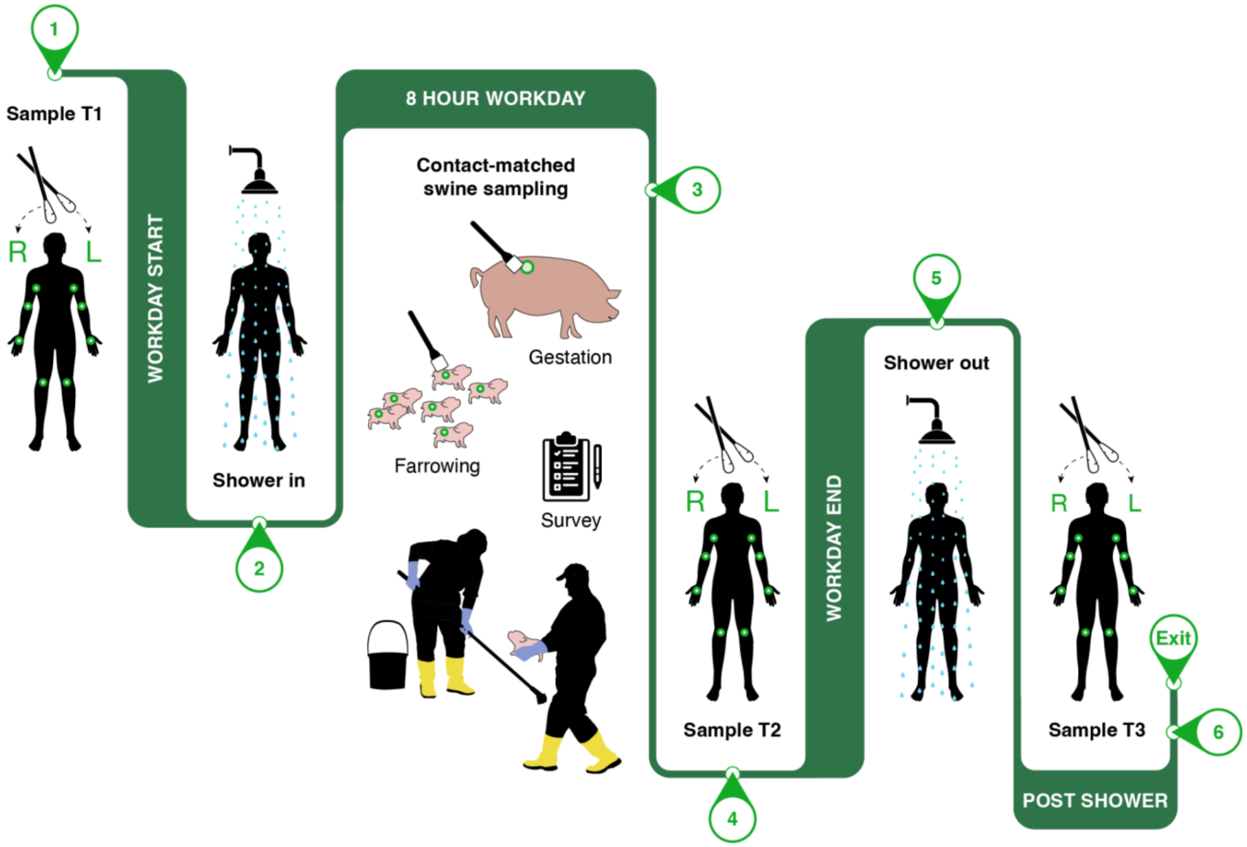

Figure 1. Study overview. Farm workers from a commercial farrow-to-wean operation in the Midwestern United States were voluntarily enrolled into a single longitudinal microbiome sampling campaign during a typical 8-hour workday shift. For each worker, swab kits were used to self-collect samples from the epidermis in a standardized fashion by passing each swab across four body sites, achieving a single composite skin sample for the left and right body representing microbiomes from the manus, interdigital space, antecubital fossa, popliteal fossa, and axilla. Workers were asked to perform the first self-collection ('Sample **T1**') prior to entry into the swine facility (**1**). Workers underwent mandatory showering prior to entry into the animal holding areas (**2**). During the day shift, workers were observed handling animals or working in specific animal pens, and dorsal skin swabs (from withers to tail-base) were taken from contact-matched animals on a pooled multi-pen level. Additionally, a 15-minute questionnaire was administered to collect biometric, health, lifestyle, and occupational task performance information from each worker (**3**). In a similar fashion, self-collected skin samples were taken immediately upon conclusion of the workday ('Sample **T2**') (**4**). Workers underwent mandatory showering procedures immediately after exiting the animal holding areas (**5**) and a third self-collection of samples was performed ('Sample **T3**') after showering and immediately prior to exiting the farm facility (**6**).

RESULTS

Individual skin microbiomes experience dramatic yet transient shifts during on-farm work with swine

16S rRNA sequencing and analysis was performed on 40 skin samples collected longitudinally from 10 healthy and predominantly male swine workers and contact-matched swine (SI Appendix SI Methods, **Supplementary note 1**, **Supplementary file 1**). The skin microbiome composition underwent significant shifts in the course of a single 8-hour workday (ANOSIM $p < 0.001$; R=57.6%, adjusted PERMANOVA $p < 0.001$, **Figure 2a**). Specifically, samples taken at the end of work but prior to showering (T2) had a significantly different composition than

samples taken at the beginning of work (T1) (pairwise R^2=22.0%; FDR adjusted p=0.004); and at the end of the workday following showering (T3) the microbiome underwent yet another shift relative to T2 (pairwise R^2=18.6%; FDR adjusted p=0.004). However, the microbiome at T3 was not significantly different than at T1, suggesting at least a partial reversion of the skin microbial composition after showering (pairwise R^2=4.9%; FDR adjusted p=0.12). These shifts corresponded with changes in skin-borne bacterial load as quantified using 16S gene concentration (copies/µl, **Figure 2b**). Specifically, 16S gene concentration increased by ~200-fold from T1 to T2, but then decreased back to baseline levels at T3 (Type III ANOVA p< 0.0001 with Tukey's *post hoc* analysis and adjustment for FDR). There were no significant differences in average sequencing depth, sequencing quality and taxonomic discovery rates between the three collection time points, suggesting that these technical factors did not significantly bias comparisons across collection phases (**Supplementary note 2**, **Supplementary figures 1, 2**).

We next assessed associations between crude swine exposure rates and 16S gene concentration, i.e., bacterial load. There was large variation in estimated hourly swine exposure rates (**Supplementary table 1**), and the hourly exposure density was found to be inversely correlated with bacterial load (Estimate[SE]= -1.23[0.44], *glm* p=0.01, **Figure 2c**). This relationship persisted at T2 and T3 (**Figure 2c**). Though more granular and systematic exposure assessments are needed, these patterns suggest that differences in swine exposure density may be a proxy for different workday tasks that ultimately dictate levels of microbial biomass acquisition. For example, tasks such as feeding, health-checks, and decontamination require walking through swine holding rooms, but involve very little direct interfacing with animals and their byproducts; such tasks would be classified as "high density", but in reality there may be less opportunity for direct acquisition of swine-related microbes. In contrast, activities such as vaccinating and obstetrical management require prolonged contact with individual pigs, but not necessarily moving through multiple swine holding rooms; thus, such tasks may have lower density but more opportunities for acquisition of swine-sourced microbes through direct contact.

A total of 6,840 unique amplicon sequence variants (ASVs) representing 356 distinct genera were recovered across all worker skin samples (**Supplemental figure 2, Supplementary datafile 2,3**). The ASV diversity and the dominant phyla were consistent with findings from previous microbiome studies of human skin [21–23]. The relative abundances of dominant phyla remained largely similar across T1–T3, with the exception of Cyanobacteriota, which were more abundant on skin at T2 versus T1 and T3 (**Supplemental figure 3a-b**). Phylum-level and genus-level richness and evenness of the worker skin microbiome remained unchanged throughout the course of the day, and did not significantly differ from contact-matched swine skin samples nor the environmental samples (Type III ANOVA adjusted p> 0.1, **Supplemental figure 4a-d**). More than 400 genera were detected in at least one sample from each of the pairwise collection phases under comparison (T1 vs. T2 [n=76], T2 vs. T3 [n= 76], and T1 vs. T3 [n=75]), and fewer than 13% of these genera exhibited significant changes in relative abundance over the three timepoints (**Supplementary datafile 4**, **Supplementary figure 5**). The relative abundances of *Methanobrevibacter, Negativibacillus, Butyricicoccus, Agathobacter*, and *Lachnospiraceae UCG-010* were significantly higher in T2 versus T1 samples. These genera inhabit mammalian oral cavity and digestive tracts. Genera with significantly higher relative abundance in T3 compared to T2 skin samples included taxa also primarily found as gastrointestinal microbiota.

These include relatively new and unclassified rumenal genera such as *UCG-005(Oscillospiraceae) and Candidatus Soleaferrea (Ruminococcaceae)* that have been previously reported in swine intestinal microbiomes but not functionally described [24,25], and *CHKCI001 (Lachnospiraceae)* which to our knowledge has hitherto been only described in chicken intestinal microbiomes [26,27]. Other differentially abundant genera at T3 relative to T2 include *Agathobacter* which exhibited the largest fold-change increase in relative abundance, and was also found at a significantly greater relative abundance at T2 vs T1 (**Supplementary datafile 4**). *Agathobacter* is a fiber-degrading and butyrate producing keystone genus that has been described in growing piglets [28,29]. We noted that a mixed population of genera were differentially abundant at T3 relative to T1, including intestinal genera such as *Negativibacillus* which accounted for the greatest fold-change at T2 relative to T1, *Fastidiosipila*, a poorly characterized genus of methanogenic anaerobic bacteria [30], and highly ubiquitous environmental bacteria Brevundimonas.

The skin microbiome becomes unstructured and dominated by enteric and environmental microbes during on-farm work

Inferred association networks were explored using a compositional modeling approach to describe the topology and connectivity of microbial constituents in worker skin microbiomes across collection phases and in comparison to contact-matched animals (SI Appendix, SI Methods). Networks were constructed using standard cutoffs, and ASVs with >100 counts present in >10% sample prevalence were included, which represented 4.1–5.7% of all ASVs used as input into network generation, depending on the collection phase. The resulting networks (one per collection phase) were each composed of a singular interconnected component, with the most connected network being T1 and the most sparse T2 (**Figure 2d**).

Given the significant differences in network topology across T1, T2 and T3 (**Supplementary note 4**), we further analyzed each network to identify the most dominant and interconnected genera (i.e., keystone taxa), as indicated by high eigenvector centrality and high node degree. At T1, keystone genera included a mixed population of bacteria not typically considered ubiquitous inhabitants of the human skin microbiome [31], including usually enteric members such as *Clostridium* and *Mediterraneibacter* and environmental bacteria such as *Blastococcus* and *Cryobacterium*. Other keystone genera included human skin commensals like *Anaerococcus* and *Fusobacterium*. However, *Corynebacterium*, well-known as a ubiquitous inhabitant of both swine and human skin, was among the least influential members of the skin microbiome (**Figure 2e**). Conversely, at T2, the interactions and modular domains within the skin microbiota became notably sparse, with *Corynebacterium* genera by far the most extensively represented among the keystone bacteria [21,32,33] (**Figure 2e**). The least influential bacteria at T2 were typically enteric bacteria characteristic of mammalian intestinal microbiota, including *Clostridium*, *Rothia*, *Kocuria*, and *Terrisporobacter*. After showering and prior to exit from the swine facility (T3), dominant keystone genera were an admixture of Actinobacteria and Clostridia classes, encompassing genera associated with the mammalian gastrointestinal tract including *Sellimonas*, *Rothia*, *Mediterraneibacter*, UCG-005; as well as commensal human genera such as *Peptoniphilus* and Family XI of the Peptostreptococcales-Tissierellales Order, previously identified among medically important inhabitants of human axillae [34]. We note that the swine

skin ecological network was composed of dominant keystone genera *Fusobacterium*, *Schaalia*, and *Akkermansia*, typically associated with skin, oral mucous membrane, and intestinal microbiota. Notably, *Fusobacterium* in swine corresponded with the same genus found among keystone organisms at T1 in swine workers collected prior to entry into the main animal holding areas, while the least interconnected swine skin genera of *Corynebacterium* were observed in worker skin ecosystem dominance at T2, collected at the end of the workday.

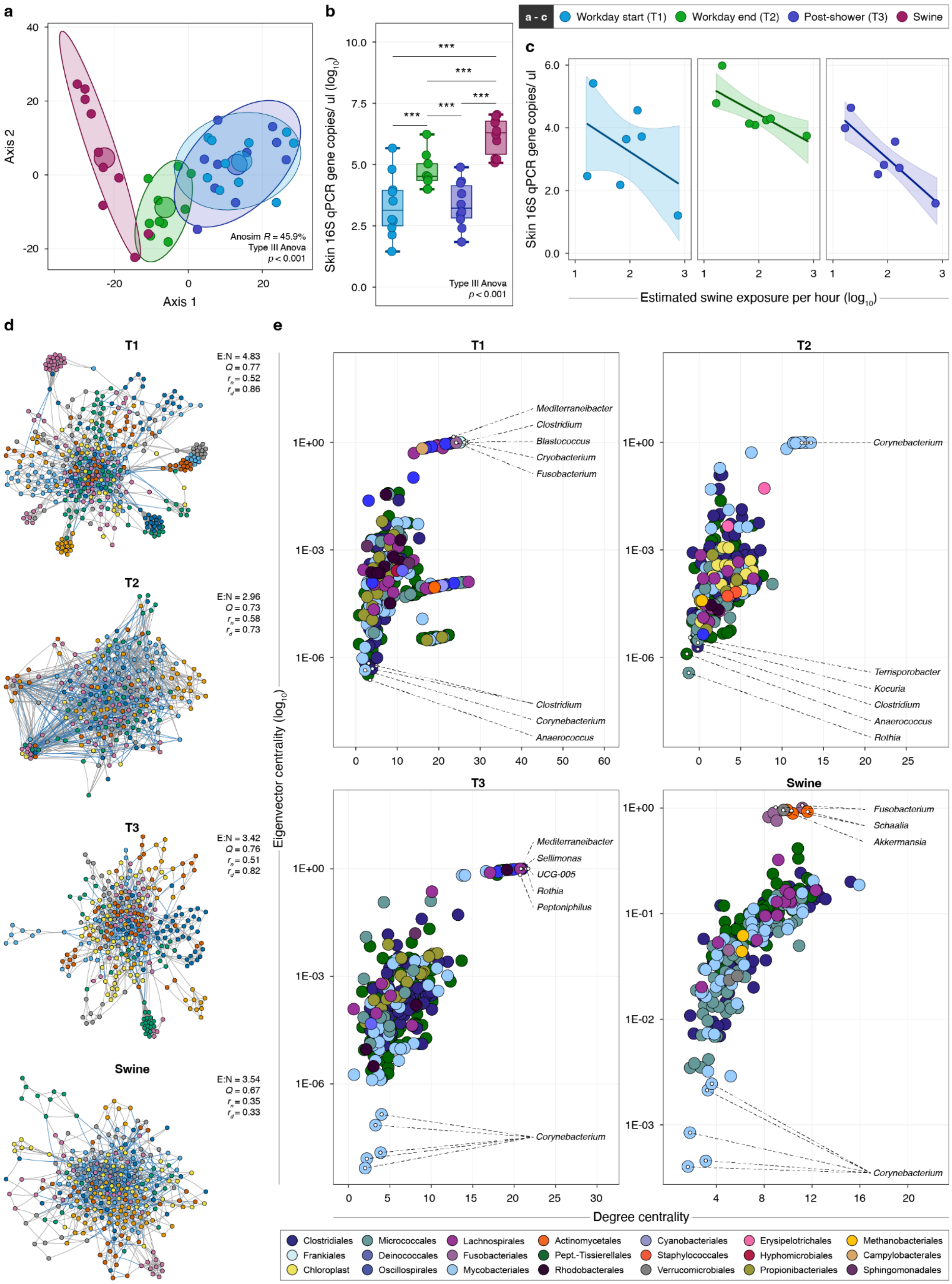
a
Axis 1
Axis 2
Anosim R = 45.9%
Type III Anova
p < 0.001
b
Skin 16S qPCR gene copies/ ul (log10)
***
Type III Anova
p < 0.001
a - c
Workday start (T1)
Workday end (T2)
Post-shower (T3)
Swine
c
Skin 16S qPCR gene copies/ ul
Estimated swine exposure per hour (log10)
d
T1
E:N = 4.83
Q = 0.77
r_n = 0.52
r_d = 0.86
T2
E:N = 2.96
Q = 0.73
r_n = 0.58
r_d = 0.73
T3
E:N = 3.42
Q = 0.76
r_n = 0.51
r_d = 0.82
Swine
E:N = 3.54
Q = 0.67
r_n = 0.35
r_d = 0.33
e
Eigenvector centrality (log10)
Degree centrality
Mediterraneibacter
Clostridium
Blastococcus
Cryobacterium
Fusobacterium
Corynebacterium
Anaerococcus
Terrisporobacter
Kocuria
Rothia
Sellimonas
UCG-005
Peptoniphilus
Schaalia
Akkermansia
Clostridiales
Micrococcales
Lachnospirales
Actinomycetales
Cyanobacteriales
Erysipelotrichales
Methanobacteriales
Frankiales
Deinococcales
Fusobacteriales
Pept.-Tissierellales
Staphylococcales
Hyphomicrobiales
Campylobacterales
Chloroplast
Oscillospirales
Mycobacteriales
Rhodobacterales
Verrucomicrobiales
Propionibacteriales
Sphingomonadales

238
239

Figure 2. Changes in skin microbial load, microbiome composition, and community structure. a. Genus-level worker skin microbiome ***β***-diversity across collection phases (T1–T3) and contact-matched swine skin samples using principal component ordination of robust Aitchison compositions. Within-group centroids and 95% confidence intervals are depicted with a large circle and shaded ellipsoids, respectively. **b.** Log_{10}-normalized 16S rRNA qPCR copy number / µl (y-axis), stratified by collection phase (x-axis). *** indicates statistical significance (p<0.001) of pairwise comparisons based on a linear regression model with Tukey's adjustment for multiple comparisons. **c.** General linear analysis of the log_{10}-normalized 16S rRNA qPCR copy number / µl (y-axis) and log_{10}-normalized hourly exposure to swine (x-axis) based on workers' estimates from daily task assignments indicates a negative correlation (p= 0.01) across all collection phases. Shaded areas represent the 95% confidence interval around the linear trendline. **d.** Worker skin microbiome networks across workday collection phases and for contact-matched swine were inferred from inverse covariance estimation for compositions based on centered-log ratios of subsetted ASV counts containing >100 counts and >10% prevalence per ASV per sample. Inferred networks consist of nodes representing ASVs colored by shared subcommunity membership. Edges between nodes represent a significant predicted positive (blue) or negative (gray) interaction. Reported topology characteristics include network connectivity based on the edge to node ratio (*E:N*), modularity (*Q*), subcommunity assortativity (r_n), and degree assortativity (r_d). **e**. Scatter plots of microbial constituents from the corresponding networks are displayed based on the log_{10}-normalized node eigenvector centrality (y-axis) and node degree centrality (x-axis). Taxa with the highest centrality measures (top right of the distribution) are considered to be critical connectors and major hubs in community networks, and thus putative keystone taxa. ASV-level nodes are colored based on their taxonomic classification at the Class level. Genus-level labels are displayed only for genera most likely to be keystone, i.e., >95th percentile of the plot distribution (top right) and least likely to be keystone, i.e.<5th percentile of the plot distribution (bottom left).

The worker skin resistome and mobilome shifted significantly during the workday, and differed from that of contact-matched swine

Target enrichment was used to selectively capture and amplify all potential known ARGs and MGEs within the metagenomic DNA of all samples [35]. As with the microbiome, the resistome shifted significantly between each collection phase (ANOSIM p< 0.0001; R=37.5%, **Supplementary figure 6a**), but there were no statistically significant differences in ARG group richness or Shannon's diversity across the three collection phases (**Supplementary figure 7**). However, when normalized to the bacterial load as measured by the 16S rRNA gene copy number, the total ARG burden was significantly altered over the course of the work day. While bacterial load increased at the end of the workshift (T2) vs the start (**Figure 2b**), we highlight in **Supplementary figure 8a** that workers at T1 and T3 carried a significantly greater total ARG abundance than at T2, though it was also observed that showering led to a significant reduction of total skin ARGs relative to T1 (Type III ANOVA FDR-adjusted p< 0.001 with Tukey's *post hoc* analysis). The T1 worker skin also harbored a greater ARG burden than the skin of pigs (Type III ANOVA FDR-adjusted p=0.005) and the total ARG burden in the environment did not significantly differ from that of any worker or swine skin sample. Similarly, plasmids, ICE, IS, and TE underwent significant shifts in ***β***-diversity. Collection phase accounted for >40% of the variation in composition of ICE and TE, >20% of variation in composition of plasmids and IS, and ~9% of variation in composition of viruses and prophages (Plasmids, ICE, TE, and IS ANOSIM p<0.001; viruses and prophage ANOSIM p=0.02 **Supplementary figure 6b**). Plasmids, ICE, IS and TE compositions were significantly different between T1 and T2, and between T2 and T3 (all PERMANOVA p<0.001), while virus and prophage composition differed between the collection phases T1 and T2 (PERMANOVA p<0.05). The observed MGE compositional shifts at T2 coincided with a greater relative abundance of ICE genes and a reduced relative abundance of plasmids, including plasmidic mechanisms of replication, transcription, translation, and regulation (**Supplementary figure 6c-d**). Between T1 and T3, there were significant differences in composition of IS and TE genes (PERMANOVA p=0.048 and 0.037, respectively), but not plasmids and ICE. The worker resistome and MGE composition

were significantly different from swine at all three collection phases, with the exception of viral and prophages at T2 (**Supplementary figure 6b**). Regarding the mobilome, the total MGE burden on the skin normalized to bacterial load (**Supplementary figure 8b**), was significantly higher at T1 and T3 compared to T2 (Type III ANOVA, FDR-adjusted $p< 0.0001$, Tukey's *post hoc* analysis). While the total MGE abundance on swine skin did not differ significantly from worker skin at T1 and T2, worker skin at T3 (after showering) harbored a greater number of MGE alleles than swine skin (Type III ANOVA, FDR-adjusted $p = 0.0268$, Tukey's *post hoc* analysis). In contrast to the resistome samples, environmental samples contained the highest abundance of MGEs observed in the study (Type III ANOVA, FDR-adjusted $p < 0.001$, Tukey's *post hoc* analysis).

The clinically important fraction of the worker skin resistome varied throughout the workday and remained distinct from swine

We subsetted the MEGARes v2.0 database for 29 specific ARG groups previously identified as 'clinically important' (i.e., priority ARGs) [36,37]. In 41/42 enriched metagenomic samples, we detected 19 distinct priority ARG groups at gene coverage fraction >99.9%; one T2 sample did not contain any priority ARGs. These ARGs represented a low proportion of the total resistome across all worker (median[IQR]= 7.46%[8.23]) and swine samples (median[IQR]= 5.57%[4.27]), and their overall median relative abundance did not differ between workday collection phases and swine samples (Type III ANOVA $p> 0.05$).

Tetracycline (*TetM*), sulfonamide (*Sul1*), multi-drug resistance to classes of antibiotics including lincosamides, streptogramins, and pleuromutilins (*Vga*), and methicillin (*mecA*) genes were the most prevalent and abundant of the priority ARGs (**Figure 3)**, and strongly influenced hierarchical clustering of samples into four major groups (i.e. subclades). Subclade 1 was characterized by high *TetM* relative abundance and contained eight of the 10 swine samples and one or two worker samples from each of T1-T3. Subclade 2 contained five of the 9 T2 samples, one swine sample, one T1 and two T3 samples, and was characterized by a higher relative abundance of *Sul1*. Subclades 3 and 4 contained the majority of the T1 and T3 samples (i.e., 14/20), with subclade 3 defined by a higher abundance of *Vga* and subclade 4 containing the highest relative abundance of *mecA*.

The *mecA* gene, a methicillin resistance allele, consistently appeared in worker but rarely in swine samples (**Figure 3**). *Staphylococcus aureus* in swine has been proposed as a key source of methicillin resistant *S. aureus* (MRSA) in Danish swine workers [38], especially among workers of Danish pig herds in which historical MRSA prevalence exceeds 85%. However, recent reports suggest that *Staphylococcus* spp. are actually rare members of the porcine skin microbiome, and typically account for <1% of the overall relative abundance of all Staphylococci [33]. We performed marker-based strain-level taxonomic profiling of metagenomic reads via StrainPhlAn to ascertain possible Staphylococcal sources of *mecA*. Strains of *S. epiderimidis*, *S. haemolyticus*, *S. hominis*, and *S. equorum* were the only prevalent strains identified (>75% prevalence), and no *S. aureus* strains were identified at this pre-specified prevalence level (**Supplementary datafile 9**, **Supplementary figure 9**). Further phylogenetic analysis suggested that these Staphylococci were rarely shared between workers and swine, as most strains were tightly clustered by worker

ID rather than collection phase (**Supplementary figure 10**). Major coagulase negative Staphylococci (CoNS) are known carriers of *mecA*, and ~90% of U.S. *Staphylococcus epidermidis* clinical isolates in particular are methicillin resistant [39–41]. Taken together, these findings suggest that worker CoNS and not *S. aureus* were likely sources of *mecA* in this study. ~~Interestingly, no major Staphylococcal strains were detected at T2. This could be due to the increased microbial biomass in T2 samples (**Figure 2b**), which may have reduced the relative abundance of *Staphylococcus* sequences within the extracted DNA and resulting metagenomic data, effectively pushing *Staphylococcus* under the limit of detection needed for robust StrainPhlan analysis.~~

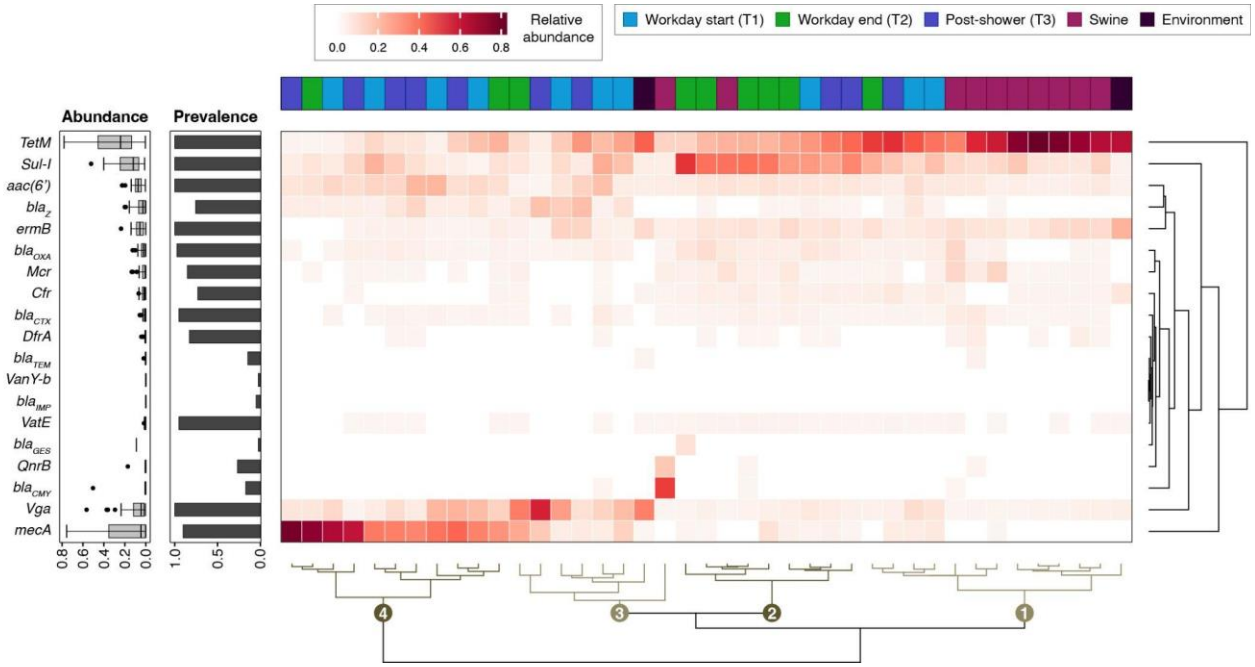

Figure 3. Occurrence of medically important ARGs on the skin of workers and swine. Unique medically important (i.e., priority) ARG alleles were identified at >99.9% alignment gene coverage across all collection phases (top ribbon annotation) and are displayed using a heatmap summarizing their sample-level relative abundance across each of the respective 19 ARG gene groups. The cladograms along the x-axis demonstrate the hierarchical clustering of samples according to their medically important resistome composition using optimal leaf sorting and euclidean distances. Four major subclades are colored and numbered. Major ARG group prevalence and median abundance across study samples are summarized via the associated barplots and boxplots along the y-axis.

Post-work showering incompletely reverses changes in resistome and mobilome gene abundance

After controlling for worker age, gender, BMI, smoking status, frequency of pork consumption, as well as host-removed sequencing depth, only 5.4%, 3.7%, and 2.7% of ARG groups exhibited significant changes in relative abundance at T1 vs. T2, T2 vs. T3, and T1 vs. T3, respectively. Between T1 and T2 collection phases, the vast majority of significantly changing ARG groups exhibited increases in abundance (i.e., 27/29 ARG groups, 93%) (**Figure 4a, Supplementary datafile 10**). Because log-fold differential abundance testing can produce false positives for low-count features, we highlighted only high-abundance ARG groups with a statistically significant change in relative abundance. For the T1 versus T2 comparison, this included ARG groups

within the β-lactams (e.g., *mecA*), fosfomycins (e.g., *fosA* and *fosB*), and mupirocins (e.g., *mupA*). In contrast to the T1-T2 comparison, far fewer ARG groups experienced statistically significant changes in relative abundance from T2 to T3 (n=18), and most of these (i.e., n=13, or 72%) decreased in relative abundance, including β-lactams (e.g., *bla$_{GES}$*), fusidic acids (e.g., *fusB*), phenicols (e.g., *cmlA*), sulfonamides (e.g., *sulIV*), MLS (e.g., *ereA*), and multi-drug or multi-compound classes (e.g., *fexA, ttgB, mexW, lmrD*). Two abundant ARG groups that exhibited significant increases in relative abundance at T2 compared to T1 also remained elevated after showering in T3, most notably *mecA*, and *norA*, the general drug and biocide efflux system of Staphylococci [42]. When compared to T1, 13 ARG groups at T3 were significantly differentially abundant, and 10 of these (77%) exhibited a significant decrease in relative abundance (**Figure 4a**). For example, among the most abundant T3 ARGs, there was a significant decrease in multi-compound and fusidic acid resistance (e.g., *fexA*, *mepA*, *fusB*), and multi-drug resistance regulators and efflux systems (**Supplementary datafile 10**).

Among MGEs with significant changes in relative abundance between collection phases, ICE were most prominent (**Figure 4b**). Specifically, *ICEPaeLESB58-1*, *ICETn4371* and *ICESsu(BM407)* were more abundant in T2 versus T1 samples; the first two mobilize heavy metal resistance [43], while the latter mobilizes ARGs narrowly within *Streptococcus suis*, an emergent pathogen in humans that is considered a host-adapted swine pathobiont [43,44]. Further strain analysis confirmed presence of *S. suis* in all swine and 9/10 of worker samples in each collection phase with a mean relative abundance of 10.2% at T1, 26.1% at T2, and 12.8% at T3 (**Supplementary datafile 9**). Compared to T1, T2 samples also contained significantly higher relative abundance of replication and recombination machinery of the host-adapted *Staphylococcus epidermidis* bacteriophage (e.g., helicase loader and replication helicases and Holliday junction resolvases), as well as *IS6* sequences associated with methicillin resistance (*IS431mec*), erythromycin resistance (*IS257-1*), and transposable components of *IS6*/*IS26* and *TnAS3* involved in mobilizable resistance [44] at human-animal interface contexts.

Following showering, the most abundant ICE module *ICETn6087* was reduced in relative abundance compared to T2; however, the next 9 most-prevalent MGEs increased in relative abundance at T3 versus T2 (**Figure 4b**), notably *IS431mec* which already exhibited a significant increase from T1 to T2. Other significantly more abundant MGEs in T3 versus T2 samples included plasmids of *Staphylococcus epidermidis* (*pSepCH*, *SE_p410*), *Staphylococcus aureus* (repV: *pT181*; pgi: *pSJH901*); as well as *Staphylococcus epidermidis* and *Bacillus cereus* bacteriophages and prophages. *S. suis*-adapted *ICEPaeLESB58-1* and *ICETn4371* were significantly more abundant at T3 versus T1; while promiscuous tetracycline-associated Tn916-like ICE *ICETn6085a*, *ICETn6085b*, and *ICETn6084* were significantly less abundant in T3 compared to T1 [45] (**Supplementary datafile 11**).

Worker skin at T2 (i.e., following work with swine) had a higher MGE abundance than contact-matched swine (**Figure 4b**), dominated by mucous membrane, respiratory tract, and enterically adapted ICE. Prominent among these were Streptococcal RD2 element (10750-RD.2), *ICETn1806*, as well as *ICESauJKD6008* and *ICECTn4* known to mobilize vancomycin and tetracycline resistance in *Staphylococcus aureus*, Enterococci, and *Clostridioides difficile* [46–49]. *Staphylococcus aureus* and *Escherichia coli* plasmid replicon modules were also in higher abundance in T2 worker versus swine skin (e.g., *repUS12_pUB110*,

repUS23._repA(SAP099B)_GQ900449.1, and *IncY_1__K02380*). Additionally, worker skin contained a higher relative abundance of *Psychrobacter*-associated plasmid *pRWF101_PsycPRwf*. *Psychrobacter* species were identified as the most abundant member of the swine skin microbiota in strain analysis (**Figure 2e**), especially *P. pasteurii* and *P. piechaudii*, whereas strains of *P. faecalis* and *P. maritums* were detected in nearly all human, swine, and environmental samples. The detection of *psychrobacter*-associated MGE alleles in human samples was, however, unexpected. Historically, *Psychrobacter* isolates have been obtained from arctic, marine, sediment, and limited terrestrial environments [50]. However, recently this genus has been detected in pig slurries, manure, and swine carcass processing facilities [51,52] and *Psychrobacter* spp. have been identified as dominant microbes within the nares of workers involved in swine transport [53].

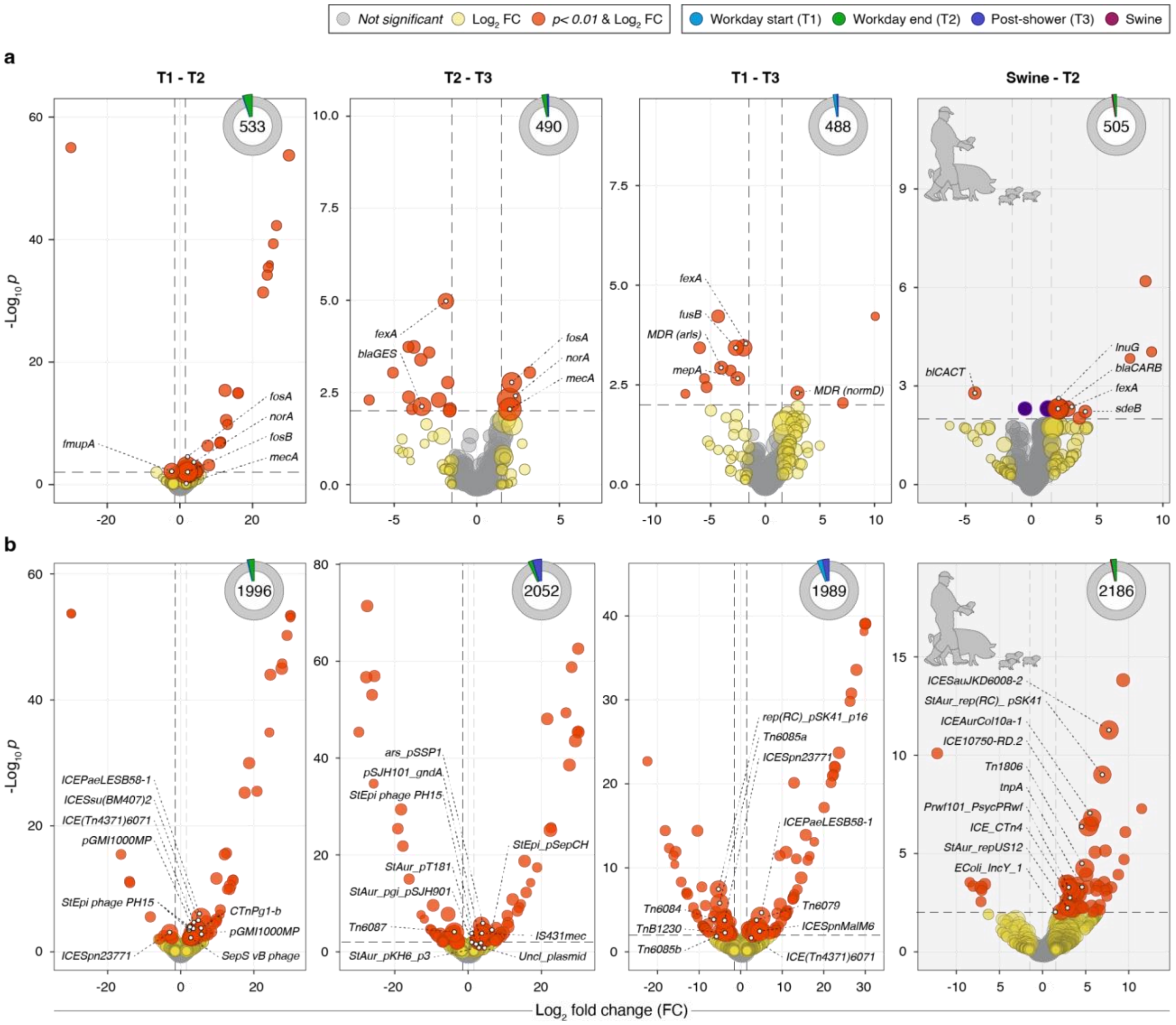

Figure 4. Differential abundance analysis across collection phases and at the interface period between workers and pigs. Volcano plots are used to visualize differential abundance of unique **a** ARG groups and **b** MGE accessions in log_2-fold change (x-axis) and -$log_{10}P$ value (y-axis) of the global worker skin resistome or mobilome between key workshift collection phases: Workday start (**T1**) vs. workday end (**T2**); Workday end (**T2**) vs. post-shower (**T3**); and Workday start (**T1**) vs. post-shower (**T3**). An additional comparison is made between workday end and swine skin samples representing the worker's contact phase with animals (gray). Features with significant shift in abundance (Wald's $p<0.01$ with FDR adjustment for ARG group and MGE accessions) are displayed above the horizontal line, while biologically significant fold-change is demarcated by vertical dashed lines at 1.5 log_2-fold change. Labels are displayed for only the 5 most abundant ARG groups and 10 most abundant MGEs significantly amplified (log_2-fold change >1.5 or < -1.5) at each phase comparison. For each volcano plot, an associated pie chart displays the number of unique ARG groups and MGE accessions common to each of the workshift collection phases compared, as well as the proportion of the total differentially abundant MGEs associated with each phase.

MAGs recovered from worker and swine skin samples represent putatively novel strains

High quality metagenome-assembled genomes (MAGs) were constructed via *de novo* genome assembly for all individual samples and also as co-assemblies of samples within T1, T2, T3, swine and environmental (**Figure 5a–c, Supplementary datafile 12**). The *Bacillota* phylum was by far predominant across all genomes (n= 139), followed by *Actinobacteriota* (n=44), *Bacteroidota* (n=17), and *Pseudomonadota* (syn. *Proteobacteria)* (n=9). The distribution of the most abundant phyla across all MAGs was consistent with the phyla detected by 16S microbiome sequencing. A large proportion of identified MAGs had poor taxonomic representation among known Genome Taxonomy Database (GTDB) MAGs, as 47 (22%) were classified as putatively novel species (i.e., <95% ANI with a known sequenced genome in GTDB), and 167 (78%) were identified as putatively novel strains (i.e., <99% ANI with a known sequenced genome in GTDB).

Approximately 60% (28/47) of all MAGs considered to be putatively novel species were recovered from swine skin samples, even though swine samples represented <25% of analyzed samples (i.e., 10/42). Swine samples also accounted for ~53% (89/167) of the MAGs identified as putatively novel strains. We detected new strains that were highly abundant in recent swine intestinal MAG catalogs [54] and that we also identified either via 16S or strain gene-marker analysis in this study, including *Psychrobacter* (*P. pasteurii*), *Streptococcus* (*S. hyovaginalis, S. pluranimalium, S. dysgalactiae*), *Corynebacterium* (*C. xerosis, C. variabile, C. glutamicum, C. pollutisoli, C. stationis*), and *Lachnospiraceae.*

Worker skin samples accounted for ~45% of the de-replicated MAGs, retrieved predominantly from co-assembly (n=62) vs. individual (n=33) approaches. Nearly 75% (71/95) of MAGs recovered from human samples represented either novel species or strains. These novel taxa comprise 9 genera, 6 of which are known to be natural inhabitants of environmental matrices, including *Microbacterium*, *Marihabitans*, *Marmoricola*, *Chloroflexi* bacterium, *Qipengyuania*, and *Tsuneonella*. Samples representing the swine farm exposure phases (i.e., T2 and T3) accounted for 76% of the total putatively novel strains detected in worker microbiomes (T2: 28/95; T3: 26/95). Though samples from workday start (T1) accounted for the smallest proportion of all recovered human MAGs (23%), we nevertheless captured major expected cutaneous taxa as documented in previous strain-resolved MAG workflows [55,56], including *Staphylococci* (e.g. *S. hominis, S. epidermidis, S. capitis*), *Corynebacterium* (e.g. *C. xerosis, C. mucifaciens, C. kefirresidentii*), *Cutibacterium* (e.g. *C. granulosum* and *C. acnes*), and

Lactobacillaceae (e.g. *Lactobacillus amylovorus, Latilactobacillus sakei*, *Limosilactobacillus reuteri*).

In addition to the genera observed at T1, MAGs from T2 samples also included 6 genera of the Clostridial co-abundance gene group 138 (i.e., CAG-138) previously linked with critical functions for fiber degradation in the swine enteric system [57]. Genera not assigned with NCBI taxonomic nomenclature from Lachnospiraceae, Butyricicoccaceae, Oscillospiraceae, and Treponemataceae were also recovered, and their identities were concordant with best-matched NCBI genomes sequenced from fecal samples of piglets <30 days old. Among MAGs recovered from T3, ~55% (20/36) included species identical to those observed in both T1 and T2 samples. However, T3 MAGs also included species that were only observed at T2 (and not at T1), including taxa typically identified in livestock such as *Streptococcus alactolyticus* known as part of the *Streptococcus bovis*/*Streptococcus equinus* complex (SBSEC), *Aerococcus urinaeequi*, as well as uncharacterized MAGs previously identified in swine fecal samples (GenBank ID: *GCA_016293975.1*, *GCA_004558825.1*, *GCA_004556755.1*) [58]. Though minor human skin commensals were exclusively detected in T3 samples, such as *Lawsonella clevelandensis* and *Corynebacterium aurimucosum*, genera previously isolated from environmental matrices were also exclusively recovered in T3 samples, including *Tsuneonella* sp., *Qipengyuania* sp., *Marmoricola* sp., and *Chloroflexi* bacterium UBA6265.

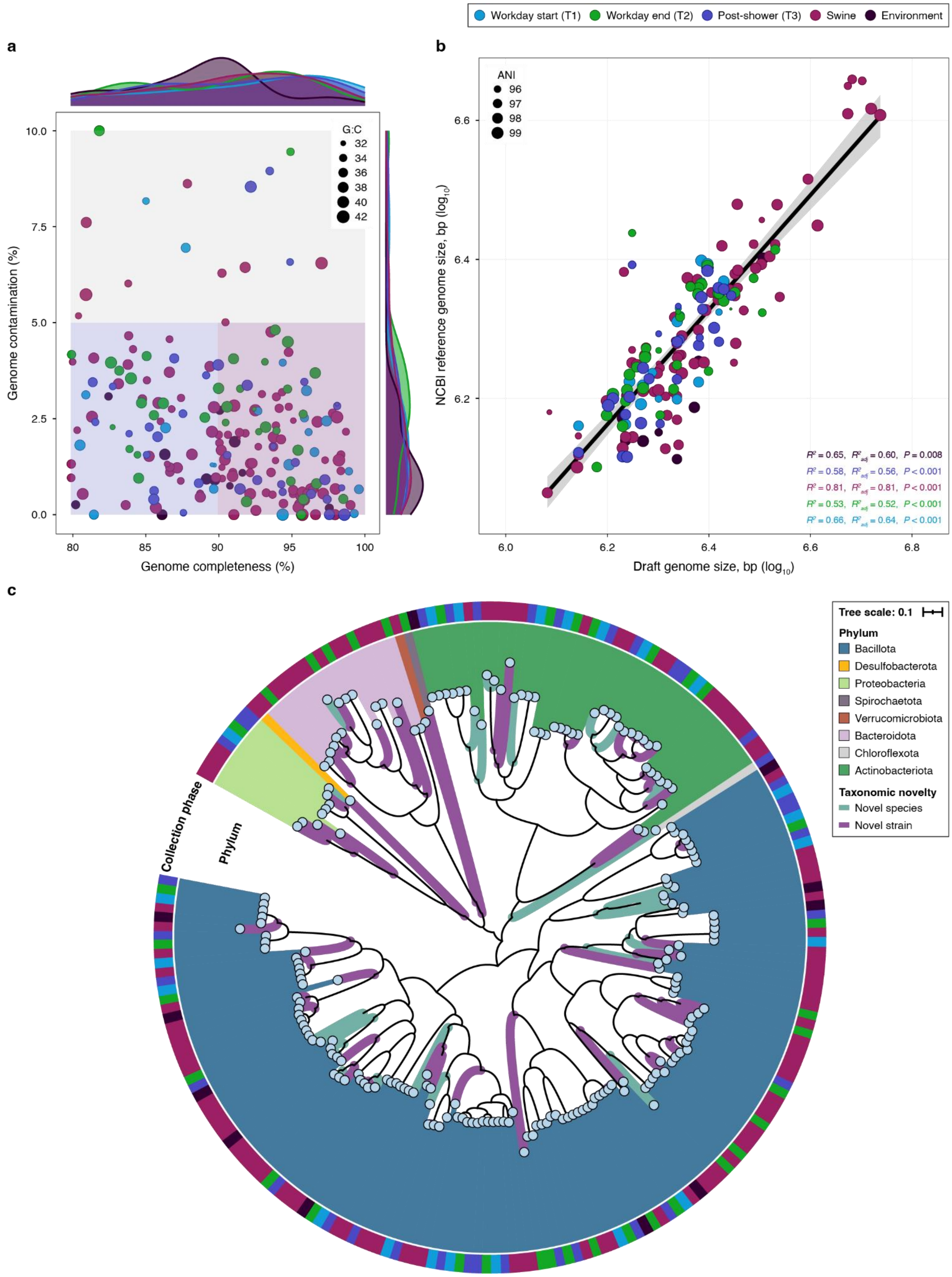
Workday start (T1)
Workday end (T2)
Post-shower (T3)
Swine
Environment
a
G:C
32
34
36
38
40
42
Genome contamination (%)
Genome completeness (%)
b
ANI
96
97
98
99
NCBI reference genome size, bp (log10)
Draft genome size, bp (log10)
R² = 0.65, R²adj = 0.60, P = 0.008
R² = 0.58, R²adj = 0.56, P < 0.001
R² = 0.81, R²adj = 0.81, P < 0.001
R² = 0.53, R²adj = 0.52, P < 0.001
R² = 0.66, R²adj = 0.64, P < 0.001
c
Tree scale: 0.1
Phylum
Bacillota
Desulfobacterota
Proteobacteria
Spirochaetota
Verrucomicrobiota
Bacteroidota
Chloroflexota
Actinobacteriota
Taxonomic novelty
Novel species
Novel strain
Collection phase
Phylum

Figure 5. Taxonomic diversity and novelty of resolved metagenomic assembled genomes (MAGs) recovered from the human-swine interface and ambient environment. a Scatter plot of points representing individual GTDB taxonomically binned MAGs according to their estimated percent completeness (x-axis) relative to their estimated percent contamination (y-axis). Point size is proportional to the quantified MAG GC-content. Pink, purple, and gray regions of the plot demarcate the density of 'high quality', 'medium quality-low contamination', and 'medium quality' MAGs retrieved across all collection phases. **b** Scatter plot of estimated MAG size is displayed based on the log_{10}-normalized nucleotide count (x-axis) and the nearest assigned NCBI reference genome size expressed in log_{10}-normalized nucleotide count (y-axis). A global regression line and 95% confidence interval (shaded region) is displayed and results of significance testing (R^2; p<0.05) using a generalized linear model are colored for MAGs recovered for each collection phase. **c** Phylogenomic tree of de-replicated and high-confidence MAGs recovered from target-enriched metagenomes across each collection phase (outer ring). The area below each leaf is colored according to the taxonomically assigned Phylum. Branches for proposed novel species (ANI <95%) are displayed in teal, and for proposed novel strains (ANI <99%) are displayed in purple.

DISCUSSION

Environmental exposure histories play a determinative role in shaping adult microbiomes, even more so than individual-level variables [59–61]. Cutaneous microbiota are recognized for their remarkable stability in the face of environmental perturbation over short time scales [62–64]. Despite this, we demonstrate that swine worker skin experiences a significant increase in bacterial load and a significant shift in microbiota composition during a single 8-hour workday. However, showering at the end of work seems to dampen these changes, indicating that biosecurity interventions not only reduce worker-borne swine pathogen transmission [65,66], but also work-associated microbiome impacts. We likewise demonstrate that accumulation of ARGs on the skin can be counteracted with showering, suggesting that such biosecurity practices could be important public health measures to reduce the bidirectional flow of resistant bacteria between animal-associated workplaces and the general community [67–69]. Our work highlights that showering may have a variable impact on other aspects of the microbial metagenome, such as mobile genetic elements which were in some cases enriched even after showering at the end of the workday. Effects of farm protocols and biosecurity have not been expressly evaluated in recent investigations of 'shareable' microbial features between humans, animals, and the farm environment [5,53,70–73]. Our results suggest that a more nuanced understanding of these practices on different components of the metagenome is warranted, particularly given the different intra-shift dynamics we observed across the resistome, mobilome and microbiome. Future studies should include detailed characterization or even measurement of process controls, including the use of biosecurity practices for decontamination and exposure reduction via personal protective equipment (PPE), occupational training and monitoring, and environmental management.

Within-farm sources of microbes that shape worker microbiomes remain unknown, and there are no systematic, established methods for conducting microbiome-based surveillance in occupational health contexts, and particularly for commercial farm work. The striking proportion of possibly novel species that we recovered from the skin of both swine and workers suggests that skin may be an important yet under-represented sampling target for on-farm occupational health research (**Figure 5**). We focused our study on the skin for several reasons, including ease of sampling and a high proportion of skin-associated diseases within livestock workers; additionally, the skin surface is continuously exposed to the farm environment and thus likely to serve as a competent catchment for air-borne bacteria. Bacteria from livestock feces, soil, and water are relevant sources of exposure that may induce shifts in human microbiomes. For example, swine farms and especially swine feces can impact the antibiotic resistome of

individuals living in proximity to the farm [74]. However, emerging evidence suggests that air and dust should not be overlooked as important sources of microbiome and resistome richness [75–77]. Farm dust has also been shown to be protective against asthma [77], and together, these studies underpin the need to further investigate the skin-environment interface. Though our study was not expressly designed for robust source-attribution of the microbes, ARGs, and MGEs in worker skin, future work could conduct more precise analyses of the bacteria and their genetic features. Such detail could inform methods to control or reduce bi-directional exchange of bacteria along the human-animal-environment continuum. To achieve this, more thorough sampling of diverse environmental matrices would be needed, and microbiomes of workers should be studied with more precise measures of exposures to air, soil, feed, dust, feces, and animals when performing a range of tasks over the course of the workday.

One major question is whether work-acquired microbes become incorporated into the cutaneous microbiome as long-term, stable members of the community. Our study design did not include long-term follow-up, and thus we could not quantify the proportion of taxa that become *de facto* colonizers following repeated workday exposures. However, we did demonstrate that showering seemed to literally wash away many of the microbes, ARGs, and MGEs that accumulated on the skin during the workday. This may be due to the fact that bacteria acquired during the workday become only weakly adherent to the skin and thus are easily washed away. The impact of showering is even more robust when one considers the heterogeneity in showering practices, as workers in this study were told to shower as they normally would at the end of their workday, including use of their own preferred soaps and other personal care products. However, it is important to note that showering did not completely eliminate newly-acquired taxa, and workers may continue to harbor microbes from enteric and environmental taxa that are characteristic of the swine farm context, as shown in our 16S rRNA and MAG results (**Figures 2** and **5**), and reported in a previous study of swine farm workers who resided in a Chinese swine farm for ~3 months [5]. In this study, we focused on showering as a key biosecurity intervention to reduce the theoretical carryover of farm microbiota, ARGs, and MGEs. However, swine workers followed multiple biosecurity practices throughout their workday, which may have influenced the microbiome composition at T2 and ultimately at T3. The effects of additional protective measures—such as wearing coveralls, boots, gloves, handwashing, and sanitizer use—could not be isolated from the impacts of swine farm exposure at T2, as these practices were either mandatory or encouraged for all workers as part of occupational safety programs at the commercial swine farm. However, the detection of significant microbiota shifts at T2, despite the variability in how swine workers traditionally adhere to personal protective equipment (PPE) protocols and hygiene [78,79], suggests that farm factors beyond PPE and other biosafety measures contribute to dynamic changes in worker skin microbiota at T2 and T3.

Our findings underscore several critical considerations for future research on workplace and environmental influences on human microbiota. Most importantly, this area of occupational research requires refined methodologies for sampling, microbiome quantification and molecular epidemiologic analysis. These methodologies must be developed for the diverse environmental conditions, varying microbial burdens, and complex interactions at human-animal-environment interfaces. In this study, we employed a multi-site composite sampling strategy to examine the skin microbiome and metagenome across different workday phases. This approach was carefully designed to address three key challenges in occupational skin microbiome research: (a) We

sampled multiple skin sites representing distinct microenvironments (e.g., dry, moist, oily) that support different bacterial taxa; (b) We selected sampling sites based on a range of exposure likelihoods to air, dust, feces and other components of the swine environment, considering sampling areas that were both protected and unprotected by PPE; (c) By pooling samples into a composite, we streamlined the collection process, making it less burdensome and more easily integrated into the daily routines of agricultural workers. Additionally, occupational microbiome research is challenged to establish causal links, i.e., do workplace exposures influence the human microbiome in the long-term, and do these influences lead to different health outcomes? This study is a prime example of this challenge, as we enrolled farm workers who already had months or years of on-farm exposures. Thus, the "baseline" T1 skin microbiome may have already been impacted by previous on-farm exposures, but we have no robust method for detecting these prior impacts. Similarly, it is tempting to compare our data to publicly available human skin microbiome data, but such a comparison would be inextricably biased by confounders such as the well-documented 'healthy worker' effect and other demographic variables known to impact human microbiomes. The need for robust epidemiological study design is even more pronounced in cohort-based microbiome studies because of the lability of the human microbiome. We therefore expressly avoided comparisons across worker and non-worker cohorts and took care not to extrapolate our findings into long-term microbiome or health impacts. Instead, we focused our analysis on a time-series sampling design of the same workers before and after exposure and showering, allowing for a targeted analysis of daily farmwork and biosecurity interventions. This intra-individual focus allowed us to circumvent many of the biases that beset ecological analyses, but at the expense of external validity and ability to draw conclusions about the long-term impact of farm work as compared to non-farm work.

Finally, we used a combination of sequencing and bioinformatic approaches that were tailored to maximize sensitivity for resistome and mobilome analysis while also supporting a broader investigation of the microbiome. Specifically, we used target-enriched shotgun sequencing, an adaptation of traditional shotgun metagenomics, to enhance detection of ARGs and MGEs which are typically undetected by metagenomic workflows [35,80,81]. Such false negative findings are most pronounced in low biomass samples, such as skin, which are replete with non-microbial gDNA [64,82]. Thus, our choice of target-enriched metagenomics was driven by its capacity to reduce false negatives in resistome and mobilome profiling. The enrichment procedure hinges on biotinylated hybridization probes, which tolerate >40% sequence mismatch between the sequence target and the probe (i.e., up to 48 base mismatches across a 120-*mer* oligo probe). This inherent hybridizing flexibility facilitates the capture of both pre-defined targets and other potential closely related variants. Despite this enrichment, resistome and mobilome genes constituted <16% of the host-filtered metagenomic reads in this study, meaning that >84% of sequencing data across samples did not originate from ARGs or MGEs (i.e., was 'off-target'). We used this remaining off-target data to reveal strain-level dynamics from the recovery of specific strain markers, and the analysis of high-quality genomes recovered from human, animal, and environmental metagenomes. However, because the process of target-enrichment purposefully induces a non-uniform impact on metagenomic content, its effects on compositional analyses remain poorly characterized at this time. Given these potential biases, we complemented our analysis with 16S rRNA amplicon sequencing to provide a less biased assessment of microbial community structure across the skin of swine workers, swine, and environmental samples. This dual sequencing strategy ensured both high-resolution resistome-mobilome characterization and robust taxonomic profiling of the skin microbiome.

It is difficult to extrapolate our single-day, single-farm study to longer-term dynamics of the worker skin microbiome, and our results suggest that further work is needed to determine whether daily environmental exposures impact the long-term profile of the worker skin microbiome; and whether such impacts carry over into the general community via human-to-human transfer. We note, however, that this study is the first of its kind to deeply and systematically interrogate the stability of the microbiome, resistome, and mobilome of farm workers as they enter and exit a commercial U.S. swine production system. Owing to difficulties in accessing tightly controlled swine environments, such microbiome exposure assessments on U.S. farms are scarce. These results therefore offer an important foundation from which more robust microbiome investigations can emerge.

MATERIALS AND METHODS

Study design and procedures

Ten workers from a single commercial farrow-to-wean operation in the Midwestern region of the United States were voluntarily enrolled into the study after providing written informed consent. At time of sampling, the farm housed approximately 3,500 sows and >15,000 piglets, and weaning occurred at an average age of 22.5 days of age. This large-scale swine farm had been in operation for >20 years, and was chosen because all facility personnel were day-shift workers and tended to have specialized job tasks requiring a wide array of swine contact, ranging from no direct contact (e.g., facilities maintenance, sanitation, manure management) to intensive direct contact (e.g., assisting with farrowing, piglet processing, or providing veterinary care). The human sample collection events for this study were integrated into a pre-existing, long-standing biosecurity pathogen surveillance program which already involved self-collection of skin swabs. Incorporating microbiome sampling within the existing workplace surveillance program increased study participation and self-sampling consistency, and was determined to be least disruptive to work-related schedules and task performance.

All workers were enrolled and sampled on the same day. Worker enrollment eligibility criteria included a restricted age range (18–60 years); proficiency of spoken and written English; minimum of 6 months continuous employment at the facility; no other contact with swine outside of the workplace; no exposure to antibiotics, immunosuppressants, antifungal, or antiviral drugs in the prior 3 months; no hospitalization or incarceration for > 24 hours in the 3 months prior to enrollment; free of any *known* symptoms, infections, or diseases of the upper-respiratory tract (including ears, nose, throat) and skin at the time of enrollment; no *known* current or past diagnosis of autoimmune disease; no cancer diagnosis or related therapy in the previous 5 years; and an afebrile status at the time of enrollment. Participants were asked to abstain from any additional showering or application of personal care products to their skin other than those required at the swine facility, for a period of 24 hours prior to sampling. Following the provision of study description information and free informed consent forms for review, formal consent was obtained in confidence and without the presence of farm management personnel from all participants. Participants agreed to self-collect repeated skin swabs at three points in their working day, and to complete a short questionnaire regarding personal demographic and biometric information, smoking history, dietary and hygiene habits, and occupational and non-occupational related exposure assessments. Variables from the questionnaire were used in

downstream multivariable statistical analyses. All study participants, their samples, and questionnaire data were kept deidentified to study personnel and investigators using an assigned alphanumeric coding scheme and an electronic tracking system. Monetary remuneration for study participation was provided in the form of $100 debit cards.

Sample collection

Epidermal swabs (hereafter referred to as "skin swabs") were self-collected at three workday collection phases: prior to entry into the animal holding areas of the swine operation and before showering-in (T1: "Workday start"); at the conclusion of the 8-hour period of assigned work, but before showering-out (T2: "Workday end"); and upon exiting the facility following a mandatory showering procedure (T3: "Post-shower"). At each time point, collection kits were provided to workers for self-sampling. The kits contained two sterile BD BBL CultureSwabs EZ (Becton, Dickinson and Co., Franklin Lakes, NJ, USA) pre-moistened with a solution containing 0.9% NaCl and 0.1% Tween-20 biosurfactant (Thermo Fisher Scientific, Waltham, MA, USA). Each swab was used for self-swabbing of all locations on one half of the body (i.e., left or right), to obtain a single composite representing the following locations on each body half: manus and interdigital space, antecubital fossa, popliteal fossa, and axilla[83]. These locations were selected to represent sebaceous, moist, and dry epidermal microenvironments of the human skin[83]. Swab pattern, pressure, duration, and frequency were performed in accordance with the Human Microbiome Project[84] core microbiota sampling protocol A, including the use of a z-like pattern of consistent swabbings over specified surfaces via 50 passes over a 30 second period. For the manus, 'z-like' pattern swabbing was conducted across the palm and surface of the fingers for a 30 second duration as well as an additional 30 seconds of linear passing in the interdigital spaces. For all workday collection phases, the left and right composite swab tips were separated from the swab stems and placed into 1X phosphate-buffered saline (PBS) at pH 7.4, immediately transferred to dry ice for transport, and subsequently placed in -80 ℃ for long-term storage.

Swine skin swab samples were collected as follows: each worker was observed handling or working near specific pens containing sows, piglets, or both in a room during their dayshift. Immediately after the worker completed their contact or their required duties in the specific pen, pen-level composite skin samples were collected by passing a sterile EZ Reach Sponge sampler on the dorsal aspect of the skin of each pig in the pen, from withers to tail-base (World Bioproducts, Woodinville, WA, USA) impregnated with 10 mL of 1X PBS . In cases of farrowing pens, the sow and all piglets in a litter were sampled. In gestational pens, only the pregnant sows contacted by workers assigned to a specific area of the facility were sampled. Swine samples were assigned a worker-matched ID, transferred to sterile Whirl-Pak bags (Nasco, Fork Atkinson, WI, USA) and placed on dry ice for transport.

Two additional sterile EZ Reach Sponges were exposed to ambient air for ~20 seconds, one each in gestational and weaning environments of the swine facility, where workers spent the majority of their dayshift; these swabs were meant to capture the airborne environmental microbes within the primary working areas of the facility. After transport (~2 hours driving distance), all samples were immediately placed in -80 ℃ for long-term storage at the Food Centric Corridor infectious diseases laboratory at the University of Minnesota.

Sample processing and gDNA extraction

All sample processing occurred in a class II biological safety cabinet decontaminated using UV radiation and 70% ethanol in between handling human, animal, and environmental samples. After thawing samples at -20 °C and then at room temperature, human swab tips and excess storage fluid were transferred for DNA extraction. An additional 100 μl of 1X PBS was used to recover possible residual microbial material in each sample tube. For swine and environmental samples, the ~10 mL buffer adsorbed to each polyurethane sponge paddle was expressed and reserved in 50 mL conical centrifuge tubes. An additional 10 mL of fresh 1X PBS was allowed to re-adsorb to each sponge and equilibrate for 10 minutes, and this 'rinse' was expressed into the same conical tube as the original rinsate for subsequent centrifugation (8,000 x g 15 min at 4 °C) and transfer of pellets into ~200 μl PBS for DNA extraction.

Genomic DNA (gDNA) extraction began with mechanical and chemical lysis using the Qiagen DNEasy Powersoil Pro kit (Hilden, Germany, Lot# 163044275) and PowerBead Pro tubes containing zirconium beads and 800 µl lysis buffer. Molecular-grade sterile water was placed into 3 randomly selected tubes to serve as negative controls (extraction blanks). After 6 s of vortexer-mediated homogenization, samples were placed on a 115V BioSpec Products Mini-Beadbeater-96 (Bartlesville, OK, USA) for mechanical lysis. Sample bead beating proceeded at 2400 rpm for 30 s for a total of 3 rounds with a 2-minute pause on ice between each round, to prevent overheating. The remainder of the extraction and purification procedure was performed following the PowerSoil Pro protocol with inhibitor removal steps using the QIAcube Connect automated instrument. The final 50 µl of eluted gDNA was stored at -20 °C.

Additionally, gDNA was extracted from two 200 µl aliquots of ZymoBIOMICS microbial community II standard Catalog# D6310 (Irvine, CA, USA), consisting of 8 prokaryotic and 2 eukaryotic microorganisms in known log-distributed abundance to serve as positive controls. The aliquots were individually centrifuged at 15,000 x g for 5 min, and the pellets were suspended in 400 μl of Qiagen CD1 lysis buffer, transferred to PowerBead Pro tubes, and vortexed for 10 min at maximum speed on a Vortex Genie 2 mixer (Scientific Industries, Bohemia, NY, USA) with a Qiagent adaptor (Catalog# 13000-V1-24). After isolating and reserving the initial supernatant, an additional 400 μl of CD1 lysis buffer was added to the same PowerBead Pro tubes, and these were subjected to bead beating (Mini-Beadbeater-96) at 2400 rpm for four 5-min cycles with 5-min rest intervals on ice in between each cycle. After centrifugation at 15,000 x g for 2 min, the resulting supernatant was recombined with the previously reserved supernatants for each aliquot. The gDNA from the two ~700 μl lysates were isolated and purified on the QIAcube Connect according to the same protocol as for the previously described samples.

16S rRNA amplicon library preparation and sequencing

The 16S rRNA gene copy number in each sample was measured using qPCR prior to library preparation. For 16S sequencing, the target copy number threshold was set at 167,000 molecules / µl. Amplification of libraries was performed using a dual-indexing Illumina primer set (Forward primer: *5'*— TCGTCGGCAGCGTCAGATGTGTATAAGAGACAGCCTACGGGAGGCAGCAG —*3'* and Reverse primer: *5'*— GTCTCGTGGGCTCGGAGATGTGTATAAGAGACAGGGACTACHVGGGTWTCTAAT —*3'*) targeting the V3-V4 region[85]. Amplicons were quantified using a PicoGreen dsDNA assay kit

(Life Technologies, Carlsbad, CA, USA). Sequencing was performed at the University of Minnesota Genomics Center (UMGC) using Illumina's v3 chemistry (2x300 bp paired-end reads) on the Illumina MiSeq platform (San Diego, CA, USA). All libraries were sequenced on the same sequencing run.

Target-enriched metagenomic library preparation and sequencing

All gDNA samples were subjected to a targeted capture and deep sequencing workflow to increase detection sensitivity for the resistome and mobilome features within the metagenomic DNA. This approach was chosen to enhance sequencing and thus detection of ARG and MGE targets, which are rare genomic features within microbiomes[35]. Targeted enrichment was performed using a custom-designed biotinylated cDNA probe panel for selective hybridization and capture. For probe design, a comprehensive list of pre-defined publicly available unique nucleotide sequences was compiled for 7,868 ARGs from MEGARes v2.0[86] (including accessions for: drug resistance, metal resistance, multi-compound resistance, and biocide resistance) and for 738 MGE accessions (including full-length sequences for: (a) integrative conjugative elements (ICE) from ICEBerg v2.0[87] and (b) plasmid replicons of *Enterobacteriaceae* and gram positive bacteria from PlasmidFinder v2.1[88]. These ARG and MGE sequences comprised 8.55 Mb of total sequence. The CATCH pipeline[89] was used to generate the custom probe panel using the following parameters: probe stride: 120; probe length: 120; mismatches: 5; extension coverage: 100. The final probe design contained 71,309 unique 120mer oligos, which provided 100% horizontal coverage of all ARG and MGE targets, with at least 2x depth of probe coverage for every nucleotide. Probes were manufactured by Agilent (Santa Clara, CA, USA) with additional 'bait-boosting' to amplify GC-rich regions (defined as GC >65%) for fast hybridization reactions to produce a final panel of 148,162 probes covering 17.78 Mb. Probes were stored at -80 °C prior to use.

All gDNA samples (n=42) were initially subjected to additional Agencourt AMPure XP (Beckman Coulter, Brea, CA) bead-based purification to retain fragments >100 bp using a 40:50 vol/vol ratio. After size selection, targeted enrichment and library preparation using the Agilent SureSelect XTHS V2 system (Santa Clara, CA, USA) and our custom bait design was performed using a minimum input of 100 ng purified DNA. Initial enzymatic fragmentation employing the Agilent XT Low Input Enzymatic Fragmentation Kit was used to generate short (150-250 bp) fragments which were subsequently end-repaired, dA-tailed, and adapter ligated for Illumina paired-end sequencing with multiplexing, following manufacturer recommendations. After probe-based hybridization following the 90-min PCR protocol, capture using MyOne streptavidin T1 beads (Thermo Fisher Scientific, Waltham, MA, USA) was increased to 2 hours with minimized vortexing (1200 rpm). Additional quality control steps were followed, including use of a Qubit 4.0 fluorometer (Thermo Fisher Scientific, Waltham, MA, USA) and TapeStation 4200 for gDNA analysis (Agilent, Santa Clara, CA, USA), as well as high-sensitivity TapeStation 4200 analysis for pre- and post-capture libraries.

After pooling libraries, a KAPA qPCR Library Quantification kit (Roche, Basel, Switzerland) was used to confirm functionality of the barcoded pool, and a MiSeq Nano run (V2 chemistry, 2x150bp paired-end reads) was used to assess final barcode balance. The final multiplexed library was sequenced by the University of Minnesota Genomics Center on a single lane of a

NovaSeq6000 (Illumina, San Diego, CA, USA) with S4 cell chemistry to obtain 2x150 paired-end reads (675 Gb/lane), with an expected depth of ~54 M paired-end reads per sample.

16S rRNA bioinformatic analysis

Amplicon primers were trimmed from the 5' and 3' ends of forward and reverse reads using cutadapt with default settings[90]. The trimmed sequences were then input to the DADA2 v1.26 pipeline[91] to generate amplicon sequence variants (ASVs). The *filterandtrim* function was used for additional quality trimming, including truncation of forward and reverse reads to 250 bp and 220 bp as well as filtering of phiX reads and reads with a maximum expected error rate >4. Cleaned sequence reads were used as input to the *learnerrors* function and the output error-rate matrix was used in read error correction (i.e., denoising) using the *dada* function. Error corrected reads were aligned and combined into contigs using the *mergepairs* function with the minimum overlap threshold set to 12 bp. An ASV table was generated after removing chimeric contigs using the *removechimera* function. ASVs with a sequence length between 401 and 431 were retained. The *assigntaxonomy* function was used for taxonomic assignment of ASVs using the SILVA v138.2 reference database[92] via the native Bayesian classifier, and species annotation was performed via the *addspecies* function. Potential contaminating ASVs were identified using 16S qPCR copy number results per μl using *isContaminant* function in the Decontam v3.6 R package as implemented in the frequency method[93], and were removed from downstream analysis. The final ASV count matrix with taxonomy file was generated from the DADA2 pipeline and saved in RDS file format for subsequent downstream analysis.

Targeted shotgun metagenomics bioinformatic analysis

An alignment-based approach was used to detect ARG and MGE target sequences in all enriched metagenomic data, as implemented in the AMRPlusPlus v2.0 pipeline[86]. Briefly, read trimming and quality filtering was performed using TRIMMOMATIC v0.33[94]. Host reads were identified in worker swab and swine/environmental samples by aligning sequence reads to the *Homo sapiens* (hg19) and *Sus scrofa* reference genomes, respectively, using BWA v0.7.17[95]. Non-host reads were then extracted using SAMtools v1.9[96] and aligned to the MEGARes v2.0 reference database using BWA-MEM. To mitigate the impact of mobilome misclassification due to sequence homology between MGEs and ARGs[97], we developed a custom script (extract_paired_unmapped.py) to extract paired reads not aligned to any resistome accessions. The ARG-filtered reads were separately aligned to sequences of a concatenated MGE database for ICE, plasmid replicons, plasmid modules, prophage, and virus (bacteriophage) extracted from ICEberg v2.0, PlasmidFinder v2.0.2, and ACLAME v0.4[98]. For PlasmidFinder, only replicon accessions for Gram-positive bacteria and *Enterobacteriaceae* were included. Hits to accessions of plasmid modules, prophage, and virus / bacteriophage within the ACLAME database were further parsed for insertional sequences (IS) and other transposable elements (TEs) utilizing a custom scripting process that automates interfacing with ISfinder[99] and ISbrowser[100] (https://github.com/IS233489/IS_Finder_Annotation). Briefly, this script takes as input reads that are aligned to any aforementioned ACLAME accessions, and uses Entrez queries of the associated NCBI GenBank accessions. Each resulting query outputs metadata details for the accession (e.g., accession_id, start, stop, gene_description, locus name) and the associated full sequences. The Selenium webdriver for Python v4.8.3 is used to automate submission of the GenBank-queried ACLAME sequences to ISbrowser. The native BLASTN algorithm is used to parse hits for plasmid, prophage, and virulence accessions, to detect and differentiate IS families

and related TEs. Query hits for genes were retained if they met the following criteria: minimum identity of 80%, >80% coverage of the query length; and an E-value $\leq 1 \times 10^{-10}$. When multiple hits with similar threshold values were obtained, the assignment with the highest bitscore was chosen. BLASTN results were parsed and each sequence query was called as “likely IS”, “likely TE”, or “unclassified” and flagged for additional manual analysis if an accession was discontinued from further curation in NCBI GenBank. Additionally, all ACLAME accessions not identified as IS or TE were manually checked against current GenBank annotations, and grouped according to putative functional attributes as confirmed with Universal Protein KnowledgeBase (UniProtKB) annotation[101], including: biosynthesis regulation, non-conjugative efflux and transport, relaxase and mobilization machinery, replication initiation and maintenance, plasmid replicon module, DNA secretion and conjugative machinery, stress-SOS-tox/anti-tox-partitioning, transcription and translation regulation, transposition and recombination, and virulence and pathogenicity domains. Accessions with unconfirmed protein function and unassigned annotation in GenBank were considered “unclassified”, and accessions with both an unconfirmed protein function and an unsupported GenBank accession were categorized as “hypothetical”.

To reduce false positive detection of ARGs and MGEs, only ARG and MGE accessions attaining a conservative gene fraction cut-off of 80% within a given sample were considered positively identified, using the default settings in AMRPlusPlus v2.0. Gene fraction was defined as the proportion of nucleotides within a given accession aligned by at least one sequence read. The default deduplication procedures of the AMRPlusPlus v2.0 pipeline were used to account for potential amplification bias introduced during the molecular target enrichment process described above[86]. Identified ARGs and associated alignment counts were aggregated at the ‘group’, ‘mechanism’, and ‘class’ levels using the standard MEGARes ontology. Additionally, 29 ARG groups were flagged as ‘clinically important’ (i.e., priority ARGs) due to their prevalence in clinical disease and frequent co-occurrence with MGEs[36,37,102]: bla_{CTX-M}, bla_{GES}, bla_{IMI}, bla_{KPC},bla_{SHV}, bla_{TEM}, bla_{IMP}, bla_{NDM}, bla_{CMY}, bla_{OXA}, *mecA*, *mcr-1*, *mcr-2*, *vat*, *vga*, *vgb*, bla_{SME}, *cfr*, *aac(6')-I*, bla_{Z}, bla_{VIM}, *ermB*, *qnrA*, *qnrB*, *tetM*, *dfrA*, *vanY-b*, *vanY-d*, *vanY-a*, *sulI*. Prior to analysis, all hits to ARG accessions requiring SNP confirmation (signified by the “*RequiresSNPConfirmation*” in the MEGARes header) were removed, as additional confirmatory assessments would be needed to ensure accurate detection of these genes. Since no mobilome-wide ontology currently exists, counts for MGEs were aggregated to the HGT mechanism type (i.e., ICE, plasmid, prophage, virulence, and IS/TE).

Assessment of sequencing depth, quality, and host genome abundance

Microbiome sequencing variables, including the number of raw reads generated from 16S rRNA sequencing, 16S copy number, and 16S mean quality score were assessed with respect to the following independent variables: collection phase (i.e. Workday start, Workday end, Post-shower, Swine, and Environment), worker exposure type (i.e., direct vs. indirect), and sample type (i.e., human skin swab, swine skin swab, standard mock microbial community, and negative control). Enriched metagenomic sequencing variables, including total generated raw reads (i.e. sequencing depth), total number of host vs. non-host reads, and mean quality score were similarly assessed with respect to collection phase, worker exposure type, and sample type. Associations between these independent variables on 16S and shotgun metagenomic sequencing metrics were independently quantified via linear mixed models using the *lme4* v1.1-29

package[103] with subject ID as random effect to account for repeated measures. Statistical significance of each independent variable was evaluated using ANOVA with a pre-defined alpha of 0.05. All independent variables in the final models were subjected to Tukey-adjusted pairwise comparisons using the Emmeans v2.30-0 package [104], again with a predefined alpha of 0.05. Statistically significant variables were included as covariates in all subsequent models to account for potential confounding related to sequencing effort and/or quality. Assumptions of normality for dependent variables were assessed using visual inspection of residuals, and $\log_{10}$ variable transformations were applied to meet model assumptions.

Quantification of microbiome, resistome, and mobilome diversity and abundance

Richness and Shannon's diversity were estimated for each collection phase and for swine samples using the *estimate_richness* function in the Phyloseq v3.20 package under the Bioconductor release repository. Diversity metrics were calculated at the genus level for 16S data; at the group level for ARGs; and at the mechanism level for MGEs. Differences in diversity were evaluated using linear mixed models via the *lme4* function, specifying 16S copy number (and host-removed sequencing depth for enriched shotgun data) as covariates and worker ID as a random effect. Model building and extraction of model estimates were performed as reported above for the assessment of sequencing depth and quality. Rarefaction analysis and relative abundance analysis of genera, ARG groups, and MGE accessions was performed using the MicrobiotaProcess R package v1.18 [105]. Due to the limited sample size, the 'Environment' samples were excluded from all diversity analyses.

Prior to assessing ***β***-diversity, genera not present in the 16S data or ARGs / MGEs absent from shotgun data across all human and swine samples and were handled using a compositional approach[106] to imputation of zero-inflated count matrices performed using the zCompositions package v1.5.0-4 [107] calling the geometric Bayesian-multiplicative replacement of zero counts function *cmultRepl*, which outputs pseudo-counts. To account for differences in sequencing depth and to attenuate the influence of highly abundant accessions, robust center log ratio (rclr) normalization was applied to all microbiome, resistome, and mobilome counts, which were then transformed to Euclidean distances using the *vegdist* and *decostand* functions in the Vegan package v2.6-8 [108]. The *ordinate* function in Phyloseq was applied to the resulting Aitchison compositions using principal component analysis (PCA). As with the alpha-diversity metrics, beta-diversity was assessed at the genus level for 16S data; at the group level for ARGs; and at the mechanism level for MGEs; with a predefined alpha of 0.05 for all statistical tests. Differences by collection phase were first assessed via the omnibus analysis of similarities test (ANOSIM). Permutational multivariate analysis of variance (PERMANOVA) was performed using the *adonis* function in Vegan based on 10,000 permutations. *Post-hoc* pairwise comparisons were performed using the *pairwise.adonis* function as well as *adonis2* with stratification on worker ID for skin samples when comparing across collection phases T1–T3. As both ANOSIM and PERMANOVA tests are susceptible to dispersion heterogeneity, which may confound between-group with within-group variance, ***β***-dispersion was assessed via the *betadisper* function in Vegan.

Resistome and mobilome feature counts were utilized to analyze the total ARG and MGE sample load. This was done using a modified approach of Li et al [109] where the gene feature counts

are expressed on the basis of the sample qPCR 16S gene copy number as well as the reference feature sequence length. This 'abundance' of the resistome or mobilome was then log_{10}-normalized and summarized by collection phase. Statistical differences in resistome or mobilome abundance were assessed using the linear mixed model approach and pairwise comparisons as described for sequencing and diversity assessments.

Microbiome, resistome, and mobilome differential abundance analysis

Raw count matrices from the microbiome, resistome, and mobilome were utilized as input for differential abundance testing. In addition to filtering for sparse features as described in the beta-diversity analysis, ARGs and MGEs were subjected to additional pruning; specifically, ARGs and MGEs with < 80% prevalence in any of the three collection phases (T1, T2 or T3) or the swine samples were removed from differential abundance analysis. Additionally, for each pairwise set of collection phases being compared during differential abundance analysis, features were censored if they had <10% prevalence and <10 counts. Prior to each differential abundance test, ARGs were agglomerated to both the group and mechanism levels, while MGE accessions were not subjected to agglomeration as no unified MGE taxonomy exists.

All count matrices and associated metadata stored as phyloseq objects were passed to the DESeq2 v1.46.0 R package[110]. The *poscounts* option was used for estimation of size factors which utilizes a modified relative log expression to account for missing alleles across samples. Estimates of dispersions were based on the negative binomial likelihood for each allele as implemented natively in DESeq2. Shrinkage of dispersion estimates and subsequent log_2-fold change (Log_2FC) effect sizes were performed via the empirical Bayes method of adaptive shrinkage implemented in the ashr R package to minimize the false discovery rate (FDR)[111]. Log_2FC estimation was performed using results of a negative binomial generalized linear model specifying collection phase as the main predictor and library cDNA concentration, host-removed sequencing depth, gender, BMI, smoking status, and consumption of pork as model covariates. Hypothesis testing was performed with the default Wald test and FDR adjustment. Differential abundance testing was performed for the following comparisons: workday start (T1) vs. workday end (T2); workday end (T2) vs. post-shower (T3); workday start (T1) vs. post-shower (T3); and the 'interfacing period' represented by swine vs. T2 samples. To guard against spurious findings, pre-defined cut-offs for statistical significance and effect size were used. At the genus level, we used an adjusted alpha of 0.05 and an effect size (log_2FC) of +/-1.5. For ARG group and MGE mechanism levels, we used an adjusted alpha of 0.01 and an effect size (log_2FC) of +/-1.5; for ARGs at the mechanism level, the adjusted alpha was increased to 0.05.

Inferring microbial community connectivity and ecological dominance from sparse datasets

The SParse InversE Covariance Estimation for Ecological Association Inference (SPIEC-EASI)[112] approach was deployed as implemented in R v1.1.1 to infer and analyze ecological networks and keystone members of the microbiome. Separate networks were built for each collection phase and for the swine samples. To build each network, we fit a negative binomial distribution to *clr*-normalized ASV count data. For input, ASV counts found in >1 sample with >100 raw counts were included in all network analyses, based on recommended filtering procedures[112]. Since only two samples were collected for the 'Environment' collection phase,

these samples were not assessed. Model inferences were performed using the Meinshausen and Bühlmann neighborhood selection framework[113], and model sparseness was inferred using the Stability Approach to Regularization Selection (StARS) as implemented in the Pulsar R package v0.3.10 [114] with parameters *lambda.min.ratio*= 0.01 and *nlambda*=20. Stable networks were produced under subsampling with 100 rounds, and subsequently analyzed and visualized using igraph v2.0 R package[115]. Community detection procedures based on analysis of optimized '*spin state*' configurations[116] were applied and modular clustering of subcommunities was evaluated based on Newman and Girvan[117] global modularity (Q) estimation. Network assortativity based on modular identity and node degree was evaluated using base igraph procedures. Eigenvector centrality was regressed according to degree centrality for each ASV (node) to estimate putative keystone membership within each network by identifying taxa in the top 5th percentile.

Identifying strain-level detail in targeted metagenomes

Trimmed and host-removed target-enriched metagenomic reads were mapped to the 1.1M taxonomic markers contained in the MetaPhlAn4 v4.1[118] database using bowtie2 and default parameters[119]. Clade-specific marker coverage and normalization across all detected clades using default parameters produced a relative abundance taxonomic profile for each sample. The sample-specific abundance profiles were merged into a single matrix using the MetaPhlAn4 utility script (merge_metaphlan_tables.py). The matrix was then filtered to species prevalence ≥ 75% of samples. Alignment results on each sample from MetaPhlan3 were used to create files of consensus marker genes for each species. Additional marker sequences were extracted from the MetaPhlAn4 database and in turn were blasted against *Lactobacillus amylovorus* (RefSeq accession: GCA_000194115.1) The sample and reference reconstructed strain marker files were inputted into StrainPhlAn v4.1 (available in MetaPhlAn4), which filters based on the presence of selected clade markers. Multiple sequence alignments for each marker were created by calling PhyloPhlAn (available in MetaPhlAn4) for phylogenetic reconstruction using RAxML and based on default PhyloPhlAn bootstrap values. StrainPhlAn results were used to confirm the presence of specific species of bacteria that had been identified via 16S analysis. StrainPhlAn results were also used to infer sample-level '*sharing*' of strains across collection phases. Briefly, consensus sequences for taxonomic markers were identified by alignment via the MetaPhlan4 pipeline across all metagenomic samples. Major species-level genome bins (SGBs) for all Staphylococci, Streptococci, and *Escherichia coli* identified in initial StrainPhlan results were used to extract SGB marker genes. After extracting SGBs (*Staphylococcus epidermidis*: SGB7865; *Staphylococcus haemolyticus*: SGB7860, SGB7861; *Staphylococcus hominis*: SGB7858; *Escherichia coli*: SGB10068; *Streptococcus suis*: SGB8209, SGB29820; *Streptococcus alactolyticus*: SGB8017), strain sharing was assessed by profiling the maximum similarity across as many samples as possibles using the required inputs and the following parameters: *strainphlan --mutation_rates --marker_in_n_samples 1 --sample_with_n_markers 10 --phylophlan_mode accurate*. Pairwise alignment between targeted SGB markers from each sample pair were aligned and an RAxML phylogenetic distance was inferred for any putative transmission events.

De novo assembly and binning of genomes from targeted metagenomes

Adapter-trimmed and host-removed target-enriched metagenomic reads were used as input for metagenomic assembly with MEGAHIT v1.2.9 [120]. Single-sample assembly for each of the 42 metagenomes was carried out using default parameters. In addition, the 42 metagenomes were co-assembled using MEGAHIT on forward and reverse reads with options: *--continue --kmin-1pass --min-contig-len 1000*. Contigs >2000bp were mapped to single and co-assemblies via BWA-MEM[95], and SAMtools was used to sort and convert SAM files to BAM files[96]. The MetaBAT2 v2.15 [121] pipeline was used to assess coverage of assembled contigs and for both single- and co-assembly binning. Co-assemblies and single-sample assemblies were constructed separately to produce 638 and 712 bins, respectively. All bins were aggregated and dRep v3.4.0 [122] was used for *post hoc* dereplication with the following flags: *dereplicate -comp 80 -con 10 -sa 0.95* and CheckM v1.2.2 [123] lineage workflow was used to retain only those MAGs with contamination <10 % and completeness >80% using *--checkM_method lineage_wf*. After retaining all primary bins and only the highest-scoring secondary cluster bins, we produced 145 metagenome-assembled genomes (i.e., MAGs) from co-assemblies and 73 MAGs from single-sample assemblies. MAGs were assigned a taxonomy using GTDB-tk v2.1.1 [124], and of the 218 MAGs, 5 were assigned to archaea and not considered in further downstream analysis. Of the remaining 213 draft genomes, >57% (n= 123) were categorized as '*high-quality*', >34% (n= 74) as '*medium- quality*' with minimal contamination, and <8% (n=16) as '*medium-quality*' draft genomes (**Figure 7a**). We detected no significant differences (Type III ANOVA p>0.1) in genomic parameters for recovered MAGs across T1–T3, swine, and environmental samples (**Supplementary datafile 12**), including GC ratio (median range: 35.5–37.4), ANI distribution (median range: 98.2–98.8%), and N50 contig length distribution (median range: 11,949–15,798 bp). MAGs were assessed for their taxonomic novelty by following the procedures of Glendinning et al. [125] Briefly, MAGs with ANI <95% were considered as putatively novel species, and ANI <99% as putatively novel strains. Additionally, MAGs not assigned a provisional genus name were assessed for genus-level novelty via CompareM v0.1.2 at <60% AAI. Multiple sequence alignment files generated from the GTDB-tk workflow were concatenated across all collection phases and used as input for phylogenomic clustering as implemented in IQTREE v2.2.0 [126]. A best-fit substitution model was chosen using the native *ModelFinder Plus* via the Bayesian Information Criterion (BIC). The resulting model (LG+R8) was used to construct the taxonomic tree of MAGs, which was visualized across all study samples using iTOL [127].

ETHICAL APPROVAL AND CONSENT TO PARTICIPATE

The University of Minnesota's Institutional Review Board approved the study (protocol: *STUDY 00007351*) as no greater than minimal risk to study participants. All procedures, including obtaining informed consent, were followed in accordance with the ethical stands of the Office for Human Research Protections (U.S. Department of Health and Human Services) and with the Helsinki Declaration (2013). All animals were sampled under authorization from the Institutional Animal Care and Use Committees of the University of Minnesota and participating farms under a collaborative agreement (protocol #5-19).

DATA AVAILABILITY

Upon publication, raw sequence data and sample metadata can be accessed via the Sequence Read Archive (SRA) hosted by the National Center for Biotechnology Information (NCBI) under BioProject PRJNA987158. Sample metadata was recorded using the MIMARKS host-associated metagenomic sample guidelines (Yilmaz et al., 2011) All statistical analysis scripts were executed on R V4.2.0 and are publicly available at https://github.com/IS233489/LaborOME-project.

AUTHOR CONTRIBUTIONS

IBS, NRN, SAD, JN, and CO conceptualized the study design. IBS and CO executed the experimental design and IBS conducted the laboratory work. IBS and PMF performed all bioinformatic analysis with guidance from CB and NRN. JEB was instrumental

in generating parsing scripts to facilitate mobilome annotation. IBS and TNG performed quantitative analysis of resulting datasets. IBS conceptualized and edited graphical data in the manuscript with input from NRN. IBS and NRN wrote the manuscript with critical input from SAD, JN, CO, JEB, and CB. All authors read and approved the final version of the manuscript.

FUNDING

Financial support for this work was provided by the National Institute of Health (NIH) National Institute of Allergy and Infectious Disease (NIAID), *Project No. 1R01AI141810-01*; the Midwest Center for Occupational Safety and Health (MCOHS) Pilot Projects Research Training Program (PPRTP) funded through the National Institute of Occupational Safety and Health (NIOSH), *Project No.* T42 OH008434; and the University of Minnesota Doctoral Dissertation Fellowship.

FIGURE CAPTIONS

Figure 1. Study overview. Farm workers from a commercial farrow-to-wean operation in the Midwestern United States were voluntarily enrolled into a single longitudinal microbiome sampling campaign during a typical 8-hour workday shift. For each worker, swab kits were used to self-collect samples from the epidermis in a standardized fashion by passing each swab across four body sites, achieving a single composite skin sample for the left and right body representing microbiomes from the manus, interdigital space, antecubital fossa, popliteal fossa, and axilla. Workers were asked to perform the first self-collection ('Sample **T1**') prior to entry into the swine facility (**1**). Workers underwent mandatory showering prior to entry into the animal holding areas (**2**). During the day shift, workers were observed handling animals or working in specific animal pens, and dorsal skin swabs (from withers to tail-base) were taken from contact-matched animals on a pooled multi-pen level. Additionally, a 15-minute questionnaire was administered to collect biometric, health, lifestyle, and occupational task performance information from each worker (**3**). In a similar fashion, self-collected skin samples were taken immediately upon conclusion of the workday ('Sample **T2**') (**4**). Workers underwent mandatory showering procedures immediately after exiting the animal holding areas (**5**) and a third self-collection of samples was performed ('Sample **T3**') after showering and immediately prior to exiting the farm facility (**6**).

Figure 2. Changes in skin microbial load, microbiome composition, and community structure. a. Genus-level worker skin microbiome $\boldsymbol{\beta}$-diversity across collection phases (T1–T3) and contact-matched swine skin samples using principal component ordination of robust Aitchison compositions. Within-group centroids and 95% confidence intervals are depicted with a large circle and shaded ellipsoids, respectively. **b.** Log_{10}-normalized 16S rRNA qPCR copy number / µl (y-axis), stratified by collection phase (x-axis). *** indicates statistical significance (p<0.001) of pairwise comparisons based on a linear regression model with Tukey's adjustment for multiple comparisons. **c.** General linear analysis of the log_{10}-normalized 16S rRNA qPCR copy number / µl (y-axis) and log_{10}-normalized hourly exposure to swine (x-axis) based on workers' estimates from daily task assignments indicates a negative correlation (p= 0.01) across all collection phases. Shaded areas represent the 95% confidence interval around the linear trendline. **d.** Worker skin microbiome networks across workday collection phases and for contact-matched swine were inferred from inverse covariance estimation for compositions based on centered-log ratios of subsetted ASV counts containing >100 counts and >10% prevalence per ASV per sample. Inferred networks consist of nodes representing ASVs colored by shared subcommunity membership. Edges between nodes represent a significant predicted positive (blue) or negative (gray) interaction. Reported topology characteristics include network connectivity based on the edge to node ratio ($E:N$), modularity (Q), subcommunity assortativity (r_n), and degree assortativity (r_d). **e**. Scatter plots of microbial constituents from the corresponding networks are displayed based on the log_{10}-normalized node eigenvector centrality (y-axis) and node degree centrality (x-axis). Taxa with the highest centrality measures (top right of the distribution) are considered to be critical connectors and major hubs in community networks, and thus putative keystone taxa. ASV-level nodes are colored based on their taxonomic classification at the Class level. Genus-level labels are displayed only for genera most likely to be keystone, i.e., >95th percentile of the plot distribution (top right) and least likely to be keystone, i.e.<5th percentile of the plot distribution (bottom left).

Figure 3. Workday fluctuations in worker skin resistome and mobilome composition, compared to contact-matched swine. Principal component ordination of robust Aitchison compositions for the **a** total resistome and **b** major components of the total mobilome, including plasmids, integrative conjugative elements, virus, prophage, insertional sequences, and transposable elements. Clustering significance was explored with the omnibus analysis of similarity (ANOSIM) test (p<0.05). Ordinations are summarized by depicting within-group centroids and shaded ellipsoids representing the 90% confidence interval. The distribution of major mobilome accessions are displayed as relative abundance stacked bar graphs across collection phases and individual samples for **c** major MGE mechanisms; **d** plasmidic sequences; and. **e** insertional sequence family identity. Legend abbreviations include ICE: integrative conjugative element, IS: insertional

sequences, Plasmid ARG: Plasmid-borne antimicrobial resistance genes, TE: Transposable elements; PREPIM: Plasmid replication initiation and maintenance; PTTREG: Plasmid transcription, translation, and regulation.

Figure 4. Occurrence of medically important ARGs on the skin of workers and swine. Unique medically important (i.e., priority) ARG alleles were identified at >99.9% alignment gene coverage across all collection phases (top ribbon annotation) and are displayed using a heatmap summarizing their sample-level relative abundance across each of the respective 19 ARG gene groups. The cladograms along the x-axis demonstrate the hierarchical clustering of samples according to their medically important resistome composition using optimal leaf sorting and euclidean distances. Four major subclades are colored and numbered. Major ARG group prevalence and median abundance across study samples are summarized via the associated barplots and boxplots along the y-axis.

Figure 5. Differential abundance analysis across collection phases and at the interface period between workers and pigs. Volcano plots are used to visualize differential abundance of unique **a** ARG groups and **b** MGE accessions in log_2-fold change (x-axis) and $-log_{10}P$ value (y-axis) of the global worker skin resistome or mobilome between key workshift collection phases: Workday start (**T1**) vs. workday end (**T2**); Workday end (**T2**) vs. post-shower (**T3**); and Workday start (**T1**) vs. post-shower (**T3**). An additional comparison is made between workday end and swine skin samples representing the worker's contact phase with animals (gray). Features with significant shift in abundance (Wald's $p<0.01$ with FDR adjustment for ARG group and MGE accessions) are displayed above the horizontal line, while biologically significant fold-change is demarcated by vertical dashed lines at 1.5 log_2-fold change. Labels are displayed for only the 5 most abundant ARG groups and 10 most abundant MGEs significantly amplified (log_2-fold change >1.5 or < -1.5) at each phase comparison. For each volcano plot, an associated pie chart displays the number of unique ARG groups and MGE accessions common to each of the workshift collection phases compared, as well as the proportion of the total differentially abundant MGEs associated with each phase.

Figure 6. Taxonomic diversity and novelty of resolved metagenomic assembled genomes (MAGs) recovered from the human-swine interface and ambient environment. a Scatter plot of points representing individual GTDB taxonomically binned MAGs according to their estimated percent completeness (x-axis) relative to their estimated percent contamination (y-axis). Point size is proportional to the quantified MAG GC-content. Pink, purple, and gray regions of the plot demarcate the density of 'high quality', 'medium quality-low contamination', and 'medium quality' MAGs retrieved across all collection phases. **b** Scatter plot of estimated MAG size is displayed based on the log_{10}-normalized nucleotide count (x-axis) and the nearest assigned NCBI reference genome size expressed in log_{10}-normalized nucleotide count (y-axis). A global regression line and 95% confidence interval (shaded region) is displayed and results of significance testing (R^2; $p<0.05$) using a generalized linear model are colored for MAGs recovered for each collection phase. **c** Phylogenomic tree of de-replicated and high-confidence MAGs recovered from target-enriched metagenomes across each collection phase (outer ring). The area below each leaf is colored according to the taxonomically assigned Phylum. Branches for proposed novel species (ANI <95%) are displayed in teal, and for proposed novel strains (ANI <99%) are displayed in purple.

REFERENCES

1. Wu BG, Kapoor B, Cummings KJ, Stanton ML, Nett RJ, Kreiss K, et al.. Evidence for Environmental–Human Microbiota Transfer at a Manufacturing Facility with Novel Work-related Respiratory Disease. *Am J Respir Crit Care Med*. American Thoracic Society - AJRCCM; 2020; doi: 10.1164/rccm.202001-0197OC.
2. Lai PS, Christiani DC. Impact of occupational exposure on human microbiota. *Curr Opin Allergy Clin Immunol*. 2019; doi: 10.1097/ACI.0000000000000502.
3. Lai PS, Allen JG, Hutchinson DS, Ajami NJ, Petrosino JF, Winters T, et al.. Impact of environmental microbiota on human microbiota of workers in academic mouse research facilities: An observational study. *PLOS ONE*. Public Library of Science; 2017; doi: 10.1371/journal.pone.0180969.
4. M. Marcelloni A, Chiominto A, Di Renzi S, Melis P, Wirz A, C. Riviello M, et al.. How Working Tasks Influence Biocontamination in an Animal Facility. *Applied Sciences*. Multidisciplinary Digital Publishing Institute; 2019; doi: 10.3390/app9112216.
5. Sun J, Liao X-P, D'Souza AW, Boolchandani M, Li S-H, Cheng K, et al.. Environmental remodeling of human gut microbiota and antibiotic resistome in livestock farms. *Nature Communications*. Nature Publishing Group; 2020; doi: 10.1038/s41467-020-15222-y.
6. Song SJ, Lauber C, Costello EK, Lozupone CA, Humphrey G, Berg-Lyons D, et al.. Cohabiting family

members share microbiota with one another and with their dogs. *Elife*. 2013; doi: 10.7554/eLife.00458.

7. Mucci N, Tommasi E, Chiarelli A, Lulli L, Traversini V, Galea R, et al.. WORKbiota: A Systematic Review about the Effects of Occupational Exposure on Microbiota and Workers' Health. *IJERPH*. 2022; doi: 10.3390/ijerph19031043.

8. Peng M, Biswas D. Environmental Influences of High-Density Agricultural Animal Operation on Human Forearm Skin Microflora. *Microorganisms*. 2020; doi: 10.3390/microorganisms8101481.

9. Yang D, Heederik DJJ, Scherpenisse P, Van Gompel L, Luiken REC, Wadepohl K, et al.. Antimicrobial resistance genes aph(3')-III, erm(B), sul2 and tet(W) abundance in animal faeces, meat, production environments and human faeces in Europe. *J Antimicrob Chemother*. 2022; doi: 10.1093/jac/dkac133.

10. Van Gompel L, Luiken REC, Hansen RB, Munk P, Bouwknegt M, Heres L, et al.. Description and determinants of the faecal resistome and microbiome of farmers and slaughterhouse workers: A metagenome-wide cross-sectional study. *Environ Int*. 2020; doi: 10.1016/j.envint.2020.105939.

11. Hammerum AM, Larsen J, Andersen VD, Lester CH, Skovgaard Skytte TS, Hansen F, et al.. Characterization of extended-spectrum β-lactamase (ESBL)-producing Escherichia coli obtained from Danish pigs, pig farmers and their families from farms with high or no consumption of third- or fourth-generation cephalosporins. *Journal of Antimicrobial Chemotherapy*. 2014; doi: 10.1093/jac/dku180.

12. Oppliger A, Moreillon P, Charrière N, Giddey M, Morisset D, Sakwinska O. Antimicrobial Resistance of Staphylococcus aureus Strains Acquired by Pig Farmers from Pigs. *Appl Environ Microbiol*. 2012; doi: 10.1128/AEM.01902-12.

13. Sun J, Yang M, Sreevatsan S, Bender JB, Singer RS, Knutson TP, et al.. Longitudinal study of Staphylococcus aureus colonization and infection in a cohort of swine veterinarians in the United States. *BMC Infect Dis*. 2017; doi: 10.1186/s12879-017-2802-1.

14. Hatcher SM, Rhodes SM, Stewart JR, Silbergeld E, Pisanic N, Larsen J, et al.. The Prevalence of Antibiotic-Resistant Staphylococcus aureus Nasal Carriage among Industrial Hog Operation Workers, Community Residents, and Children Living in Their Households: North Carolina, USA. *Environ Health Perspect*. 2017; doi: 10.1289/EHP35.

15. Chen D, Cheng K, Wan L, Cui C, Li G, Zhao D, et al.. Daily occupational exposure in swine farm alters human skin microbiota and antibiotic resistome. *Imeta*. 2024; doi: 10.1002/imt2.158.

16. Ding D, Zhu J, Gao Y, Yang F, Ma Y, Cheng X, et al.. Effect of cattle farm exposure on oropharyngeal and gut microbial communities and antibiotic resistance genes in workers. *Science of The Total Environment*. 2022; doi: 10.1016/j.scitotenv.2021.150685.

17. : Wages and Benefits for Farm Employees | Ag Decision Maker. https://www.extension.iastate.edu/agdm/wholefarm/html/c1-60.html Accessed 2023 Mar 27.

18. : Swine Human Resources: Managing Employees – Hogs, Pigs, and Pork. https://swine.extension.org/swine-human-resources-managing-employees/ Accessed 2023 Mar 27.

19. : CDC Interim Guidance for Workers who are Employed at Commercial Swine Farms: Preventing the Spread of Influenza A Viruses | CDC. https://www.cdc.gov/flu/swineflu/guidance-commercial-pigs.htm (2020). Accessed 2023 Apr 1.

20. : USDA APHIS | Pork Producers. https://www.aphis.usda.gov/aphis/resources/pests-diseases/asf/asf-producers Accessed 2023 Apr 1.

21. Grice EA, Kong HH, Conlan S, Deming CB, Davis J, Young AC, et al.. Topographical and Temporal Diversity of the Human Skin Microbiome. *Science*. American Association for the Advancement of Science; 2009; doi: 10.1126/science.1171700.

22. Bouslimani A, Porto C, Rath CM, Wang M, Guo Y, Gonzalez A, et al.. Molecular cartography of the human skin surface in 3D. *Proceedings of the National Academy of Sciences*. Proceedings of the National Academy of Sciences; 2015; doi: 10.1073/pnas.1424409112.

23. Agostinetto G, Bozzi D, Porro D, Casiraghi M, Labra M, Bruno A. SKIOME Project: a curated collection of skin microbiome datasets enriched with study-related metadata. *Database*. 2022; doi: 10.1093/database/baac033.

24. Song Y, Chen K, Lv L, Xiang Y, Du X, Zhang X, et al.. Uncovering the biogeography of the

microbial commmunity and its association with nutrient metabolism in the intestinal tract using a pig model. *Front Nutr*. Frontiers; 2022; doi: 10.3389/fnut.2022.1003763.
25. Heras-Molina A, Estellé J, Vázquez-Gómez M, López-García A, Pesantez-Pacheco J-L, Astiz S, et al.. The impact of host genetics on porcine gut microbiota composition excluding maternal and postnatal environmental influences. *PLoS One*. 2024; doi: 10.1371/journal.pone.0315199.
26. Emami NK, Schreier LL, Greene E, Tabler T, Orlowski SK, Anthony NB, et al.. Ileal microbial composition in genetically distinct chicken lines reared under normal or high ambient temperatures. *Animal Microbiome*. 2022; doi: 10.1186/s42523-022-00183-y.
27. Duggett NA, Kay GL, Sergeant MJ, Bedford M, Constantinidou CI, Penn CW, et al.. Draft Genome Sequences of Six Novel Bacterial Isolates from Chicken Ceca. *Genome Announcements*. American Society for Microbiology; 2016; doi: 10.1128/genomea.00448-16.
28. Buiatte V, Fonseca A, Alonso Madureira P, Nakashima Vaz AC, Tizioto PC, Centola Vidal AM, et al.. A comparative study of the bacterial diversity and composition of nursery piglets' oral fluid, feces, and housing environment. *Sci Rep*. Nature Publishing Group; 2024; doi: 10.1038/s41598-024-54269-5.
29. Vlasblom AA, Duim B, Patel S, Luiken REC, Crespo-Piazuelo D, Eckenberger J, et al.. The developing pig respiratory microbiome harbors strains antagonistic to common respiratory pathogens. *mSystems*. American Society for Microbiology; 2024; doi: 10.1128/msystems.00626-24.
30. Li A, Chu Y, Wang X, Ren L, Yu J, Liu X, et al.. A pyrosequencing-based metagenomic study of methane-producing microbial community in solid-state biogas reactor. *Biotechnology for Biofuels*. 2013; doi: 10.1186/1754-6834-6-3.
31. van Rensburg JJ, Lin H, Gao X, Toh E, Fortney KR, Ellinger S, et al.. The Human Skin Microbiome Associates with the Outcome of and Is Influenced by Bacterial Infection. *mBio*. American Society for Microbiology; 2015; doi: 10.1128/mbio.01315-15.
32. Flowers L, Grice EA. The Skin Microbiota: Balancing Risk and Reward. *Cell Host & Microbe*. Elsevier; 2020; doi: 10.1016/j.chom.2020.06.017.
33. Strube ML, Hansen JE, Rasmussen S, Pedersen K. A detailed investigation of the porcine skin and nose microbiome using universal and Staphylococcus specific primers. *Sci Rep*. Nature Publishing Group; 2018; doi: 10.1038/s41598-018-30689-y.
34. Arikan M, Yildiz Z, Kahraman Demir T, Yilmaz NH, Sen A, Hanoglu L, et al.. Axillary Microbiota Is Associated with Cognitive Impairment in Parkinson's Disease Patients. *Microbiology Spectrum*. American Society for Microbiology; 2022; doi: 10.1128/spectrum.02358-21.
35. Noyes NR, Weinroth ME, Parker JK, Dean CJ, Lakin SM, Raymond RA, et al.. Enrichment allows identification of diverse, rare elements in metagenomic resistome-virulome sequencing. *Microbiome*. 2017; doi: 10.1186/s40168-017-0361-8.
36. Zhang A-N, Gaston JM, Dai CL, Zhao S, Poyet M, Groussin M, et al.. An omics-based framework for assessing the health risk of antimicrobial resistance genes. *Nat Commun*. 2021; doi: 10.1038/s41467-021-25096-3.
37. Nielsen TK, Browne PD, Hansen LH. Antibiotic resistance genes are differentially mobilized according to resistance mechanism. *GigaScience*. 2022; doi: 10.1093/gigascience/giac072.
38. Davis MF, Pisanic N, Rhodes SM, Brown A, Keller H, Nadimpalli M, et al.. Occurrence of Staphylococcus aureus in swine and swine workplace environments on industrial and antibiotic-free hog operations in North Carolina, USA: a One Health pilot study. *Environ Res*. 2018; doi: 10.1016/j.envres.2017.12.010.
39. May L, Klein EY, Rothman RE, Laxminarayan R. Trends in Antibiotic Resistance in Coagulase-Negative Staphylococci in the United States, 1999 to 2012. *Antimicrobial Agents and Chemotherapy*. American Society for Microbiology; 2014; doi: 10.1128/aac.01908-13.
40. Becker K, Heilmann C, Peters G. Coagulase-Negative Staphylococci. *Clin Microbiol Rev*. 2014; doi: 10.1128/CMR.00109-13.
41. Barros EM, Ceotto H, Bastos MCF, dos Santos KRN, Giambiagi-deMarval M. Staphylococcus haemolyticus as an Important Hospital Pathogen and Carrier of Methicillin Resistance Genes. *J Clin Microbiol*. 2012; doi: 10.1128/JCM.05563-11.

42. Costa SS, Sobkowiak B, Parreira R, Edgeworth JD, Viveiros M, Clark TG, et al.. Genetic Diversity of norA, Coding for a Main Efflux Pump of Staphylococcus aureus. *Frontiers in Genetics*. 92019;
43. Houdt RV, Toussaint A, Ryan MP, Pembroke JT, Mergeay M, Adley CC. The Tn4371 ICE Family of Bacterial Mobile Genetic Elements. Madame Curie Bioscience Database [Internet]. Landes Bioscience;
44. Razavi M, Kristiansson E, Flach C-F, Larsson DGJ. The Association between Insertion Sequences and Antibiotic Resistance Genes. *mSphere*. American Society for Microbiology; 2020; doi: 10.1128/mSphere.00418-20.
45. Roberts AP, Mullany P. Tn916-like genetic elements: a diverse group of modular mobile elements conferring antibiotic resistance. *FEMS Microbiology Reviews*. 2011; doi: 10.1111/j.1574-6976.2011.00283.x.
46. Howden BP, Seemann T, Harrison PF, McEvoy CR, Stanton J-AL, Rand CJ, et al.. Complete Genome Sequence of Staphylococcus aureus Strain JKD6008, an ST239 Clone of Methicillin-Resistant Staphylococcus aureus with Intermediate-Level Vancomycin Resistance. *Journal of Bacteriology*. American Society for Microbiology; 2010; doi: 10.1128/JB.00951-10.
47. Sanderson H, Ortega-Polo R, Zaheer R, Goji N, Amoako KK, Brown RS, et al.. Comparative genomics of multidrug-resistant Enterococcus spp. isolated from wastewater treatment plants. *BMC Microbiol*. 2020; doi: 10.1186/s12866-019-1683-4.
48. Bellanger X, Payot S, Leblond-Bourget N, Guédon G. Conjugative and mobilizable genomic islands in bacteria: evolution and diversity. *FEMS Microbiology Reviews*. 2014; doi: 10.1111/1574-6976.12058.
49. Sebaihia M, Wren BW, Mullany P, Fairweather NF, Minton N, Stabler R, et al.. The multidrug-resistant human pathogen Clostridium difficile has a highly mobile, mosaic genome. *Nat Genet*. 2006; doi: 10.1038/ng1830.
50. Welter DK, Ruaud A, Henseler ZM, De Jong HN, van Coeverden de Groot P, Michaux J, et al.. Free-Living, Psychrotrophic Bacteria of the Genus Psychrobacter Are Descendants of Pathobionts. *mSystems*. American Society for Microbiology; 2021; doi: 10.1128/mSystems.00258-21.
51. Byrne-Bailey KG, Gaze WH, Kay P, Boxall ABA, Hawkey PM, Wellington EMH. Prevalence of sulfonamide resistance genes in bacterial isolates from manured agricultural soils and pig slurry in the United Kingdom. *Antimicrob Agents Chemother*. 2009; doi: 10.1128/AAC.00652-07.
52. Cobo-Díaz JF, Alvarez-Molina A, Alexa EA, Walsh CJ, Mencía-Ares O, Puente-Gómez P, et al.. Microbial colonization and resistome dynamics in food processing environments of a newly opened pork cutting industry during 1.5 years of activity. *Microbiome*. 2021; doi: 10.1186/s40168-021-01131-9.
53. Ingham AC, Urth TR, Sieber RN, Stegger M, Edslev SM, Angen Ø, et al.. Dynamics of the Human Nasal Microbiota and Staphylococcus aureus CC398 Carriage in Pig Truck Drivers across One Workweek. *Applied and Environmental Microbiology*. American Society for Microbiology; 2021; doi: 10.1128/AEM.01225-21.
54. Chen C, Zhou Y, Fu H, Xiong X, Fang S, Jiang H, et al.. Expanded catalog of microbial genes and metagenome-assembled genomes from the pig gut microbiome. *Nat Commun*. Nature Publishing Group; 2021; doi: 10.1038/s41467-021-21295-0.
55. Saheb Kashaf S, Proctor DM, Deming C, Saary P, Hölzer M, NISC Comparative Sequencing Program, et al.. Integrating cultivation and metagenomics for a multi-kingdom view of skin microbiome diversity and functions. *Nat Microbiol*. 2022; doi: 10.1038/s41564-021-01011-w.
56. Arikawa K, Ide K, Kogawa M, Saeki T, Yoda T, Endoh T, et al.. Recovery of strain-resolved genomes from human microbiome through an integration framework of single-cell genomics and metagenomics. *Microbiome*. 2021; doi: 10.1186/s40168-021-01152-4.
57. Liu G, Li P, Hou L, Niu Q, Pu G, Wang B, et al.. Metagenomic Analysis Reveals New Microbiota Related to Fiber Digestion in Pigs. *Frontiers in Microbiology*. 122021;
58. Crossfield M, Gilroy R, Ravi A, Baker D, La Ragione RM, Pallen MJ. Archaeal and Bacterial Metagenome-Assembled Genome Sequences Derived from Pig Feces. *Microbiol Resour Announc*. 2022; doi: 10.1128/mra.01142-21.
59. Ahn J, Hayes RB. Environmental Influences on the Human Microbiome and Implications for Noncommunicable Disease. *Annual Review of Public Health*. 2021; doi: 10.1146/annurev-publhealth-

012420-105020.
60. Rothschild D, Weissbrod O, Barkan E, Kurilshikov A, Korem T, Zeevi D, et al.. Environment dominates over host genetics in shaping human gut microbiota. *Nature*. 2018; doi: 10.1038/nature25973.
61. Gacesa R, Kurilshikov A, Vich Vila A, Sinha T, Klaassen M a. Y, Bolte LA, et al.. Environmental factors shaping the gut microbiome in a Dutch population. *Nature*. Nature Publishing Group; 2022; doi: 10.1038/s41586-022-04567-7.
62. Oh J, Byrd AL, Park M, Kong HH, Segre JA. Temporal Stability of the Human Skin Microbiome. *Cell*. 2016; doi: 10.1016/j.cell.2016.04.008.
63. Costello EK, Lauber CL, Hamady M, Fierer N, Gordon JI, Knight R. Bacterial Community Variation in Human Body Habitats Across Space and Time. *Science*. American Association for the Advancement of Science; 2009; doi: 10.1126/science.1177486.
64. Boxberger M, Cenizo V, Cassir N, La Scola B. Challenges in exploring and manipulating the human skin microbiome. *Microbiome*. 2021; doi: 10.1186/s40168-021-01062-5.
65. Larson KRL, Smith TC, Donham KJ. Self-reported Methicillin-resistant Staphylococcus aureus infection in USA pork producers.
66. Beaudoin A, Johnson S, Davies P, Bender J, Gramer M. Characterization of Influenza A Outbreaks in Minnesota Swine Herds and Measures Taken to Reduce the Risk of Zoonotic Transmission. *Zoonoses and Public Health*. 2012; doi: 10.1111/j.1863-2378.2011.01423.x.
67. Myers KP, Olsen CW, Setterquist SF, Capuano AW, Donham KJ, Thacker EL, et al.. Are swine workers in the United States at increased risk of infection with zoonotic influenza virus? *Clin Infect Dis*. 2006; doi: 10.1086/498977.
68. Chen C, Wu F. Livestock-associated methicillin-resistant Staphylococcus aureus (LA-MRSA) colonisation and infection among livestock workers and veterinarians: a systematic review and meta-analysis. *Occup Environ Med*. BMJ Publishing Group Ltd; 2021; doi: 10.1136/oemed-2020-106418.
69. Sieber RN, Skov RL, Nielsen J, Schulz J, Price LB, Aarestrup FM, et al.. Drivers and Dynamics of Methicillin-Resistant Livestock-Associated Staphylococcus aureus CC398 in Pigs and Humans in Denmark. *mBio*. American Society for Microbiology; 2018; doi: 10.1128/mBio.02142-18.
70. Sudatip D, Mostacci N, Thamlikitkul V, Oppliger A, Hilty M. Influence of occupational exposure to pigs or chickens on human gut microbiota composition in Thailand. *One health*. Elsevier; 15:1004632022;
71. Sudatip D, Mostacci N, Tiengrim S, Thamlikitkul V, Chasiri K, Kritiyakan A, et al.. The risk of pig and chicken farming for carriage and transmission of Escherichia coli containing extended-spectrum beta-lactamase (ESBL) and mobile colistin resistance (mcr) genes in Thailand. *Microbial Genomics*. Microbiology Society,; 2023; doi: 10.1099/mgen.0.000951.
72. Maciel-Guerra A, Baker M, Hu Y, Wang W, Zhang X, Rong J, et al.. Dissecting microbial communities and resistomes for interconnected humans, soil, and livestock. *ISME J*. Nature Publishing Group; 2023; doi: 10.1038/s41396-022-01315-7.
73. Mencía-Ares O, Borowiak M, Argüello H, Cobo-Díaz JF, Malorny B, Álvarez-Ordóñez A, et al.. Genomic Insights into the Mobilome and Resistome of Sentinel Microorganisms Originating from Farms of Two Different Swine Production Systems. *Microbiology Spectrum*. American Society for Microbiology; 2022; doi: 10.1128/spectrum.02896-22.
74. Gao F-Z, He L-Y, He L-X, Bai H, Zhang M, Chen Z-Y, et al.. Swine farming shifted the gut antibiotic resistome of local people. *Journal of Hazardous Materials*. 2024; doi: 10.1016/j.jhazmat.2023.133082.
75. Kraemer JG, Aebi S, Oppliger A, Hilty M. The Indoor-Air Microbiota of Pig Farms Drives the Composition of the Pig Farmers' Nasal Microbiota in a Season-Dependent and Farm-Specific Manner. *Applied and Environmental Microbiology*. American Society for Microbiology; doi: 10.1128/AEM.03038-18.
76. Luiken REC, Van Gompel L, Bossers A, Munk P, Joosten P, Hansen RB, et al.. Farm dust resistomes and bacterial microbiomes in European poultry and pig farms. *Environment International*. 2020; doi: 10.1016/j.envint.2020.105971.
77. Kirjavainen PV, Karvonen AM, Adams RI, Täubel M, Roponen M, Tuoresmäki P, et al.. Farm-like

indoor microbiota in non-farm homes protects children from asthma development. *Nat Med*. Nature Publishing Group; 2019; doi: 10.1038/s41591-019-0469-4.

78. Alarcón LV, Alberto AA, Mateu E. Biosecurity in pig farms: a review. *Porcine Health Manag*. 2021; doi: 10.1186/s40813-020-00181-z.

79. Coffman VR, Hall DJ, Pisanic N, Nadimpalli M, McCormack M, Diener-West M, et al.. Personal protective equipment use during industrial hog operation work activities and acute lung function changes in a prospective worker cohort, North Carolina 2014–2015. *American Journal of Industrial Medicine*. 2021; doi: 10.1002/ajim.23260.

80. Slizovskiy IB, Oliva M, Settle JK, Zyskina LV, Prosperi M, Boucher C, et al.. Target-enriched long-read sequencing (TELSeq) contextualizes antimicrobial resistance genes in metagenomes. *Microbiome*. 2022; doi: 10.1186/s40168-022-01368-y.

81. Slizovskiy IB, Bonin N, Bravo JE, Ferm PM, Singer J, Boucher C, et al.. Factors impacting target-enriched long-read sequencing of resistomes and mobilomes. *Genome Res*. 2024; doi: 10.1101/gr.279226.124.

82. Bjerre RD, Hugerth LW, Boulund F, Seifert M, Johansen JD, Engstrand L. Effects of sampling strategy and DNA extraction on human skin microbiome investigations. *Sci Rep*. Nature Publishing Group; 2019; doi: 10.1038/s41598-019-53599-z.

83. Byrd AL, Belkaid Y, Segre JA. The human skin microbiome. *Nat Rev Microbiol*. 2018; doi: 10.1038/nrmicro.2017.157.

84. Human Microbiome Project Consortium. Structure, function and diversity of the healthy human microbiome. *Nature*. 2012; doi: 10.1038/nature11234.

85. Gohl DM, Vangay P, Garbe J, MacLean A, Hauge A, Becker A, et al.. Systematic improvement of amplicon marker gene methods for increased accuracy in microbiome studies. *Nat Biotechnol*. Nature Publishing Group; 2016; doi: 10.1038/nbt.3601.

86. Doster E, Lakin SM, Dean CJ, Wolfe C, Young JG, Boucher C, et al.. MEGARes 2.0: a database for classification of antimicrobial drug, biocide and metal resistance determinants in metagenomic sequence data. *Nucleic Acids Res*. Oxford Academic; 2020; doi: 10.1093/nar/gkz1010.

87. Liu M, Li X, Xie Y, Bi D, Sun J, Li J, et al.. ICEberg 2.0: an updated database of bacterial integrative and conjugative elements. *Nucleic Acids Res*. 2019; doi: 10.1093/nar/gky1123.

88. Carattoli A, Zankari E, García-Fernández A, Voldby Larsen M, Lund O, Villa L, et al.. In Silico Detection and Typing of Plasmids using PlasmidFinder and Plasmid Multilocus Sequence Typing. *Antimicrob Agents Chemother*. 2014; doi: 10.1128/AAC.02412-14.

89. Metsky HC, Siddle KJ, Gladden-Young A, Qu J, Yang DK, Brehio P, et al.. Capturing sequence diversity in metagenomes with comprehensive and scalable probe design. *Nature Biotechnology*. Nature Publishing Group; 2019; doi: 10.1038/s41587-018-0006-x.

90. Martin M. Cutadapt removes adapter sequences from high-throughput sequencing reads. *EMBnet.journal*. 2011; doi: 10.14806/ej.17.1.200.

91. Callahan BJ, McMurdie PJ, Rosen MJ, Han AW, Johnson AJA, Holmes SP. DADA2: High-resolution sample inference from Illumina amplicon data. *Nature Methods*. Nature Publishing Group; 2016; doi: 10.1038/nmeth.3869.

92. Quast C, Pruesse E, Yilmaz P, Gerken J, Schweer T, Yarza P, et al.. The SILVA ribosomal RNA gene database project: improved data processing and web-based tools. *Nucleic Acids Research*. 2013; doi: 10.1093/nar/gks1219.

93. Davis NM, Proctor DM, Holmes SP, Relman DA, Callahan BJ. Simple statistical identification and removal of contaminant sequences in marker-gene and metagenomics data. *Microbiome*. 2018; doi: 10.1186/s40168-018-0605-2.

94. Bolger AM, Lohse M, Usadel B. Trimmomatic: a flexible trimmer for Illumina sequence data. *Bioinformatics*. 2014; doi: 10.1093/bioinformatics/btu170.

95. Li H. Aligning sequence reads, clone sequences and assembly contigs with BWA-MEM. arXiv;

96. Li H, Handsaker B, Wysoker A, Fennell T, Ruan J, Homer N, et al.. The Sequence Alignment/Map format and SAMtools. *Bioinformatics*. 2009; doi: 10.1093/bioinformatics/btp352.

97. Slizovskiy IB, Mukherjee K, Dean CJ, Boucher C, Noyes NR. Mobilization of antibiotic resistance: Are current approaches for colocalizing resistomes and mobilomes useful? *Front Microbiol*. Frontiers; 2020; doi: 10.3389/fmicb.2020.01376.
98. Leplae R, Lima-Mendez G, Toussaint A. ACLAME: a CLAssification of Mobile genetic Elements, update 2010. *Nucleic Acids Res*. 2010; doi: 10.1093/nar/gkp938.
99. Siguier P, Perochon J, Lestrade L, Mahillon J, Chandler M. ISfinder: the reference centre for bacterial insertion sequences. *Nucleic Acids Res*. 2006; doi: 10.1093/nar/gkj014.
100. Kichenaradja P, Siguier P, Pérochon J, Chandler M. ISbrowser: an extension of ISfinder for visualizing insertion sequences in prokaryotic genomes. *Nucleic Acids Research*. 2010; doi: 10.1093/nar/gkp947.
101. Apweiler R, Bairoch A, Wu CH, Barker WC, Boeckmann B, Ferro S, et al.. UniProt: the Universal Protein knowledgebase. *Nucleic Acids Res*. 2004; doi: 10.1093/nar/gkh131.
102. Doster E, Rovira P, Noyes NR, Burgess BA, Yang X, Weinroth MD, et al.. Investigating Effects of Tulathromycin Metaphylaxis on the Fecal Resistome and Microbiome of Commercial Feedlot Cattle Early in the Feeding Period. *Front Microbiol*. 2018; doi: 10.3389/fmicb.2018.01715.
103. Bates D, Mächler M, Bolker B, Walker S. Fitting Linear Mixed-Effects Models Using lme4. *Journal of Statistical Software*. 2015; doi: 10.18637/jss.v067.i01.
104. Lenth R, Love J, Herve M. emmeans: Estimated Marginal Means, aka Least-Squares Means.
105. Xu S, Zhan L, Tang W, Wang Q, Dai Z, Zhou L, et al.. MicrobiotaProcess: A comprehensive R package for deep mining microbiome. *The Innovation*. 2023; doi: 10.1016/j.xinn.2023.100388.
106. Quinn TP, Erb I, Gloor G, Notredame C, Richardson MF, Crowley TM. A field guide for the compositional analysis of any-omics data. *Gigascience*. 2019; doi: 10.1093/gigascience/giz107.
107. Palarea-Albaladejo J, Martín-Fernández JA. zCompositions — R package for multivariate imputation of left-censored data under a compositional approach. *Chemometrics and Intelligent Laboratory Systems*. 2015; doi: 10.1016/j.chemolab.2015.02.019.
108. Oksanen J. Vegan: an introduction to ordination. :122019;
109. Li B, Yang Y, Ma L, Ju F, Guo F, Tiedje JM, et al.. Metagenomic and network analysis reveal wide distribution and co-occurrence of environmental antibiotic resistance genes. *ISME J*. 2015; doi: 10.1038/ismej.2015.59.
110. Love MI, Huber W, Anders S. Moderated estimation of fold change and dispersion for RNA-seq data with DESeq2. *Genome Biology*. 2014; doi: 10.1186/s13059-014-0550-8.
111. Stephens M. False discovery rates: a new deal. *Biostatistics*. 2017; doi: 10.1093/biostatistics/kxw041.
112. Kurtz ZD, Müller CL, Miraldi ER, Littman DR, Blaser MJ, Bonneau RA. Sparse and Compositionally Robust Inference of Microbial Ecological Networks. *PLOS Computational Biology*. Public Library of Science; 2015; doi: 10.1371/journal.pcbi.1004226.
113. Meinshausen N, Bühlmann P. High dimensional graphs and variable selection with the LASSO. *The Annals of Statistics*. 2006; doi: 10.1214/009053606000000281.
114. Müller CL, Bonneau R, Kurtz Z. Generalized Stability Approach for Regularized Graphical Models. arXiv;
115. Csardi G, Nepusz T. The Igraph Software Package for Complex Network Research. *InterJournal*. Complex Systems:16952005;
116. Reichardt J, Bornholdt S. Statistical Mechanics of Community Detection. *Phys Rev E*. 2006; doi: 10.1103/PhysRevE.74.016110.
117. Newman MEJ, Girvan M. Finding and evaluating community structure in networks. *Phys Rev E*. American Physical Society; 2004; doi: 10.1103/PhysRevE.69.026113.
118. Beghini F, McIver LJ, Blanco-Míguez A, Dubois L, Asnicar F, Maharjan S, et al.: Integrating taxonomic, functional, and strain-level profiling of diverse microbial communities with bioBakery 3. eLife. eLife Sciences Publications Limited; https://elifesciences.org/articles/65088/figures (2021). Accessed 2023 Apr 4.
119. Langmead B, Salzberg SL. Fast gapped-read alignment with Bowtie 2. *Nat Methods*. Nature

Publishing Group; 2012; doi: 10.1038/nmeth.1923.

120. Li D, Liu C-M, Luo R, Sadakane K, Lam T-W. MEGAHIT: an ultra-fast single-node solution for large and complex metagenomics assembly via succinct de Bruijn graph. *Bioinformatics*. 2015; doi: 10.1093/bioinformatics/btv033.

121. Kang DD, Li F, Kirton E, Thomas A, Egan R, An H, et al.. MetaBAT 2: an adaptive binning algorithm for robust and efficient genome reconstruction from metagenome assemblies. *PeerJ*. 2019; doi: 10.7717/peerj.7359.

122. Olm MR, Brown CT, Brooks B, Banfield JF. dRep: a tool for fast and accurate genomic comparisons that enables improved genome recovery from metagenomes through de-replication. *ISME J*. 2017; doi: 10.1038/ismej.2017.126.

123. Parks DH, Imelfort M, Skennerton CT, Hugenholtz P, Tyson GW. CheckM: assessing the quality of microbial genomes recovered from isolates, single cells, and metagenomes. *Genome Res*. 2015; doi: 10.1101/gr.186072.114.

124. Chaumeil P-A, Mussig AJ, Hugenholtz P, Parks DH. GTDB-Tk v2: memory friendly classification with the genome taxonomy database. *Bioinformatics*. 2022; doi: 10.1093/bioinformatics/btac672.

125. Glendinning L, Stewart RD, Pallen MJ, Watson KA, Watson M. Assembly of hundreds of novel bacterial genomes from the chicken caecum. *Genome Biology*. 2020; doi: 10.1186/s13059-020-1947-1.

126. : IQ-TREE 2: New Models and Efficient Methods for Phylogenetic Inference in the Genomic Era | Molecular Biology and Evolution | Oxford Academic. https://academic.oup.com/mbe/article/37/5/1530/5721363 Accessed 2023 Mar 27.

127. : Interactive Tree Of Life (iTOL) v5: an online tool for phylogenetic tree display and annotation | Nucleic Acids Research | Oxford Academic. https://academic.oup.com/nar/article/49/W1/W293/6246398 Accessed 2023 Mar 27.

Click here to access/download
Supplementary Material
Supplementary_datafile_10_DESeq_results_resistome.xl
sx

Click here to access/download
Supplementary Material
Supplementary_datafile_11_DESeq_results_mobilome.xlsx

Click here to access/download
Supplementary Material
Supplementary_datafile_12_MAG_metadata.csv

Click here to access/download
Supplementary Material
Supplementary_Materials_Revised.docx

Supplementary_datafile_1

Click here to access/download
Supplementary Material
Supplementary_datafile_1_metadata.csv

Click here to access/download
Supplementary Material
Supplementary_datafile_2_microbiome_counts.csv

Supplementary_datafile_3

Click here to access/download
Supplementary Material
Supplementary_datafile_3_microbiome_taxonomy.csv

Supplementary_datafile_4

Click here to access/download
Supplementary Material
Supplementary_datafile_4_DESeq.xlsx

Click here to access/download
Supplementary Material
Supplementary_datafile_5_resistome_counts.csv

Supplementary_datafile_6

Click here to access/download
Supplementary Material
Supplementary_datafile_6_resistome_annotation.csv

Click here to access/download
Supplementary Material
Supplementary_datafile_7_mobilome_counts.csv

Click here to access/download
Supplementary Material
Supplementary_datafile_8_mobilome_annotation.csv

Click here to access/download
Supplementary Material
Supplementary_datafile_9_StrainPhlan.csv

Cover_Letter Click here to access/download;Personal Cover;CoverLetter.docx

February 26th, 2025

Dear editorial board,

Thank you for considering a revised version of our manuscript "*Reducing Skin Microbiome Exposure Impacts Through Swine Farm Biosecurity*" for publication in *GigaScience*.

We thank the editor and two reviewers for their comprehensive evaluation of our work. By addressing these well-considered comments, we believe the paper has been substantially strengthened, and we thank the reviewers and editor for their suggestions. Updating our analyses to use the most current versions of databases also strengthened the results and brought them in-line with the most current taxonomic labels and reference databases. The use of multiple statistical models for key analyses further bolstered our findings. Notably, these important bioinformatic and analytical updates did not alter the results of the study, highlighting the robustness of the biological and epidemiological conclusions.

This work thus provides fundamental knowledge about the microbiome and antimicrobial resistance dynamics of a relatively under-studied human worker population. Importantly, this population also plays an important interfacing role between livestock populations and rural communities. As such, we believe our work will be of great interest to a wide readership.

This manuscript has not been previously published and is not under review at any other journal. All listed authors have contributed significantly to this research based on the requirement for authorship guidelines and declare no conflicts of interest. The study from which data was generated was conducted in accordance with University of Minnesota and U.S. federal guidelines pertaining to human subject investigations, including the acquisition of informed consent following the review of study details and expectations with each participant. Steps to ensure security of any associated personal identifying information were taken. All raw sequencing data and metadata will be made accessible via the Sequenced Read Archive (SRA) under BioProject PRJNA987158. All statistical scripts are made publicly available at https://github.com/IS233489/LaborOME-project.

Thank you for your consideration of our submitted manuscript.

Sincerely,

Dr. Noelle Noyes, MA, DVM, PhD
Associate Professor
University of Minnesota
1988 Fitch Avenue, St. Paul, MN, 55108
Office: 612-624-3562, Mobile: 617-953-7837
