# Supplementary material for: Reducing skin microbiome exposure impacts through swine farm biosecurity
Source: Gigascience. 2025 Jul 26;14:giaf062. doi: 10.1093/gigascience/giaf062 (PMC12810053; doi:10.1093/gigascience/giaf062)
Supplement: giaf062_GIGA-D-24-00356_Revision_2 [file giaf062_giga-d-24-00356_revision_2.pdf]

GigaScience

Reducing Skin Microbiome Exposure Impacts Through Swine Farm Biosecurity

--Manuscript Draft--

**Manuscript Number:**	GIGA-D-24-00356R2
**Full Title:**	Reducing Skin Microbiome Exposure Impacts Through Swine Farm Biosecurity
**Article Type:**	Research
**Funding Information:**	Division of Intramural Research, National Institute of Allergy and Infectious Diseases (1R01AI141810-01) — Dr Christina Boucher<br>National Institute for Occupational Safety and Health (T42 OH008434) — Dr Ilya B Slizovskiy<br>National Institute for Occupational Safety and Health (2 U54OH010170-11-00) — Dr Noelle R Noyes
**Abstract:**	Livestock work is unique due to worker exposure to animal-associated microbiomes within the workplace. Swine workers are a unique cohort within the United States livestock labor force, as they have direct daily contact with pigs and undertake mandatory biosecurity interventions. However, investigating this occupational cohort is challenging, particularly within tightly regulated commercial swine operations. Thus, little is known about the impacts of animal exposure and biosecurity protocols on the swine worker microbiome. We obtained unique samples from United States swine workers, using a longitudinal study design to investigate temporal microbiome dynamics. We observed a significant increase in bacterial DNA load on worker skin during the workday, with concurrent changes in the composition and abundance of microbial taxa, resistance genes and mobile genetic elements. However, mandatory showers at the end of the workday partially returned the skin's microbiome and resistome to their original state. These novel results from a human cohort demonstrate that existing biosecurity practices ameliorate work-associated microbiome impacts.
**Corresponding Author:**	Noelle Noyes<br>University of Minnesota College of Veterinary Medicine: University of Minnesota Twin Cities College of Veterinary Medicine<br>UNITED STATES
**Corresponding Author Secondary Information:**	
**Corresponding Author's Institution:**	University of Minnesota College of Veterinary Medicine: University of Minnesota Twin Cities College of Veterinary Medicine
**Corresponding Author's Secondary Institution:**	
**First Author:**	Ilya B Slizovskiy, DVM, PhD, MPH
**First Author Secondary Information:**	
**Order of Authors:**	Ilya B Slizovskiy, DVM, PhD, MPH
	Tara N Gaire, DVM, PhD
	Peter M Ferm, M.Sc.
	Carissa A Odland, DVM, M.Sc.
	Scott A Dee, DVM, PhD
	Joel Nerem, DVM, M.Sc.
	Jonathan E Bravo, M.Sc.
	Alejandro D. Kimball
	Christina Boucher, PhD

Powered by Editorial Manager® and ProduXion Manager® from Aries Systems Corporation

	Noelle R Noyes, DVM, PhD
**Order of Authors Secondary Information:**	
**Response to Reviewers:**	RE: GIGA-D-24-00356<br>Reducing Skin Microbiome Exposure Impacts Through Swine Farm Biosecurity<br>Ilya B. Slizovskiy; Tara N Gaire; Peter M Ferm; Carissa A Odland; Scott A Dee; Joel Nerem; Jonathan E Bravo; Christina Boucher; Noelle R Noyes<br><br>Dear Editor and Reviewers,<br><br>We thank you for your thorough and thoughtful reviews of our manuscript "Reducing Skin Microbiome Exposure Impacts Through Swine Farm Biosecurity" (GIGA-D-24-00356). We appreciate the results of the second round of review, and have included here our comments and changes commensurate with the reviewer's minor revision comments.<br>Thank you again for your time and insightful suggestions.<br><br>Editorial comment- Please also ensure that your revised manuscript conforms to the journal style, which can be found in the Instructions for Authors on the journal homepage. (For example, please structure the abstract, with sections "Background", "Results", "Conclusions").<br>Response: We thank the editor for their comment. We revisited the formatting instructions and ensured that our manuscript conforms to the GigaScience publishing format standards, including the changes made to the abstract section. Additionally, we added all in-text references and citations related to the GigaDB archival process of supporting datasets and sequencing information.<br><br>Reviewer #1:The article titled "Reducing Skin Microbiome Exposure Impacts Through Swine Farm Biosecurity" has improved considerably. I appreciate the authors' efforts in addressing the comments, particularly updating the 16S rRNA database. I have only a few minor suggestions that should be addressed. Below are the detailed comments:<br><br>General comments:<br>- Comment 1. Despite the improvements, there are still instances (e.g., line 78) where there is no space between the citation and the preceding text. This is especially noticeable in the Materials and Methods section. Please review thoroughly and ensure proper spacing throughout.<br>Response: We noted this issue, and made changes to in-text citation spacing throughout the manuscript. We believe we have made corrections throughout all facets of the manuscript now.<br><br>Abstract:<br>- Comment 2. Line 47. Include the abbreviation for "United States" for further use in Line 52.<br>Response: We made this change in the abstract section.<br><br>Introduction:<br>- Comment 3. Line 81. Please replace "Actinobacteria" and "Bacterioidetes" with the updated phylum names "Actinomycetota" and "Bacteroidota," respectively.<br>Response: We have made this suggested change, in ~line 80, first paragraph of the Introduction.<br><br>Results:<br>- Comment 4. Figure 5c. Ensure that the updated phylum name for "Proteobacteria" is reflected here (i.e., "Pseudomonadota"), as previously recommended for a fully up-to-date classification.<br>Response: We have made the suggested change to this figure.<br><br>Reviewer #2: The authors have done a thorough job to update their analysis with more recent versions of databases, tools and statistics. It was good to see that beside some smaller changes the major results did not change. This is also comforting for earlier studies that used those tools and databases.<br><br>While reviewing the revision;

	- The answers to queries and suggestions seem to be incorporated well in the revised text and figures.<br><br>- I noticed the strain level addition of S suis detection on page 10. Since the authors have the strain level information, it is important to mention which so-called serotypes were detected since not all S suis serotypes are zoonotic.<br>Response: We thank the reviewer for raising the issue of S. suis serotyping. We added a clarification that detection of S. suis strains is based on the gene marker strategy in lines 379–380, page 12. Given the approach used, we do not find it possible to comment on differentiation of S. suis serotypes, as the gene marker approach is not conducive to such analysis.
**Additional Information:**	
**Question**	**Response**
Are you submitting this manuscript to a special series or article collection?	No
**Experimental design and statistics**<br><br>Full details of the experimental design and statistical methods used should be given in the Methods section, as detailed in our Minimum Standards Reporting Checklist. Information essential to interpreting the data presented should be made available in the figure legends.<br><br>Have you included all the information requested in your manuscript?	Yes
**Resources**<br><br>A description of all resources used, including antibodies, cell lines, animals and software tools, with enough information to allow them to be uniquely identified, should be included in the Methods section. Authors are strongly encouraged to cite Research Resource Identifiers (RRIDs) for antibodies, model organisms and tools, where possible.<br><br>Have you included the information requested as detailed in our Minimum Standards Reporting Checklist?	Yes
**Availability of data and materials**	Yes

All datasets and code on which the conclusions of the paper rely must be either included in your submission or deposited in publicly available repositories (where available and ethically appropriate), referencing such data using a unique identifier in the references and in the "Availability of Data and Materials" section of your manuscript.<br><br>Have you have met the above requirement as detailed in our Minimum Standards Reporting Checklist?	

Powered by Editorial Manager® and ProduXion Manager® from Aries Systems Corporation

Manuscript
Click here to access/download;Manuscript;Manuscript_Revised2.docx

Reducing Skin Microbiome Exposure Impacts Through Swine Farm Biosecurity

***Ilya B. Slizovskiy*[1,2,3], *Tara N. Gaire*[3], *Peter M. Ferm*[3], *Carissa A. Odland*[4], *Scott A. Dee*[5], *Joel Nerem*[5], *Jonathan E. Bravo*[6], *Alejandro D. Kimball*[7], *Christina Boucher*[6], *Noelle R. Noyes**[3]**

Purdue Applied Microbiome Sciences Program, Purdue University, West Lafayette, IN, USA[1]
Veterinary Clinical Sciences Department, College of Veterinary Medicine, Purdue University, West Lafayette, IN, USA[2]
Food-Centric Corridor, Infectious Disease Laboratory, Department of Veterinary Population Medicine, College of Veterinary Medicine, University of Minnesota, St. Paul, MN, USA[3]
Pipestone Veterinary Services, Pipestone, MN, USA[4]
Pipestone Applied Research, Pipestone, MN, US[5]
Department of Computer and Information Science and Engineering, Herbert Wertheim College of Engineering, University of Florida, Gainesville, FL, USA[6]
Regenstrief Center for Healthcare Engineering, Purdue University, West Lafayette, IN, USA[7]

To whom correspondence shall be addressed:

Dr. Noelle R. Noyes
nnoyes@umn.edu
385D AnSci/VM
1988 Fitch Avenue
St. Paul, MN 55108
United States

***Running head*:**

Farm biosecurity shapes microbiomes

Study funding:

Financial support for this work was provided by the National Institute of Health (NIH) National Institute of Allergy and Infectious Disease (NIAID), *Project No. 1R01AI141810-01*; the Midwest Center for Occupational Safety and Health (MCOHS) Pilot Projects Research Training Program (PPRTP) funded through the National Institute of Occupational Safety and Health (NIOSH), *Project No.* T42 OH008434; and the University of Minnesota Doctoral Dissertation Fellowship.

SUPPLEMENTARY FIGURES

SUPPLEMENTARY DATAFILES

ABSTRACT

Background: Livestock work is unique due to worker exposure to animal-associated microbiomes within the workplace. Swine workers are a unique cohort within the United States (U.S.) livestock labor force, as they have direct daily contact with pigs and undertake mandatory biosecurity interventions. However, investigating this occupational cohort is challenging, particularly within tightly regulated commercial swine operations. Thus, little is known about the impacts of animal exposure and biosecurity protocols on the swine worker microbiome. We obtained unique samples from U.S. swine workers, using a longitudinal study design to investigate temporal microbiome dynamics.

Results: We observed a significant increase in bacterial DNA load on worker skin during the workday, with concurrent changes in the composition and abundance of microbial taxa, resistance genes and mobile genetic elements. However, mandatory showering at the end of the workday partially returned the skin's microbiome and resistome to their original state.

Conclusions: These novel results from a human cohort demonstrate that existing biosecurity practices can ameliorate work-associated microbiome impacts.

KEY WORDS

Metagenomics; Microbiome; Antimicrobial resistance; Mobile genetic elements; Farm workers; Public health

INTRODUCTION

Occupational exposures can significantly influence the microbiomes of workers, and in some cases have been linked to health outcomes [1,2]. People working with animals encounter a unique workplace microbiome with frequent exposure to animal microbiomes, either through direct contact or indirect exposures. The impact of animal exposure on human microbiomes has been demonstrated across several settings including research facilities [3,4] and livestock farms [5], as well as within homes [6]. For example, dairy and swine workers have more diverse oral and nasal bacterial taxa than non-livestock workers [7]. Additionally, the skin microbiome of livestock workers harbors a higher relative abundance of Pseudomonadota and lower relative abundance of Actinomycetota and Bacteroidota compared to people with non-livestock occupations [8]. Livestock-associated bacteria and their antimicrobial resistance genes (ARGs) have been documented in agricultural worker cohorts, including farmers [9–11], veterinarians [12,13] , and abattoir workers [10,14] ; and short-term visitation to swine farms has been linked to an enrichment in farm-associated bacteria and ARGs in the human gut [5]. These findings have been attributed to livestock exposure, but few studies have actually tracked daily animal exposure and on-site worker behavior, particularly on commercial farms [15,16]. Such studies require careful consideration of workplace habits and exposures, and sampling must occur within the constraints of commercial livestock production. Given these challenges, detailed studies of livestock worker microbiomes are uncommon, and the specific influence of occupational exposures on livestock worker microbiomes remains poorly understood.

In the United States (U.S.), swine workers are a unique cohort within the livestock labor force. Their job tasks involve intensive one-on-one animal handling, working within enclosed and climatically regulated facilities, with work duration ranging 48–54 hours per week [17,18]. These working conditions contrast markedly with other production systems; beef cattle and poultry are rarely handled by workers, and cattle work tends to occur in open-air facilities. Moreover, most workers in North American swine farms adhere to strict biosecurity measures to control pathogen transmission between and within farms [19,20], including showering into and out of the farm, which may reduce transfer of microbes and ARGs between swine farms and the general public. However, the impact of mandatory showering on the likelihood of worker-mediated farm-to-community transmission is unknown.

We report on a longitudinal investigation of the swine worker microbiome-resistome on a commercial U.S. swine facility, with sampling occurring as part of a normal workday that included showering as a mandatory biosecurity intervention (**Figure 1**). We observed a significant increase in bacterial DNA load on worker skin during the workday, with concurrent changes in the composition and abundance of specific microbial taxa, ARGs, and mobile genetic elements (MGEs) that could harbor ARGs. We further observed that compulsory showering at the end of the workday reverted the skin microbiome and resistome to a baseline state, which differs from previous results that did not include showering in the study design [5] . These results suggest that occupational work in swine facilities can significantly impact workers' skin microbiomes, but that these impacts can be transient if biosecurity interventions are implemented. The relevancy of these findings for short- and long-term swine worker health requires further study. However, the observation that showering may dampen daily microbiome impacts have important public health implications, as it demonstrates that biosecurity protocols could be leveraged to minimize microbial transmission from farms to general communities.

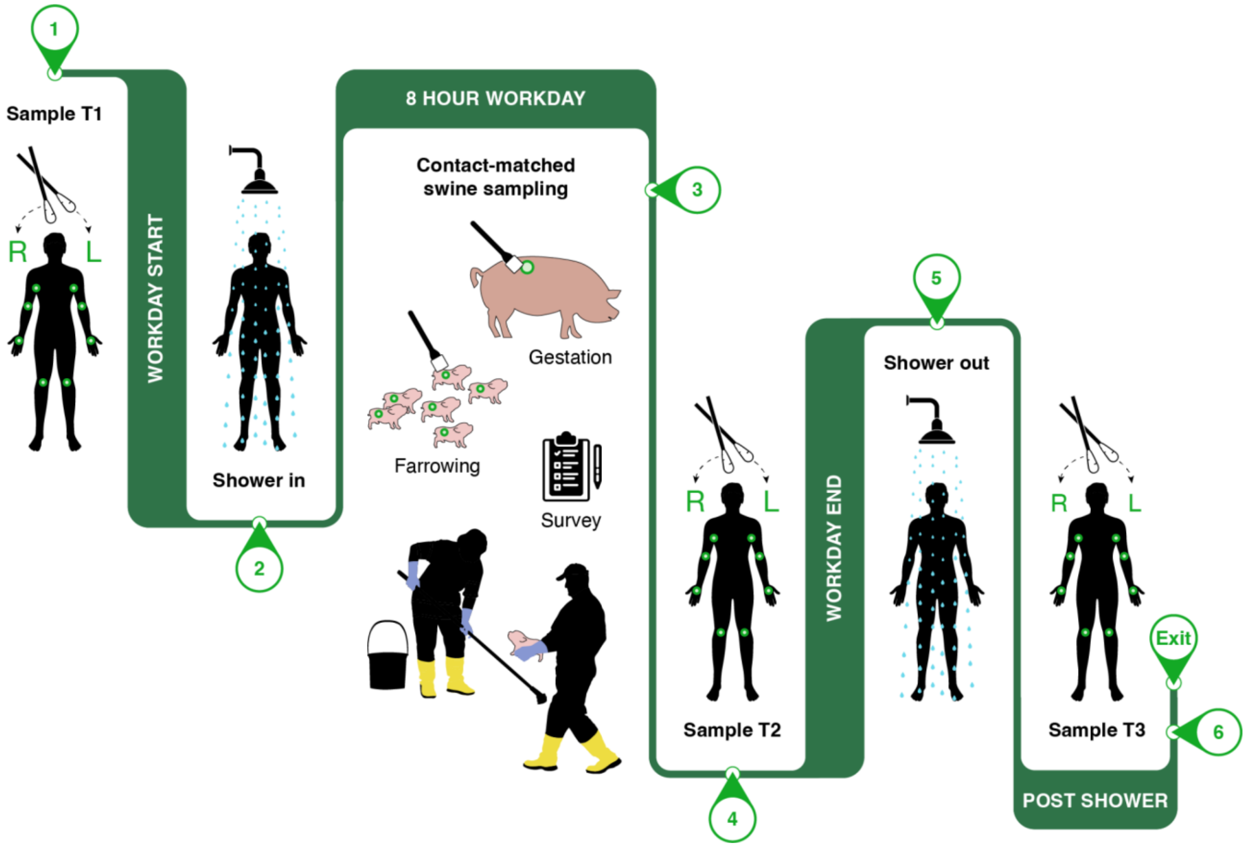

Figure 1. Study overview. Farm workers from a commercial farrow-to-wean operation in the Midwestern United States were voluntarily enrolled into a single longitudinal microbiome sampling campaign during a typical 8-hour workday shift. For each worker, swab kits were used to self-collect samples from the epidermis in a standardized fashion by passing each swab across four body sites, achieving a single composite skin sample for the left and right body representing microbiomes from the manus, interdigital space, antecubital fossa, popliteal fossa, and axilla. Workers were asked to perform the first self-collection ('Sample **T1**') prior to entry into the swine facility (**1**). Workers underwent mandatory showering prior to entry into the animal holding areas (**2**). During the day shift, workers were observed handling animals or working in specific animal pens, and dorsal skin swabs (from withers to tail-base) were taken from contact-matched animals on a pooled multi-pen level. Additionally, a 15-minute questionnaire was administered to collect biometric, health, lifestyle, and occupational task performance information from each worker (**3**). In a similar fashion, self-collected skin samples were taken immediately upon conclusion of the workday ('Sample **T2**') (**4**). Workers underwent mandatory showering procedures immediately after exiting the animal holding areas (**5**) and a third self-collection of samples was performed ('Sample **T3**') after showering and immediately prior to exiting the farm facility (**6**).

RESULTS

Individual skin microbiomes experience dramatic yet transient shifts during on-farm work with swine

16S rRNA sequencing and analysis was performed on 40 skin samples collected longitudinally from 10 healthy and predominantly male swine workers and contact-matched swine (SI Appendix SI Methods, **Supplementary note 1**, **Supplementary file 1**). The skin microbiome composition underwent significant shifts in the course of a single 8-hour workday (ANOSIM $p<$ 0.001; R=57.6%, adjusted PERMANOVA $p<$ 0.001, **Figure 2a**). Specifically, samples taken at the end of work but prior to showering (T2) had a significantly different composition than

samples taken at the beginning of work (T1) (pairwise R^2=22.0%; FDR adjusted p=0.004); and at the end of the workday following showering (T3) the microbiome underwent yet another shift relative to T2 (pairwise R^2=18.6%; FDR adjusted p=0.004). However, the microbiome at T3 was not significantly different than at T1, suggesting at least a partial reversion of the skin microbial composition after showering (pairwise R^2=4.9%; FDR adjusted p=0.12). These shifts corresponded with changes in skin-borne bacterial load as quantified using 16S gene concentration (copies/µl, **Figure 2b**). Specifically, 16S gene concentration increased by ~200-fold from T1 to T2, but then decreased back to baseline levels at T3 (Type III ANOVA $p<$ 0.0001 with Tukey's *post hoc* analysis and adjustment for FDR). There were no significant differences in average sequencing depth, sequencing quality and taxonomic discovery rates between the three collection time points, suggesting that these technical factors did not significantly bias comparisons across collection phases (**Supplementary note 2**, **Supplementary figures 1, 2**).

We next assessed associations between crude swine exposure rates and 16S gene concentration, i.e., bacterial load. There was large variation in estimated hourly swine exposure rates (**Supplementary table 1**), and the hourly exposure density was found to be inversely correlated with bacterial load (Estimate[SE]= -1.23[0.44], *glm* p=0.01, **Figure 2c**). This relationship persisted at T2 and T3 (**Figure 2c**). Though more granular and systematic exposure assessments are needed, these patterns suggest that differences in swine exposure density may be a proxy for different workday tasks that ultimately dictate levels of microbial biomass acquisition. For example, tasks such as feeding, health-checks, and decontamination require walking through swine holding rooms, but involve very little direct interfacing with animals and their byproducts; such tasks would be classified as "high density", but in reality there may be less opportunity for direct acquisition of swine-related microbes. In contrast, activities such as vaccinating and obstetrical management require prolonged contact with individual pigs, but not necessarily moving through multiple swine holding rooms; thus, such tasks may have lower density but more opportunities for acquisition of swine-sourced microbes through direct contact.

A total of 6,840 unique amplicon sequence variants (ASVs) representing 356 distinct genera were recovered across all worker skin samples (**Supplemental figure 2, Supplementary datafile 2,3**). The ASV diversity and the dominant phyla were consistent with findings from previous microbiome studies of human skin [21–23]. The relative abundances of dominant phyla remained largely similar across T1–T3, with the exception of Cyanobacteriota, which were more abundant on skin at T2 versus T1 and T3 (**Supplemental figure 3a-b**). Phylum-level and genus-level richness and evenness of the worker skin microbiome remained unchanged throughout the course of the day, and did not significantly differ from contact-matched swine skin samples nor the environmental samples (Type III ANOVA adjusted $p>$ 0.1, **Supplemental figure 4a-d**). More than 400 genera were detected in at least one sample from each of the pairwise collection phases under comparison (T1 vs. T2 [n=76], T2 vs. T3 [n= 76], and T1 vs. T3 [n=75]), and fewer than 13% of these genera exhibited significant changes in relative abundance over the three timepoints (**Supplementary datafile 4**, **Supplementary figure 5**). The relative abundances of *Methanobrevibacter*, *Negativibacillus*, *Butyricicoccus*, *Agathobacter*, and *Lachnospiraceae UCG-010* were significantly higher in T2 versus T1 samples. These genera inhabit mammalian oral cavity and digestive tracts. Genera with significantly higher relative abundance in T3 compared to T2 skin samples included taxa also primarily found as gastrointestinal microbiota.

These include relatively new and unclassified rumenal genera such as *UCG-005(Oscillospiraceae) and Candidatus Soleaferrea (Ruminococcaceae)* that have been previously reported in swine intestinal microbiomes but not functionally described [24,25], and *CHKCI001 (Lachnospiraceae)* which to our knowledge has hitherto been only described in chicken intestinal microbiomes [26,27]. Other differentially abundant genera at T3 relative to T2 include *Agathobacter* which exhibited the largest fold-change increase in relative abundance, and was also found at a significantly greater relative abundance at T2 vs T1 (**Supplementary datafile 4**). *Agathobacter* is a fiber-degrading and butyrate producing keystone genus that has been described in growing piglets [28,29]. We noted that a mixed population of genera were differentially abundant at T3 relative to T1, including intestinal genera such as *Negativibacillus* which accounted for the greatest fold-change at T2 relative to T1, *Fastidiosipila*, a poorly characterized genus of methanogenic anaerobic bacteria [30], and highly ubiquitous environmental bacteria Brevundimonas.

The skin microbiome becomes unstructured and dominated by enteric and environmental microbes during on-farm work

Inferred association networks were explored using a compositional modeling approach to describe the topology and connectivity of microbial constituents in worker skin microbiomes across collection phases and in comparison to contact-matched animals (SI Appendix, SI Methods). Networks were constructed using standard cutoffs, and ASVs with >100 counts present in >10% sample prevalence were included, which represented 4.1–5.7% of all ASVs used as input into network generation, depending on the collection phase. The resulting networks (one per collection phase) were each composed of a singular interconnected component, with the most connected network being T1 and the most sparse T2 (**Figure 2d**).

Given the significant differences in network topology across T1, T2 and T3 (**Supplementary note 4**), we further analyzed each network to identify the most dominant and interconnected genera (i.e., keystone taxa), as indicated by high eigenvector centrality and high node degree. At T1, keystone genera included a mixed population of bacteria not typically considered ubiquitous inhabitants of the human skin microbiome [31], including usually enteric members such as *Clostridium* and *Mediterraneibacter* and environmental bacteria such as *Blastococcus* and *Cryobacterium*. Other keystone genera included human skin commensals like *Anaerococcus* and *Fusobacterium*. However, *Corynebacterium,* well-known as a ubiquitous inhabitant of both swine and human skin, was among the least influential members of the skin microbiome (**Figure 2e**). Conversely, at T2, the interactions and modular domains within the skin microbiota became notably sparse, with *Corynebacterium* genera by far the most extensively represented among the keystone bacteria [21,32,33] (**Figure 2e**). The least influential bacteria at T2 were typically enteric bacteria characteristic of mammalian intestinal microbiota, including *Clostridium, Rothia*, *Kocuria*, and *Terrisporobacter*. After showering and prior to exit from the swine facility (T3), dominant keystone genera were an admixture of Actinobacteria and Clostridia classes, encompassing genera associated with the mammalian gastrointestinal tract including *Sellimonas, Rothia, Mediterraneibacter,* UCG-005; as well as commensal human genera such as *Peptoniphilus* and Family XI of the Peptostreptococcales-Tissierellales Order, previously identified among medically important inhabitants of human axillae [34]. We note that the swine

skin ecological network was composed of dominant keystone genera *Fusobacterium*, *Schaalia*, and *Akkermansia*, typically associated with skin, oral mucous membrane, and intestinal microbiota. Notably, *Fusobacterium* in swine corresponded with the same genus found among keystone organisms at T1 in swine workers collected prior to entry into the main animal holding areas, while the least interconnected swine skin genera of *Corynebacterium* were observed in worker skin ecosystem dominance at T2, collected at the end of the workday.

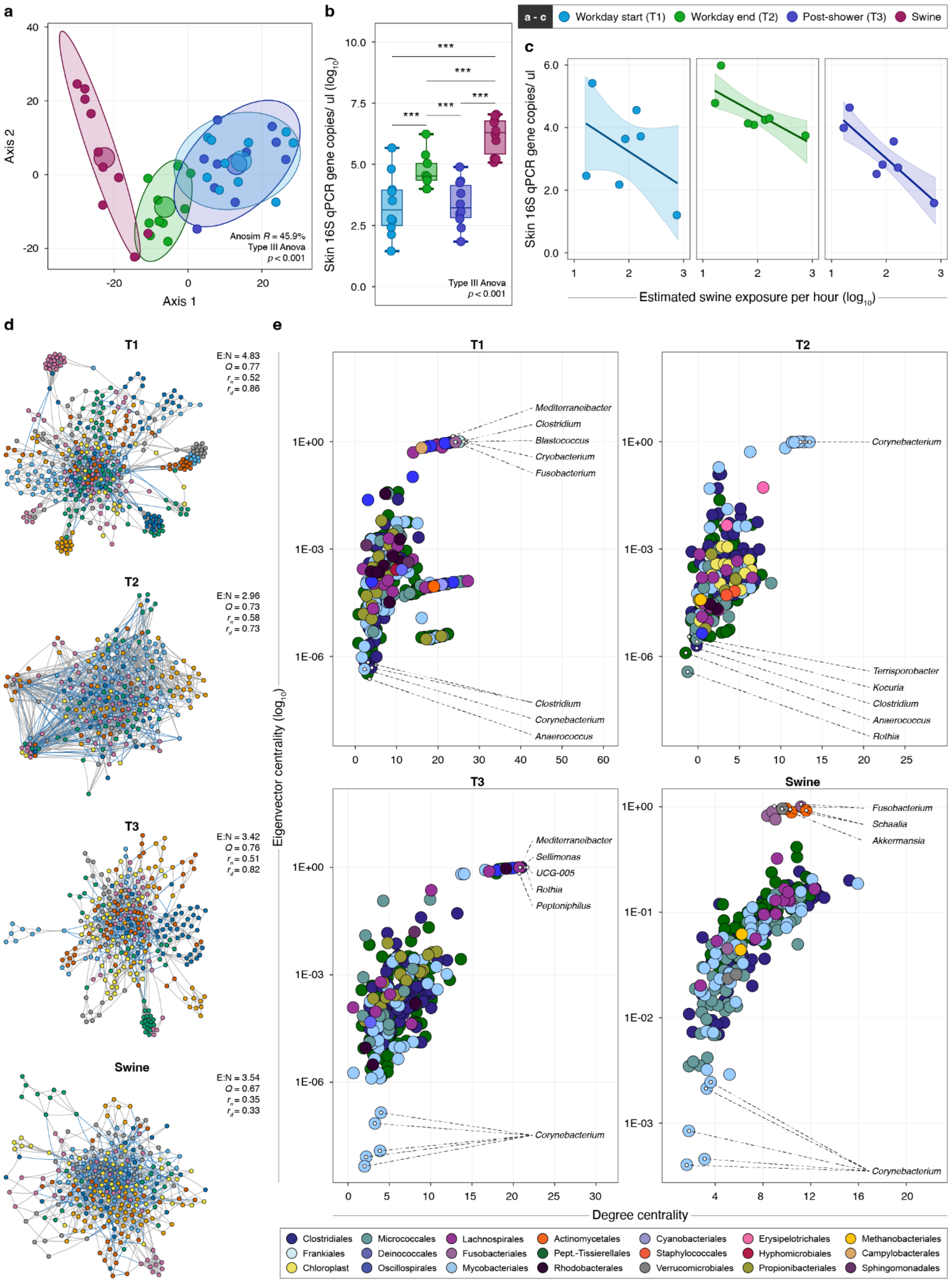

a
Axis 2
Axis 1
Anosim R = 45.9%
Type III Anova
p < 0.001
b
Skin 16S qPCR gene copies/ ul (log10)
***
Type III Anova
p < 0.001
a - c
Workday start (T1)
Workday end (T2)
Post-shower (T3)
Swine
c
Skin 16S qPCR gene copies/ ul
Estimated swine exposure per hour (log10)
d
T1
E:N = 4.83
Q = 0.77
rn = 0.52
rd = 0.86
T2
E:N = 2.96
Q = 0.73
rn = 0.58
rd = 0.73
T3
E:N = 3.42
Q = 0.76
rn = 0.51
rd = 0.82
Swine
E:N = 3.54
Q = 0.67
rn = 0.35
rd = 0.33
e
Eigenvector centrality (log10)
Degree centrality
T1
Mediterraneibacter
Clostridium
Blastococcus
Cryobacterium
Fusobacterium
Clostridium
Corynebacterium
Anaerococcus
T2
Corynebacterium
Terrisporobacter
Kocuria
Clostridium
Anaerococcus
Rothia
T3
Mediterraneibacter
Sellimonas
UCG-005
Rothia
Peptoniphilus
Corynebacterium
Swine
Fusobacterium
Schaalia
Akkermansia
Corynebacterium
Clostridiales
Micrococcales
Lachnospirales
Actinomycetales
Cyanobacteriales
Erysipelotrichales
Methanobacteriales
Frankiales
Deinococcales
Fusobacteriales
Pept.-Tissierellales
Staphylococcales
Hyphomicrobiales
Campylobacterales
Chloroplast
Oscillospirales
Mycobacteriales
Rhodobacterales
Verrucomicrobiales
Propionibacteriales
Sphingomonadales

Figure 2. Changes in skin microbial load, microbiome composition, and community structure. **a.** Genus-level worker skin microbiome ***β***-diversity across collection phases (T1–T3) and contact-matched swine skin samples using principal component ordination of robust Aitchison compositions. Within-group centroids and 95% confidence intervals are depicted with a large circle and shaded ellipsoids, respectively. **b.** Log_{10}-normalized 16S rRNA qPCR copy number / µl (y-axis), stratified by collection phase (x-axis). *** indicates statistical significance (p<0.001) of pairwise comparisons based on a linear regression model with Tukey's adjustment for multiple comparisons. **c.** General linear analysis of the log_{10}-normalized 16S rRNA qPCR copy number / µl (y-axis) and log_{10}-normalized hourly exposure to swine (x-axis) based on workers' estimates from daily task assignments indicates a negative correlation (p= 0.01) across all collection phases. Shaded areas represent the 95% confidence interval around the linear trendline. **d.** Worker skin microbiome networks across workday collection phases and for contact-matched swine were inferred from inverse covariance estimation for compositions based on centered-log ratios of subsetted ASV counts containing >100 counts and >10% prevalence per ASV per sample. Inferred networks consist of nodes representing ASVs colored by shared subcommunity membership. Edges between nodes represent a significant predicted positive (blue) or negative (gray) interaction. Reported topology characteristics include network connectivity based on the edge to node ratio (*E:N*), modularity (*Q*), subcommunity assortativity (r_n), and degree assortativity (r_d). **e**. Scatter plots of microbial constituents from the corresponding networks are displayed based on the log_{10}-normalized node eigenvector centrality (y-axis) and node degree centrality (x-axis). Taxa with the highest centrality measures (top right of the distribution) are considered to be critical connectors and major hubs in community networks, and thus putative keystone taxa. ASV-level nodes are colored based on their taxonomic classification at the Class level. Genus-level labels are displayed only for genera most likely to be keystone, i.e., >95th percentile of the plot distribution (top right) and least likely to be keystone, i.e.<5th percentile of the plot distribution (bottom left).

The worker skin resistome and mobilome shifted significantly during the workday, and differed from that of contact-matched swine

Target enrichment was used to selectively capture and amplify all potential known ARGs and MGEs within the metagenomic DNA of all samples [35]. As with the microbiome, the resistome shifted significantly between each collection phase (ANOSIM p< 0.0001; R=37.5%, **Supplementary figure 6a**), but there were no statistically significant differences in ARG group richness or Shannon's diversity across the three collection phases (**Supplementary figure 7**). However, when normalized to the bacterial load as measured by the 16S rRNA gene copy number, the total ARG burden was significantly altered over the course of the work day. While bacterial load increased at the end of the workshift (T2) vs the start (**Figure 2b**), we highlight in **Supplementary figure 8a** that workers at T1 and T3 carried a significantly greater total ARG abundance than at T2, though it was also observed that showering led to a significant reduction of total skin ARGs relative to T1 (Type III ANOVA FDR-adjusted p< 0.001 with Tukey's *post hoc* analysis). The T1 worker skin also harbored a greater ARG burden than the skin of pigs (Type III ANOVA FDR-adjusted p=0.005) and the total ARG burden in the environment did not significantly differ from that of any worker or swine skin sample. Similarly, plasmids, ICE, IS, and TE underwent significant shifts in ***β***-diversity. Collection phase accounted for >40% of the variation in composition of ICE and TE, >20% of variation in composition of plasmids and IS, and ~9% of variation in composition of viruses and prophages (Plasmids, ICE, TE, and IS ANOSIM p<0.001; viruses and prophage ANOSIM p=0.02 **Supplementary figure 6b**). Plasmids, ICE, IS and TE compositions were significantly different between T1 and T2, and between T2 and T3 (all PERMANOVA p<0.001), while virus and prophage composition differed between the collection phases T1 and T2 (PERMANOVA p<0.05). The observed MGE compositional shifts at T2 coincided with a greater relative abundance of ICE genes and a reduced relative abundance of plasmids, including plasmidic mechanisms of replication, transcription, translation, and regulation (**Supplementary figure 6c-d**). Between T1 and T3, there were significant differences in composition of IS and TE genes (PERMANOVA p=0.048 and 0.037, respectively), but not plasmids and ICE. The worker resistome and MGE composition

were significantly different from swine at all three collection phases, with the exception of viral and prophages at T2 (**Supplementary figure 6b**). Regarding the mobilome, the total MGE burden on the skin normalized to bacterial load (**Supplementary figure 8b**), was significantly higher at T1 and T3 compared to T2 (Type III ANOVA, FDR-adjusted $p< 0.0001$, Tukey's *post hoc* analysis). While the total MGE abundance on swine skin did not differ significantly from worker skin at T1 and T2, worker skin at T3 (after showering) harbored a greater number of MGE alleles than swine skin (Type III ANOVA, FDR-adjusted $p = 0.0268$, Tukey's *post hoc* analysis). In contrast to the resistome samples, environmental samples contained the highest abundance of MGEs observed in the study (Type III ANOVA, FDR-adjusted $p < 0.001$, Tukey's *post hoc* analysis).

The clinically important fraction of the worker skin resistome varied throughout the workday and remained distinct from swine

We subsetted the MEGARes v2.0 database for 29 specific ARG groups previously identified as 'clinically important' (i.e., priority ARGs) [36,37]. In 41/42 enriched metagenomic samples, we detected 19 distinct priority ARG groups at gene coverage fraction >99.9%; one T2 sample did not contain any priority ARGs. These ARGs represented a low proportion of the total resistome across all worker (median[IQR]= 7.46%[8.23]) and swine samples (median[IQR]= 5.57%[4.27]), and their overall median relative abundance did not differ between workday collection phases and swine samples (Type III ANOVA $p> 0.05$).

Tetracycline (*TetM*), sulfonamide (*Sul1*), multi-drug resistance to classes of antibiotics including lincosamides, streptogramins, and pleuromutilins (*Vga*), and methicillin (*mecA*) genes were the most prevalent and abundant of the priority ARGs (**Figure 3)**, and strongly influenced hierarchical clustering of samples into four major groups (i.e. subclades). Subclade 1 was characterized by high *TetM* relative abundance and contained eight of the 10 swine samples and one or two worker samples from each of T1-T3. Subclade 2 contained five of the 9 T2 samples, one swine sample, one T1 and two T3 samples, and was characterized by a higher relative abundance of *Sul1*. Subclades 3 and 4 contained the majority of the T1 and T3 samples (i.e., 14/20), with subclade 3 defined by a higher abundance of *Vga* and subclade 4 containing the highest relative abundance of *mecA*.

The *mecA* gene, a methicillin resistance allele, consistently appeared in worker but rarely in swine samples (**Figure 3**). *Staphylococcus aureus* in swine has been proposed as a key source of methicillin resistant *S. aureus* (MRSA) in Danish swine workers [38], especially among workers of Danish pig herds in which historical MRSA prevalence exceeds 85%. However, recent reports suggest that *Staphylococcus* spp. are actually rare members of the porcine skin microbiome, and typically account for <1% of the overall relative abundance of all Staphylococci [33]. We performed marker-based strain-level taxonomic profiling of metagenomic reads via StrainPhlAn to ascertain possible Staphylococcal sources of *mecA*. Strains of *S. epiderimidis*, *S. haemolyticus*, *S. hominis,* and *S. equorum* were the only prevalent strains identified (>75% prevalence), and no *S. aureus* strains were identified at this pre-specified prevalence level (**Supplementary datafile 9**, **Supplementary figure 9**). Further phylogenetic analysis suggested that these Staphylococci were rarely shared between workers and swine, as most strains were tightly clustered by worker

ID rather than collection phase (**Supplementary figure 10**). Major coagulase negative Staphylococci (CoNS) are known carriers of *mecA*, and ~90% of U.S. *Staphylococcus epidermidis* clinical isolates in particular are methicillin resistant [39–41]. Taken together, these findings suggest that worker CoNS and not *S. aureus* were likely sources of *mecA* in this study.

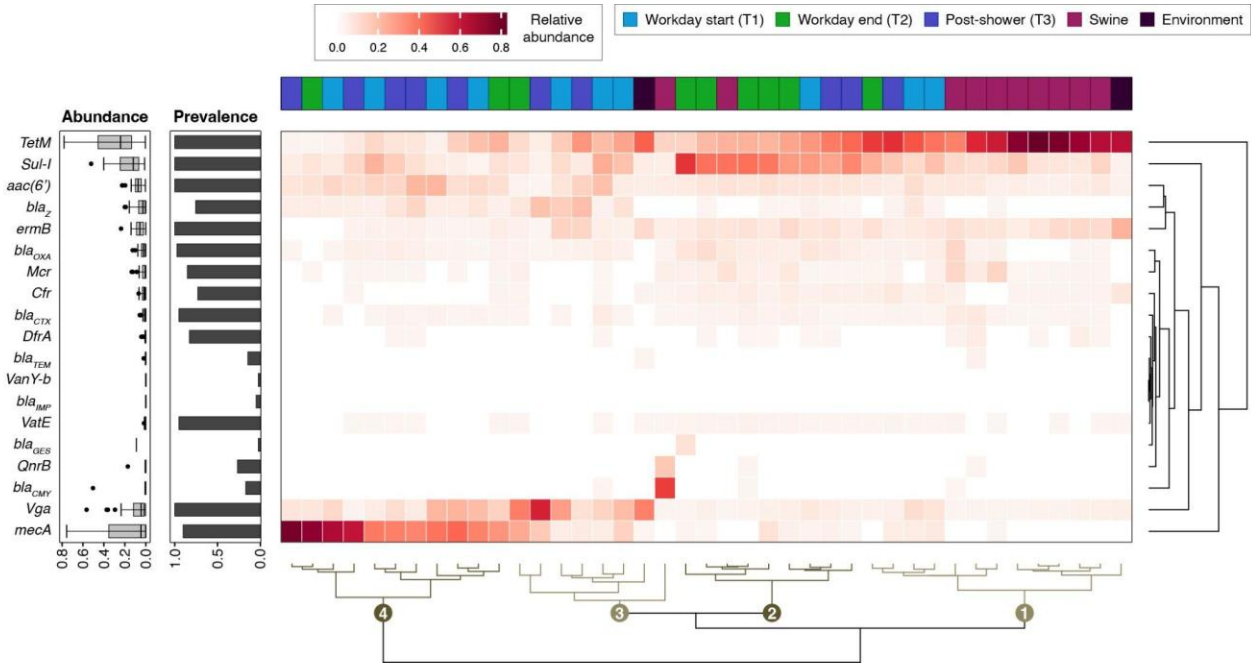

Figure 3. Occurrence of medically important ARGs on the skin of workers and swine. Unique medically important (i.e., priority) ARG alleles were identified at >99.9% alignment gene coverage across all collection phases (top ribbon annotation) and are displayed using a heatmap summarizing their sample-level relative abundance across each of the respective 19 ARG gene groups. The cladograms along the x-axis demonstrate the hierarchical clustering of samples according to their medically important resistome composition using optimal leaf sorting and euclidean distances. Four major subclades are colored and numbered. Major ARG group prevalence and median abundance across study samples are summarized via the associated barplots and boxplots along the y-axis.

Post-work showering incompletely reverses changes in resistome and mobilome gene abundance

After controlling for worker age, gender, BMI, smoking status, frequency of pork consumption, as well as host-removed sequencing depth, only 5.4%, 3.7%, and 2.7% of ARG groups exhibited significant changes in relative abundance at T1 vs. T2, T2 vs. T3, and T1 vs. T3, respectively. Between T1 and T2 collection phases, the vast majority of significantly changing ARG groups exhibited increases in abundance (i.e., 27/29 ARG groups, 93%) (**Figure 4a, Supplementary datafile 10**). Because log-fold differential abundance testing can produce false positives for low-count features, we highlighted only high-abundance ARG groups with a statistically significant change in relative abundance. For the T1 versus T2 comparison, this included ARG groups within the $\boldsymbol{\beta}$-lactams (e.g., *mecA*), fosfomycins (e.g., *fosA* and *fosB*), and mupirocins (e.g., *mupA*). In contrast to the T1-T2 comparison, far fewer ARG groups experienced statistically significant changes in relative abundance from T2 to T3 (n=18), and most of these (i.e., n=13, or 72%) decreased in relative abundance, including $\boldsymbol{\beta}$-lactams (e.g., bla_{GES}), fusidic acids (e.g., *fusB*), phenicols (e.g., *cmlA*), sulfonamides (e.g., *sulIV*), MLS (e.g., *ereA*), and multi-drug or

multi-compound classes (e.g., *fexA, ttgB, mexW, lmrD*). Two abundant ARG groups that exhibited significant increases in relative abundance at T2 compared to T1 also remained elevated after showering in T3, most notably *mecA*, and *norA*, the general drug and biocide efflux system of Staphylococci [42]. When compared to T1, 13 ARG groups at T3 were significantly differentially abundant, and 10 of these (77%) exhibited a significant decrease in relative abundance (**Figure 4a**). For example, among the most abundant T3 ARGs, there was a significant decrease in multi-compound and fusidic acid resistance (e.g., *fexA*, *mepA*, *fusB*), and multi-drug resistance regulators and efflux systems (**Supplementary datafile 10**).

Among MGEs with significant changes in relative abundance between collection phases, ICE were most prominent (**Figure 4b**). Specifically, *ICEPaeLESB58-1*, *ICETn4371* and *ICESsu(BM407)* were more abundant in T2 versus T1 samples; the first two mobilize heavy metal resistance [43], while the latter mobilizes ARGs narrowly within *Streptococcus suis*, an emergent pathogen in humans that is considered a host-adapted swine pathobiont [43,44]. Further strain analysis using gene markers confirmed presence of *S. suis* in all swine and 9/10 of worker samples in each collection phase with a mean relative abundance of 10.2% at T1, 26.1% at T2, and 12.8% at T3 (**Supplementary datafile 9**). However, this marker-based approach is unable to further distinguish zoonotic from non-zoonotic serotypes. Compared to T1, T2 samples also contained significantly higher relative abundance of replication and recombination machinery of the host-adapted *Staphylococcus epidermidis* bacteriophage (e.g., helicase loader and replication helicases and Holliday junction resolvases), as well as *IS6* sequences associated with methicillin resistance (*IS431mec*), erythromycin resistance (*IS257-1*), and transposable components of *IS6*/*IS26* and *TnAS3* involved in mobilizable resistance [44] at human-animal interface contexts.

Following showering, the most abundant ICE module *ICETn6087* was reduced in relative abundance compared to T2; however, the next 9 most-prevalent MGEs increased in relative abundance at T3 versus T2 (**Figure 4b**), notably *IS431mec* which already exhibited a significant increase from T1 to T2. Other significantly more abundant MGEs in T3 versus T2 samples included plasmids of *Staphylococcus epidermidis* (*pSepCH*, *SE_p410*), *Staphylococcus aureus* (repV: *pT181*; pgi: *pSJH901*); as well as *Staphylococcus epidermidis* and *Bacillus cereus* bacteriophages and prophages. *S. suis*-adapted *ICEPaeLESB58-1* and *ICETn4371* were significantly more abundant at T3 versus T1; while promiscuous tetracycline-associated Tn916-like ICE *ICETn6085a*, *ICETn6085b*, and *ICETn6084* were significantly less abundant in T3 compared to T1 [45] (**Supplementary datafile 11**).

Worker skin at T2 (i.e., following work with swine) had a higher MGE abundance than contact-matched swine (**Figure 4b**), dominated by mucous membrane, respiratory tract, and enterically adapted ICE. Prominent among these were Streptococcal RD2 element (10750-RD.2), *ICETn1806*, as well as *ICESauJKD6008* and *ICECTn4* known to mobilize vancomycin and tetracycline resistance in *Staphylococcus aureus*, Enterococci, and *Clostridioides difficile* [46–49]. *Staphylococcus aureus* and *Escherichia coli* plasmid replicon modules were also in higher abundance in T2 worker versus swine skin (e.g., *repUS12_pUB110*, *repUS23._repA(SAP099B)_GQ900449.1*, and *IncY_1__K02380*). Additionally, worker skin contained a higher relative abundance of *Psychrobacter*-associated plasmid *pRWF101_PsycPRwf*. *Psychrobacter* species were identified as the most abundant member of

the swine skin microbiota in strain analysis (**Figure 2e**), especially *P. pasteurii* and *P. piechaudii*, whereas strains of *P. faecalis* and *P. maritums* were detected in nearly all human, swine, and environmental samples. The detection of *psychrobacter*-associated MGE alleles in human samples was, however, unexpected. Historically, *Psychrobacter* isolates have been obtained from arctic, marine, sediment, and limited terrestrial environments [50]. However, recently this genus has been detected in pig slurries, manure, and swine carcass processing facilities [51,52] and *Psychrobacter* spp. have been identified as dominant microbes within the nares of workers involved in swine transport [53]. We demonstrate that skin-borne *Psychrobacter* and its mobilomic components may be suitable indicators of microbial exposure to the swine environment.

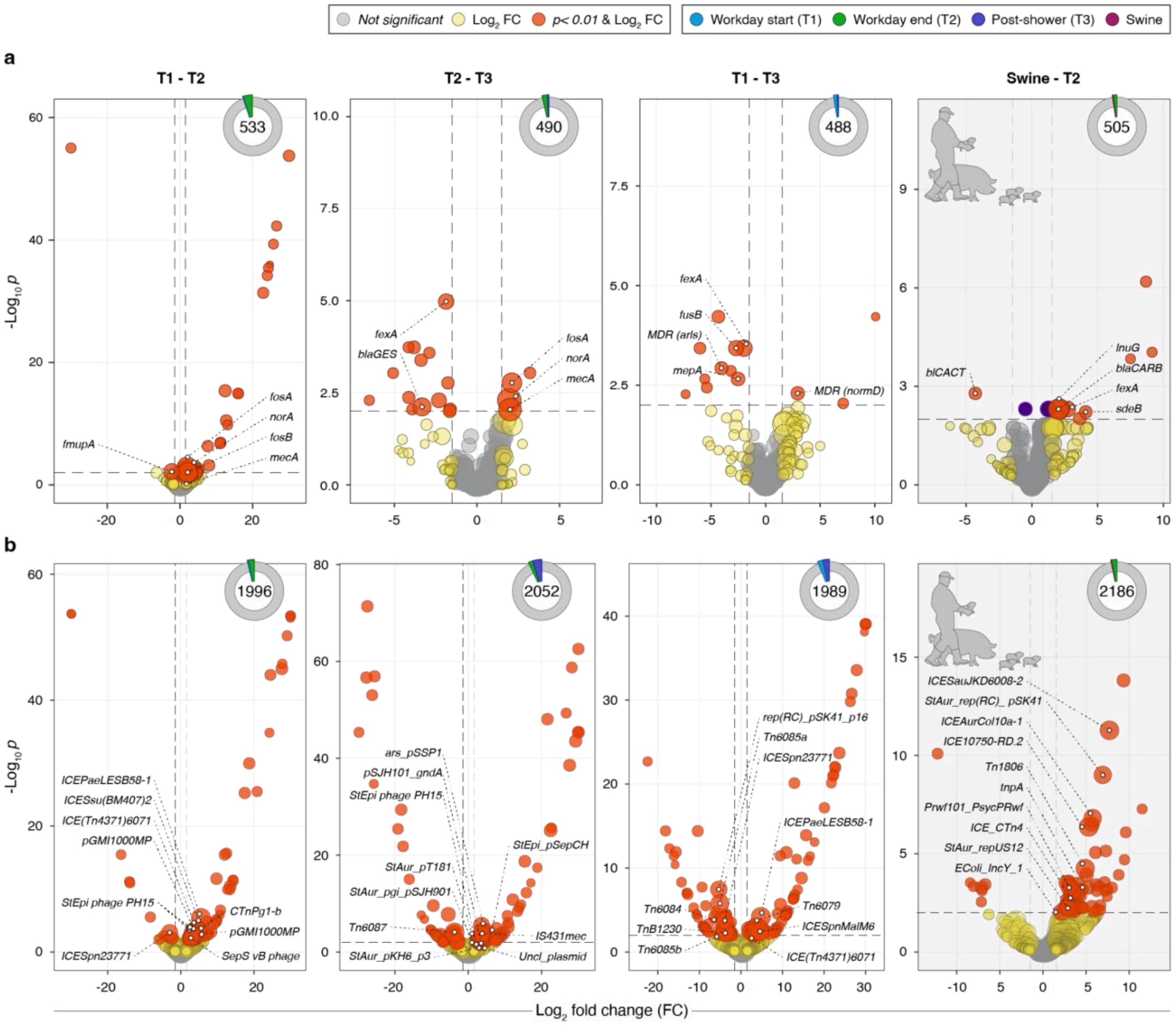

Figure 4. Differential abundance analysis across collection phases and at the interface period between workers and pigs. Volcano plots are used to visualize differential abundance of unique **a** ARG groups and **b** MGE accessions in log_2-fold change (x-axis) and -$log_{10}P$ value (y-axis) of the global worker skin resistome or mobilome between key workshift collection phases: Workday start (**T1**) vs. workday end (**T2**); Workday end (**T2**) vs. post-shower (**T3**); and Workday start (**T1**) vs. post-shower (**T3**). An additional comparison is made between workday end and swine skin samples representing the worker's contact phase with animals (gray). Features with significant shift in abundance (Wald's $p<0.01$ with FDR adjustment for ARG group and MGE accessions) are displayed above the horizontal line, while biologically significant fold-change is demarcated by vertical dashed lines at 1.5 log_2-fold change. Labels are displayed for only the 5 most abundant ARG groups and 10 most abundant MGEs significantly amplified (log_2-fold change >1.5 or < -1.5) at each phase comparison. For each volcano plot, an associated pie chart displays the number of unique ARG groups and MGE accessions common to each of the workshift collection phases compared, as well as the proportion of the total differentially abundant MGEs associated with each phase.

MAGs recovered from worker and swine skin samples represent putatively novel strains

High quality metagenome-assembled genomes (MAGs) were constructed via *de novo* genome assembly for all individual samples and also as co-assemblies of samples within T1, T2, T3, swine and environmental (**Figure 5a–c, Supplementary datafile 12**). The *Bacillota* phylum was by far predominant across all genomes (n= 139), followed by *Actinobacteriota* (n=44), *Bacteroidota* (n=17), and *Pseudomonadota* (syn. *Proteobacteria)* (n=9). The distribution of the most abundant phyla across all MAGs was consistent with the phyla detected by 16S microbiome sequencing. A large proportion of identified MAGs had poor taxonomic representation among known Genome Taxonomy Database (GTDB) MAGs, as 47 (22%) were classified as putatively novel species (i.e., <95% ANI with a known sequenced genome in GTDB), and 167 (78%) were identified as putatively novel strains (i.e., <99% ANI with a known sequenced genome in GTDB).

Approximately 60% (28/47) of all MAGs considered to be putatively novel species were recovered from swine skin samples, even though swine samples represented <25% of analyzed samples (i.e., 10/42). Swine samples also accounted for ~53% (89/167) of the MAGs identified as putatively novel strains. We detected new strains that were highly abundant in recent swine intestinal MAG catalogs [54] and that we also identified either via 16S or strain gene-marker analysis in this study, including *Psychrobacter* (*P. pasteurii*), *Streptococcus* (*S. hyovaginalis, S. pluranimalium, S. dysgalactiae*), *Corynebacterium* (*C. xerosis, C. variabile, C. glutamicum, C. pollutisoli, C. stationis*), and *Lachnospiraceae.*

Worker skin samples accounted for ~45% of the de-replicated MAGs, retrieved predominantly from co-assembly (n=62) vs. individual (n=33) approaches. Nearly 75% (71/95) of MAGs recovered from human samples represented either novel species or strains. These novel taxa comprise 9 genera, 6 of which are known to be natural inhabitants of environmental matrices, including *Microbacterium*, *Marihabitans*, *Marmoricola*, *Chloroflexi* bacterium, *Qipengyuania*, and *Tsuneonella*. Samples representing the swine farm exposure phases (i.e., T2 and T3) accounted for 76% of the total putatively novel strains detected in worker microbiomes (T2: 28/95; T3: 26/95). Though samples from workday start (T1) accounted for the smallest proportion of all recovered human MAGs (23%), we nevertheless captured major expected cutaneous taxa as documented in previous strain-resolved MAG workflows [55,56], including *Staphylococci* (e.g. *S. hominis, S. epidermidis, S. capitis*), *Corynebacterium* (e.g. *C. xerosis, C. mucifaciens, C. kefirresidentii*), *Cutibacterium* (e.g. *C. granulosum* and *C. acnes*), and

Lactobacillaceae (e.g. *Lactobacillus amylovorus, Latilactobacillus sakei*, *Limosilactobacillus reuteri*).

In addition to the genera observed at T1, MAGs from T2 samples also included 6 genera of the Clostridial co-abundance gene group 138 (i.e., CAG-138) previously linked with critical functions for fiber degradation in the swine enteric system [57]. Genera not assigned with NCBI taxonomic nomenclature from Lachnospiraceae, Butyricicoccaceae, Oscillospiraceae, and Treponemataceae were also recovered, and their identities were concordant with best-matched NCBI genomes sequenced from fecal samples of piglets <30 days old. Among MAGs recovered from T3, ~55% (20/36) included species identical to those observed in both T1 and T2 samples. However, T3 MAGs also included species that were only observed at T2 (and not at T1), including taxa typically identified in livestock such as *Streptococcus alactolyticus* known as part of the *Streptococcus bovis*/*Streptococcus equinus* complex (SBSEC), *Aerococcus urinaeequi*, as well as uncharacterized MAGs previously identified in swine fecal samples (GenBank ID: *GCA_016293975.1*, *GCA_004558825.1*, *GCA_004556755.1*) [58]. Though minor human skin commensals were exclusively detected in T3 samples, such as *Lawsonella clevelandensis* and *Corynebacterium aurimucosum*, genera previously isolated from environmental matrices were also exclusively recovered in T3 samples, including *Tsuneonella* sp., *Qipengyuania* sp., *Marmoricola* sp., and *Chloroflexi* bacterium UBA6265.

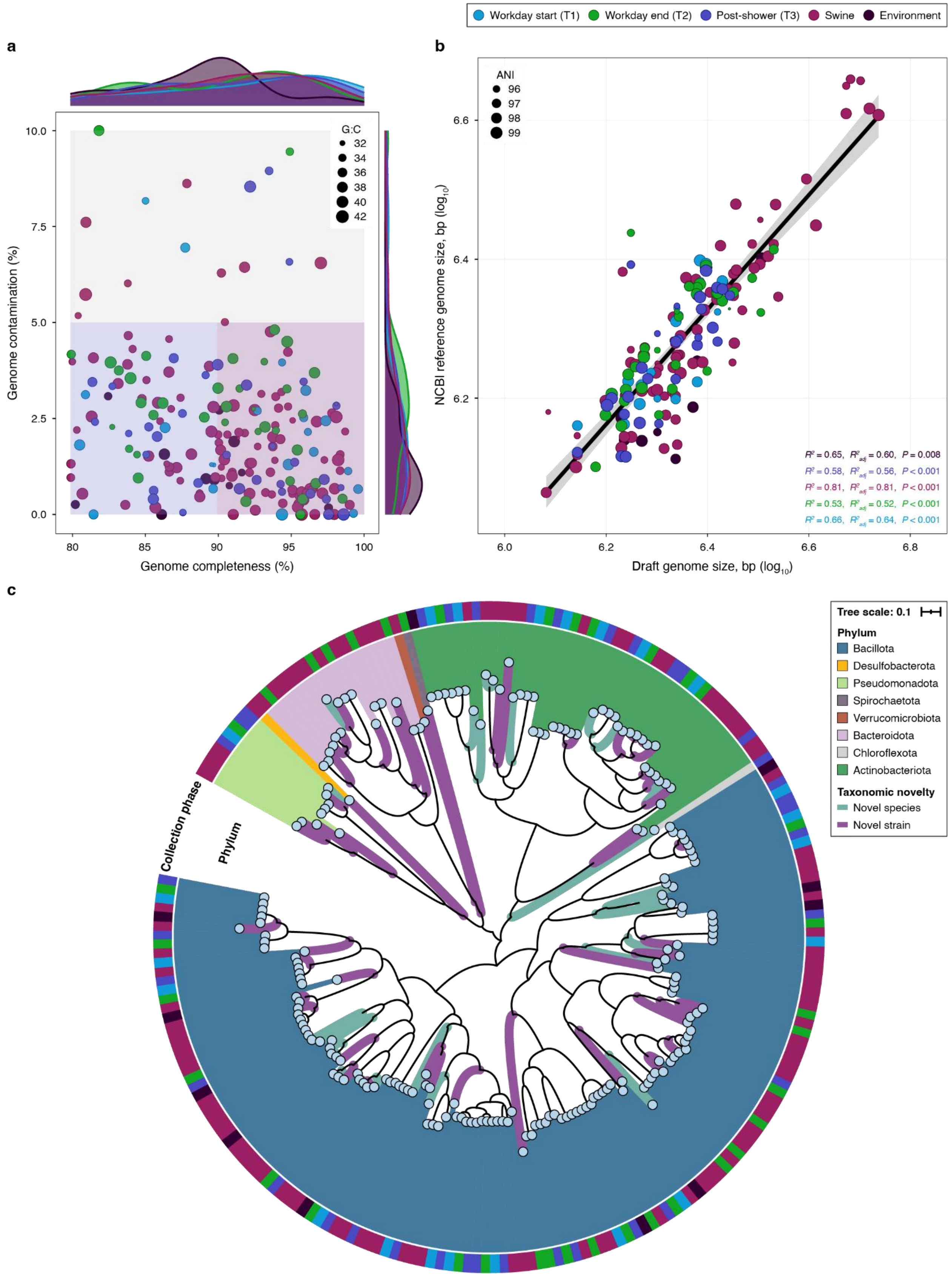

Workday start (T1)
Workday end (T2)
Post-shower (T3)
Swine
Environment
a
G:C
32
34
36
38
40
42
Genome contamination (%)
Genome completeness (%)
b
ANI
96
97
98
99
NCBI reference genome size, bp (log10)
Draft genome size, bp (log10)
R^2 = 0.65, R^2_{adj} = 0.60, P = 0.008
R^2 = 0.58, R^2_{adj} = 0.56, P < 0.001
R^2 = 0.81, R^2_{adj} = 0.81, P < 0.001
R^2 = 0.53, R^2_{adj} = 0.52, P < 0.001
R^2 = 0.66, R^2_{adj} = 0.64, P < 0.001
c
Collection phase
Phylum
Tree scale: 0.1
Phylum
Bacillota
Desulfobacterota
Pseudomonadota
Spirochaetota
Verrucomicrobiota
Bacteroidota
Chloroflexota
Actinobacteriota
Taxonomic novelty
Novel species
Novel strain

Figure 5. Taxonomic diversity and novelty of resolved metagenomic assembled genomes (MAGs) recovered from the human-swine interface and ambient environment. a Scatter plot of points representing individual GTDB taxonomically binned MAGs according to their estimated percent completeness (x-axis) relative to their estimated percent contamination (y-axis). Point size is proportional to the quantified MAG GC-content. Pink, purple, and gray regions of the plot demarcate the density of 'high quality', 'medium quality-low contamination', and 'medium quality' MAGs retrieved across all collection phases. **b** Scatter plot of estimated MAG size is displayed based on the $\log_{10}$-normalized nucleotide count (x-axis) and the nearest assigned NCBI reference genome size expressed in $\log_{10}$-normalized nucleotide count (y-axis). A global regression line and 95% confidence interval (shaded region) is displayed and results of significance testing (R^2; $p<0.05$) using a generalized linear model are colored for MAGs recovered for each collection phase. **c** Phylogenomic tree of de-replicated and high-confidence MAGs recovered from target-enriched metagenomes across each collection phase (outer ring). The area below each leaf is colored according to the taxonomically assigned Phylum. Branches for proposed novel species (ANI <95%) are displayed in teal, and for proposed novel strains (ANI <99%) are displayed in purple.

DISCUSSION

Environmental exposure histories play a determinative role in shaping adult microbiomes, even more so than individual-level variables [59–61]. Cutaneous microbiota are recognized for their remarkable stability in the face of environmental perturbation over short time scales [62–64]. Despite this, we demonstrate that swine worker skin experiences a significant increase in bacterial load and a significant shift in microbiota composition during a single 8-hour workday. However, showering at the end of work seems to dampen these changes, indicating that biosecurity interventions not only reduce worker-borne swine pathogen transmission [65,66], but also work-associated microbiome impacts. We likewise demonstrate that accumulation of ARGs on the skin can be counteracted with showering, suggesting that such biosecurity practices could be important public health measures to reduce the bidirectional flow of resistant bacteria between animal-associated workplaces and the general community [67–69]. Our work highlights that showering may have a variable impact on other aspects of the microbial metagenome, such as mobile genetic elements which were in some cases enriched even after showering at the end of the workday. Effects of farm protocols and biosecurity have not been expressly evaluated in recent investigations of 'shareable' microbial features between humans, animals, and the farm environment [5,53,70–73]. Our results suggest that a more nuanced understanding of these practices on different components of the metagenome is warranted, particularly given the different intra-shift dynamics we observed across the resistome, mobilome and microbiome. Future studies should include detailed characterization or even measurement of process controls, including the use of biosecurity practices for decontamination and exposure reduction via personal protective equipment (PPE), occupational training and monitoring, and environmental management.

Within-farm sources of microbes that shape worker microbiomes remain unknown, and there are no systematic, established methods for conducting microbiome-based surveillance in occupational health contexts, and particularly for commercial farm work. The striking proportion of possibly novel species that we recovered from the skin of both swine and workers suggests that skin may be an important yet under-represented sampling target for on-farm occupational health research (**Figure 5**). We focused our study on the skin for several reasons, including ease of sampling and a high proportion of skin-associated diseases within livestock workers; additionally, the skin surface is continuously exposed to the farm environment and thus likely to serve as a competent catchment for air-borne bacteria. Bacteria from livestock feces, soil, and water are relevant sources of exposure that may induce shifts in human microbiomes. For example, swine farms and especially swine feces can impact the antibiotic resistome of

individuals living in proximity to the farm [74]. However, emerging evidence suggests that air and dust should not be overlooked as important sources of microbiome and resistome richness [75–77]. Farm dust has also been shown to be protective against asthma [77], and together, these studies underpin the need to further investigate the skin-environment interface. Though our study was not expressly designed for robust source-attribution of the microbiome, ARGs, and MGEs in worker skin, future work could conduct more precise analyses of the bacteria and their genetic features. Such detail could inform methods to control or reduce bi-directional exchange of bacteria along the human-animal-environment continuum. To achieve this, more thorough sampling of diverse environmental matrices would be needed, and microbiomes of workers should be studied with more precise measures of exposures to air, soil, feed, dust, feces, and animals when performing a range of tasks over the course of the workday.

One major question is whether work-acquired microbes become incorporated into the cutaneous microbiome as long-term, stable members of the community. Our study design did not include long-term follow-up, and thus we could not quantify the proportion of taxa that become *de facto* colonizers following repeated workday exposures. However, we did demonstrate that showering seemed to literally wash away many of the microbes, ARGs, and MGEs that accumulated on the skin during the workday. This may be due to the fact that bacteria acquired during the workday become only weakly adherent to the skin and thus are easily washed away. The impact of showering is even more robust when one considers the heterogeneity in showering practices, as workers in this study were told to shower as they normally would at the end of their workday, including use of their own preferred soaps and other personal care products. However, it is important to note that showering did not completely eliminate newly-acquired taxa, and workers may continue to harbor microbes from enteric and environmental taxa that are characteristic of the swine farm context, as shown in our 16S rRNA and MAG results (**Figures 2** and **5**), and reported in a previous study of swine farm workers who resided in a Chinese swine farm for ~3 months [5]. In this study, we focused on showering as a key biosecurity intervention to reduce the theoretical carryover of farm microbiota, ARGs, and MGEs. However, swine workers followed multiple biosecurity practices throughout their workday, which may have influenced the microbiome composition at T2 and ultimately at T3. The effects of additional protective measures—such as wearing coveralls, boots, gloves, handwashing, and sanitizer use—could not be isolated from the impacts of swine farm exposure at T2, as these practices were either mandatory or encouraged for all workers as part of occupational safety programs at the commercial swine farm. However, the detection of significant microbiota shifts at T2, despite the variability in how swine workers traditionally adhere to personal protective equipment (PPE) protocols and hygiene [78,79], suggests that farm factors beyond PPE and other biosafety measures contribute to dynamic changes in worker skin microbiota at T2 and T3.

Our findings underscore several critical considerations for future research on workplace and environmental influences on human microbiota. Most importantly, this area of occupational research requires refined methodologies for sampling, microbiome quantification and molecular epidemiologic analysis. These methodologies must be developed for the diverse environmental conditions, varying microbial burdens, and the complex interactions at human-animal-environment interfaces. In this study, we employed a multi-site composite sampling strategy to examine the skin microbiome and metagenome across different phases of collection. This approach was carefully designed to address key challenges in occupational skin microbiome

research: (a) We sampled multiple skin sites representing distinct microenvironments (e.g., dry, moist, oily) that support different bacterial taxa; (b) We selected sampling sites based on a range of exposure likelihoods to air, dust, feces and other components of the swine environment, considering sampling areas that were both protected and unprotected by PPE; (c) By pooling samples into a composite, we streamlined the collection process, making it less burdensome and more easily integrated into the daily routines of agricultural workers. Second, occupational microbiome research is challenged to establish causal links, i.e., do workplace exposures influence the human microbiome in the long-term, and do these influences lead to different health outcomes? This study is a prime example of this challenge, as we enrolled farm workers who already had months or years of on-farm exposures. Thus, the "baseline" T1 skin microbiome may have already been impacted by previous on-farm exposures, but we have no robust method for detecting these prior impacts. Similarly, it is tempting to compare our data to publicly available human skin microbiome data, but such a comparison would be inextricably biased by confounders such as the well-documented 'healthy worker' effect and other demographic variables known to impact human microbiomes. The need for robust epidemiological study design is even more pronounced in cohort-based microbiome studies because of the lability of the human microbiome. We therefore expressly avoided comparisons across worker and non-worker cohorts and took care not to extrapolate our findings into long-term microbiome or health impacts. Instead, we focused our analysis on a time-series sampling design of the same workers before and after exposure and showering, allowing for a targeted analysis of daily farmwork and biosecurity interventions. This intra-individual focus allowed us to circumvent many of the biases that beset ecological analyses, but at the expense of external validity and ability to draw conclusions about the long-term impact of farm work as compared to non-farm work.

Finally, we used a combination of sequencing and bioinformatic approaches that were tailored to maximize sensitivity for resistome and mobilome analysis while also supporting a broader investigation of the microbiome. Specifically, we used target-enriched shotgun sequencing, an adaptation of traditional shotgun metagenomics, to enhance detection of ARGs and MGEs which are typically undetected by metagenomic workflows [35,80,81]. Such false negative findings are most pronounced in low biomass samples, such as skin samples, which are replete with non-microbial gDNA [64,82]. Thus, our choice of target-enriched metagenomics was driven by its capacity to reduce false negatives in resistome and mobilome profiling. The enrichment procedure hinges on biotinylated hybridization probes, which tolerate >40% sequence mismatch between the sequence target and the probe (i.e. up to 48 base mismatches across a 120-*mer* oligo probe). This inherent hybridizing flexibility facilitates the capture of both pre-defined targets and other potential closely related variants. Despite this enrichment, resistome and mobilome genes constituted <16% of the host-filtered metagenomic reads in this study, meaning that >84% of sequencing data across samples did not remain from ARGs or MGEs (i.e. was 'off-target'). We used this remaining off-target data to reveal strain-level dynamics from the recovery of specific strain markers, and the analysis of high-quality genomes recovered from human, animal, and environmental metagenomes. However, because the process of target-enrichment purposefully induces a non-uniform impact on metagenomic content, its effects on compositional analyses remain poorly characterized at this time. Given these potential biases, we complemented our analysis with 16S rRNA amplicon sequencing to provide a less biased assessment of microbial community structure across the skin of swine workers, swine, and environmental samples. This dual sequencing strategy ensured both

high-resolution resistome-mobilome characterization and robust taxonomic profiling of the skin microbiome.

It is difficult to extrapolate our single-day, single-farm study to longer-term dynamics of the worker skin microbiome, and our results suggest that further work is needed to determine whether daily environmental exposures impact the long-term profile of the worker skin microbiome; and whether such impacts carry over into the general community via human-to-human transfer. We note, however, that this study is the first of its kind to deeply and systematically interrogate the stability of the microbiome, resistome, and mobilome of farm workers as they enter and exit a commercial U.S. swine production system. Owing to difficulties in accessing tightly controlled swine environments, such microbiome exposure assessments on U.S. farms are scarce. These results therefore offer an important foundation from which more robust microbiomic investigations can emerge.

MATERIALS AND METHODS

Study design and procedures

Ten workers from a single commercial farrow-to-wean operation in the Midwestern region of the United States were voluntarily enrolled into the study after providing written informed consent. At time of sampling, the farm housed approximately 3,500 sows and >15,000 piglets, and weaning occurred at an average age of 22.5 days of age. This large-scale swine farm had been in operation for >20 years, and was chosen because all facility personnel were day-shift workers and tended to have specialized job tasks requiring a wide array of swine contact, ranging from no direct contact (e.g., facilities maintenance, sanitation, manure management) to intensive direct contact (e.g., assisting with farrowing, piglet processing, or providing veterinary care). The human sample collection events for this study were integrated into a pre-existing, long-standing biosecurity pathogen surveillance program which already involved self-collection of skin swabs. Incorporating microbiome sampling within the existing workplace surveillance program increased study participation and self-sampling consistency, and was determined to be least disruptive to work-related schedules and task performance.

All workers were enrolled and sampled on the same day. Worker enrollment eligibility criteria included a restricted age range (18–60 years); proficiency of spoken and written English; minimum of 6 months continuous employment at the facility; no other contact with swine outside of the workplace; no exposure to antibiotics, immunosuppressants, antifungal, or antiviral drugs in the prior 3 months; no hospitalization or incarceration for > 24 hours in the 3 months prior to enrollment; free of any *known* symptoms, infections, or diseases of the upper-respiratory tract (including ears, nose, throat) and skin at the time of enrollment; no *known* current or past diagnosis of autoimmune disease; no cancer diagnosis or related therapy in the previous 5 years; and an afebrile status at the time of enrollment. Participants were asked to abstain from any additional showering or application of personal care products to their skin other than those required at the swine facility, for a period of 24 hours prior to sampling. Following the provision of study description information and free informed consent forms for review, formal consent was obtained in confidence and without the presence of farm management personnel from all participants. Participants agreed to self-collect repeated skin swabs at three points in their working day, and to complete a short questionnaire regarding personal demographic and

biometric information, smoking history, dietary and hygiene habits, and occupational and non-occupational related exposure assessments. Variables from the questionnaire were used in downstream multivariable statistical analyses. All study participants, their samples, and questionnaire data were kept deidentified to study personnel and investigators using an assigned alphanumeric coding scheme and an electronic tracking system. Monetary remuneration for study participation was provided in the form of $100 debit cards.

Sample collection

Epidermal swabs (hereafter referred to as “skin swabs”) were self-collected at three workday collection phases: prior to entry into the animal holding areas of the swine operation and before showering-in (T1: “Workday start”); at the conclusion of the 8-hour period of assigned work, but before showering-out (T2: “Workday end”); and upon exiting the facility following a mandatory showering procedure (T3: “Post-shower”). At each time point, collection kits were provided to workers for self-sampling. The kits contained two sterile BD BBL CultureSwabs EZ (Becton, Dickinson and Co., Franklin Lakes, NJ, USA) pre-moistened with a solution containing 0.9% NaCl and 0.1% Tween-20 biosurfactant (Thermo Fisher Scientific, Waltham, MA, USA). Each swab was used for self-swabbing of all locations on one half of the body (i.e., left or right), to obtain a single composite representing the following locations on each body half: manus and interdigital space, antecubital fossa, popliteal fossa, and axilla [83]. These locations were selected to represent sebaceous, moist, and dry epidermal microenvironments of the human skin [83]. Swab pattern, pressure, duration, and frequency were performed in accordance with the Human Microbiome Project [84] core microbiota sampling protocol A, including the use of a z-like pattern of consistent swabbings over specified surfaces via 50 passes over a 30 second period. For the manus, ‘z-like’ pattern swabbing was conducted across the palm and surface of the fingers for a 30 second duration as well as an additional 30 seconds of linear passing in the interdigital spaces. For all workday collection phases, the left and right composite swab tips were separated from the swab stems and placed into 1X phosphate-buffered saline (PBS) at pH 7.4, immediately transferred to dry ice for transport, and subsequently placed in -80 °C for long-term storage.

Swine skin swab samples were collected as follows: each worker was observed handling or working near specific pens containing sows, piglets, or both in a room during their dayshift. Immediately after the worker completed their contact or their required duties in the specific pen, pen-level composite skin samples were collected by passing a sterile EZ Reach Sponge sampler on the dorsal aspect of the skin of each pig in the pen, from withers to tail-base (World Bioproducts, Woodinville, WA, USA) impregnated with 10 mL of 1X PBS . In cases of farrowing pens, the sow and all piglets in a litter were sampled. In gestational pens, only the pregnant sows contacted by workers assigned to a specific area of the facility were sampled. Swine samples were assigned a worker-matched ID, transferred to sterile Whirl-Pak bags (Nasco, Fork Atkinson, WI, USA) and placed on dry ice for transport.

Two additional sterile EZ Reach Sponges were exposed to ambient air for ~20 seconds, one each in gestational and weaning environments of the swine facility, where workers spent the majority of their dayshift; these swabs were meant to capture the airborne environmental microbes within the primary working areas of the facility. After transport (~2 hours driving distance), all samples

were immediately placed in -80 °C for long-term storage at the Food Centric Corridor infectious diseases laboratory at the University of Minnesota.

Sample processing and gDNA extraction

All sample processing occurred in a class II biological safety cabinet decontaminated using UV radiation and 70% ethanol in between handling human, animal, and environmental samples. After thawing samples at -20 °C and then at room temperature, human swab tips and excess storage fluid were transferred for DNA extraction. An additional 100 μl of 1X PBS was used to recover possible residual microbial material in each sample tube. For swine and environmental samples, the ~10 mL buffer adsorbed to each polyurethane sponge paddle was expressed and reserved in 50 mL conical centrifuge tubes. An additional 10 mL of fresh 1X PBS was allowed to re-adsorb to each sponge and equilibrate for 10 minutes, and this 'rinse' was expressed into the same conical tube as the original rinsate for subsequent centrifugation (8,000 x g 15 min at 4 °C) and transfer of pellets into ~200 μl PBS for DNA extraction.

Genomic DNA (gDNA) extraction began with mechanical and chemical lysis using the Qiagen DNEasy Powersoil Pro kit (Hilden, Germany, Lot# 163044275) and PowerBead Pro tubes containing zirconium beads and 800 µl lysis buffer. Molecular-grade sterile water was placed into 3 randomly selected tubes to serve as negative controls (extraction blanks). After 6 s of vortexer-mediated homogenization, samples were placed on a 115V BioSpec Products Mini-Beadbeater-96 (Bartlesville, OK, USA) for mechanical lysis. Sample bead beating proceeded at 2400 rpm for 30 s for a total of 3 rounds with a 2-minute pause on ice between each round, to prevent overheating. The remainder of the extraction and purification procedure was performed following the PowerSoil Pro protocol with inhibitor removal steps using the QIAcube Connect automated instrument. The final 50 µl of eluted gDNA was stored at -20 °C.

Additionally, gDNA was extracted from two 200 µl aliquots of ZymoBIOMICS microbial community II standard Catalog# D6310 (Irvine, CA, USA), consisting of 8 prokaryotic and 2 eukaryotic microorganisms in known log-distributed abundance to serve as positive controls. The aliquots were individually centrifuged at 15,000 x g for 5 min, and the pellets were suspended in 400 μl of Qiagen CD1 lysis buffer, transferred to PowerBead Pro tubes, and vortexed for 10 min at maximum speed on a Vortex Genie 2 mixer (Scientific Industries, Bohemia, NY, USA) with a Qiagent adaptor (Catalog# 13000-V1-24). After isolating and reserving the initial supernatant, an additional 400 μl of CD1 lysis buffer was added to the same PowerBead Pro tubes, and these were subjected to bead beating (Mini-Beadbeater-96) at 2400 rpm for four 5-min cycles with 5-min rest intervals on ice in between each cycle. After centrifugation at 15,000 x g for 2 min, the resulting supernatant was recombined with the previously reserved supernatants for each aliquot. The gDNA from the two ~700 μl lysates were isolated and purified on the QIAcube Connect according to the same protocol as for the previously described samples.

16S rRNA amplicon library preparation and sequencing

The 16S rRNA gene copy number in each sample was measured using qPCR prior to library preparation. For 16S sequencing, the target copy number threshold was set at 167,000 molecules / µl. Amplification of libraries was performed using a dual-indexing Illumina primer set (Forward primer: *5'*— TCGTCGGCAGCGTCAGATGTGTATAAGAGACAGCCTACGGGAGGCAGCAG —*3'* and Reverse

primer: *5'*— GTCTCGTGGGCTCGGAGATGTGTATAAGAGACAGGGACTACHVGGGTWTCTAAT —*3'*) targeting the V3-V4 region [85]. Amplicons were quantified using a PicoGreen dsDNA assay kit (Life Technologies, Carlsbad, CA, USA). Sequencing was performed at the University of Minnesota Genomics Center (UMGC) using Illumina's v3 chemistry (2x300 bp paired-end reads) on the Illumina MiSeq platform (San Diego, CA, USA). All libraries were sequenced on the same sequencing run.

Target-enriched metagenomic library preparation and sequencing

All gDNA samples were subjected to a targeted capture and deep sequencing workflow to increase detection sensitivity for the resistome and mobilome features within the metagenomic DNA. This approach was chosen to enhance sequencing and thus detection of ARG and MGE targets, which are rare genomic features within microbiomes [35]. Targeted enrichment was performed using a custom-designed biotinylated cDNA probe panel for selective hybridization and capture. For probe design, a comprehensive list of pre-defined publicly available unique nucleotide sequences was compiled for 7,868 ARGs from MEGARes v2.0 [86] (including accessions for: drug resistance, metal resistance, multi-compound resistance, and biocide resistance) and for 738 MGE accessions (including full-length sequences for: (a) integrative conjugative elements (ICE) from ICEBerg v2.0 [87] and (b) plasmid replicons of *Enterobacteriaceae* and gram positive bacteria from PlasmidFinder v2.1 [88]. These ARG and MGE sequences comprised 8.55 Mb of total sequence. The CATCH pipeline [89] was used to generate the custom probe panel using the following parameters: probe stride: 120; probe length: 120; mismatches: 5; extension coverage: 100. The final probe design contained 71,309 unique 120mer oligos, which provided 100% horizontal coverage of all ARG and MGE targets, with at least 2x depth of probe coverage for every nucleotide. Probes were manufactured by Agilent (Santa Clara, CA, USA) with additional 'bait-boosting' to amplify GC-rich regions (defined as GC >65%) for fast hybridization reactions to produce a final panel of 148,162 probes covering 17.78 Mb. Probes were stored at -80 °C prior to use.

All gDNA samples (n=42) were initially subjected to additional Agencourt AMPure XP (Beckman Coulter, Brea, CA) bead-based purification to retain fragments >100 bp using a 40:50 vol/vol ratio. After size selection, targeted enrichment and library preparation using the Agilent SureSelect XTHS V2 system (Santa Clara, CA, USA) and our custom bait design was performed using a minimum input of 100 ng purified DNA. Initial enzymatic fragmentation employing the Agilent XT Low Input Enzymatic Fragmentation Kit was used to generate short (150-250 bp) fragments which were subsequently end-repaired, dA-tailed, and adapter ligated for Illumina paired-end sequencing with multiplexing, following manufacturer recommendations. After probe-based hybridization following the 90-min PCR protocol, capture using MyOne streptavidin T1 beads (Thermo Fisher Scientific, Waltham, MA, USA) was increased to 2 hours with minimized vortexing (1200 rpm). Additional quality control steps were followed, including use of a Qubit 4.0 fluorometer (Thermo Fisher Scientific, Waltham, MA, USA) and TapeStation 4200 for gDNA analysis (Agilent, Santa Clara, CA, USA), as well as high-sensitivity TapeStation 4200 analysis for pre- and post-capture libraries.

After pooling libraries, a KAPA qPCR Library Quantification kit (Roche, Basel, Switzerland) was used to confirm functionality of the barcoded pool, and a MiSeq Nano run (V2 chemistry, 2x150bp paired-end reads) was used to assess final barcode balance. The final multiplexed

library was sequenced by the University of Minnesota Genomics Center on a single lane of a NovaSeq6000 (Illumina, San Diego, CA, USA) with S4 cell chemistry to obtain 2x150 paired-end reads (675 Gb/lane), with an expected depth of ~54 M paired-end reads per sample.

16S rRNA bioinformatic analysis

Amplicon primers were trimmed from the 5' and 3' ends of forward and reverse reads using cutadapt with default settings [90]. The trimmed sequences were then input to the DADA2 v1.26 pipeline [91] to generate amplicon sequence variants (ASVs). The *filterandtrim* function was used for additional quality trimming, including truncation of forward and reverse reads to 250 bp and 220 bp as well as filtering of phiX reads and reads with a maximum expected error rate >4. Cleaned sequence reads were used as input to the *learnerrors* function and the output error-rate matrix was used in read error correction (i.e., denoising) using the *dada* function. Error corrected reads were aligned and combined into contigs using the *mergepairs* function with the minimum overlap threshold set to 12 bp. An ASV table was generated after removing chimeric contigs using the *removechimera* function. ASVs with a sequence length between 401 and 431 were retained. The *assigntaxonomy* function was used for taxonomic assignment of ASVs using the SILVA v138.2 reference database [92] via the native Bayesian classifier, and species annotation was performed via the *addspecies* function. Potential contaminating ASVs were identified using 16S qPCR copy number results per μl using *isContaminant* function in the Decontam v3.6 R package as implemented in the frequency method [93], and were removed from downstream analysis. The final ASV count matrix with taxonomy file was generated from the DADA2 pipeline and saved in RDS file format for subsequent downstream analysis.

Targeted shotgun metagenomics bioinformatic analysis

An alignment-based approach was used to detect ARG and MGE target sequences in all enriched metagenomic data, as implemented in the AMRPlusPlus v2.0 pipeline [86]. Briefly, read trimming and quality filtering was performed using TRIMMOMATIC v0.33 [94]. Host reads were identified in worker swab and swine/environmental samples by aligning sequence reads to the *Homo sapiens* (hg19) and *Sus scrofa* reference genomes, respectively, using BWA v0.7.17 [95]. Non-host reads were then extracted using SAMtools v1.9 [96] and aligned to the MEGARes v2.0 reference database using BWA-MEM. To mitigate the impact of mobilome misclassification due to sequence homology between MGEs and ARGs [97], we developed a custom script (extract_paired_unmapped.py) to extract paired reads not aligned to any resistome accessions. The ARG-filtered reads were separately aligned to sequences of a concatenated MGE database for ICE, plasmid replicons, plasmid modules, prophage, and virus (bacteriophage) extracted from ICEberg v2.0, PlasmidFinder v2.0.2, and ACLAME v0.4 [98]. For PlasmidFinder, only replicon accessions for Gram-positive bacteria and *Enterobacteriaceae* were included. Hits to accessions of plasmid modules, prophage, and virus / bacteriophage within the ACLAME database were further parsed for insertional sequences (IS) and other transposable elements (TEs) utilizing a custom scripting process that automates interfacing with ISfinder [99] and ISbrowser [100] (https://github.com/IS233489/IS_Finder_Annotation). Briefly, this script takes as input reads that are aligned to any aforementioned ACLAME accessions, and uses Entrez queries of the associated NCBI GenBank accessions. Each resulting query outputs metadata details for the accession (e.g., accession_id, start, stop, gene_description, locus name) and the associated full sequences. The Selenium webdriver for Python v4.8.3 is used to automate submission of the GenBank-queried ACLAME sequences to ISbrowser. The native BLASTN

algorithm is used to parse hits for plasmid, prophage, and virulence accessions, to detect and differentiate IS families and related TEs. Query hits for genes were retained if they met the following criteria: minimum identity of 80%, >80% coverage of the query length; and an E-value $\leq 1 \times 10^{-10}$. When multiple hits with similar threshold values were obtained, the assignment with the highest bitscore was chosen. BLASTN results were parsed and each sequence query was called as “likely IS”, “likely TE”, or “unclassified” and flagged for additional manual analysis if an accession was discontinued from further curation in NCBI GenBank. Additionally, all ACLAME accessions not identified as IS or TE were manually checked against current GenBank annotations, and grouped according to putative functional attributes as confirmed with Universal Protein KnowledgeBase (UniProtKB) annotation [101], including: biosynthesis regulation, non-conjugative efflux and transport, relaxase and mobilization machinery, replication initiation and maintenance, plasmid replicon module, DNA secretion and conjugative machinery, stress-SOS-tox/anti-tox-partitioning, transcription and translation regulation, transposition and recombination, and virulence and pathogenicity domains. Accessions with unconfirmed protein function and unassigned annotation in GenBank were considered “unclassified”, and accessions with both an unconfirmed protein function and an unsupported GenBank accession were categorized as “hypothetical”.

To reduce false positive detection of ARGs and MGEs, only ARG and MGE accessions attaining a conservative gene fraction cut-off of 80% within a given sample were considered positively identified, using the default settings in AMRPlusPlus v2.0. Gene fraction was defined as the proportion of nucleotides within a given accession aligned by at least one sequence read. The default deduplication procedures of the AMRPlusPlus v2.0 pipeline were used to account for potential amplification bias introduced during the molecular target enrichment process described above [86]. Identified ARGs and associated alignment counts were aggregated at the ‘group’, ‘mechanism’, and ‘class’ levels using the standard MEGARes ontology. Additionally, 29 ARG groups were flagged as ‘clinically important’ (i.e., priority ARGs) due to their prevalence in clinical disease and frequent co-occurrence with MGEs [36,37,102]: *bla*$_{CTX\text{-}M}$, *bla*$_{GES}$, *bla*$_{IMI}$, *bla*$_{KPC}$,*bla*$_{SHV}$, *bla*$_{TEM}$, *bla*$_{IMP}$, *bla*$_{NDM}$, *bla*$_{CMY}$, *bla*$_{OXA}$, *mecA*, *mcr-1*, *mcr-2*, *vat*, *vga*, *vgb*, *bla*$_{SME}$, *cfr*, *aac(6')-I*, *bla*$_{Z}$, *bla*$_{VIM}$, *ermB*, *qnrA*, *qnrB*, *tetM*, *dfrA*, *vanY-b*, *vanY-d*, *vanY-a*, *sulI*. Prior to analysis, all hits to ARG accessions requiring SNP confirmation (signified by the “*RequiresSNPConfirmation*” in the MEGARes header) were removed, as additional confirmatory assessments would be needed to ensure accurate detection of these genes. Since no mobilome-wide ontology currently exists, counts for MGEs were aggregated to the HGT mechanism type (i.e., ICE, plasmid, prophage, virulence, and IS/TE).

Assessment of sequencing depth, quality, and host genome abundance

Microbiome sequencing variables, including the number of raw reads generated from 16S rRNA sequencing, 16S copy number, and 16S mean quality score were assessed with respect to the following independent variables: collection phase (i.e. Workday start, Workday end, Post-shower, Swine, and Environment), worker exposure type (i.e., direct vs. indirect), and sample type (i.e., human skin swab, swine skin swab, standard mock microbial community, and negative control). Enriched metagenomic sequencing variables, including total generated raw reads (i.e. sequencing depth), total number of host vs. non-host reads, and mean quality score were similarly assessed with respect to collection phase, worker exposure type, and sample type. Associations between these independent variables on 16S and shotgun metagenomic sequencing

metrics were independently quantified via linear mixed models using the *lme4* v1.1-29 package [103] with subject ID as random effect to account for repeated measures. Statistical significance of each independent variable was evaluated using ANOVA with a pre-defined alpha of 0.05. All independent variables in the final models were subjected to Tukey-adjusted pairwise comparisons using the Emmeans v2.30-0 package [104], again with a predefined alpha of 0.05. Statistically significant variables were included as covariates in all subsequent models to account for potential confounding related to sequencing effort and/or quality. Assumptions of normality for dependent variables were assessed using visual inspection of residuals, and $\log_{10}$ variable transformations were applied to meet model assumptions.

Quantification of microbiome, resistome, and mobilome diversity and abundance

Richness and Shannon's diversity were estimated for each collection phase and for swine samples using the *estimate_richness* function in the Phyloseq v3.20 package under the Bioconductor release repository. Diversity metrics were calculated at the genus level for 16S data; at the group level for ARGs; and at the mechanism level for MGEs. Differences in diversity were evaluated using linear mixed models via the *lme4* function, specifying 16S copy number (and host-removed sequencing depth for enriched shotgun data) as covariates and worker ID as a random effect. Model building and extraction of model estimates were performed as reported above for the assessment of sequencing depth and quality. Rarefaction analysis and relative abundance analysis of genera, ARG groups, and MGE accessions was performed using the MicrobiotaProcess R package v1.18 [105]. Due to the limited sample size, the 'Environment' samples were excluded from all diversity analyses.

Prior to assessing ***β***-diversity, genera not present in the 16S data or ARGs / MGEs absent from shotgun data across all human and swine samples and were handled using a compositional approach [106] to imputation of zero-inflated count matrices performed using the zCompositions package v1.5.0-4 [107] calling the geometric Bayesian-multiplicative replacement of zero counts function *cmultRepl*, which outputs pseudo-counts. To account for differences in sequencing depth and to attenuate the influence of highly abundant accessions, robust center log ratio (rclr) normalization was applied to all microbiome, resistome, and mobilome counts, which were then transformed to Euclidean distances using the *vegdist* and *decostand* functions in the Vegan package v2.6-8 [108]. The *ordinate* function in Phyloseq was applied to the resulting Aitchison compositions using principal component analysis (PCA). As with the alpha-diversity metrics, beta-diversity was assessed at the genus level for 16S data; at the group level for ARGs; and at the mechanism level for MGEs; with a predefined alpha of 0.05 for all statistical tests. Differences by collection phase were first assessed via the omnibus analysis of similarities test (ANOSIM). Permutational multivariate analysis of variance (PERMANOVA) was performed using the *adonis* function in Vegan based on 10,000 permutations. *Post-hoc* pairwise comparisons were performed using the *pairwise.adonis* function as well as *adonis2* with stratification on worker ID for skin samples when comparing across collection phases T1–T3. As both ANOSIM and PERMANOVA tests are susceptible to dispersion heterogeneity, which may confound between-group with within-group variance, ***β***-dispersion was assessed via the *betadisper* function in Vegan.

Resistome and mobilome feature counts were utilized to analyze the total ARG and MGE sample load. This was done using a modified approach of Li et al [109] where the gene feature counts are expressed on the basis of the sample qPCR 16S gene copy number as well as the reference feature sequence length. This 'abundance' of the resistome or mobilome was then log_{10}-normalized and summarized by collection phase. Statistical differences in resistome or mobilome abundance were assessed using the linear mixed model approach and pairwise comparisons as described for sequencing and diversity assessments.

Microbiome, resistome, and mobilome differential abundance analysis

Raw count matrices from the microbiome, resistome, and mobilome were utilized as input for differential abundance testing. In addition to filtering for sparse features as described in the beta-diversity analysis, ARGs and MGEs were subjected to additional pruning; specifically, ARGs and MGEs with < 80% prevalence in any of the three collection phases (T1, T2 or T3) or the swine samples were removed from differential abundance analysis. Additionally, for each pairwise set of collection phases being compared during differential abundance analysis, features were censored if they had <10% prevalence and <10 counts. Prior to each differential abundance test, ARGs were agglomerated to both the group and mechanism levels, while MGE accessions were not subjected to agglomeration as no unified MGE taxonomy exists.

All count matrices and associated metadata stored as phyloseq objects were passed to the DESeq2 v1.46.0 R package [110]. The *poscounts* option was used for estimation of size factors which utilizes a modified relative log expression to account for missing alleles across samples. Estimates of dispersions were based on the negative binomial likelihood for each allele as implemented natively in DESeq2. Shrinkage of dispersion estimates and subsequent log_2-fold change (Log_2FC) effect sizes were performed via the empirical Bayes method of adaptive shrinkage implemented in the ashr R package to minimize the false discovery rate (FDR) [111]. Log_2FC estimation was performed using results of a negative binomial generalized linear model specifying collection phase as the main predictor and library cDNA concentration, host-removed sequencing depth, gender, BMI, smoking status, and consumption of pork as model covariates. Hypothesis testing was performed with the default Wald test and FDR adjustment. Differential abundance testing was performed for the following comparisons: workday start (T1) vs. workday end (T2); workday end (T2) vs. post-shower (T3); workday start (T1) vs. post-shower (T3); and the 'interfacing period' represented by swine vs. T2 samples. To guard against spurious findings, pre-defined cut-offs for statistical significance and effect size were used. At the genus level, we used an adjusted alpha of 0.05 and an effect size (log_2FC) of +/-1.5. For ARG group and MGE mechanism levels, we used an adjusted alpha of 0.01 and an effect size (log_2FC) of +/-1.5; for ARGs at the mechanism level, the adjusted alpha was increased to 0.05.

Inferring microbial community connectivity and ecological dominance from sparse datasets

The SParse InversE Covariance Estimation for Ecological Association Inference (SPIEC-EASI) [112] approach was deployed as implemented in R v1.1.1 to infer and analyze ecological networks and keystone members of the microbiome. Separate networks were built for each collection phase and for the swine samples. To build each network, we fit a negative binomial distribution to *clr*-normalized ASV count data. For input, ASV counts found in >1 sample with >100 raw counts were included in all network analyses, based on recommended filtering

procedures [112]. Since only two samples were collected for the ‘Environment’ collection phase, these samples were not assessed. Model inferences were performed using the Meinshausen and Bühlmann neighborhood selection framework [113], and model sparseness was inferred using the Stability Approach to Regularization Selection (StARS) as implemented in the Pulsar R package v0.3.10 [114] with parameters *lambda.min.ratio*= 0.01 and *nlambda*=20. Stable networks were produced under subsampling with 100 rounds, and subsequently analyzed and visualized using igraph v2.0 R package [115]. Community detection procedures based on analysis of optimized ‘*spin state*’ configurations [116] were applied and modular clustering of subcommunities was evaluated based on Newman and Girvan [117] global modularity (*Q*) estimation. Network assortativity based on modular identity and node degree was evaluated using base igraph procedures. Eigenvector centrality was regressed according to degree centrality for each ASV (node) to estimate putative keystone membership within each network by identifying taxa in the top 5th percentile.

Identifying strain-level detail in targeted metagenomes

Trimmed and host-removed target-enriched metagenomic reads were mapped to the 1.1M taxonomic markers contained in the MetaPhlAn4 v4.1 [118] database using bowtie2 and default parameters [119]. Clade-specific marker coverage and normalization across all detected clades using default parameters produced a relative abundance taxonomic profile for each sample. The sample-specific abundance profiles were merged into a single matrix using the MetaPhlAn4 utility script (merge_metaphlan_tables.py). The matrix was then filtered to species prevalence ≥ 75% of samples. Alignment results on each sample from MetaPhlan3 were used to create files of consensus marker genes for each species. Additional marker sequences were extracted from the MetaPhlAn4 database and in turn were blasted against *Lactobacillus amylovorus* (RefSeq accession: GCA_000194115.1) The sample and reference reconstructed strain marker files were inputted into StrainPhlAn v4.1 (available in MetaPhlAn4), which filters based on the presence of selected clade markers. Multiple sequence alignments for each marker were created by calling PhyloPhlAn (available in MetaPhlAn4) for phylogenetic reconstruction using RAxML and based on default PhyloPhlAn bootstrap values. StrainPhlAn results were used to confirm the presence of specific species of bacteria that had been identified via 16S analysis. StrainPhlAn results were also used to infer sample-level ‘*sharing*’ of strains across collection phases. Briefly, consensus sequences for taxonomic markers were identified by alignment via the MetaPhlan4 pipeline across all metagenomic samples. Major species-level genome bins (SGBs) for all Staphylococci, Streptococci, and *Escherichia coli* identified in initial StrainPhlan results were used to extract SGB marker genes. After extracting SGBs (*Staphylococcus epidermidis*: SGB7865; *Staphylococcus haemolyticus*: SGB7860, SGB7861; *Staphylococcus hominis*: SGB7858; *Escherichia coli*: SGB10068; *Streptococcus suis*: SGB8209, SGB29820; *Streptococcus alactolyticus*: SGB8017), strain sharing was assessed by profiling the maximum similarity across as many samples as possibles using the required inputs and the following parameters: *strainphlan --mutation_rates --marker_in_n_samples 1 --sample_with_n_markers 10 --phylophlan_mode accurate*. Pairwise alignment between targeted SGB markers from each sample pair were aligned and an RAxML phylogenetic distance was inferred for any putative transmission events.

De novo assembly and binning of genomes from targeted metagenomes

Adapter-trimmed and host-removed target-enriched metagenomic reads were used as input for metagenomic assembly with MEGAHIT v1.2.9 [120]. Single-sample assembly for each of the 42 metagenomes was carried out using default parameters. In addition, the 42 metagenomes were co-assembled using MEGAHIT on forward and reverse reads with options: *--continue --kmin-1pass --min-contig-len 1000*. Contigs >2000bp were mapped to single and co-assemblies via BWA-MEM [95], and SAMtools was used to sort and convert SAM files to BAM files [96]. The MetaBAT2 v2.15 [121] pipeline was used to assess coverage of assembled contigs and for both single- and co-assembly binning. Co-assemblies and single-sample assemblies were constructed separately to produce 638 and 712 bins, respectively. All bins were aggregated and dRep v3.4.0 [122] was used for *post hoc* dereplication with the following flags: *dereplicate -comp 80 -con 10 -sa 0.95* and CheckM v1.2.2 [123] lineage workflow was used to retain only those MAGs with contamination <10 % and completeness >80% using *--checkM_method lineage_wf*. After retaining all primary bins and only the highest-scoring secondary cluster bins, we produced 145 metagenome-assembled genomes (i.e., MAGs) from co-assemblies and 73 MAGs from single-sample assemblies. MAGs were assigned a taxonomy using GTDB-tk v2.1.1 [124], and of the 218 MAGs, 5 were assigned to archaea and not considered in further downstream analysis. Of the remaining 213 draft genomes, >57% (n= 123) were categorized as '*high-quality*', >34% (n= 74) as '*medium- quality*' with minimal contamination, and <8% (n=16) as '*medium-quality*' draft genomes (**Figure 7a**). We detected no significant differences (Type III ANOVA p>0.1) in genomic parameters for recovered MAGs across T1–T3, swine, and environmental samples (**Supplementary datafile 12**), including GC ratio (median range: 35.5–37.4), ANI distribution (median range: 98.2–98.8%), and N50 contig length distribution (median range: 11,949–15,798 bp). MAGs were assessed for their taxonomic novelty by following the procedures of Glendinning et al. [125] Briefly, MAGs with ANI <95% were considered as putatively novel species, and ANI <99% as putatively novel strains. Additionally, MAGs not assigned a provisional genus name were assessed for genus-level novelty via CompareM v0.1.2 at <60% AAI. Multiple sequence alignment files generated from the GTDB-tk workflow were concatenated across all collection phases and used as input for phylogenomic clustering as implemented in IQTREE v2.2.0 [126]. A best-fit substitution model was chosen using the native *ModelFinder Plus* via the Bayesian Information Criterion (BIC). The resulting model (LG+R8) was used to construct the taxonomic tree of MAGs, which was visualized across all study samples using iTOL [127].

ETHICAL APPROVAL AND CONSENT TO PARTICIPATE

The University of Minnesota's Institutional Review Board approved the study (protocol: *STUDY 00007351*) as no greater than minimal risk to study participants. All procedures, including obtaining informed consent, were followed in accordance with the ethical stands of the Office for Human Research Protections (U.S. Department of Health and Human Services) and with the Helsinki Declaration (2013). All animals were sampled under authorization from the Institutional Animal Care and Use Committees of the University of Minnesota and participating farms under a collaborative agreement (protocol #5-19).

DATA AVAILABILITY

Upon publication, raw sequence data and sample metadata can be accessed via the Sequence Read Archive (SRA) hosted by the National Center for Biotechnology Information (NCBI) under BioProject PRJNA987158. Sample metadata was recorded using the MIMARKS host-associated metagenomic sample guidelines of Yilmaz et al. [128]. All statistical analysis scripts were executed in R V4.4.0 and are publicly available at https://github.com/IS233489/LaborOME-project. The data sets supporting the results of this article are available in GigaDB v4.4.7 [129] and can be referenced at the persistent DOI: https://doi.org/10.5524/102696 [130]. An archived software heritage snapshot of the LaborOME project has been made publicly available [131].

AUTHOR CONTRIBUTIONS

IBS, NRN, SAD, JN, and CO conceptualized the study design. IBS and CO executed the experimental design and IBS conducted the laboratory work. IBS and PMF performed all bioinformatic analysis with guidance from CB and NRN. JEB was instrumental in generating parsing scripts to facilitate mobilome annotation. IBS and TNG performed quantitative analysis of resulting datasets. IBS conceptualized and edited graphical data in the manuscript with input from NRN. IBS and NRN wrote the manuscript with critical input from SAD, JN, CO, JEB, and CB. All authors read and approved the final version of the manuscript.

FUNDING

Financial support for this work was provided by the National Institute of Health (NIH) National Institute of Allergy and Infectious Disease (NIAID), *Project No. 1R01AI141810-01*; the Midwest Center for Occupational Safety and Health (MCOHS) Pilot Projects Research Training Program (PPRTP) funded through the National Institute of Occupational Safety and Health (NIOSH), *Project No.* T42 OH008434; and the University of Minnesota Doctoral Dissertation Fellowship.

FIGURE CAPTIONS

Figure 1. Study overview. Farm workers from a commercial farrow-to-wean operation in the Midwestern United States were voluntarily enrolled into a single longitudinal microbiome sampling campaign during a typical 8-hour workday shift. For each worker, swab kits were used to self-collect samples from the epidermis in a standardized fashion by passing each swab across four body sites, achieving a single composite skin sample for the left and right body representing microbiomes from the manus, interdigital space, antecubital fossa, popliteal fossa, and axilla. Workers were asked to perform the first self-collection ('Sample **T1**') prior to entry into the swine facility (**1**). Workers underwent mandatory showering prior to entry into the animal holding areas (**2**). During the day shift, workers were observed handling animals or working in specific animal pens, and dorsal skin swabs (from withers to tail-base) were taken from contact-matched animals on a pooled multi-pen level. Additionally, a 15-minute questionnaire was administered to collect biometric, health, lifestyle, and occupational task performance information from each worker (**3**). In a similar fashion, self-collected skin samples were taken immediately upon conclusion of the workday ('Sample **T2**') (**4**). Workers underwent mandatory showering procedures immediately after exiting the animal holding areas (**5**) and a third self-collection of samples was performed ('Sample **T3**') after showering and immediately prior to exiting the farm facility (**6**).

Figure 2. Changes in skin microbial load, microbiome composition, and community structure. a. Genus-level worker skin microbiome ***β***-diversity across collection phases (T1–T3) and contact-matched swine skin samples using principal component ordination of robust Aitchison compositions. Within-group centroids and 95% confidence intervals are depicted with a large circle and shaded ellipsoids, respectively. **b.** Log_{10}-normalized 16S rRNA qPCR copy number / µl (y-axis), stratified by collection phase (x-axis). *** indicates statistical significance ($p<0.001$) of pairwise comparisons based on a linear regression model with Tukey's adjustment for multiple comparisons. **c.** General linear analysis of the log_{10}-normalized 16S rRNA qPCR copy number / µl (y-axis) and log_{10}-normalized hourly exposure to swine (x-axis) based on workers' estimates from daily task assignments indicates a negative correlation ($p= 0.01$) across all collection phases. Shaded areas represent the 95% confidence interval around the linear trendline. **d.** Worker skin microbiome networks across workday collection phases and for contact-matched swine were inferred from inverse covariance estimation for compositions based on centered-log ratios of subsetted ASV counts containing >100 counts and >10% prevalence per ASV per sample. Inferred networks consist of nodes representing ASVs colored by shared subcommunity membership. Edges between nodes represent a significant predicted positive (blue) or negative (gray) interaction. Reported topology characteristics include network connectivity based on the edge to node ratio (*E:N*), modularity (*Q*), subcommunity assortativity (r_n), and degree assortativity (r_d). **e**. Scatter plots of microbial constituents from the corresponding networks are displayed based on the log_{10}-normalized node eigenvector centrality (y-axis) and node degree centrality (x-axis). Taxa with the highest centrality measures (top right of the distribution) are considered to be critical connectors and major hubs in community networks, and thus putative keystone taxa. ASV-level nodes are colored based on their taxonomic classification at the Class level. Genus-level labels are displayed only for genera most likely to be keystone, i.e., >95th percentile of the plot distribution (top right) and least likely to be keystone, i.e.<5th percentile of the plot distribution (bottom left).

Figure 3. Workday fluctuations in worker skin resistome and mobilome composition, compared to contact-matched swine. Principal component ordination of robust Aitchison compositions for the **a** total resistome and **b** major components of the total mobilome, including plasmids, integrative conjugative elements, virus, prophage, insertional sequences, and transposable elements. Clustering significance was explored with the omnibus analysis of similarity (ANOSIM) test ($p<0.05$). Ordinations are summarized by depicting within-group centroids and shaded ellipsoids

representing the 90% confidence interval. The distribution of major mobilome accessions are displayed as relative abundance stacked bar graphs across collection phases and individual samples for **c** major MGE mechanisms; **d** plasmidic sequences; and. **e** insertional sequence family identity. Legend abbreviations include ICE: integrative conjugative element, IS: insertional sequences, Plasmid ARG: Plasmid-borne antimicrobial resistance genes, TE: Transposable elements; PREPIM: Plasmid replication initiation and maintenance; PTTREG: Plasmid transcription, translation, and regulation.

Figure 4. Occurrence of medically important ARGs on the skin of workers and swine. Unique medically important (i.e., priority) ARG alleles were identified at >99.9% alignment gene coverage across all collection phases (top ribbon annotation) and are displayed using a heatmap summarizing their sample-level relative abundance across each of the respective 19 ARG gene groups. The cladograms along the x-axis demonstrate the hierarchical clustering of samples according to their medically important resistome composition using optimal leaf sorting and euclidean distances. Four major subclades are colored and numbered. Major ARG group prevalence and median abundance across study samples are summarized via the associated barplots and boxplots along the y-axis.

Figure 5. Differential abundance analysis across collection phases and at the interface period between workers and pigs. Volcano plots are used to visualize differential abundance of unique **a** ARG groups and **b** MGE accessions in $\log_2$-fold change (x-axis) and $-\log_{10}P$ value (y-axis) of the global worker skin resistome or mobilome between key workshift collection phases: Workday start (**T1**) vs. workday end (**T2**); Workday end (**T2**) vs. post-shower (**T3**); and Workday start (**T1**) vs. post-shower (**T3**). An additional comparison is made between workday end and swine skin samples representing the worker's contact phase with animals (gray). Features with significant shift in abundance (Wald's $p<0.01$ with FDR adjustment for ARG group and MGE accessions) are displayed above the horizontal line, while biologically significant fold-change is demarcated by vertical dashed lines at 1.5 $\log_2$-fold change. Labels are displayed for only the 5 most abundant ARG groups and 10 most abundant MGEs significantly amplified ($\log_2$-fold change >1.5 or < -1.5) at each phase comparison. For each volcano plot, an associated pie chart displays the number of unique ARG groups and MGE accessions common to each of the workshift collection phases compared, as well as the proportion of the total differentially abundant MGEs associated with each phase.

Figure 6. Taxonomic diversity and novelty of resolved metagenomic assembled genomes (MAGs) recovered from the human-swine interface and ambient environment. a Scatter plot of points representing individual GTDB taxonomically binned MAGs according to their estimated percent completeness (x-axis) relative to their estimated percent contamination (y-axis). Point size is proportional to the quantified MAG GC-content. Pink, purple, and gray regions of the plot demarcate the density of 'high quality', 'medium quality-low contamination', and 'medium quality' MAGs retrieved across all collection phases. **b** Scatter plot of estimated MAG size is displayed based on the $\log_{10}$-normalized nucleotide count (x-axis) and the nearest assigned NCBI reference genome size expressed in $\log_{10}$-normalized nucleotide count (y-axis). A global regression line and 95% confidence interval (shaded region) is displayed and results of significance testing (R^2; $p<0.05$) using a generalized linear model are colored for MAGs recovered for each collection phase. **c** Phylogenomic tree of de-replicated and high-confidence MAGs recovered from target-enriched metagenomes across each collection phase (outer ring). The area below each leaf is colored according to the taxonomically assigned Phylum. Branches for proposed novel species (ANI <95%) are displayed in teal, and for proposed novel strains (ANI <99%) are displayed in purple.

REFERENCES

1. Wu BG, Kapoor B, Cummings KJ, Stanton ML, Nett RJ, Kreiss K, et al.. Evidence for Environmental–Human Microbiota Transfer at a Manufacturing Facility with Novel Work-related Respiratory Disease. *Am J Respir Crit Care Med*. American Thoracic Society - AJRCCM; 2020; doi: 10.1164/rccm.202001-0197OC.

2. Lai PS, Christiani DC. Impact of occupational exposure on human microbiota. *Curr Opin Allergy Clin Immunol*. 2019; doi: 10.1097/ACI.0000000000000502.

3. Lai PS, Allen JG, Hutchinson DS, Ajami NJ, Petrosino JF, Winters T, et al.. Impact of environmental microbiota on human microbiota of workers in academic mouse research facilities: An observational study. *PLOS ONE*. Public Library of Science; 2017; doi: 10.1371/journal.pone.0180969.

4. M. Marcelloni A, Chiominto A, Di Renzi S, Melis P, Wirz A, C. Riviello M, et al.. How Working Tasks Influence Biocontamination in an Animal Facility. *Applied Sciences*. Multidisciplinary Digital

Publishing Institute; 2019; doi: 10.3390/app9112216.

5. Sun J, Liao X-P, D'Souza AW, Boolchandani M, Li S-H, Cheng K, et al.. Environmental remodeling of human gut microbiota and antibiotic resistome in livestock farms. *Nature Communications*. Nature Publishing Group; 2020; doi: 10.1038/s41467-020-15222-y.

6. Song SJ, Lauber C, Costello EK, Lozupone CA, Humphrey G, Berg-Lyons D, et al.. Cohabiting family members share microbiota with one another and with their dogs. *Elife*. 2013; doi: 10.7554/eLife.00458.

7. Mucci N, Tommasi E, Chiarelli A, Lulli L, Traversini V, Galea R, et al.. WORKbiota: A Systematic Review about the Effects of Occupational Exposure on Microbiota and Workers' Health. *IJERPH*. 2022; doi: 10.3390/ijerph19031043.

8. Peng M, Biswas D. Environmental Influences of High-Density Agricultural Animal Operation on Human Forearm Skin Microflora. *Microorganisms*. 2020; doi: 10.3390/microorganisms8101481.

9. Yang D, Heederik DJJ, Scherpenisse P, Van Gompel L, Luiken REC, Wadepohl K, et al.. Antimicrobial resistance genes aph(3')-III, erm(B), sul2 and tet(W) abundance in animal faeces, meat, production environments and human faeces in Europe. *J Antimicrob Chemother*. 2022; doi: 10.1093/jac/dkac133.

10. Van Gompel L, Luiken REC, Hansen RB, Munk P, Bouwknegt M, Heres L, et al.. Description and determinants of the faecal resistome and microbiome of farmers and slaughterhouse workers: A metagenome-wide cross-sectional study. *Environ Int*. 2020; doi: 10.1016/j.envint.2020.105939.

11. Hammerum AM, Larsen J, Andersen VD, Lester CH, Skovgaard Skytte TS, Hansen F, et al.. Characterization of extended-spectrum β-lactamase (ESBL)-producing Escherichia coli obtained from Danish pigs, pig farmers and their families from farms with high or no consumption of third- or fourth-generation cephalosporins. *Journal of Antimicrobial Chemotherapy*. 2014; doi: 10.1093/jac/dku180.

12. Oppliger A, Moreillon P, Charrière N, Giddey M, Morisset D, Sakwinska O. Antimicrobial Resistance of Staphylococcus aureus Strains Acquired by Pig Farmers from Pigs. *Appl Environ Microbiol*. 2012; doi: 10.1128/AEM.01902-12.

13. Sun J, Yang M, Sreevatsan S, Bender JB, Singer RS, Knutson TP, et al.. Longitudinal study of Staphylococcus aureus colonization and infection in a cohort of swine veterinarians in the United States. *BMC Infect Dis*. 2017; doi: 10.1186/s12879-017-2802-1.

14. Hatcher SM, Rhodes SM, Stewart JR, Silbergeld E, Pisanic N, Larsen J, et al.. The Prevalence of Antibiotic-Resistant Staphylococcus aureus Nasal Carriage among Industrial Hog Operation Workers, Community Residents, and Children Living in Their Households: North Carolina, USA. *Environ Health Perspect*. 2017; doi: 10.1289/EHP35.

15. Chen D, Cheng K, Wan L, Cui C, Li G, Zhao D, et al.. Daily occupational exposure in swine farm alters human skin microbiota and antibiotic resistome. *Imeta*. 2024; doi: 10.1002/imt2.158.

16. Ding D, Zhu J, Gao Y, Yang F, Ma Y, Cheng X, et al.. Effect of cattle farm exposure on oropharyngeal and gut microbial communities and antibiotic resistance genes in workers. *Science of The Total Environment*. 2022; doi: 10.1016/j.scitotenv.2021.150685.

17. : Wages and Benefits for Farm Employees | Ag Decision Maker.

https://www.extension.iastate.edu/agdm/wholefarm/html/c1-60.html Accessed 2023 Mar 27.

18. : Swine Human Resources: Managing Employees – Hogs, Pigs, and Pork. https://swine.extension.org/swine-human-resources-managing-employees/ Accessed 2023 Mar 27.

19. : CDC Interim Guidance for Workers who are Employed at Commercial Swine Farms: Preventing the Spread of Influenza A Viruses | CDC. https://www.cdc.gov/flu/swineflu/guidance-commercial-pigs.htm (2020). Accessed 2023 Apr 1.

20. : USDA APHIS | Pork Producers. https://www.aphis.usda.gov/aphis/resources/pests-diseases/asf/asf-producers Accessed 2023 Apr 1.

21. Grice EA, Kong HH, Conlan S, Deming CB, Davis J, Young AC, et al.. Topographical and Temporal Diversity of the Human Skin Microbiome. *Science*. American Association for the Advancement of Science; 2009; doi: 10.1126/science.1171700.

22. Bouslimani A, Porto C, Rath CM, Wang M, Guo Y, Gonzalez A, et al.. Molecular cartography of the human skin surface in 3D. *Proceedings of the National Academy of Sciences*. Proceedings of the National Academy of Sciences; 2015; doi: 10.1073/pnas.1424409112.

23. Agostinetto G, Bozzi D, Porro D, Casiraghi M, Labra M, Bruno A. SKIOME Project: a curated collection of skin microbiome datasets enriched with study-related metadata. *Database*. 2022; doi: 10.1093/database/baac033.

24. Song Y, Chen K, Lv L, Xiang Y, Du X, Zhang X, et al.. Uncovering the biogeography of the microbial commmunity and its association with nutrient metabolism in the intestinal tract using a pig model. *Front Nutr*. Frontiers; 2022; doi: 10.3389/fnut.2022.1003763.

25. Heras-Molina A, Estellé J, Vázquez-Gómez M, López-García A, Pesantez-Pacheco J-L, Astiz S, et al.. The impact of host genetics on porcine gut microbiota composition excluding maternal and postnatal environmental influences. *PLoS One*. 2024; doi: 10.1371/journal.pone.0315199.

26. Emami NK, Schreier LL, Greene E, Tabler T, Orlowski SK, Anthony NB, et al.. Ileal microbial composition in genetically distinct chicken lines reared under normal or high ambient temperatures. *Animal Microbiome*. 2022; doi: 10.1186/s42523-022-00183-y.

27. Duggett NA, Kay GL, Sergeant MJ, Bedford M, Constantinidou CI, Penn CW, et al.. Draft Genome Sequences of Six Novel Bacterial Isolates from Chicken Ceca. *Genome Announcements*. American Society for Microbiology; 2016; doi: 10.1128/genomea.00448-16.

28. Buiatte V, Fonseca A, Alonso Madureira P, Nakashima Vaz AC, Tizioto PC, Centola Vidal AM, et al.. A comparative study of the bacterial diversity and composition of nursery piglets' oral fluid, feces, and housing environment. *Sci Rep*. Nature Publishing Group; 2024; doi: 10.1038/s41598-024-54269-5.

29. Vlasblom AA, Duim B, Patel S, Luiken REC, Crespo-Piazuelo D, Eckenberger J, et al.. The developing pig respiratory microbiome harbors strains antagonistic to common respiratory pathogens. *mSystems*. American Society for Microbiology; 2024; doi: 10.1128/msystems.00626-24.

30. Li A, Chu Y, Wang X, Ren L, Yu J, Liu X, et al.. A pyrosequencing-based metagenomic study of methane-producing microbial community in solid-state biogas reactor. *Biotechnology for Biofuels*. 2013; doi: 10.1186/1754-6834-6-3.

31. van Rensburg JJ, Lin H, Gao X, Toh E, Fortney KR, Ellinger S, et al.. The Human Skin Microbiome Associates with the Outcome of and Is Influenced by Bacterial Infection. *mBio*. American Society for Microbiology; 2015; doi: 10.1128/mbio.01315-15.

32. Flowers L, Grice EA. The Skin Microbiota: Balancing Risk and Reward. *Cell Host & Microbe*. Elsevier; 2020; doi: 10.1016/j.chom.2020.06.017.

33. Strube ML, Hansen JE, Rasmussen S, Pedersen K. A detailed investigation of the porcine skin and nose microbiome using universal and Staphylococcus specific primers. *Sci Rep*. Nature Publishing Group; 2018; doi: 10.1038/s41598-018-30689-y.

34. Arikan M, Yildiz Z, Kahraman Demir T, Yilmaz NH, Sen A, Hanoglu L, et al.. Axillary Microbiota Is Associated with Cognitive Impairment in Parkinson's Disease Patients. *Microbiology Spectrum*. American Society for Microbiology; 2022; doi: 10.1128/spectrum.02358-21.

35. Noyes NR, Weinroth ME, Parker JK, Dean CJ, Lakin SM, Raymond RA, et al.. Enrichment allows identification of diverse, rare elements in metagenomic resistome-virulome sequencing. *Microbiome*. 2017; doi: 10.1186/s40168-017-0361-8.

36. Zhang A-N, Gaston JM, Dai CL, Zhao S, Poyet M, Groussin M, et al.. An omics-based framework for assessing the health risk of antimicrobial resistance genes. *Nat Commun*. 2021; doi: 10.1038/s41467-021-25096-3.

37. Nielsen TK, Browne PD, Hansen LH. Antibiotic resistance genes are differentially mobilized according to resistance mechanism. *GigaScience*. 2022; doi: 10.1093/gigascience/giac072.

38. Davis MF, Pisanic N, Rhodes SM, Brown A, Keller H, Nadimpalli M, et al.. Occurrence of Staphylococcus aureus in swine and swine workplace environments on industrial and antibiotic-free hog operations in North Carolina, USA: a One Health pilot study. *Environ Res*. 2018; doi: 10.1016/j.envres.2017.12.010.

39. May L, Klein EY, Rothman RE, Laxminarayan R. Trends in Antibiotic Resistance in Coagulase-Negative Staphylococci in the United States, 1999 to 2012. *Antimicrobial Agents and Chemotherapy*. American Society for Microbiology; 2014; doi: 10.1128/aac.01908-13.

40. Becker K, Heilmann C, Peters G. Coagulase-Negative Staphylococci. *Clin Microbiol Rev*. 2014; doi: 10.1128/CMR.00109-13.

41. Barros EM, Ceotto H, Bastos MCF, dos Santos KRN, Giambiagi-deMarval M. Staphylococcus haemolyticus as an Important Hospital Pathogen and Carrier of Methicillin Resistance Genes. *J Clin Microbiol*. 2012; doi: 10.1128/JCM.05563-11.

42. Costa SS, Sobkowiak B, Parreira R, Edgeworth JD, Viveiros M, Clark TG, et al.. Genetic Diversity of norA, Coding for a Main Efflux Pump of Staphylococcus aureus. *Frontiers in Genetics*. 92019;

43. Houdt RV, Toussaint A, Ryan MP, Pembroke JT, Mergeay M, Adley CC. The Tn4371 ICE Family of Bacterial Mobile Genetic Elements. Madame Curie Bioscience Database [Internet]. Landes Bioscience;

44. Razavi M, Kristiansson E, Flach C-F, Larsson DGJ. The Association between Insertion Sequences and Antibiotic Resistance Genes. *mSphere*. American Society for Microbiology; 2020; doi: 10.1128/mSphere.00418-20.

45. Roberts AP, Mullany P. Tn916-like genetic elements: a diverse group of modular mobile elements conferring antibiotic resistance. *FEMS Microbiology Reviews*. 2011; doi: 10.1111/j.1574-6976.2011.00283.x.

46. Howden BP, Seemann T, Harrison PF, McEvoy CR, Stanton J-AL, Rand CJ, et al.. Complete Genome Sequence of Staphylococcus aureus Strain JKD6008, an ST239 Clone of Methicillin-Resistant Staphylococcus aureus with Intermediate-Level Vancomycin Resistance. *Journal of Bacteriology*. American Society for Microbiology; 2010; doi: 10.1128/JB.00951-10.

47. Sanderson H, Ortega-Polo R, Zaheer R, Goji N, Amoako KK, Brown RS, et al.. Comparative genomics of multidrug-resistant Enterococcus spp. isolated from wastewater treatment plants. *BMC Microbiol*. 2020; doi: 10.1186/s12866-019-1683-4.

48. Bellanger X, Payot S, Leblond-Bourget N, Guédon G. Conjugative and mobilizable genomic islands in bacteria: evolution and diversity. *FEMS Microbiology Reviews*. 2014; doi: 10.1111/1574-6976.12058.

49. Sebaihia M, Wren BW, Mullany P, Fairweather NF, Minton N, Stabler R, et al.. The multidrug-resistant human pathogen Clostridium difficile has a highly mobile, mosaic genome. *Nat Genet*. 2006; doi: 10.1038/ng1830.

50. Welter DK, Ruaud A, Henseler ZM, De Jong HN, van Coeverden de Groot P, Michaux J, et al.. Free-Living, Psychrotrophic Bacteria of the Genus Psychrobacter Are Descendants of Pathobionts. *mSystems*. American Society for Microbiology; 2021; doi: 10.1128/mSystems.00258-21.

51. Byrne-Bailey KG, Gaze WH, Kay P, Boxall ABA, Hawkey PM, Wellington EMH. Prevalence of sulfonamide resistance genes in bacterial isolates from manured agricultural soils and pig slurry in the United Kingdom. *Antimicrob Agents Chemother*. 2009; doi: 10.1128/AAC.00652-07.

52. Cobo-Díaz JF, Alvarez-Molina A, Alexa EA, Walsh CJ, Mencía-Ares O, Puente-Gómez P, et al.. Microbial colonization and resistome dynamics in food processing environments of a newly opened pork cutting industry during 1.5 years of activity. *Microbiome*. 2021; doi: 10.1186/s40168-021-01131-9.

53. Ingham AC, Urth TR, Sieber RN, Stegger M, Edslev SM, Angen Ø, et al.. Dynamics of the Human Nasal Microbiota and Staphylococcus aureus CC398 Carriage in Pig Truck Drivers across One Workweek. *Applied and Environmental Microbiology*. American Society for Microbiology; 2021; doi: 10.1128/AEM.01225-21.

54. Chen C, Zhou Y, Fu H, Xiong X, Fang S, Jiang H, et al.. Expanded catalog of microbial genes and metagenome-assembled genomes from the pig gut microbiome. *Nat Commun*. Nature Publishing Group; 2021; doi: 10.1038/s41467-021-21295-0.

55. Saheb Kashaf S, Proctor DM, Deming C, Saary P, Hölzer M, NISC Comparative Sequencing Program, et al.. Integrating cultivation and metagenomics for a multi-kingdom view of skin microbiome diversity and functions. *Nat Microbiol*. 2022; doi: 10.1038/s41564-021-01011-w.

56. Arikawa K, Ide K, Kogawa M, Saeki T, Yoda T, Endoh T, et al.. Recovery of strain-resolved genomes from human microbiome through an integration framework of single-cell genomics and metagenomics. *Microbiome*. 2021; doi: 10.1186/s40168-021-01152-4.

57. Liu G, Li P, Hou L, Niu Q, Pu G, Wang B, et al.. Metagenomic Analysis Reveals New Microbiota Related to Fiber Digestion in Pigs. *Frontiers in Microbiology*. 122021;

58. Crossfield M, Gilroy R, Ravi A, Baker D, La Ragione RM, Pallen MJ. Archaeal and Bacterial Metagenome-Assembled Genome Sequences Derived from Pig Feces. *Microbiol Resour Announc*. 2022; doi: 10.1128/mra.01142-21.

59. Ahn J, Hayes RB. Environmental Influences on the Human Microbiome and Implications for Noncommunicable Disease. *Annual Review of Public Health*. 2021; doi: 10.1146/annurev-publhealth-012420-105020.

60. Rothschild D, Weissbrod O, Barkan E, Kurilshikov A, Korem T, Zeevi D, et al.. Environment dominates over host genetics in shaping human gut microbiota. *Nature*. 2018; doi: 10.1038/nature25973.

61. Gacesa R, Kurilshikov A, Vich Vila A, Sinha T, Klaassen M a. Y, Bolte LA, et al.. Environmental factors shaping the gut microbiome in a Dutch population. *Nature*. Nature Publishing Group; 2022; doi: 10.1038/s41586-022-04567-7.

62. Oh J, Byrd AL, Park M, Kong HH, Segre JA. Temporal Stability of the Human Skin Microbiome. *Cell*. 2016; doi: 10.1016/j.cell.2016.04.008.

63. Costello EK, Lauber CL, Hamady M, Fierer N, Gordon JI, Knight R. Bacterial Community Variation in Human Body Habitats Across Space and Time. *Science*. American Association for the Advancement of Science; 2009; doi: 10.1126/science.1177486.

64. Boxberger M, Cenizo V, Cassir N, La Scola B. Challenges in exploring and manipulating the human skin microbiome. *Microbiome*. 2021; doi: 10.1186/s40168-021-01062-5.

65. Larson KRL, Smith TC, Donham KJ. Self-reported Methicillin-resistant Staphylococcus aureus infection in USA pork producers.

66. Beaudoin A, Johnson S, Davies P, Bender J, Gramer M. Characterization of Influenza A Outbreaks in Minnesota Swine Herds and Measures Taken to Reduce the Risk of Zoonotic Transmission. *Zoonoses and Public Health*. 2012; doi: 10.1111/j.1863-2378.2011.01423.x.

67. Myers KP, Olsen CW, Setterquist SF, Capuano AW, Donham KJ, Thacker EL, et al.. Are swine workers in the United States at increased risk of infection with zoonotic influenza virus? *Clin Infect Dis*. 2006; doi: 10.1086/498977.

68. Chen C, Wu F. Livestock-associated methicillin-resistant Staphylococcus aureus (LA-MRSA) colonisation and infection among livestock workers and veterinarians: a systematic review and meta-analysis. *Occup Environ Med*. BMJ Publishing Group Ltd; 2021; doi: 10.1136/oemed-2020-106418.

69. Sieber RN, Skov RL, Nielsen J, Schulz J, Price LB, Aarestrup FM, et al.. Drivers and Dynamics of Methicillin-Resistant Livestock-Associated Staphylococcus aureus CC398 in Pigs and Humans in Denmark. *mBio*. American Society for Microbiology; 2018; doi: 10.1128/mBio.02142-18.

70. Sudatip D, Mostacci N, Thamlikitkul V, Oppliger A, Hilty M. Influence of occupational exposure to pigs or chickens on human gut microbiota composition in Thailand. *One health*. Elsevier; 15:1004632022;

71. Sudatip D, Mostacci N, Tiengrim S, Thamlikitkul V, Chasiri K, Kritiyakan A, et al.. The risk of pig and chicken farming for carriage and transmission of Escherichia coli containing extended-spectrum beta-lactamase (ESBL) and mobile colistin resistance (mcr) genes in Thailand. *Microbial Genomics*.

Microbiology Society,; 2023; doi: 10.1099/mgen.0.000951.

72. Maciel-Guerra A, Baker M, Hu Y, Wang W, Zhang X, Rong J, et al.. Dissecting microbial communities and resistomes for interconnected humans, soil, and livestock. *ISME J*. Nature Publishing Group; 2023; doi: 10.1038/s41396-022-01315-7.

73. Mencía-Ares O, Borowiak M, Argüello H, Cobo-Díaz JF, Malorny B, Álvarez-Ordóñez A, et al.. Genomic Insights into the Mobilome and Resistome of Sentinel Microorganisms Originating from Farms of Two Different Swine Production Systems. *Microbiology Spectrum*. American Society for Microbiology; 2022; doi: 10.1128/spectrum.02896-22.

74. Gao F-Z, He L-Y, He L-X, Bai H, Zhang M, Chen Z-Y, et al.. Swine farming shifted the gut antibiotic resistome of local people. *Journal of Hazardous Materials*. 2024; doi: 10.1016/j.jhazmat.2023.133082.

75. Kraemer JG, Aebi S, Oppliger A, Hilty M. The Indoor-Air Microbiota of Pig Farms Drives the Composition of the Pig Farmers’ Nasal Microbiota in a Season-Dependent and Farm-Specific Manner. *Applied and Environmental Microbiology*. American Society for Microbiology; doi: 10.1128/AEM.03038-18.

76. Luiken REC, Van Gompel L, Bossers A, Munk P, Joosten P, Hansen RB, et al.. Farm dust resistomes and bacterial microbiomes in European poultry and pig farms. *Environment International*. 2020; doi: 10.1016/j.envint.2020.105971.

77. Kirjavainen PV, Karvonen AM, Adams RI, Täubel M, Roponen M, Tuoresmäki P, et al.. Farm-like indoor microbiota in non-farm homes protects children from asthma development. *Nat Med*. Nature Publishing Group; 2019; doi: 10.1038/s41591-019-0469-4.

78. Alarcón LV, Alberto AA, Mateu E. Biosecurity in pig farms: a review. *Porcine Health Manag*. 2021; doi: 10.1186/s40813-020-00181-z.

79. Coffman VR, Hall DJ, Pisanic N, Nadimpalli M, McCormack M, Diener-West M, et al.. Personal protective equipment use during industrial hog operation work activities and acute lung function changes in a prospective worker cohort, North Carolina 2014–2015. *American Journal of Industrial Medicine*. 2021; doi: 10.1002/ajim.23260.

80. Slizovskiy IB, Oliva M, Settle JK, Zyskina LV, Prosperi M, Boucher C, et al.. Target-enriched long-read sequencing (TELSeq) contextualizes antimicrobial resistance genes in metagenomes. *Microbiome*. 2022; doi: 10.1186/s40168-022-01368-y.

81. Slizovskiy IB, Bonin N, Bravo JE, Ferm PM, Singer J, Boucher C, et al.. Factors impacting target-enriched long-read sequencing of resistomes and mobilomes. *Genome Res*. 2024; doi: 10.1101/gr.279226.124.

82. Bjerre RD, Hugerth LW, Boulund F, Seifert M, Johansen JD, Engstrand L. Effects of sampling strategy and DNA extraction on human skin microbiome investigations. *Sci Rep*. Nature Publishing Group; 2019; doi: 10.1038/s41598-019-53599-z.

83. Byrd AL, Belkaid Y, Segre JA. The human skin microbiome. *Nat Rev Microbiol*. 2018; doi: 10.1038/nrmicro.2017.157.

84. Human Microbiome Project Consortium. Structure, function and diversity of the healthy human

microbiome. *Nature*. 2012; doi: 10.1038/nature11234.

85. Gohl DM, Vangay P, Garbe J, MacLean A, Hauge A, Becker A, et al.. Systematic improvement of amplicon marker gene methods for increased accuracy in microbiome studies. *Nat Biotechnol*. Nature Publishing Group; 2016; doi: 10.1038/nbt.3601.

86. Doster E, Lakin SM, Dean CJ, Wolfe C, Young JG, Boucher C, et al.. MEGARes 2.0: a database for classification of antimicrobial drug, biocide and metal resistance determinants in metagenomic sequence data. *Nucleic Acids Res*. Oxford Academic; 2020; doi: 10.1093/nar/gkz1010.

87. Liu M, Li X, Xie Y, Bi D, Sun J, Li J, et al.. ICEberg 2.0: an updated database of bacterial integrative and conjugative elements. *Nucleic Acids Res*. 2019; doi: 10.1093/nar/gky1123.

88. Carattoli A, Zankari E, García-Fernández A, Voldby Larsen M, Lund O, Villa L, et al.. In Silico Detection and Typing of Plasmids using PlasmidFinder and Plasmid Multilocus Sequence Typing. *Antimicrob Agents Chemother*. 2014; doi: 10.1128/AAC.02412-14.

89. Metsky HC, Siddle KJ, Gladden-Young A, Qu J, Yang DK, Brehio P, et al.. Capturing sequence diversity in metagenomes with comprehensive and scalable probe design. *Nature Biotechnology*. Nature Publishing Group; 2019; doi: 10.1038/s41587-018-0006-x.

90. Martin M. Cutadapt removes adapter sequences from high-throughput sequencing reads. *EMBnet.journal*. 2011; doi: 10.14806/ej.17.1.200.

91. Callahan BJ, McMurdie PJ, Rosen MJ, Han AW, Johnson AJA, Holmes SP. DADA2: High-resolution sample inference from Illumina amplicon data. *Nature Methods*. Nature Publishing Group; 2016; doi: 10.1038/nmeth.3869.

92. Quast C, Pruesse E, Yilmaz P, Gerken J, Schweer T, Yarza P, et al.. The SILVA ribosomal RNA gene database project: improved data processing and web-based tools. *Nucleic Acids Research*. 2013; doi: 10.1093/nar/gks1219.

93. Davis NM, Proctor DM, Holmes SP, Relman DA, Callahan BJ. Simple statistical identification and removal of contaminant sequences in marker-gene and metagenomics data. *Microbiome*. 2018; doi: 10.1186/s40168-018-0605-2.

94. Bolger AM, Lohse M, Usadel B. Trimmomatic: a flexible trimmer for Illumina sequence data. *Bioinformatics*. 2014; doi: 10.1093/bioinformatics/btu170.

95. Li H. Aligning sequence reads, clone sequences and assembly contigs with BWA-MEM. arXiv;

96. Li H, Handsaker B, Wysoker A, Fennell T, Ruan J, Homer N, et al.. The Sequence Alignment/Map format and SAMtools. *Bioinformatics*. 2009; doi: 10.1093/bioinformatics/btp352.

97. Slizovskiy IB, Mukherjee K, Dean CJ, Boucher C, Noyes NR. Mobilization of antibiotic resistance: Are current approaches for colocalizing resistomes and mobilomes useful? *Front Microbiol*. Frontiers; 2020; doi: 10.3389/fmicb.2020.01376.

98. Leplae R, Lima-Mendez G, Toussaint A. ACLAME: a CLAssification of Mobile genetic Elements, update 2010. *Nucleic Acids Res*. 2010; doi: 10.1093/nar/gkp938.

99. Siguier P, Perochon J, Lestrade L, Mahillon J, Chandler M. ISfinder: the reference centre for bacterial insertion sequences. *Nucleic Acids Res*. 2006; doi: 10.1093/nar/gkj014.

100. Kichenaradja P, Siguier P, Pérochon J, Chandler M. ISbrowser: an extension of ISfinder for visualizing insertion sequences in prokaryotic genomes. *Nucleic Acids Research*. 2010; doi: 10.1093/nar/gkp947.

101. Apweiler R, Bairoch A, Wu CH, Barker WC, Boeckmann B, Ferro S, et al.. UniProt: the Universal Protein knowledgebase. *Nucleic Acids Res*. 2004; doi: 10.1093/nar/gkh131.

102. Doster E, Rovira P, Noyes NR, Burgess BA, Yang X, Weinroth MD, et al.. Investigating Effects of Tulathromycin Metaphylaxis on the Fecal Resistome and Microbiome of Commercial Feedlot Cattle Early in the Feeding Period. *Front Microbiol*. 2018; doi: 10.3389/fmicb.2018.01715.

103. Bates D, Mächler M, Bolker B, Walker S. Fitting Linear Mixed-Effects Models Using lme4. *Journal of Statistical Software*. 2015; doi: 10.18637/jss.v067.i01.

104. Lenth R, Love J, Herve M. emmeans: Estimated Marginal Means, aka Least-Squares Means.

105. Xu S, Zhan L, Tang W, Wang Q, Dai Z, Zhou L, et al.. MicrobiotaProcess: A comprehensive R package for deep mining microbiome. *The Innovation*. 2023; doi: 10.1016/j.xinn.2023.100388.

106. Quinn TP, Erb I, Gloor G, Notredame C, Richardson MF, Crowley TM. A field guide for the compositional analysis of any-omics data. *Gigascience*. 2019; doi: 10.1093/gigascience/giz107.

107. Palarea-Albaladejo J, Martín-Fernández JA. zCompositions — R package for multivariate imputation of left-censored data under a compositional approach. *Chemometrics and Intelligent Laboratory Systems*. 2015; doi: 10.1016/j.chemolab.2015.02.019.

108. Oksanen J. Vegan: an introduction to ordination. :122019;

109. Li B, Yang Y, Ma L, Ju F, Guo F, Tiedje JM, et al.. Metagenomic and network analysis reveal wide distribution and co-occurrence of environmental antibiotic resistance genes. *ISME J*. 2015; doi: 10.1038/ismej.2015.59.

110. Love MI, Huber W, Anders S. Moderated estimation of fold change and dispersion for RNA-seq data with DESeq2. *Genome Biology*. 2014; doi: 10.1186/s13059-014-0550-8.

111. Stephens M. False discovery rates: a new deal. *Biostatistics*. 2017; doi: 10.1093/biostatistics/kxw041.

112. Kurtz ZD, Müller CL, Miraldi ER, Littman DR, Blaser MJ, Bonneau RA. Sparse and Compositionally Robust Inference of Microbial Ecological Networks. *PLOS Computational Biology*. Public Library of Science; 2015; doi: 10.1371/journal.pcbi.1004226.

113. Meinshausen N, Bühlmann P. High dimensional graphs and variable selection with the LASSO. *The Annals of Statistics*. 2006; doi: 10.1214/009053606000000281.

114. Müller CL, Bonneau R, Kurtz Z. Generalized Stability Approach for Regularized Graphical Models. arXiv;

115. Csardi G, Nepusz T. The Igraph Software Package for Complex Network Research. *InterJournal*.

Complex Systems:16952005;

116. Reichardt J, Bornholdt S. Statistical Mechanics of Community Detection. *Phys Rev E*. 2006; doi: 10.1103/PhysRevE.74.016110.

117. Newman MEJ, Girvan M. Finding and evaluating community structure in networks. *Phys Rev E*. American Physical Society; 2004; doi: 10.1103/PhysRevE.69.026113.

118. Beghini F, McIver LJ, Blanco-Míguez A, Dubois L, Asnicar F, Maharjan S, et al.: Integrating taxonomic, functional, and strain-level profiling of diverse microbial communities with bioBakery 3. eLife. eLife Sciences Publications Limited; https://elifesciences.org/articles/65088/figures (2021). Accessed 2023 Apr 4.

119. Langmead B, Salzberg SL. Fast gapped-read alignment with Bowtie 2. *Nat Methods*. Nature Publishing Group; 2012; doi: 10.1038/nmeth.1923.

120. Li D, Liu C-M, Luo R, Sadakane K, Lam T-W. MEGAHIT: an ultra-fast single-node solution for large and complex metagenomics assembly via succinct de Bruijn graph. *Bioinformatics*. 2015; doi: 10.1093/bioinformatics/btv033.

121. Kang DD, Li F, Kirton E, Thomas A, Egan R, An H, et al.. MetaBAT 2: an adaptive binning algorithm for robust and efficient genome reconstruction from metagenome assemblies. *PeerJ*. 2019; doi: 10.7717/peerj.7359.

122. Olm MR, Brown CT, Brooks B, Banfield JF. dRep: a tool for fast and accurate genomic comparisons that enables improved genome recovery from metagenomes through de-replication. *ISME J*. 2017; doi: 10.1038/ismej.2017.126.

123. Parks DH, Imelfort M, Skennerton CT, Hugenholtz P, Tyson GW. CheckM: assessing the quality of microbial genomes recovered from isolates, single cells, and metagenomes. *Genome Res*. 2015; doi: 10.1101/gr.186072.114.

124. Chaumeil P-A, Mussig AJ, Hugenholtz P, Parks DH. GTDB-Tk v2: memory friendly classification with the genome taxonomy database. *Bioinformatics*. 2022; doi: 10.1093/bioinformatics/btac672.

125. Glendinning L, Stewart RD, Pallen MJ, Watson KA, Watson M. Assembly of hundreds of novel bacterial genomes from the chicken caecum. *Genome Biology*. 2020; doi: 10.1186/s13059-020-1947-1.

126. : IQ-TREE 2: New Models and Efficient Methods for Phylogenetic Inference in the Genomic Era | Molecular Biology and Evolution | Oxford Academic. https://academic.oup.com/mbe/article/37/5/1530/5721363 Accessed 2023 Mar 27.

127. : Interactive Tree Of Life (iTOL) v5: an online tool for phylogenetic tree display and annotation | Nucleic Acids Research | Oxford Academic. https://academic.oup.com/nar/article/49/W1/W293/6246398 Accessed 2023 Mar 27.

128. Yilmaz P, Kottmann R, Field D, Knight R, Cole JR, Amaral-Zettler L, et al.. Minimum information about a marker gene sequence (MIMARKS) and minimum information about any (x) sequence (MIxS) specifications. *Nat Biotechnol*. 2011; doi: 10.1038/nbt.1823.

129. Sneddon TP, Li P, Edmunds SC. GigaDB: announcing the GigaScience database. *GigaScience*.

2012; doi: 10.1186/2047-217X-1-11.

130. Slizovskiy I, Gaire T, Ferm P, Odland C, Dee S, Bravo J, et al.. Supporting data for: “Reducing Skin Microbiome Exposure Impacts Through Swine Farm Biosecurity” GigaScience Database.". GigaScience Database. 2025.

131. Slizovskiy I, Gaire T, Ferm P, Odland C, Dee S, Bravo J, et al. (2025) LaborOME-project (Version 1). [Computer software]. Software Heritage, https://archive.softwareheritage.org/swh:1:snp:0a6ab68851054df4232f28018b40947868ba0514

Personal Cover Click here to access/download;Personal Cover;CoverLetter.docx

April 28th, 2025

Dear editorial board,

Thank you for considering a revised version of our manuscript “*Reducing Skin Microbiome Exposure Impacts Through Swine Farm Biosecurity*” for publication in *GigaScience*.

We have addressed all remaining reviewer comments, and are in the process of confirming all associated data artefacts with the data curation team at GigaScience.

This manuscript has not been previously published and is not under review at any other journal. All listed authors have contributed significantly to this research based on the requirement for authorship guidelines and declare no conflicts of interest. The study from which data was generated was conducted in accordance with University of Minnesota and U.S. federal guidelines pertaining to human subject investigations, including the acquisition of informed consent following the review of study details and expectations with each participant. Steps to ensure security of any associated personal identifying information were taken. All raw sequencing data and metadata will be made accessible via the Sequenced Read Archive (SRA) under BioProject PRJNA987158. All statistical scripts are made publicly available at https://github.com/IS233489/LaborOME-project.

Thank you for your consideration of our submitted manuscript.

Sincerely,

Noelle Noyes

Dr. Noelle Noyes, MA, DVM, PhD
Associate Professor
University of Minnesota
1988 Fitch Avenue, St. Paul, MN, 55108
Office: 612-624-3562, Mobile: 617-953-7837
