# Supplementary material for: Reducing skin microbiome exposure impacts through swine farm biosecurity
Source: Gigascience. 2025 Jul 26;14:giaf062. doi: 10.1093/gigascience/giaf062 (PMC12810053; doi:10.1093/gigascience/giaf062)
Supplement: giaf062_Authors_Response_To_Reviewer_Comments_original_submission [file giaf062_authors_response_to_reviewer_comments_original_submission.pdf]

Author's Response To Reviewer Comments

RE: GIGA-D-24-00356
Reducing Skin Microbiome Exposure Impacts Through Swine Farm Biosecurity
Ilya Slizovskiy; Tara N Gaire; Peter M Ferm; Carissa A Odland; Scott A Dee; Joel Nerem; Jonathan E Bravo; Christina Boucher; Noelle R Noyes
Dear Editor and Reviewers,
We thank you for your thorough and thoughtful reviews of our manuscript "Reducing Skin Microbiome Exposure Impacts Through Swine Farm Biosecurity" (GIGA-D-24-00356). We appreciate your detailed feedback and constructive comments, which have been invaluable in improving our study. We have addressed all points raised and made the necessary revisions throughout the manuscript, including updates to the 16S rRNA analysis, methodological clarifications, and textual refinements.
Thank you again for your time and insightful suggestions.

- According to reviewer #1, the 16S reference database used for taxonomic assignment is outdated and should no longer be used; the analysis should be re-done with the up-to-date set.
Response: We re-analyzed all 16S-related sequencing data and have uploaded new figures / supporting files to reflect these changes. We have made alterations where 16S analysis is relevant to the text of the resubmission.

- Both reviewers have technical questions and concerns regarding the protocol and statistical analyses; for example with respect to using 16S data instead of direct metagenomic sequencing, and generally with respect to the sampling protocol.
Response: We addressed all concerns regarding our analytical and sequencing approaches. In addition, we re-analyzed metagenomic sequencing data with updated reference databases.

- Addressing the comments of Reviewer #2 in particular will also require a more careful discussion and more exact wording of conclusions, interpretations, and potential confounders.
Response: We made substantial modifications within the text describing the results and in particular within discussion of results, methods, and interpretations in the discussion section.

On an editorial note, please include the "supplemental methods" in the main manuscript. We have no word limit, and all methods that were used for obtaining the results described in the article should also be part of the main paper instead of the supplement.
Response: We incorporated all of the methods described in the supplemental section into the main document of the resubmission.

Reviewer #1:

The article titled "Reducing Skin Microbiome Exposure Impacts Through Swine Farm Biosecurity" is very interesting and well-structured. It provides solid methodological information and results, written in a detailed manner that makes it easy to follow. However, there are some major comments that need to be addressed, particularly related to the 16S rRNA sequencing database used for taxonomic assignment (Silva 132), which is from 2017 and does not reflect the important taxonomic changes made since then. Therefore, I highly recommend reanalyzing these data with an updated database. Below are the detailed comments.

General comments:

- Comment 1. Please include spaces between citations and the text. Review this structure throughout the manuscript.
Response: We noted this suggestion, and made changes to in-text citations throughout the main text and supplementary materials document.

- Comment 2. Greek letters (alpha, beta) appear as squares in several places throughout the text. Please review.
Response: Thank you for bringing this to our attention. After reviewing our document we believe this could have happened during the conversion step to PDF in the submission portal. We ensured that this did not appear in the resubmission stage.

Abstract:
- Comment 3. Line 51. Replace "U.S." with "United States".
Response: We have made the suggested change in the abstract, in line 47.

Introduction:
- Comment 4. Section title. Please replace "MAIN TEXT" with "INTRODUCTION".
Response: We have made the suggested change to the section title.

- Comment 5. Line 90. The phylum Proteobacteria is currently categorized as Pseudomonadota.
Response: We made the suggested change to the Phylum name of Proteobacteria, in line 80.

- Comment 6. Line 97. Please include a citation for studies tracking daily animal exposure and on-site worker behavior.
Response: We made the suggested change by adding two relevant citations, in lines 85–87.

- Comment 7. Line 102. Include the abbreviation for "United States" for further use throughout the manuscript.
Response: We made the suggested change by introducing the 'U.S.' abbreviation, in line 92.

Materials and methods - Supplementary Information
- Comment 8. Materials and Methods general comment. I understand the relevance of using a target-enriched metagenomic library preparation for AMR and MGE, given their low representation in samples. However, many studies assessing the mobilome and resistome conduct sequencing without prior enrichment, which reduces bias in the analysis, especially in uncharacterized MGEs. Additionally, this approach created the need for 16S rRNA sequencing analysis, which used an outdated database; this would not have been necessary with direct metagenomic sequencing without enrichment, enabling taxonomic assignment using updated tools such as Kraken. Furthermore, MetaPhlAn3 and MetaBAT2 were used for detecting taxonomic markers and MAGs binning, respectively, which introduces some bias due to AMR and MGE marker enrichment. Please explain the reasoning behind this approach and its potential limitations. Wouldn't it be possible to assign taxonomy directly from the metagenomic approach?
Response: We thank the reviewer for the opportunity to clarify these issues, and we address them here and in the main text of the manuscript, especially in the Discussion section:

Use of targeted enrichment vs. traditional metagenomics: The target-enriched shotgun sequencing approach was utilized for high-sensitivity resistome and mobilome analysis (Figures 3 and 4). We have considerable experience in the use of targeted metagenomic approaches for the characterization of resistomes and mobilomes in both short read (PMID: 29041965) and long-read (PMID: 36324140, PMID: 39500537) sequencing platforms. It has been established that targeted sequencing yields a more accurate characterization of low-abundance genes of public health importance, including ARGs and MGEs that are traditionally under-sequenced by standard shotgun metagenomics, by multiple orders of magnitude. However, even with targeted approaches, molecular enrichment in metagenomic workflows does not result in 100% on-target sequencing of targets—Indeed, our metadata (Supplementary datafile 1) demonstrates that the resistome and mobilome accounted for a median of 15% and 10% across all metagenomes analyzed in this study. The remaining >85% of the 'off-target' metagenomic data is still useful. We used this off-target data to perform MAG construction and select strain-level analyses that focused primarily on sentinel strains commonly associated with human and animal enteric and adnexal microbiomes (E. coli, Streptococci, and Staphylococci). Because the off-target sequence data may be biased (due to the enrichment and PCR amplification process), it is not suitable for comprehensive taxonomic analyses with metagenomic tools like Kraken. We therefore relied on the 16S rRNA amplicon sequencing approach for more robust taxonomic characterization. This approach yields reliable classification to the genus level. We

have added further discussion on the reasoning of using targeted-metagenomic approaches in the study, as well as their advantages and limitations in lines 638–660.

Target-enrichment approaches may bias sequencing: The reviewer notes that many studies utilize traditional metagenomic sequencing without any prior enrichment, and that this "...reduces bias in the analysis, especially in uncharacterized MGEs." We extended our discussion describing this method as well as its well-researched foundations, including work that has been extensively done in our labs over the years. We note that the study of uncharacterized genes was not the focus of our manuscript. Nevertheless, we would like to emphasize that the use of traditional shotgun metagenomic sequencing would not alleviate the issue of under-discovery of novel targets that the reviewer describes, because such data would need to still be compared to a database of known reference sequences either by direct read alignment, or the alignment of de novo assembled contigs. Because we utilized a targeted sequencing approach with built-in allelic flexibility, a larger proportion of sequencing effort was dedicated to ARG and MGE regions and their potential homologs than what otherwise would have been possible in traditional shotgun sequencing. Therefore, our approach may lead to less bias, particularly in terms of detection sensitivity.

Outdated reference database in the 16S rRNA analysis: We agree with both reviewers that at this point the 16S rRNA SILVA database that we originally used has become outdated. We therefore repeated our entire microbiome analyses using the most updated SILVA reference available, and we updated all of our microbiome results (lines 137–237). This resulted in changes to some of the key taxa that were found to be dominant at various work phase timepoints (and their taxonomic nomenclature), however, the overall patterns in alpha diversity, beta diversity, and network interactions paralleled the original results presented in the primary manuscript submission.

- Comment 9. The Silva database 132 is updated from December 13, 2017, meaning the taxonomic information is quite outdated, with significant changes in taxonomic classifications in the last seven years, such as phylum renaming. Therefore, I highly recommend reanalyzing the 16S rRNA sequencing data with an updated database that reflects current phyla. Otherwise, I would unfortunately find it difficult to accept this manuscript for publication, given the high impact and relevance of the journal, and despite the quality of the study's statistical and bioinformatics approaches.
Response: We thank the reviewer for this important observation, and we agree. As stated above, we re-analyzed and updated all of the microbiome results throughout the manuscript, including in the supplementary figures and datafiles.

- Comment 10. Materials and Methods general comment. Please ensure that the version of each program and pipeline used in the study is included. For example, lines 231 and 234 mention bioinformatics tools without version numbers. Please also include the version of all R packages.
Response: We have made these changes throughout the entire Materials and Methods section, now found in the main manuscript.

- Comment 11. Line 301. Why were parametric analyses such as ANOVA used? Did the data follow a normal distribution or were normalized?
Response: We thank the reviewer for their question regarding meeting assumptions of normality in ANOVA. In all cases, visual analysis of residuals did not indicate significant departure from normality. The inclusion of random effects in the linear mixed model further ensures that the non-independence of the repeated observations per worker were accounted for in the model. Additionally, the lme4 package implementation and the use of linear mixed models are known to be robust to departures from normality, we therefore believe that the use of ANOVA is appropriate. We included additional language to clarify these assumptions in the MATERIALS AND METHODS section (lines 941–943).

- Comment 12. Lines 318-336. In this section, beta diversity is said to have been analyzed with PCA. However, in line 512, NMDS is mentioned. Please review the analytical details in the methodology.
Response: We thank the reviewer for catching this discrepancy. We have updated the language, as we have used PCA for this analysis.

Results:

- Comment 13. Sections 1 and 2. The information provided in these sections, while interesting and very well-analyzed, is outdated due to the use of an outdated database, as noted in Comment 9. Therefore, I highly recommend reanalyzing the 16S rRNA sequencing data with an updated database reflecting new phyla classification, as the provided data relies on a 2017 database. For instance, the Prevotella genus has recently been reclassified into different genera, potentially altering the study's results (lines 205-207).
Response: We thank the reviewer for this important observation, and we agree. As stated above, we re-analyzed and updated all of the microbiome results throughout the manuscript.

- Comment 14. Figure 2d. The colors of the lines are difficult to distinguish for positive or negative associations. Please revise the colors or increase the line thickness.
Response: We thank the reviewer for this comment. We increased the line thickness of the network graphs to distinguish the positive and negative associations in Figure 2d.

- Comment 15. Line 233. Replace "flora" with "microbiota."
Response: We thank the reviewer for this comment. We have replaced 'flora' with 'microbiota' in the revised manuscript (line 234).

- Comment 16. Section "The skin microbiome becomes unstructured and dominated by enteric and environmental microbes during on-farm work." Consider adding a reference to Figure 2e related to Swine in this section and explaining if there is a relationship with the predominant taxa in T2 or T3.
Response: We thank the reviewer for noting this. We omitted referencing to results of the swine network, and we have done that in the resubmission (lines 231–237).

- Comment 17. Line 268. Regarding "up to 44%," this figure applies only to ICEs and TEs, while plasmids and IS reach only about 24%. Please revise to avoid misinterpretation.
Response: We thank the reviewer for catching this obscure wording. We clarified this in the resubmission (lines 278–281).

- Comment 18. Lines 269-270. In Figure S6, significance ($p < 0.05$) is indicated for both viruses and prophages.
Response: We thank the reviewer for catching this mistake, we clarified the wording here as we meant to describe that shifts in viruses and prophages between the collection phases T1 and T2 were more modest by comparison to the other MGEs. (lines 283–284).

- Comment 19. Line 292. Please include the antimicrobial classes for which vga genes confer resistance.
Response: We made additional notes of the antimicrobial classes for which the vga genes confer resistance (lines 313–314).

- Comment 20. Lines 349-358. It is noteworthy that while Figure 3 shows the highest abundance and prevalence of mecA in worker samples from T1 and T3, there is a significant increase in Figure 4a for mecA in T2, which remains elevated in T3. Could you provide an explanation or further discuss this?
Response: We thank the reviewer for noting this. Two sources contribute to these differences between the two figures, including threshold of detection as well as method of analysis. Figure 3 shows normalized abundance and prevalence results on a per-sample basis using a uniquely high-threshold gene detection approach (i.e. >99.99% gene fraction cutoff in the read alignment step) applied to a subset of the resistome that we term: medically important ARGs. Conversely, Figure 4a shows results of differential abundance for the overall resistome, detected by read alignment with a default >80% gene fraction cutoff. The results in Figure 4a are based on an aggregate of counts across all samples at the ARG group level, with model inclusion for both sequencing and worker biometric covariates.

- Comment 21. MAGs section and Figure 5c. Please include the updated phylum taxonomy.
Response: We thank the reviewer for noting this, we made the changes in this figure as well as in the corresponding text (1st paragraph of MAG section). We also checked the other names to ensure that all other phyla are up to date with the current List of Prokaryotic names with Standing in Nomenclature.

- Comment 22. Line 429. Please include the abbreviation of GTDB.
Response: We thank the reviewer for this comment. We clarified the GTDB acronym in the revised

manuscript (1st paragraph of MAG section).

- Comment 23. Figure S10 is not referenced in the manuscript. Please check if it is needed or should be removed.
Response: This figure was not referenced and was not needed. Hence, we removed this figure from the supplementary files portion of the manuscript.

Discussion:

- Comment 24. Line 578. Replace "his" with "this."
Response: This change was made in line 666.

- Comment 25. General comment. I would recommend including information on the limitations and benefits of metagenomic enrichment, as the mobilome may include MGEs that remain undescribed, which could lead to their underrepresentation. Additionally, it would be beneficial to highlight the advantages of combining 16S with metagenomic enrichment, given that the more common approach is metagenomic analysis with taxonomic assignment from these sequences, without prior enrichment.
Response: We thank the reviewer for their comment, we addressed it in our response to comment 8.

Reviewer #2: Slizovskiy et al. studied the skin microbiome, resistome and mobilome compositions of swine workers at three stages of their working day, before, during and at the end. They conclude that showering as part of their biosecurity measures at the swine farm effectively contains potential pathogen and AMR spread. The study is of interest and too little is known on occupational exposure effects in the flow of pathogen and AMR spread.

However, I do have a few more generic and more specific questions/remarks related to the study, the text and figures.

Generic remarks;

1) The statement of restoring the microbiome to baseline (i.e. line 60 and 536) seems too strong as the authors themselves state for instance at page 14 that the microbiome composition is not restored. Furthermore there are significant differences between T1 and T3 (lines 200-204).
Response: Thank you for highlighting the need for precision in describing our findings. We have revised the wording in the abstract (lines 55–56) to clarify that while there is evidence of partial reversion towards baseline, complete restoration was not observed—as also detailed on page 14. Additionally, we have acknowledged the significant differences between T1 and T3 and adjusted the discussion accordingly. We appreciate your input, which has helped us better reflect the nuances of our results.

2) I am also a bit puzzled about the actual exposure and what the authors measured; skin microbiome at various places on the body (fig 1) which were pooled to one sample. Is protective clothing (as imaged in fig 1) not part of biosecurity? How come the workers get microbial exposure at most likely completely covered regions of their body? In line 554 they write full exposure of the skin. Could another confounder be the result of all these microbial changes at T2? This should be made more clear in text and discussion (and/or change fig1 image as that might be misleading?).
Response: We thank the reviewer for their comment. We clarified the sampling procedure and protocol in

lines (lines 605–618) of the discussion section. Our sampling procedure was strategically conducted to account for a number of possible sources of microbial variation. This includes skin biogeography and its variable impact on how taxa will change over the workday, as well as different regions that may have either higher or lower exposures to the swine farm environment. We purposely chose skin sampling sites that represent a range of exposures (both clothing-covered- and uncovered- skin). This was done as there have been no studies to suggest the existence of optimal skin sites for microbiome sampling in occupational contexts. Our approach is highly conservative. We reason that since microbiome alterations were detected despite sampling skin locations that are less exposed to the farm environment, this shift represents a likely under-estimate and enhances the credibility and robustness of the results.
We agree that biosecurity measures such as protective clothing may impact worker microbiomes throughout the workday. However, the effect of PPE during the workday cannot be discerned in this study design as all workers are required to wear the same type of PPE, for approximately the same duration, and used approximately in the same way. We made this more clear in the text (lines 593–603). Therefore, we believe that Figure 1 accurately represents our sampling design and does not introduce any confusion regarding the methodology.

3) Linked to remark #2; The authors do not see a clear link with for instance environmental samples or pig skin microbiomes. So where did all the stuff in T2 come from? In their discussion it might be worthwhile to also address the potential influence of pig feces as a source? Knowing the source can help to minimize spillover events. (Ilya–I thought we discussed this at length, but perhaps we eliminated some of the language for brevity for the other journals?)
Response: We thank the reviewer for their comment. We expanded our discussion of possible sources that may contribute to alteration to worker skin microbiomes, including the influence of swine feces (lines 564–567). However, again, we note that in this preliminary investigation, precise source-tracking of bacteria was not possible as we now discuss in the updated discussion section (lines 570–577).

4) Some discussion seems to be required on other routes (besides skin) for pathogen and AMR transmission/exposure. The nasal cavity is known to be a direct reflection of the (air) environment, while oral/ingestion might result in colonization of the airways and gut. Thereby providing a continuous source of "baseline" exposure. This might also explain some of the strange observations from Figure 3, for instance the high increase at T2 samples for Vga and Sul1 which do not seem to be highly abundant at high prevalence in non-T2 samples.
Response: We appreciate the reviewer’s valuable insight regarding alternative exposure routes such as the nasal cavity and oral ingestion. Although our study primarily focused on skin microbiome changes rather than transmission per se, we recognize that these other pathways may contribute to baseline exposures and could help contextualize some of the observations in Figure 3, such as the notable increases in Vga and Sul1 at T2. We discuss in lines 622–624 the influence of prior exposures that may have contributed to the worker’s baseline microbiome. As stated in comment #3, we also now reference relevant literature on shifts in the nasal and gastrointestinal microbiomes and acknowledge their potential roles in AMR exposure / burden. We hope this addition adequately addresses your perspective while staying within the scope of our study.

5) The authors used ANOSIM to do GROUP comparisons of microbial compositions and later PERMANOVAs. Since the same worker is sampled 3 times we have repeated measures for each individual. ANOSIM as rank based group comparison seems not the optimal choice. Why not utilizing PERMANOVA for abundance over time using stratification/blocking approach on the individual IDs? This has been applied for 16S qPCR count data (lme). The same accounts for alpha diversity metrics and a glmm approach.
Response: We thank the reviewer for raising this point. We performed this additional step using the adonis2 function in the Vegan package during our re-analysis of the ordination PERMANOVAs with individual worker IDs as strata for repeated measures. We updated these results in the manuscript (lines 270–299), and described this step in the methods (lines 973–975). We note that this updated approach did not alter the conclusions reached in comparison to those using the approach from the original analysis.

6) Why did the authors first use 16S rRNA amplicon sequencing to determine microbial diversity at mostly genus taxonomic level while later shotgun-metagenomics and assembly are utilized of the same samples at strain resolution? Authors describe that similar results for microbial diversity were obtained at lines 427-428.

Response: We thank the reviewer for raising this question. We made further clarifications on this point in the main manuscript discussion section (lines 654–660).

7) Why are "only" 40 samples from phase 1 of the laborOme project utilized and not the complete set of phase 2 (2 facilities, each 40 workers, 3 timepoints)?
Response: We apologize to the reviewer for any confusion. We included all of the samples in our analysis for this project. There is no phase 2 and no additional samples were collected beyond those we reported on. We removed any indication of this in our Github page.

8) The authors measured microbial load using 16S rRNA-based qPCR. Most of the AMR and mobilome data are analysed as compositional data. Even though compositions might answer some of the questions for absence/presence, the actual AMR load is an important metric as described in literature. This AMR load might be inferred using the compositional data and the qPCR data.
Response: We agree with this point. We performed additional quantitative and statistical analyses of ARG/MGE burdens, with updated details in the methods (lines 980–986) and results (lines 270–277 / lines 291–299).

More specific comments.

1) References #17 #18 and #53 seem incomplete.
Response: We thank the reviewer for catching these. We made updates to these references.

2) Line 215; filtering conditions. Isn't this filtering way to strict? This will exclude (near) zero or <100 taxon counts taxa which actually may be the most interesting ones. Was the 100 cutoff set and used on even depth rarefied data? Would using a prevalence cutoff not being better?
Response: We applied a strict cut-off by taxonomic count, as this is a recommendation provided in the documentation of the SPIEC-EASI tool. While the taxa at near zero (<100 taxon counts) may be of interest, we nevertheless are wary of the possibility of spurious associations that can be reached from these rare taxa, even with the use of robust network approaches for compositional datasets and appropriate normalization techniques as used in the manuscript. Consequently, we consider the implementation of this stringent filtering approach a necessary measure to ensure the reliability of our analyses. However, we agree with the reviewer regarding the use of a prevalence cutoff. In our re-analysis of the microbiome with the updated SILVA reference database, we applied a 10% cutoff (i.e. >10% / >1 sample prevalence) in addition to the counts cutoff. This was updated in the methods section of the resubmission (lines 1025).

3) Line 221 significant; stats are missing.
Response: We thank the reviewer for noting this. We altered the language in this sentence, as there are no specific statistical tests that we are referencing.

4) Line 233 flora; There is no such think as intestinal flora. Revise to microbiota.
Response: We have replaced 'flora' with 'microbiota' in the revised manuscript across multiple areas of the manuscript.

5) At many places in the document the symbols for beta etc got replaced with a square.
Response: Thank you for bringing this to our attention. After reviewing our document we believe this could have happened during the conversion step to PDF in the submission portal for journal review. We ensured that this does not appear in the resubmission stage.

6) Line 286; One sample T2 was quite different. What was done with this sample (taken out)? How does this effect the previously mentioned >100 counts cutoff applied?
Response: We believe that the reviewer may have been mistaken in this comment. We did not apply a 100 count cutoff to the analysis of the medically important ARGs referenced in line 286 of the original submission. In this analysis, we applied a near 100% nucleotide alignment match requirement in order for the pre-specified clinically important ARGs to be considered 'present' in a given sample.

7) Technical issue metaphlan; It has been described and known that especially early versions of metaphlan

(the database) was strong bias to annotation for human microbiome-derived data. The authors used version 3, albeit the tool maintainers claim version 4 should have addressed this issue for non-human hosts.
Response: We agree with the reviewer's suggestion and we performed this re-analysis. We repeated our analysis using updated versions of MetaPhlAn and StrainPhlAn subsequent analysis. We updated our results of this analysis throughout the manuscript. We note that overall, this update did not alter the major conclusions reached.

8) Line 398+ and 403-405; How sure is the suggested link between psychrobacter and its actual presence in the data?
Response: Since we performed our analysis with two different versions of MetaPhlAn / StrainPhlAn, we believe we have confirmed the presence of Psychrobacter in our data. This finding parallels Psychrobacter identified via 16S rRNA sequencing, and Psychrobacter MAGs were also recovered. This consistency supports the more than likely preponderance of Psychrobacter's presence in the various samples.

9) Line 536-537 biosecurity measures; Looking at the presented data improving protective clothing might be an important consideration which is not discussed.
Response: We thank the reviewer for this comment. We increased the discussion of PPE and biosecurity measures in the discussion section (e.g. lines 593–603)

10) Line 544 process controls; Explanation required.
Response: We thank the reviewer for this comment. We provided detailed examples of process controls in the revised manuscript (lines 551–554).

Editorial Manager®

Cookie Preference Center

We use cookies which are necessary to make our site work. We may also use additional cookies to analyze, improve and personalize our content and your digital experience. For more information, see our Cookie Policy.

You may choose not to allow some types of cookies. However, blocking some types may impact your experience of our site and the services we are able to offer. See the different category headings below to find out more or change your settings.

Allow all

Manage Consent Preferences

Strictly Necessary Cookies

Always active

These cookies are necessary for the website to function and cannot be switched off in our systems. They are usually only set in response to actions made by you which amount to a request for services, such as setting your privacy preferences, logging in or filling in forms. You can set your browser to block or alert you about these cookies, but some parts of the site will not then work.

Functional Cookies

These cookies enable the website to provide enhanced functionality and personalisation. They may be set by us or by third party providers whose services we have added to our pages. If you do not allow these cookies then some or all of these services may not function properly.

Performance Cookies

These cookies allow us to count visits and traffic sources so we can measure and improve the performance of our site. They help us to know which pages are the most and least popular and see how visitors move around the site.

Targeting Cookies

These cookies may be set through our site by our advertising partners. They may be used by those companies to build a profile of your interests and show you relevant adverts on other sites. If you do not allow these cookies, you will experience less targeted advertising.

Cookie List

Clear

Apply Cancel

Consent Leg.Interest

Confirm my choices

Powered by onetrust
