# Supplementary material for: Reducing skin microbiome exposure impacts through swine farm biosecurity
Source: Gigascience. 2025 Jul 26;14:giaf062. doi: 10.1093/gigascience/giaf062 (PMC12810053; doi:10.1093/gigascience/giaf062)
Supplement: giaf062_Authors_Response_To_Reviewer_Comments_revision_1 [file giaf062_authors_response_to_reviewer_comments_revision_1.pdf]

Author's Response To Reviewer Comments

RE: GIGA-D-24-00356
Reducing Skin Microbiome Exposure Impacts Through Swine Farm Biosecurity
Ilya B. Slizovskiy; Tara N Gaire; Peter M Ferm; Carissa A Odland; Scott A Dee; Joel Nerem; Jonathan E Bravo; Christina Boucher; Noelle R Noyes

Dear Editor and Reviewers,

We thank you for your thorough and thoughtful reviews of our manuscript "Reducing Skin Microbiome Exposure Impacts Through Swine Farm Biosecurity" (GIGA-D-24-00356). We appreciate the results of the second round of review, and have included here our comments and changes commensurate with the reviewer's minor revision comments.
Thank you again for your time and insightful suggestions.

Editorial comment- Please also ensure that your revised manuscript conforms to the journal style, which can be found in the Instructions for Authors on the journal homepage. (For example, please structure the abstract, with sections "Background", "Results", "Conclusions").
Response: We thank the editor for their comment. We revisited the formatting instructions and ensured that our manuscript conforms to the GigaScience publishing format standards, including the changes made to the abstract section. Additionally, we added all in-text references and citations related to the GigaDB archival process of supporting datasets and sequencing information.

Reviewer #1:The article titled "Reducing Skin Microbiome Exposure Impacts Through Swine Farm Biosecurity" has improved considerably. I appreciate the authors' efforts in addressing the comments, particularly updating the 16S rRNA database. I have only a few minor suggestions that should be addressed. Below are the detailed comments:

General comments:
- Comment 1. Despite the improvements, there are still instances (e.g., line 78) where there is no space between the citation and the preceding text. This is especially noticeable in the Materials and Methods section. Please review thoroughly and ensure proper spacing throughout.
Response: We noted this issue, and made changes to in-text citation spacing throughout the manuscript. We believe we have made corrections throughout all facets of the manuscript now.

Abstract:
- Comment 2. Line 47. Include the abbreviation for "United States" for further use in Line 52.
Response: We made this change in the abstract section.

Introduction:
- Comment 3. Line 81. Please replace "Actinobacteria" and "Bacterioidetes" with the updated phylum names "Actinomycetota" and "Bacteroidota," respectively.
Response: We have made this suggested change, in ~line 80, first paragraph of the Introduction.

Results:
- Comment 4. Figure 5c. Ensure that the updated phylum name for "Proteobacteria" is reflected here (i.e., "Pseudomonadota"), as previously recommended for a fully up-to-date classification.
Response: We have made the suggested change to this figure.

Reviewer #2: The authors have done a thorough job to update their analysis with more recent versions of databases, tools and statistics. It was good to see that beside some smaller changes the major results did not change. This is also comforting for earlier studies that used those tools and databases.

While reviewing the revision;

- The answers to queries and suggestions seem to be incorporated well in the revised text and figures.

- I noticed the strain level addition of S suis detection on page 10. Since the authors have the strain level information, it is important to mention which so-called serotypes were detected since not all S suis serotypes are zoonotic.
Response: We thank the reviewer for raising the issue of S. suis serotyping. We added a clarification that detection of S. suis strains is based on the gene marker strategy in lines 379–380, page 12. Given the approach used, we do not find it possible to comment on differentiation of S. suis serotypes, as the gene marker approach is not conducive to such analysis.

em Editorial Manager®

Cookie Preference Center

We use cookies which are necessary to make our site work. We may also use additional cookies to analyze, improve and personalize our content and your digital experience. For more information, see our Cookie Policy.

You may choose not to allow some types of cookies. However, blocking some types may impact your experience of our site and the services we are able to offer. See the different category headings below to find out more or change your settings.

Allow all

Manage Consent Preferences

Strictly Necessary Cookies

Always active
These cookies are necessary for the website to function and cannot be switched off in our systems. They are usually only set in response to actions made by you which amount to a request for services, such as setting your privacy preferences, logging in or filling in forms. You can set your browser to block or alert you about these cookies, but some parts of the site will not then work.

Functional Cookies

These cookies enable the website to provide enhanced functionality and personalisation. They may be set by us or by third party providers whose services we have added to our pages. If you do not allow these cookies then some or all of these services may not function properly.

Performance Cookies

These cookies allow us to count visits and traffic sources so we can measure and improve the performance of our site. They help us to know which pages are the most and least popular and see how visitors move around the site.

Targeting Cookies

These cookies may be set through our site by our advertising partners. They may be used by those companies to build a profile of your interests and show you relevant adverts on other sites. If you do not allow these cookies, you will experience less targeted advertising.

Cookie List

Clear

Apply Cancel

Consent Leg.Interest

Confirm my choices

Powered by onetrus
