# Supplementary material for: Reducing skin microbiome exposure impacts through swine farm biosecurity
Source: Gigascience. 2025 Jul 26;14:giaf062. doi: 10.1093/gigascience/giaf062 (PMC12810053; doi:10.1093/gigascience/giaf062)
Supplement: giaf062_Authors_Response_To_Reviewer_Comments_revision_2 [file giaf062_authors_response_to_reviewer_comments_revision_2.pdf]

Author's Response To Reviewer Comments

Additional comment: co-author Dr. Alejandro Kimball was not listed in the latest email regarding this submission. He is listed in the "Manuscript Data" section and on the title page of the manuscript, so we wanted to confirm that he will be listed as a co-author in the submitted manuscript.

- At the beginning of the manuscript you link to supporting material that's stored on Google drive (lines 42/42). Is this data also stored in GigaDB? If this data is already stored in GigaDB , the links can be deleted here. If this is additional supporting data, please upload the files directly to Editorial Manager instead, marked as "supporting files" (e.g. as PDF).
RESPONSE: We removed these links, as they are no longer relevant.

-Please add ORCIDs for all authors to the title page, where available. We have the following on our files: ORCID iDs: Ilya Slizovskiy [0000-0002-0758-5510]; Tara N Gaire [0000-0003-1805-8051]; Peter M Ferm [0009-0008-8873-0185]; Carissa A Odland [0000-0003-0787-4985]; Scott A Dee [0000-0001-8691-3887]; Joel Nerem; Jonathan E Bravo [0000-0002-4481-8766]; Christina Boucher [0000-0001-9509-9725]; Noelle R Noyes [0000-0001-6149-1008];
RESPONSE: We added all available ORCiDs to the title page

- Please upload all figures as separate files to Editorial Manager rather than inserting them in the WORD doc.
RESPONSE: We removed all figures from the WORD doc and instead uploaded them as standalone PNG images. We also have these available as .AI and .SVG files if those are preferable.

em Editorial Manager®

Cookie Preference Center

We use cookies which are necessary to make our site work. We may also use additional cookies to analyze, improve and personalize our content and your digital experience. For more information, see our Cookie Policy.

You may choose not to allow some types of cookies. However, blocking some types may impact your experience of our site and the services we are able to offer. See the different category headings below to find out more or change your settings.

Allow all

Manage Consent Preferences

Strictly Necessary Cookies

Always active

These cookies are necessary for the website to function and cannot be switched off in our systems. They are usually only set in response to actions made by you which amount to a request for services, such as setting your privacy preferences, logging in or filling in forms. You can set your browser to block or alert you about these cookies, but some parts of the site will not then work.

Functional Cookies

These cookies enable the website to provide enhanced functionality and personalisation. They may be set by us or by third party providers whose services we have added to our pages. If you do not allow these cookies then some or all of these services may not function properly.

Performance Cookies

These cookies allow us to count visits and traffic sources so we can measure and improve the performance of our site. They help us to know which pages are the most and least popular and see how visitors move around the site.

Targeting Cookies

These cookies may be set through our site by our advertising partners. They may be used by those companies to build a profile of your interests and show you relevant adverts on other sites. If you do not allow these cookies, you will experience less targeted advertising.

Cookie List

Clear

Apply Cancel

Consent Leg.Interest

Confirm my choices

Powered by onetrust
