# Supplementary material for: Reducing skin microbiome exposure impacts through swine farm biosecurity
Source: Gigascience. 2025 Jul 26;14:giaf062. doi: 10.1093/gigascience/giaf062 (PMC12810053; doi:10.1093/gigascience/giaf062)
Supplement: giaf062_Reviewer_1_Report_Original_Submission — Oscar Menc఺-Ares, Ph.D -- 10/29/2024 [file giaf062_reviewer_1_report_original_submission.pdf]

Reviewer Report

Title: Reducing Skin Microbiome Exposure Impacts Through Swine Farm Biosecurity

Version: Original Submission **Date:** 10/29/2024

Reviewer name: Oscar MencÃ-a-Ares, Ph.D

Reviewer Comments to Author:

Revision Note:
The article titled "Reducing Skin Microbiome Exposure Impacts Through Swine Farm Biosecurity" is very interesting and well-structured. It provides solid methodological information and results, written in a detailed manner that makes it easy to follow. However, there are some major comments that need to be addressed, particularly related to the 16S rRNA sequencing database used for taxonomic assignment (Silva 132), which is from 2017 and does not reflect the important taxonomic changes made since then. Therefore, I highly recommend reanalyzing these data with an updated database. Below are the detailed comments.
General comments:
- Comment 1. Please include spaces between citations and the text. Review this structure throughout the manuscript.
- Comment 2. Greek letters (alpha, beta) appear as squares in several places throughout the text. Please review.
Abstract:
- Comment 3. Line 51. Replace "U.S." with "United States".
Introduction:
- Comment 4. Section title. Please replace "MAIN TEXT" with "INTRODUCTION".
- Comment 5. Line 90. The phylum Proteobacteria is currently categorized as Pseudomonadota.
- Comment 6. Line 97. Please include a citation for studies tracking daily animal exposure and on-site worker behavior.
- Comment 7. Line 102. Include the abbreviation for "United States" for further use throughout the manuscript.
Materials and methods - Supplementary Information
- Comment 8. Materials and Methods general comment. I understand the relevance of using a target-enriched metagenomic library preparation for AMR and MGE, given their low representation in samples. However, many studies assessing the mobilome and resistome conduct sequencing without prior enrichment, which reduces bias in the analysis, especially in uncharacterized MGEs. Additionally, this approach created the need for 16S rRNA sequencing analysis, which used an outdated database; this would not have been necessary with direct metagenomic sequencing without enrichment, enabling taxonomic assignment using updated tools such as Kraken. Furthermore, MetaPhlAn3 and MetaBAT2 were used for detecting taxonomic markers and MAGs binning, respectively, which introduces some bias due to AMR and MGE marker enrichment. Please explain the reasoning behind this approach and its potential limitations. Wouldn't it be possible to assign taxonomy directly from the metagenomic

approach?
- Comment 9. The Silva database 132 is updated from December 13, 2017, meaning the taxonomic information is quite outdated, with significant changes in taxonomic classifications in the last seven years, such as phylum renaming. Therefore, I highly recommend reanalyzing the 16S rRNA sequencing data with an updated database that reflects current phyla. Otherwise, I would unfortunately find it difficult to accept this manuscript for publication, given the high impact and relevance of the journal, and despite the quality of the study's statistical and bioinformatics approaches.
- Comment 10. Materials and Methods general comment. Please ensure that the version of each program and pipeline used in the study is included. For example, lines 231 and 234 mention bioinformatics tools without version numbers. Please also include the version of all R packages.
- Comment 11. Line 301. Why were parametric analyses such as ANOVA used? Did the data follow a normal distribution or were normalized?
- Comment 12. Lines 318-336. In this section, beta diversity is said to have been analyzed with PCA. However, in line 512, NMDS is mentioned. Please review the analytical details in the methodology.
Results:
- Comment 13. Sections 1 and 2. The information provided in these sections, while interesting and very well-analyzed, is outdated due to the use of an outdated database, as noted in Comment 9. Therefore, I highly recommend reanalyzing the 16S rRNA sequencing data with an updated database reflecting new phyla classification, as the provided data relies on a 2017 database. For instance, the Prevotella genus has recently been reclassified into different genera, potentially altering the study's results (lines 205-207).
- Comment 14. Figure 2d. The colors of the lines are difficult to distinguish for positive or negative associations. Please revise the colors or increase the line thickness.
- Comment 15. Line 233. Replace "flora" with "microbiota."
- Comment 16. Section "The skin microbiome becomes unstructured and dominated by enteric and environmental microbes during on-farm work." Consider adding a reference to Figure 2e related to Swine in this section and explaining if there is a relationship with the predominant taxa in T2 or T3.
- Comment 17. Line 268. Regarding "up to 44%," this figure applies only to ICEs and TEs, while plasmids and IS reach only about 24%. Please revise to avoid misinterpretation.
- Comment 18. Lines 269-270. In Figure S6, significance ($p < 0.05$) is indicated for both viruses and prophages.
- Comment 19. Line 292. Please include the antimicrobial classes for which vga genes confer resistance.
- Comment 20. Lines 349-358. It is noteworthy that while Figure 3 shows the highest abundance and prevalence of mecA in worker samples from T1 and T3, there is a significant increase in Figure 4a for mecA in T2, which remains elevated in T3. Could you provide an explanation or further discuss this?
- Comment 21. MAGs section and Figure 5c. Please include the updated phylum taxonomy.
- Comment 22. Line 429. Please include the abbreviation of GTDB.
- Comment 23. Figure S10 is not referenced in the manuscript. Please check if it is needed or should be removed.
Discussion:
- Comment 24. Line 578. Replace "his" with "this."
- Comment 25. General comment. I would recommend including information on the limitations and

benefits of metagenomic enrichment, as the mobilome may include MGEs that remain undescribed, which could lead to their underrepresentation. Additionally, it would be beneficial to highlight the advantages of combining 16S with metagenomic enrichment, given that the more common approach is metagenomic analysis with taxonomic assignment from these sequences, without prior enrichment.

Methods

Are the methods appropriate to the aims of the study, are they well described, and are necessary controls included? Choose an item.

Conclusions

Are the conclusions adequately supported by the data shown? Choose an item.

Reporting Standards

Does the manuscript adhere to the journal's guidelines on minimum standards of reporting? Choose an item.

Choose an item.

Statistics

Are you able to assess all statistics in the manuscript, including the appropriateness of statistical tests used? Choose an item.

Quality of Written English

Please indicate the quality of language in the manuscript: Choose an item.

Declaration of Competing Interests

Please complete a declaration of competing interests, considering the following questions:

- Have you in the past five years received reimbursements, fees, funding, or salary from an organisation that may in any way gain or lose financially from the publication of this manuscript, either now or in the future?
- Do you hold any stocks or shares in an organisation that may in any way gain or lose financially from the publication of this manuscript, either now or in the future?
- Do you hold or are you currently applying for any patents relating to the content of the manuscript?
- Have you received reimbursements, fees, funding, or salary from an organization that holds or has applied for patents relating to the content of the manuscript?
- Do you have any other financial competing interests?

- Do you have any non-financial competing interests in relation to this paper?

If you can answer no to all of the above, write 'I declare that I have no competing interests' below. If your reply is yes to any, please give details below.

I declare that I have no competing interests

I agree to the open peer review policy of the journal. I understand that my name will be included on my report to the authors and, if the manuscript is accepted for publication, my named report including any attachments I upload will be posted on the website along with the authors' responses. I agree for my report to be made available under an Open Access Creative Commons CC-BY license (http://creativecommons.org/licenses/by/4.0/). I understand that any comments which I do not wish to be included in my named report can be included as confidential comments to the editors, which will not be published.

Choose an item.

To further support our reviewers, we have joined with Publons, where you can gain additional credit to further highlight your hard work (see: https://publons.com/journal/530/gigascience). On publication of this paper, your review will be automatically added to Publons, you can then choose whether or not to claim your Publons credit. I understand this statement.

Yes Choose an item.
