# Supplementary material for: Reducing skin microbiome exposure impacts through swine farm biosecurity
Source: Gigascience. 2025 Jul 26;14:giaf062. doi: 10.1093/gigascience/giaf062 (PMC12810053; doi:10.1093/gigascience/giaf062)
Supplement: giaf062_Reviewer_2_Report_Original_Submission — Alex Bossers -- 11/29/2024 [file giaf062_reviewer_2_report_original_submission.pdf]

Reviewer Report

Title: Reducing Skin Microbiome Exposure Impacts Through Swine Farm Biosecurity

Version: Original Submission **Date:** 11/29/2024

Reviewer name: Alex Bossers

Reviewer Comments to Author:

Slizovskiy et al. studied the skin microbiome, resistome and mobilome compositions of swine workers at three stages of their working day, before, during and at the end. They conclude that showering as part of their biosecurity measures at the swine farm effectively contains potential pathogen and AMR spread. The study is of interest and too little is known on occupational exposure effects in the flow of pathogen and AMR spread.
However, I do have a few more generic and more specific questions/remarks related to the study, the text and figures.
Generic remarks;
1) The statement of restoring the microbiome to baseline (i.e. line 60 and 536) seems too strong as the authors themselves state for instance at page 14 that the microbiome composition is not restored. Furthermore there are significant differences between T1 and T3 (lines 200-204).
2) I am also a bit puzzled about the actual exposure and what the authors measured; skin microbiome at various places on the body (fig 1) which were pooled to one sample. Is protective clothing (as imaged in fig 1) not part of biosecurity? How come the workers get microbial exposure at most likely completely covered regions of their body? In line 554 they write full exposure of the skin. Could another confounder be the result of all these microbial changes at T2? This should be made more clear in text and discussion (and/or change fig1 image as that might be misleading?).
3) Linked to remark #2; The authors do not see a clear link with for instance environmental samples or pig skin microbiomes. So where did all the stuff in T2 come from? In their discussion it might be worthwhile to also address the potential influence of pig feces as a source? Knowing the source can help to minimize spillover events.
4) Some discussion seems to be required on other routes (besides skin) for pathogen and AMR transmission/exposure. The nasal cavity is known to be a direct reflection of the (air) environment, while oral/ingestion might result in colonization of the airways and gut. Thereby providing a continuous source of "baseline" exposure. This might also explain some of the strange observations from Figure 3, for instance the high increase at T2 samples for Vga and Sul1 which do not seem to be highly abundant at high prevalence in non-T2 samples.
5) The authors used ANOSIM to do GROUP comparisons of microbial compositions and later PERMANOVAs. Since the same worker is sampled 3 times we have repeated measures for each individual. ANOSIM as rank based group comparison seems not the optimal choice. Why not utilizing PERMANOVA for abundance over time using stratification/blocking approach on the individual IDs? This has been applied for 16S qPCR count data (lme). The same accounts for alpha diversity metrics and a glmm approach.

6) Why did the authors first use 16S rRNA amplicon sequencing to determine microbial diversity at mostly genus taxonomic level while later shotgun-metagenomics and assembly are utilized of the same samples at strain resolution? Authors describe that similar results for microbial diversity were obtained at lines 427-428.
7) Why are "only" 40 samples from phase 1 of the laborOme project utilized and not the complete set of phase 2 (2 facilities, each 40 workers, 3 timepoints)?
8) The authors measured microbial load using 16S rRNA-based qPCR. Most of the AMR and mobilome data are analysed as compositional data. Even though compositions might answer some of the questions for absence/presence, the actual AMR load is an important metric as described in literature. This AMR load might be inferred using the compositional data and the qPCR data.
More specific comments.
1) References #17 #18 and #53 seem incomplete.
2) Line 215; filtering conditions. Isn't this filtering way to strict? This will exclude (near) zero or <100 taxon counts taxa which actually may be the most interesting ones. Was the 100 cutoff set and used on even depth rarefied data? Would using a prevalence cutoff not being better?
3) Line 221 significant; stats are missing.
4) Line 233 flora; There is no such think as intestinal flora. Revise to microbiota.
5) At many places in the document the symbols for beta etc got replaced with a square.
6) Line 286; One sample T2 was quite different. What was done with this sample (taken out)? How does this effect the previously mentioned >100 counts cutoff applied?
7) Technical issue metaphlan; It has been described and known that especially early versions of metaphlan (the database) was strong bias to annotation for human microbiome-derived data. The authors used version 3, albeit the tool maintainers claim version 4 should have addressed this issue for non-human hosts.
8) Line 398+ and 403-405; How sure is the suggested link between psychrobacter and its actual presence in the data?
9) Line 536-537 biosecurity measures; Looking at the presented data improving protective clothing might be an important consideration which is not discussed.
10) Line 544 process controls; Explanation required.

Methods

Are the methods appropriate to the aims of the study, are they well described, and are necessary controls included? Choose an item.

Conclusions

Are the conclusions adequately supported by the data shown? Choose an item.

Reporting Standards

Does the manuscript adhere to the journal's guidelines on [minimum standards of reporting?] Choose an item.

Choose an item.

Statistics

Are you able to assess all statistics in the manuscript, including the appropriateness of statistical tests used? Choose an item.

Quality of Written English

Please indicate the quality of language in the manuscript: Choose an item.

Declaration of Competing Interests

Please complete a declaration of competing interests, considering the following questions:

- Have you in the past five years received reimbursements, fees, funding, or salary from an organisation that may in any way gain or lose financially from the publication of this manuscript, either now or in the future?
- Do you hold any stocks or shares in an organisation that may in any way gain or lose financially from the publication of this manuscript, either now or in the future?
- Do you hold or are you currently applying for any patents relating to the content of the manuscript?
- Have you received reimbursements, fees, funding, or salary from an organization that holds or has applied for patents relating to the content of the manuscript?
- Do you have any other financial competing interests?
- Do you have any non-financial competing interests in relation to this paper?

If you can answer no to all of the above, write 'I declare that I have no competing interests' below. If your reply is yes to any, please give details below.

I declare that I have no competing interests.

I agree to the open peer review policy of the journal. I understand that my name will be included on my report to the authors and, if the manuscript is accepted for publication, my named report including any attachments I upload will be posted on the website along with the authors' responses. I agree for my report to be made available under an Open Access Creative Commons CC-BY license (http://creativecommons.org/licenses/by/4.0/). I understand that any comments which I do not wish to be included in my named report can be included as confidential comments to the editors, which will not be published.

Choose an item.

To further support our reviewers, we have joined with Publons, where you can gain additional credit to further highlight your hard work (see: https://publons.com/journal/530/gigascience). On publication of this paper, your review will be automatically added to Publons, you can then choose whether or not to claim your Publons credit. I understand this statement.

Yes Choose an item.
